# Beyond 2‑Oxazolidinones: Access to *N*‑Unsubstituted Six- and Seven-Membered Analogues Using CO_2_ and Superbase Catalyst

**DOI:** 10.1021/acs.orglett.5c01410

**Published:** 2025-06-03

**Authors:** Jere K. Mannisto, Johannes Heikkinen, Jukka Puumi, Aleksi Sahari, Pablo Ramírez Veliz, Timo Repo

**Affiliations:** Department of Chemistry, 3835 University of Helsinki, P.O. Box 55, A.I. Virtasen aukio 1, 00014 Helsinki, Finland

## Abstract

We report a straightforward synthesis of *N*-unsubstituted five-, six-, and seven-membered cyclic carbamates from amino alcohols and CO_2_, employing user-friendly propanephosphonic acid anhydride (T3P) as a dehydrating agent. Demanding substrates, such as amino alcohols bearing tertiary hydroxyl groups, cyclize in good yields under mild conditions, providing access to fused and spirocyclic carbamates. Mechanistic studies indicate that the amino alcohols react with CO_2_, forming poorly soluble salts, which are then solubilized by catalytic superbase 1,8-diazabicyclo[5.4.0]­undec-7-ene (DBU).

## Graphical abstract

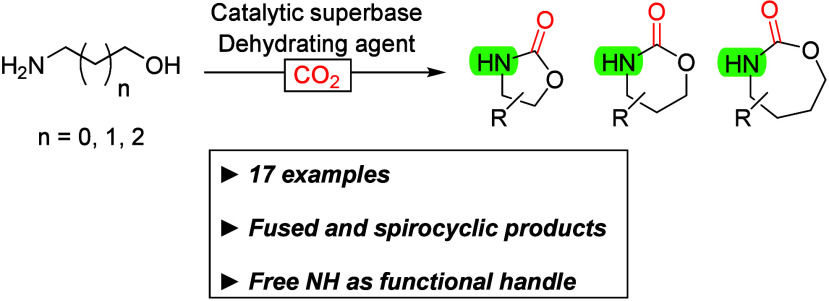

Nitrogen-containing heterocycles are a key structural motif in many pharmaceuticals and agrochemicals.^1–3^ Cyclic carbamates, a subgroup of *N*-heterocycles, have been widely utilized as peptide bond surrogates due to their pharmacokinetic improving properties and good metabolic stability.^4,5^ Indeed, cyclic carbamates of varying ring-sizes are present in several important pharmaceuticals (Scheme 1A).^6–8^

**Scheme 1:**
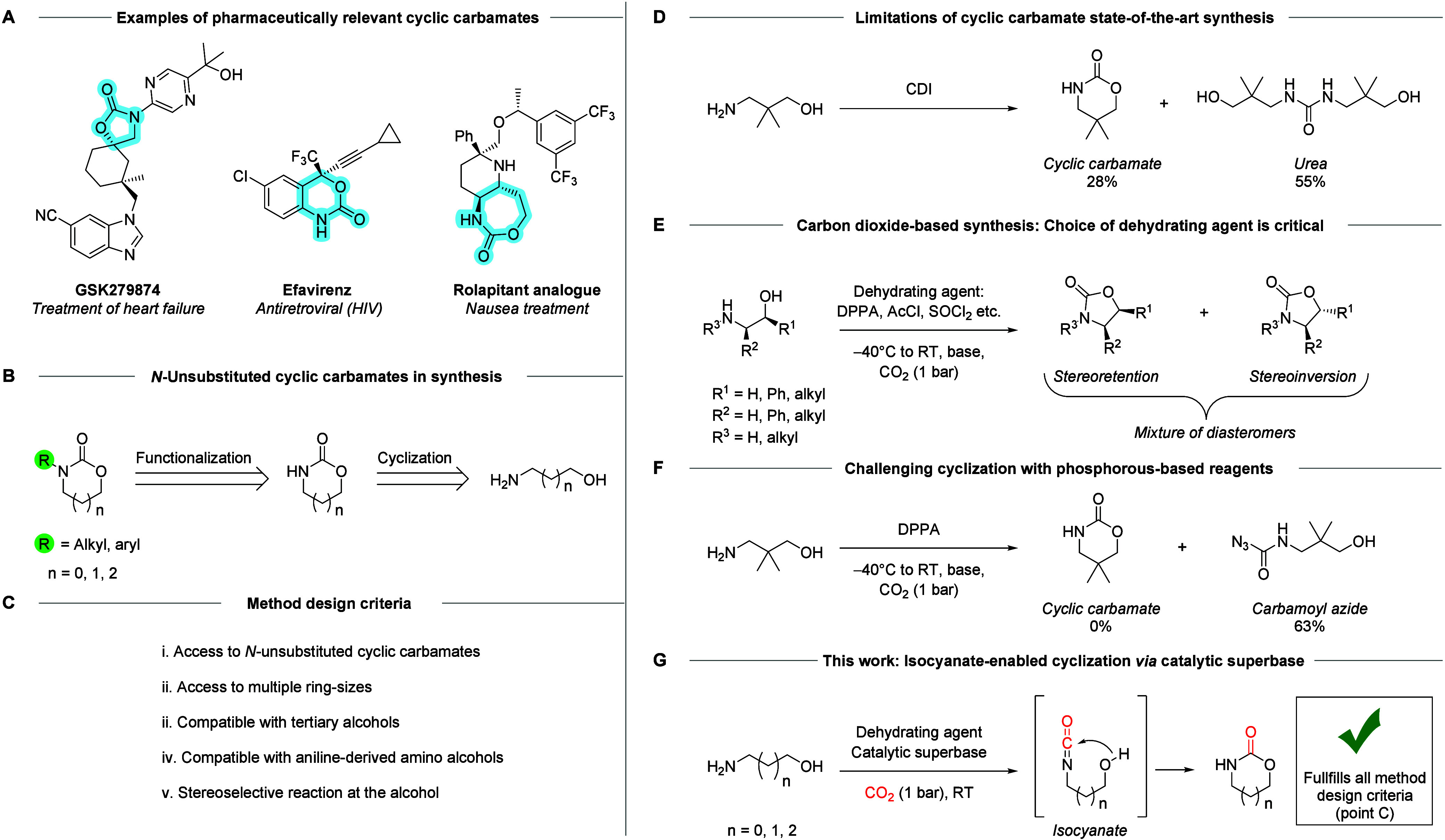
Applications and Synthesis of *N*-Unsubstituted Cyclic Carbamates

Cyclic carbamates bearing an unsubstituted nitrogen atom may be the desired end-product, but this nitrogen atom also provides a synthetic handle in the development of pharmaceutical libraries, a strategy realized in the development of anacetrapib and GSK279874 (Scheme 1B).^6,9^ In this context, the easily accessed and versatile amino alcohols are an excellent class of starting materials.^10,11^

To access the above-mentioned privileged structures from amino alcohols, we identified several important criteria that the ideal cyclization reaction should fulfill (Scheme 1C). The method should be able to produce *N*-unsubstituted cyclic carbamates (i) and offer flexibility in ring-size (ii). We had previously found difficulty in cyclizing amino alcohols bearing tertiary alcohols, which would be important to access spirocyclic structures such as GSK279874 (iii).^12^ Similarly, we wanted less nucleophilic anilines to be compatible to obtain fused structures such as Efavirenz (iv).^13^ Finally, considering that GSK279874 and Efavirenz bear chiral centers at the alcohol, we wanted cyclization to proceed in a stereoselective manner (v).

The state-of-the-art route to cyclic carbamates employs carbonyl diimidazole (CDI) as a carbonyl source (Scheme 1D).^14^ While this method readily provides five-membered rings, *i.e.*, 2-oxazolidinones, diminished yields are observed for the corresponding six-membered cyclic carbamates due to competing urea formation. This is even more pronounced for seven-membered cyclic carbamates, which are only obtained in trace amounts.^14^

Considering alternative carbonyl sources, we identified CO_2_ as a safe, abundant, and readily available carbonyl source, which would give it a competitive edge over conventional phosgene-based reagents.^15–21^ Synthetic methods yielding 2-oxazolidinones from CO_2_ are well established.^22,23^ In contrast, there are significantly fewer examples of CO_2_-based methods to access 6-membered cyclic carbamates.^24–30^ These methods require engineered starting materials, particularly for *N*-unsubstituted products.^26–30^ Examples of seven-membered carbamates from CO_2_ are rare.^31,32^ To the best of our knowledge, there exists no CO_2_-based method to *N*-unsubstituted 7-membered cyclic carbamates. This bias in chemical space likely stems from the fact that *N*-substitution significantly accelerates cyclization,^33^ whereas increasing ring-size beyond five will have the opposite effect.^31,34^ In other words, formation of *N*-unsubstituted six- or seven-membered cyclic carbamates is challenging.

Seeking to overcome the challenges listed above, we were attracted to a study where amino alcohols were cyclized in the presence of CO_2_ and a dehydrating agent under cryogenic conditions (Scheme 1E).^35^ While diastereomeric mixtures were obtained, the transformation showed good stereoselectivity when using phosphorus-based reagents, particularly diphenylphosphoryl azide (DPPA).

However, the carbamoyl azide intermediate did not always cyclize (Scheme 1F).^35^ This lead us to consider highly reactive isocyanates, which were generated from amino alcohols and CO_2_ under dehydrative Mitsunobu conditions,^36^ using tributylphosphine, an air-sensitive, toxic (LD_50_ 750 mg kg^–1^), and pyrophoric liquid with a nauseating odor. The reaction scope centered on 2-oxazolidinones, with only one example of a six-membered cyclic carbamate. Nevertheless, we saw the potential of an isocyanate-based strategy in driving challenging cyclizations.

Herein we report synthesis of valuable *N*-unsubstituted 2-oxazolidinones and elusive six- and seven-membered cyclic carbamates using a nontoxic dehydrating agent (Scheme 1G).

We began our study by selecting T3P as the dehydrating agent due to its low toxicity (LD_50_ > 2000 mg kg^–1^) and an established use on industrial scale (Table 1, graphic).^37^ The oxidized form of T3P (T3PO^–2^) is highly water-soluble and is therefore easily removed, in contrast to conventionally used phosphine oxides.^37^ We noted that T3P was recently used in a CO_2_-based synthesis of *N-*carboxyanhydrides.^38^ Amino alcohol **1a** was selected as the model compound for its challenging tertiary alcohol. An initial experiment produced traces of product **2a** when T3P was added in one portion to a homogeneous mixture of Et_3_N and superbase DBU. The latter was included for its well-known propensity to promote the reactivity of amines with CO_2_.^15^ Gratifyingly, the yield of **2a** improved when T3P was added slowly over 4 h (Table 1, entry 1). An increased amount of DBU (entry 2) or heterogeneous Cs_2_CO_3_ both improved the yield of **2a** (entry 3). Counterintuitively, combining DBU and Cs_2_CO_3_ led to a decrease in yield (entry 4). This effect persisted when DBU was lowered to 50 mol % (entry 5), but lowering to 20 mol % did improve the overall yield (entry 6). Increasing the amount of T3P gave the best yield (entry 7). Control experiments showed that removing DBU decreased the yield (entry 8). Increasing the amount of DBU was detrimental (entry 9), as was excluding the CO_2_ atmosphere (entry 10).

**Table 1:**
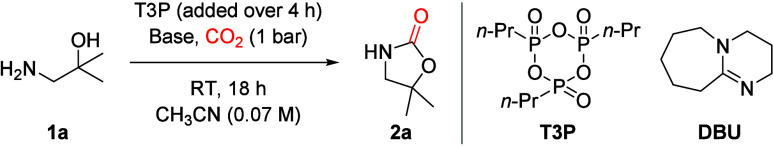
Optimization Studies^a^

Entry	Base (equiv)	T3P (equiv)	Yield (%)
1	Et_3_N (3.0) + DBU (1.0)	1.3	25
2	DBU (2.0)	1.3	49
3	Cs_2_CO_3_ (3.0)	1.3	63
4	Cs_2_CO_3_ (3.0) + DBU (1.0)	1.3	59
5	Cs_2_CO_3_ (3.0) + DBU (0.5)	1.3	56
6	Cs_2_CO_3_ (3.0) + DBU (0.2)	1.3	76
7	Cs_2_CO_3_ (3.0) + DBU (0.2)	2.0	100
8	Cs_2_CO_3_ (3.0)	2.0	86
9	Cs_2_CO_3_ (3.0) + DBU (1.0)	2.0	68
10^b^	Cs_2_CO_3_ (3.0) + DBU (0.2)	2.0	<5

a Yield (0.5 mmol scale) determined as the normalized GC-MS area of product **2a** in relation to mesitylene (1 equiv).

b Under Ar instead of a CO_2_ atmosphere.

With the optimized conditions in hand, we explored the substrate scope (Scheme 2). 2-Oxazolidinones **2a**–**2e** were obtained in good yields, with **2c** isolated as a single diasteromer. In the case of aniline **1d**, a slower addition of T3P over 12 h was found to improve the yield.

**Scheme 2:**
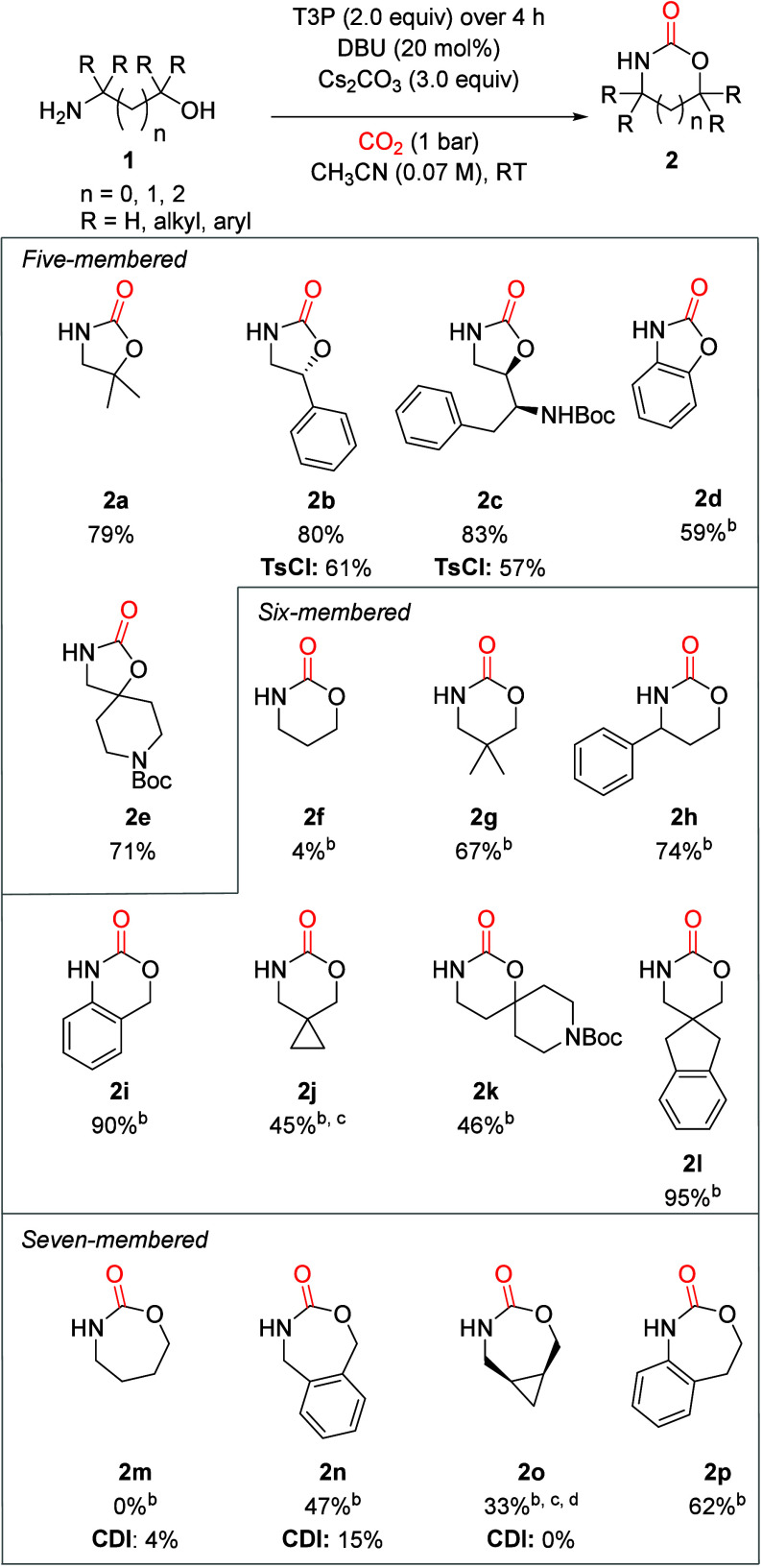
Substrate Scope of T3P-Mediated Cyclization of Amino Alcohols and CO_2_ ^a^

a Isolated yields on 4.0 mmol scale.
b T3P added over 12 h.
c Dual addition.
d Racemic, (1*S*, 7*R*)-enantiomer is shown.

Proceeding to six-membered cyclic carbamates, we initially obtained only traces of **2f**, despite extensive reoptimization (SI section 5.1). It was observed that the solution turned cloudy and the finely dispersed Cs_2_CO_3_ powder formed hard lumps when the 1,3-amino alcohol **1f** was added, irrespectively of the reagent addition order. Lumping did not occur for the studied 1,2-amino alcohols **1a–1e**. We hypothesized that when **1f** reacts with CO_2_, the forming intermediate, likely cesium carbamate, precipitates out and causes lumping. Indeed, when 1,3-amino alcohols **1g–1l** bearing lipophilic substituents were surveyed, the corresponding carbamates **2g–2l** were obtained in good yields. Adding T3P over 12 h was beneficial, likely due to the low solubility of **1g–1l** derived carbamates in solution. Under standard conditions, **2j** was obtained in only 30% yield, likely due to cyclopropane decomposition. Gratifyingly, high dilution conditions using dual addition of **1j** and T3P increased the yield of **2j** to 45%.

Finally, we studied exotic seven-membered cyclic carbamates. Unsubstituted **2m** was not observed, whereas substituted carbamates **2n–2p** were obtained in modest to good yields. No urea side products were observed, inherent to the CDI-based method.^14^ To benchmark our results, standard CDI-mediated synthesis of products **2m–2o** was attempted, but the yields were significantly reduced. This underlines that our CO_2_-based method exceeds the synthetic state-of-the-art.

The T3P-mediated dehydration is remarkably clean, with only minor impurities that can in most cases be removed by trituration or by filtering the crude product through a plug of silica. In contrast, a myriad of side-products is observed by GC-MS when TsCl is used as the dehydrating agent. Using TsCl instead of T3P resulted in lower yields of **2b** and **2c**. In this regard, T3P has significant advantages over TsCl.^12,39,40^

It was next studied whether the reaction mechanism proceeded via retention or inversion of configuration (Scheme 3A). Using enantiopure amino alcohol **1b**, cyclized product **2b** was analyzed by chiral HPLC and found to be >99 *ee* with retention of configuration. The absence of stereoinversion is consistent with an isocyanate intermediate.^36^ Although *N*-unsubstituted carbamates were our target, we were curious how *N*-methylated **1q** would behave. We observed **2q** as the exclusive diastereomer (Scheme 3B). Complete retention was surprising to us, since inversion product **2r** was the expected product under the previously reported Mitsunobu conditions (Scheme 3C).^36^ Our results suggest that T3P is a potent activator of the carbamate group, leading to rapid dehydration (Scheme 3A) or nucleophilic attack (Scheme 3B).

**Scheme 3:**
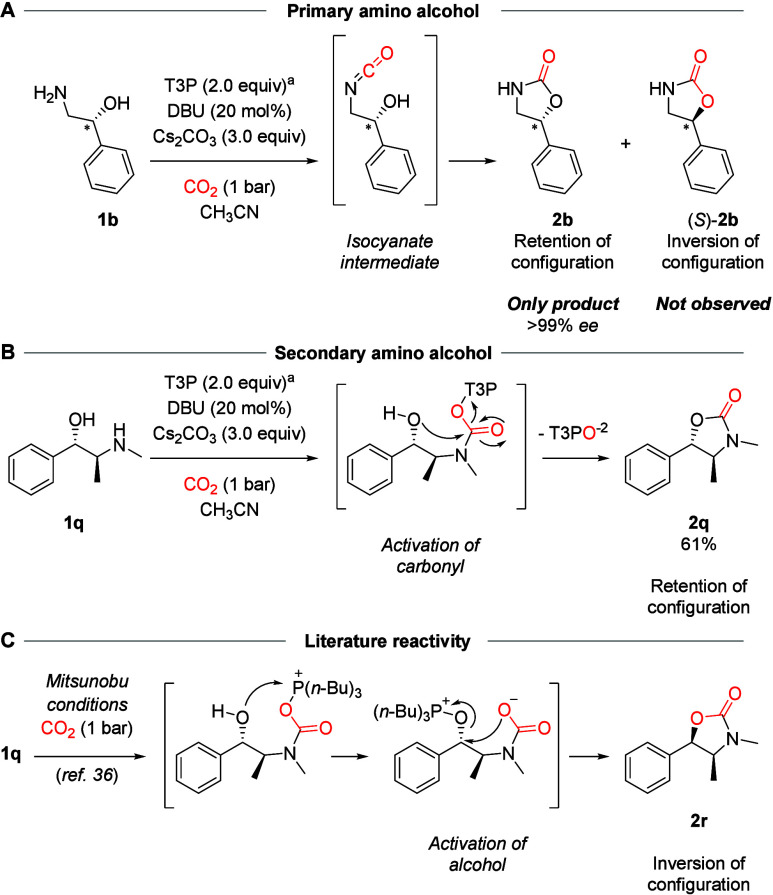
Studies on the Stereochemical Outcome of the Reaction

a T3P added over 4 h.

The role of DBU was next studied by *in situ* NMR using amino alcohol **1h**, Cs_2_CO_3_, and mesitylene as an internal standard (SI section 2.1). Under CO_2_ without DBU, only 11% of carboxylated species (carbamate anion **3h**) were observed in solution. In contrast, with 20 mol % DBU present, carboxylated species were present in 49%. These experiments suggest DBU has a bifunctional role in shifting the solution equilibrium from unreacted **1h** to the carbamate salt **3h**, and resolubilizing precipitated **3h**.

Having studied solution behavior, we turned to analyzing the solid phase by FTIR (SI section 3.1). No peaks characteristic of ammonium species (RNH_3_^+^) were observed, suggesting the Cs^+^ salt of **3h** as the major species.

Based on the above-mentioned observations, we propose the following mechanism (Scheme 4). In the presence of CO_2_, amino alcohol **1h** rapidly forms carbamic acid **3h′**.^41^ This species partially reacts with Cs_2_CO_3_ and precipitates out as salt **A**.^42,43^ In a separate pathway, **3h′** forms a salt with DBU, which further reacts with salt **A**, forming well-soluble dimer **B**. This species is stabilized by coordination to Cs^+^ and hydrogen bonds between anionic **3h** and protonated DBU. Similar dimers are known to be stabilized by coordination to Cs^+^ or a protonated superbase.^13^ The catalytic cycle begins with dimer **B** reacting with T3P, releasing salt **A** and forming carbamyl phosphate **C**. Base-mediated elimination forms isocyanate **2h′** and the mixed DBU/Cs salt **D**. Isocyanate **2h′** cyclizes to product **2h**. The DBU/Cs salt **D** undergoes anion exchange with two equivalents of salt **A**. The first equivalent precipitates less soluble Cs_2_T3PO and the second equivalent regenerates dimer **B**. Overall, it seems the reaction between dimer **B** and T3P is the rate-determining step, since it was necessary to slowly add T3P. Adding all T3P at once led to lower yields. Presumably this was due to slow carbonate-mediated decomposition of T3P, a known reactivity of oxygen-nucleophiles.^44^ The influence of amino alcohol structural features on the reaction outcome is further discussed in SI section 1.1.

**Scheme 4:**
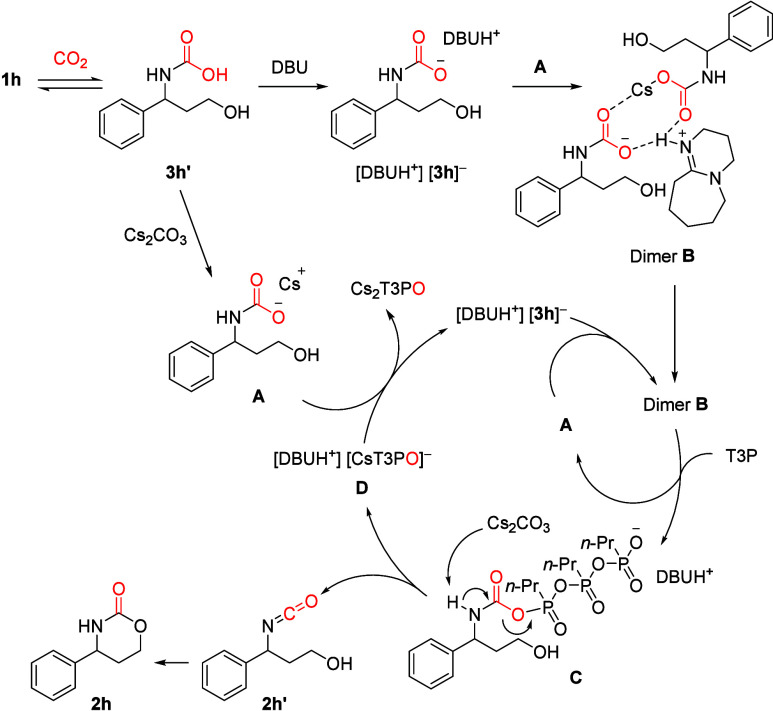
Proposed Mechanism, Where a DBU-Stabilized Carbamate Anion Dimer (B) is Dehydrated by T3P to an Isocyanate

To conclude, we have developed a CO_2_-based method to synthesize *N*-unsubstituted five-, six-, and seven-membered cyclic carbamates, including bicyclic systems. The reaction is very clean, stereospecific, and high yielding, even when using amino alcohols with sterically demanding tertiary alcohols. Rare seven-membered cyclic carbamates are obtained in low-to-moderate yields, yet which are superior to state-of-the-art CDI-based syntheses. Mechanistic work indicates that the *in situ* formed carbamate salts are dehydrated to isocyanates using T3P. Catalytic DBU has a bifunctional role in shifting the solution equilibrium to more reactive and soluble species.

## Supplementary Material



## Data Availability

The data underlying this study are available in the published article and its Supporting Information.

## Notes

The electronic Supporting Information provides general experimental information, *in situ* NMR experiments, FTIR spectra, experimental procedures, compound characterization, chiral HPLC results, and NMR spectra (PDF)

## References

[1] Heravi M. M., Zadsirjan V. (2020). Prescribed Drugs Containing Nitrogen Heterocycles:
An Overview. RSC Adv..

[2] Lamberth C. (2013). Heterocyclic
Chemistry in Crop Protection. Pest Manag Sci..

[3] Vitaku E., Smith D. T., Njardarson J. T. (2014). Analysis
of the Structural Diversity,
Substitution Patterns, and Frequency of Nitrogen Heterocycles among
U.S. FDA Approved Pharmaceuticals. J. Med. Chem..

[4] Ghosh A. K., Brindisi M. (2015). Organic Carbamates
in Drug Design and Medicinal Chemistry. J. Med.
Chem..

[5] Vacondio F., Silva C., Mor M., Testa B. (2010). Qualitative Structure-Metabolism
Relationships in the Hydrolysis of Carbamates. Drug Metab Rev..

[6] Brooks C. A., Barton L. S., Behm D. J., Eidam H. S., Fox R. M., Hammond M., Hoang T. H., Holt D. A., Hilfiker M. A., Lawhorn B. G., Patterson J. R., Stoy P., Roethke T. J., Ye G., Zhao S., Thorneloe K. S., Goodman K. B., Cheung M. (2019). Discovery
of GSK2798745: A Clinical Candidate for Inhibition of Transient Receptor
Potential Vanilloid 4 (TRPV4). ACS Med. Chem.
Lett..

[7] Pierce M. E., Parsons R. L., Radesca L. A., Lo Y. S., Silverman S., Moore J. R., Islam Q., Choudhury A., Fortunak J. M. D., Nguyen D., Luo C., Morgan S. J., Davis W. P., Confalone P. N., Chen C. Y., Tillyer R. D., Frey L., Tan L., Xu F., Zhao D., Thompson A. S., Corley E. G., Grabowski E. J. J., Reamer R., Reider P. J. (1998). Practical Asymmetric Synthesis of
Efavirenz (DMP 266), an HIV-1 Reverse Transcriptase Inhibitor. J. Org. Chem..

[8] Xiao, D. ; Palani, A. ; Wang, C. ; Tsui, H.-C. ; Shih, N.-Y. ; Reichard, G. A. . Fused Ring NK1 Antagonists, US7041682B2; USA, 2005.

[9] Smith C. J., Ali A., Hammond M. L., Li H., Lu Z., Napolitano J., Taylor G. E., Thompson C. F., Anderson M. S., Chen Y., Eveland S. S., Guo Q., Hyland S. A., Milot D. P., Sparrow C. P., Wright S. D., Cumiskey A. M., Latham M., Peterson L. B., Rosa R., Pivnichny J. V., Tong X., Xu S. S., Sinclair P. J. (2011). Biphenyl-Substituted
Oxazolidinones as Cholesteryl Ester Transfer Protein Inhibitors: Modifications
of the Oxazolidinone Ring Leading to the Discovery of Anacetrapib. J. Med. Chem..

[10] Ager D. J., Prakash I., Schaad D. R. (1996). 1,2-Amino
Alcohols and Their Heterocyclic
Derivatives as Chiral Auxiliaries in Asymmetric Synthesis. Chem. Rev..

[11] Kochi T., Tang T. P., Ellman J. A. (2002). Asymmetric Synthesis of Syn- and
Anti-1,3-Amino Alcohols. J. Am. Chem. Soc..

[12] Niemi T., Fernández I., Steadman B., Mannisto J. K., Repo T. (2018). Carbon Dioxide-Based
Facile Synthesis of Cyclic Carbamates from Amino Alcohols. Chem. Commun..

[13] Mannisto J. K., Pavlovic L., Tiainen T., Nieger M., Sahari A., Hopmann K. H., Repo T. (2021). Mechanistic
Insights into Carbamate
Formation from CO 2 and Amines: The Role of Guanidine–CO 2
Adducts. Catal. Sci. Technol..

[14] Díaz D. J., Hylton K. G., McElwee-White L. (2006). Selective Catalytic Oxidative Carbonylation
of Amino Alcohols to Ureas. J. Org. Chem..

[15] Gabriele B., Della Ca’ N., Mancuso R., Veltri L., Ziccarelli I. (2023). Amidine–
and Guanidine–based Synthetic Methods for CO2 Capture and Utilization. Curr. Opin Green Sustain Chem..

[16] Kleij A. W. (2020). Advancing
Halide-Free Catalytic Synthesis of CO2-Based Heterocycles. Curr. Opin Green Sustain Chem..

[17] Qin Y., Cauwenbergh R., Pradhan S., Maiti R., Franck P., Das S. (2023). Straightforward Synthesis of Functionalized γ-Lactams Using
Impure CO2 Stream as the Carbon Source. Nat.
Commun..

[18] Sahoo P. K., Zhang Y., Das S. (2021). CO2-Promoted Reactions: An Emerging
Concept for the Synthesis of Fine Chemicals and Pharmaceuticals. ACS Catal..

[19] Ion A., Van Doorslaer C., Parvulescu V., Jacobs P., De Vos D. (2008). Green Synthesis
of Carbamates from CO2, Amines and Alcohols. Green Chem..

[20] Schilling W., Das S. (2018). CO2-Catalyzed/Promoted Transformation of Organic Functional Groups. Tetrahedron Lett.

[21] Schilling W., Das S. (2020). Transition Metal-Free Synthesis of
Carbamates Using CO 2 as the Carbon
Source. ChemSusChem.

[22] Yu B., He L. N. (2015). Upgrading Carbon Dioxide by Incorporation into Heterocycles. ChemSusChem.

[23] Wang L., Qi C., Xiong W., Jiang H. (2022). Recent Advances in Fixation of CO2
into Organic Carbamates through Multicomponent Reaction Strategies. Chin. J. Catal..

[24] Wang W., Fu Y., Li Y., Yao R., Liu L., Chang W., Li J. (2018). Ag­(i)-Catalyzed Solvent-Free
CO2 Capture with Homopropargylic Amines:
An Efficient Access to 1,3-Oxazinan-2-Ones. Org. Chem. Front..

[25] Brunel P., Monot J., Kefalidis C. E., Maron L., Martin-Vaca B., Bourissou D. (2017). Valorization
of CO2: Preparation of 2-Oxazolidinones
by Metal-Ligand Cooperative Catalysis with SCS Indenediide Pd Complexes. ACS Catal..

[26] Sun S., Zhou C., Yu J. T., Cheng J. (2019). Visible-Light-Driven
Palladium-Catalyzed Oxy-Alkylation of 2-(1-Arylvinyl)­Anilines by Unactivated
Alkyl Bromides and CO2: Multicomponent Reactions toward 1,4-Dihydro-2
H-3,1-Benzoxazin-2-Ones. Org. Lett..

[27] Xiong H., Wu X., Wang H., Sun S., Yu J. T., Cheng J. (2019). The Reaction
of O-Aminoacetophenone N-Tosylhydrazone and CO2 toward 1,4-Dihydro-2H-3,1-Benzoxazin-2-Ones. Adv. Synth Catal.

[28] Takeda Y., Okumura S., Tone S., Sasaki I., Minakata S. (2012). Cyclizative
Atmospheric CO 2 Fixation by Unsaturated Amines with T-BuOI Leading
to Cyclic Carbamates. Org. Lett..

[29] Veltri L., Amuso R., Vitale P., Chiacchio M. A., Benincasa C., Gabriele B. (2021). Synthesis of 1,3-Oxazine-2,4-Diones
by DBU-Catalyzed Incorporation of Carbon Dioxide into 3-Ynamides. J. CO2 Util..

[30] Li X., Benet-Buchholz J., Escudero-Adán E. C., Kleij A. W. (2023). Silver-Mediated
Cascade Synthesis of Functionalized 1,4-Dihydro-2H-Benzo-1,3-Oxazin-2-Ones
from Carbon Dioxide. Angew. Chem., Int. Ed..

[31] Niemi T., Perea-Buceta J. E., Fernández I., Hiltunen O. M., Salo V., Rautiainen S., Räisänen M. T., Repo T. (2016). A One-Pot
Synthesis of N-Aryl-2-Oxazolidinones and Cyclic Urethanes by the Lewis
Base Catalyzed Fixation of Carbon Dioxide into Anilines and Bromoalkanes. Chem. Eur. J..

[32] Shi W., Benet-Buchholz J., Kleij A. W. (2025). Catalytic Transformation of Carbon
Dioxide into Seven-Membered Heterocycles and Their Domino Transformation
into Bicyclic Oxazolidinones. Nat. Commun..

[33] Agami C., Couty F., Hamon L., Venier O. (1993). Chiral Oxazolidinones
from N-Boc Derivatives of β-Amino Alcohols. Effect of a N-Methyl
Substituent on Reactivity and Stereoselectivity. Tetrahedron Lett..

[34] Kaneti J., Kirby A. J., Koedjikov A. H., Pojarlieff I. G. (2004). Thorpe-Ingold
Effects in Cyclizations to Five-Membered and Six-Membered Rings Containing
Planar Segments. The Rearrangement of N(1)-Alkyl-Substituted Dihydroorotic
Acids to Hydantoinacetic Acids in Base. Org.
Biomol Chem..

[35] Paz J., Pérez-Balado C., Iglesias B., Muñoz L. (2010). Carbon Dioxide
as a Carbonylating Agent in the Synthesis of 2-Oxazolidinones, 2-Oxazinones,
and Cyclic Ureas: Scope and Limitations. J.
Org. Chem..

[36] Dinsmore C. J., Mercer S. P. (2004). Carboxylation and Mitsunobu Reaction of Amines to Give
Carbamates: Retention vs Inversion of Configuration Is Substituent-Dependent. Org. Lett..

[37] Magano J. (2022). Large-Scale
Amidations in Process Chemistry: Practical Considerations for Reagent
Selection and Reaction Execution. Org. Process
Res. Dev.

[38] Tran T. V., Shen Y., Nguyen H. D., Deng S., Roshandel H., Cooper M. M., Watson J. R., Byers J. A., Diaconescu P. L., Do L. H. (2022). N-Carboxyanhydrides Directly from
Amino Acids and Carbon Dioxide
and Their Tandem Reactions to Therapeutic Alkaloids. Green Chem..

[39] McGuire T. M., López-Vidal E. M., Gregory G. L., Buchard A. (2018). Synthesis
of 5- to 8-Membered Cyclic Carbonates from Diols and CO2: A One-Step,
Atmospheric Pressure and Ambient Temperature Procedure. J. CO2 Util..

[40] Hedrick J. L., Piunova V., Park N. H., Erdmann T., Arrechea P. L. (2022). Simple
and Efficient Synthesis of Functionalized Cyclic Carbonate Monomers
Using Carbon Dioxide. ACS Macro Lett..

[41] Kortunov P. V., Siskin M., Baugh L. S., Calabro D. C. (2015). In Situ Nuclear
Magnetic Resonance Mechanistic Studies of Carbon Dioxide Reactions
with Liquid Amines in Non-Aqueous Systems: Evidence for the Formation
of Carbamic Acids and Zwitterionic Species. Energy Fuels.

[42] Mannisto J. K., Sahari A., Lagerblom K., Niemi T., Nieger M., Sztanó G., Repo T. (2019). One-Step Synthesis of 3,4-Disubstituted
2-Oxazolidinones by Base-Catalyzed CO 2 Fixation and Aza-Michael Addition. Chem. Eur. J..

[43] Mannisto J. K., Pavlovic L., Heikkinen J., Tiainen T., Sahari A., Maier N. M., Rissanen K., Nieger M., Hopmann K. H., Repo T. (2023). N-Heteroaryl Carbamates
from Carbon Dioxide via Chemoselective Superbase
Catalysis: Substrate Scope and Mechanistic Investigation. ACS Catal..

[44] Davidson A., Foley D. A., Frericks-Schmidt H., Ruggeri S. G., Herman M., Lacasse S., Liu Y., McInturff E. L., Morris R., Mugheirbi N., Samas B., Sarkar A., Singer R. A., Witkos F., Yu S. (2021). PH-Dependent Degradation
of T3P-Related Byproducts. Org. Process Res.
Dev.

