# Supplementary material for: Beyond 2‑Oxazolidinones: Access to N‑Unsubstituted Six- and Seven-Membered Analogues Using CO2 and Superbase Catalyst
Source: Org Lett. 2025 Jun 3;27(23):5971–6. doi: 10.1021/acs.orglett.5c01410 (PMC12172046; doi:10.1021/acs.orglett.5c01410)
Supplement: Supplementary file 1 [file ol5c01410_si_001.pdf]

Supporting Information

Beyond 2-Oxazolidinones: Access to *N*-Unsubstituted Six- and Seven-Membered Analogues Using CO_2 and Superbase Catalyst

Jere K. Mannisto,*,[a] Johannes Heikkinen,[a] Jukka Puumi,[a] Aleksi Sahari,[a] Pablo Ramírez Veliz,[a] and Timo Repo*,[a]

[a] Department of Chemistry, University of Helsinki, P.O. Box 55, A.I. Virtasen aukio 1, 00014 Helsinki, Finland

E-mail: jere.mannisto@helsinki.fi, timo.repo@helsinki.fi

Contents

1. General Experimental Information S5

1.1 Additional Mechanistic Discussion..S6

2. In situ NMR Experiments S9

2.1 Discussion S9

2.2 Experimental Details S10

Spectral Characterization – Under Air S12

Spectral Characterization – Under CO_2 (without DBU) S12

Spectral Characterization – Under CO_2 (with DBU) S13

3. FTIR Spectra S28

3.1 Discussion S28

3.2 Experimental Details S28

Cs_2CO_3 (Control)...S30

Without DBU S31

With DBU S32

The Effect of DBU (Overlaid Spectra of With and Without DBU).................................S33

All Experiments Overlaid..S34

4. Experimental Procedures and Characterization Data of Amino Alcohols........................S35

Compound 1c S35

Compound 1e S35

Compound 1j S36

Compound S01..S37

Compound 1o S38

Compound 1l...S39

5. Experimental Procedures S41

5.1 General Optimization Details...S41

5.1 Attempted Re-optimization for Cyclic Carbamate 2f S41

5.4 Synthetic Procedures S44
6. Characterization Data of Isolated Carbamates S47
Compound 2a S47
Compound 2b S47
Compound 2c S48
Compound 2d S54
Compound 2e S54
Compound 2f S55
Compound 2g S55
Compound 2h S56
Compound 2i S56
Compound 2j S57
Compound 2k S58
Compound 2l S58
Compound 2m S59
Compound 2n S59
Compound 2o S60
Compound 2p S60
Compound 2q S61
7. Chiral HPLC Analysis S65
Compound 2b (R-enantiomer), synthesized using CDI S65
Compound 2b (S-enantiomer), synthesized using CDI S66
Compound 2b (Racemic) S67
Compound 2b (R-enantiomer), synthesized using T3P S68
Compound 2b (R-enantiomer), synthesized using TsCl S69
8. FTIR Spectra of Selected Cyclic Carbamates S70
Compound 2a S70

Compound 2b S71

Compound 2i S72

Compound 2l S73

Compound 2n S74

Compound 2o S75

9. NMR spectra of Isolated Amino Alcohols..S76

10. NMR Spectra of Isolated Carbamates...S88

11. References...S120

1. General Experimental Information

All reagents employed were at the highest commercially available purity levels and used as received, unless otherwise specified. Acetonitrile (ACN) and other solvents were dried on 3 Å activated molecular sieves. Deuterated solvents were purchased at the highest purity level. Carbon dioxide (CO_2) was purchased at the 4.5 grade and used as received.

The reactions were analyzed using an Agilent 6890N gas chromatograph (GC) equipped with an HP-5MS UI column. The detector was a quadrupole mass spectrometer (MS) that operated with electron ionization (EI). Enantioselectivity was measured using an Agilent 1200 series high performance liquid chromatograph (HPLC) with a Daicel CHIRALCEL OD-H 250 x 4.6 mm column. The HPLC run was performed at 25 °C temperature and the eluent was 30:70 IPA:hexane. The solvent flow rate was 1.0 ml/min and the product was detected at 210 nm. HPLC samples were diluted in 30:70 IPA:hexane solution with a final analyte concentration of 1 mg/ml. The samples were filtered through an HPLC filter before analysis.

The nuclear magnetic resonance (NMR) experiments (^{1}H, ^{13}C{^{1}H}) were performed on Bruker Advance Neo 400 (^{1}H-frequency 400 MHz) or Bruker Advance Neo 500 (^{1}H-frequency 500 MHz) spectrometer operating with the frequency, deuterated solvent, and at the temperature indicated in parentheses. Proton and carbon chemical shift values (δ) are reported in parts per million (ppm) downfield in relation to tetramethylsilane using the residual undeuterated solvent signal as a secondary internal standard ($CDCl_3$; δ_H = 7.26 ppm and δ_C= 77.16 ppm, DMSO-d_6; δ_H = 2.50 ppm and δ_C = 39.52 ppm, CD_3CN; δ_H = 1.94 ppm and δ_C= 1.32 ppm). The resonances are noted as follows: δ chemical shift in ppm (number of nuclei, multiplicity, assignment). Splitting patterns are denoted as s (singlet), d (doublet), t (triplet), q (quartet), ABq (AB quartet, two protons (A and B) coupled to each other), h(heptet), dd (doublet of doublets), dt (doublet of triplets), dq (doublet of quartets), m (multiplet), w (weak) or br (broad resonance). All ^{13}C{^{1}H} spectra were proton-decoupled. In case of signal overlap, 2D NMR experiments (HSQC and/or HMBC) were used as additional techniques for NMR signal assignments.

IR spectra were measured with an Alpha ATR-FTIR by Bruker and data was interpreted by comparing to literature values.[1] The employed microwave reactor was Anton Paar Monowave 450 using standard 10 ml (G10 Anton Paar) or 30 ml (G30 Anton Paar) vials with a Teflon-coated silicone septum snap cap. The reaction temperature was measured with an IR sensor (Monowave IR eye), which was calibrated with an internal temperature sensor (Ruby thermometer).

1.1 Additional Mechanistic Discussion

As was discussed in the introduction of the manuscript, *N*-substitution increases solubility and promotes cyclization of the carbamate salt.[2] Steric clash with the *N*-substituent shifts the equilibrium of conformers to the one where the activated alcohol is situated close to the nucleophilic carbamate anion (**Scheme S1A**).[3] This approach works well when the activated alcohol is unhindered, favoring S_N2 type reaction. However, when the activated alcohol is hindered, *i.e.*, tertiary, cyclization becomes challenging, as was observed in our previous work (**Scheme S1B**).[3]

In this work, we decided to pursue an alternative approach where the carbamate anion is transformed into an electrophilic species (isocyanate) instead of the alcohol (**Scheme S1C** and **S1D**). In the first scenario, no additional substituents are present on the carbon chain, which favors the unproductive conformation (**Scheme S1C**). In contrast, a fully substituted alcohol, *i.e.*, tertiary, favors the productive conformation, leading to facile cyclization (**Scheme S1D**). The additional substitution that promotes cyclization is known as the Thrope-Ingold effect (TIE), which has been recently applied in a similar system.[4] It should be noted that *N*-unsubstituted carbamates can be further functionalized, for example via arylation (**Scheme S1C** and **S1D**). [5]

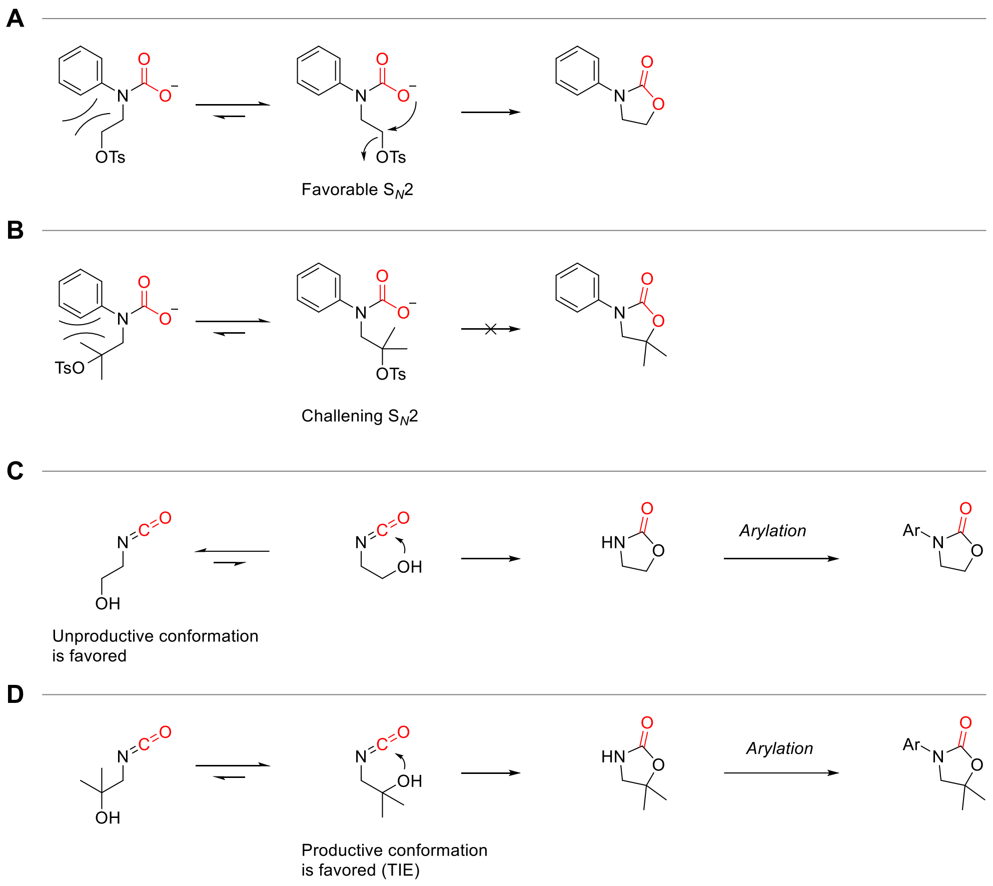

Scheme S1. An overview of various reaction design strategies in the synthesis of cyclic carbamates.

The solubility of intermediates is a key feature of this work, since substrates bearing lipophilic side-chains give higher yields, compare **2f** and **2l** (**Scheme 2**). TIE could be an alternative explanation for the divergent yields.[4] However, if this effect was dominant, geminal carbamates **2g** and **2j** should have been obtained in yields similar to **2l**.[6,7] Furthermore, let us consider product **2k**, which is formed from **1k** bearing tertiary alcohol. If TIE was the deciding factor in our reaction, we would expect product **2k** to form in a similar yield (ca 70-90%) as other high-yielding six-membered cyclic carbamates (**2g**, **2h**, **2i**, and **2l**). However, the yield of **2k** deviates from the rest, suggesting another phenomenon is also operational. During the synthesis of **2k**, it was noted that the reaction solution became quite cloudy, although not as severely as when synthesizing **2f**. This indicates that the solubility of the carbamate salt derived from **1k** is somewhat lower when compared to similar compounds.

Based on the above, four different scenarios are possible (**Chart S1**). Reaction intermediates with good solubility under the reaction conditions, but an unfavorable TIE, *i.e.*, a challenging cyclization, should be in quadrant A. However, none of the tested amino alcohols fell in this

category. Instead, in quadrant B, there are compounds with poor solubility and an unfavorable TIE. The corresponding carbamate salts of **1f** and **1m** have poor solubility, and their carbon chain is absent from substituents that may promote cyclization. Quadrant C houses compounds that have good solubility and that bear structural features that promote cyclization. Finally, quadrant D contains compounds, which are formed from the reaction intermediates with limited solubility, but this may be in part counterbalanced by favorable TIE, *i.e.*, facile cyclization.

Considering the critical role of DBU in this work, the development of novel superbases with increased lipophilicity would likely aid in solubilization of challenging intermediates, *e.g.*, the carbamate salts precursors of **2f** and **2m**.[8] In more broad terms, the use of more lipophilic superbases might drive the migration of challenging compounds from quadrant B to more favorable quadrant A, as well as poorly soluble compounds from quadrant D to well-behaved compounds in quadrant C.

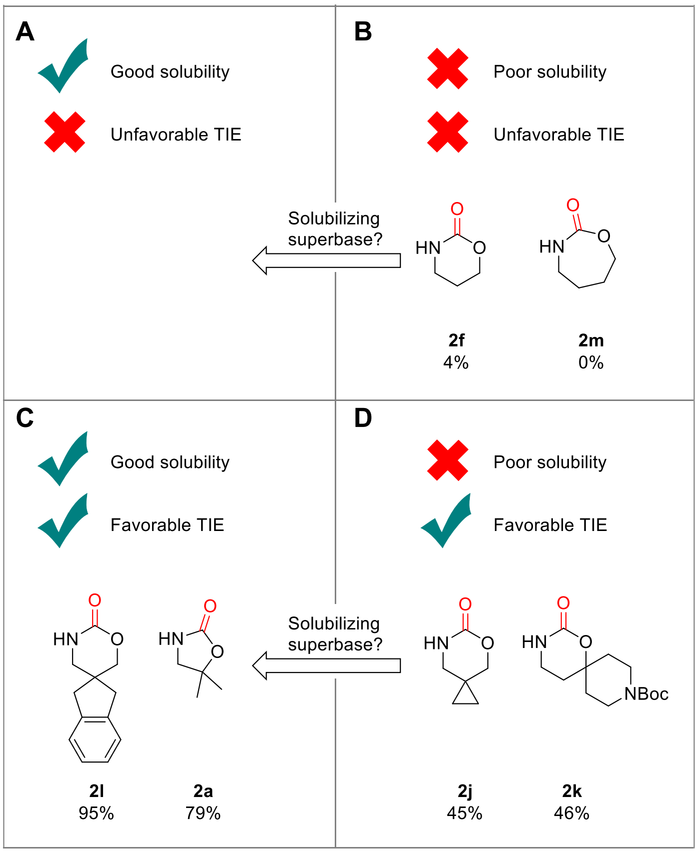

Chart S1. The behavior of amino alcohols under the reaction conditions is affected by their solubility and structural features. Future work with more lipophilic superbases may lead to an improved solubility of reaction intermediates.

2. In situ NMR Experiments

2.1 Discussion

The role of DBU was studied by *in situ* NMR using amino alcohol **1h**, Cs_2CO_3, and mesitylene as an internal standard (**Figure S1**, part **A**). Under air, the benzylic proton of **1h** was readily observed as a triplet by ^1H NMR (highlighted in green). Once the atmosphere was changed to CO_2 (1 bar), significant precipitation occurred (**Figure S2**). Unreacted **1h** remained as the major species, although its concentration was reduced to 33% (**Figure S1**, part **A**). In addition, a new signal assigned to carbamate ion **3h** appeared at 4.78 ppm (11%). The combined area of **1h** and **3h** indicates that the majority (*i.e.*, 56%) of **1h** precipitated out, most likely in the form of **3h**. The experiment was then repeated in the presence of catalytic DBU (20 mol%). Precipitation occurred to a much lesser extent (**Figure S2**). The triplet of **1h** was now observed as a small broad signal in 15% (**Figure 1A**). The major species had shifted to carboxylated **3h** (49%). It is known that DBU mediates carboxylation of amino alcohols in a 1:1 ratio.[9] This means the soluble fraction of **3h** should be the sum of baseline reactivity (11%) and DBU concentration (20%), for a total of 31%, which deviates from the observed 49%. However, assuming DBU stabilizes **3h** in a 1:2 ratio, a total 51% is expected, which aligns with the observed. These experiments suggest DBU has a bifunctional role in shifting the solution equilibrium from unreacted **1h** to the carbamate salt **3h**, and resolubilizing precipitated **3h**.

The spectral assignment of **1h** and **3h** was confirmed by HSQC. Under CO_2 and in absence of DBU, the main species **1h** (in blue/red, **Figure S1**, part **B**) is identical to the experiment under air (**Figure S1**, part **C**). In contrast, with DBU present, the HSQC spectrum (in green/purple, Figure **1B**) appears vastly different to that of the experiment under air (**Figure 1**, part **C**), suggesting the original species **1h** is nearly fully consumed. This is supported by the characteristic benzylic crosspeak (4.05; 54.4 ppm) migrating in presence of DBU (4.78; 52.0 ppm).

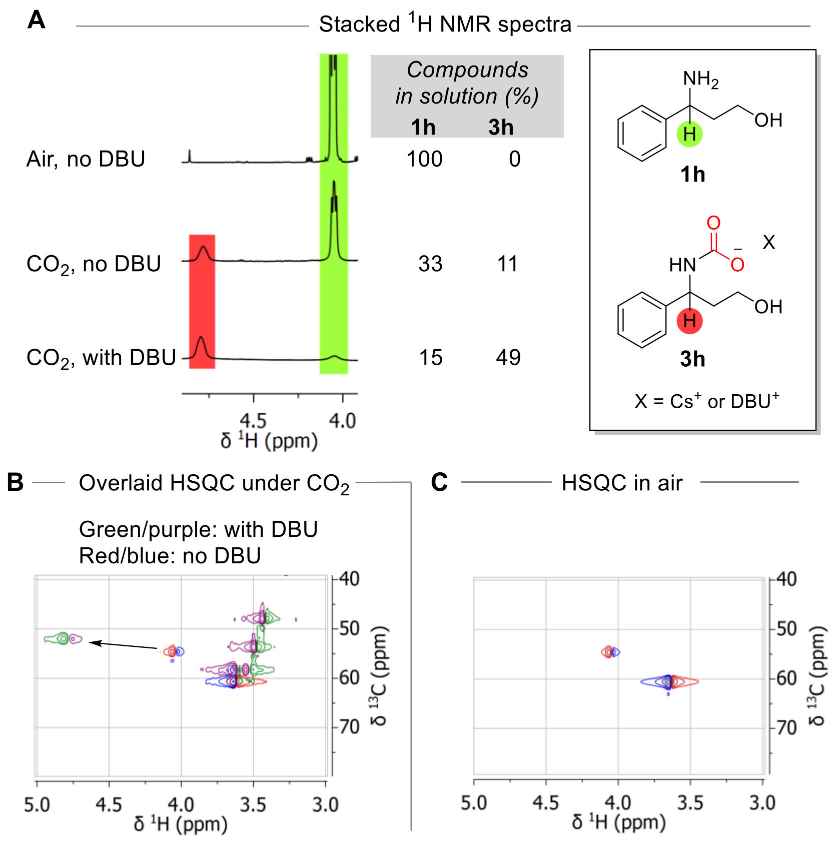

Figure S1. *In situ* NMR studies in CD_3CN with DBU (20 mol%) using mesitylene (internal standard). (A) The amount of amino alcohol-derived compounds in solution. (B) Overlaid HSQC spectra show that the major species is different when DBU is present. The arrow indicates migration of the benzylic hydrogen when DBU is added. (C) The major species under air is the same one as under CO_2 without DBU.

2.2 Experimental Details

In air, DL-phenylalaninol (363 mg, 2.4 mmol) was dissolved in 6.0 ml CD_3CN. To this solution was added mesitylene (333.6 µl, 2.4 mmol) as an internal standard. Two 25 ml Schlenk tubes were loaded each with a stir bar and Cs_2CO_3 (3 equiv, 1.2 mmol, 391 mg). The Schlenk tubes were evacuated and backfilled with CO_2 for four cycles. Under CO_2 flow, 2.5 ml of the CD_3CN solution, containing 0.4 mmol DL-phenylalaninol and 0.4 mmol mesitylene, was added to each Schlenk tube. To the second Schlenk tube was added DBU (20 mol%, 12 µl, 0.08 mmol). Approximately 0.7 ml of the remaining CD_3CN solution was added by syringe to a J. Young tube, which was sealed. The solutions in the Schlenk tubes were stirred for an hour. The DBU-containing solution had free flowing solids (**Figure S2**). In contrast, in the absence of DBU,

significant amounts of solids deposited on the sides of the Schlenk tube. At this point, stirring was stopped and the solids were allowed to settle. The glass stoppers were exchanged to rubber septa. A large Schlenk tube was loaded with two J. Young tubes, and tube was evacuated and backfilled with CO_2 for four cycles. Under CO_2 flow, ca 0.7 ml of solution was withdrawn by syringe from each 25 ml Schlenk tube and transferred to the J. Young tubes, which were sealed under CO_2. The solutions were then analyzed by NMR.

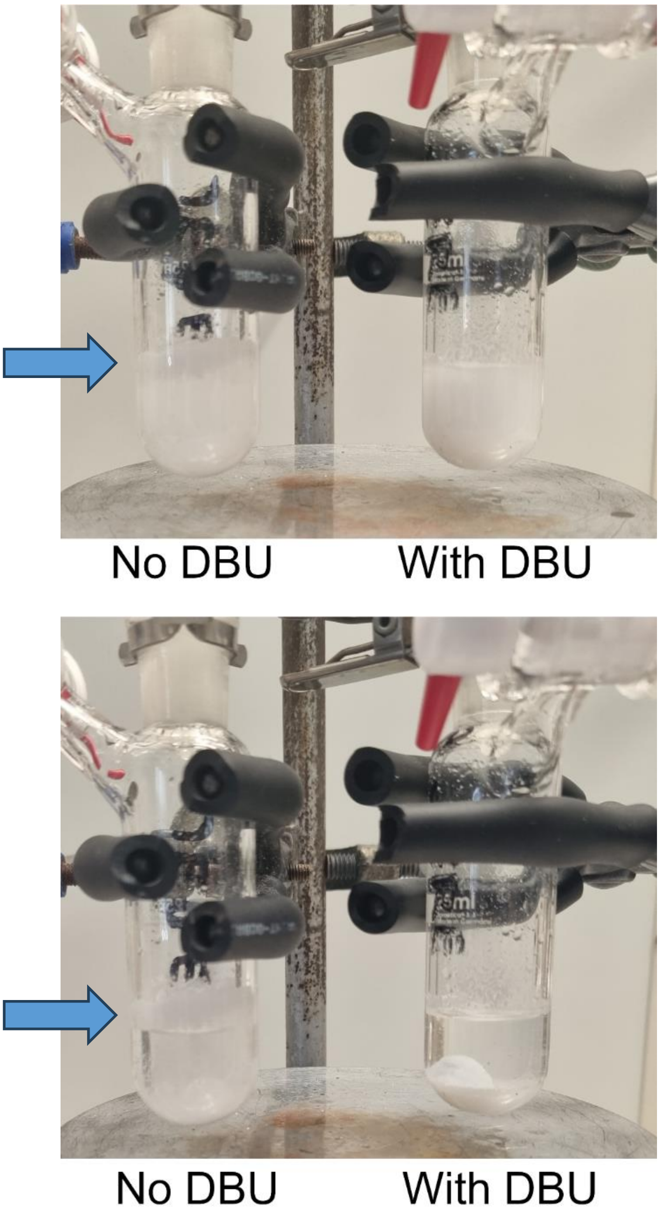

Figure S2. Reaction of DL-phenylalaninol with CO_2 and Cs_2CO_3 in CD_3CN in the presence or absence of DBU (20 mol%). The arrow indicates formation of solid on the side of the Schlenk tube in the absence of DBU. Top: stirred solution, bottom: solution left to settle.

Spectral Characterization – Under Air

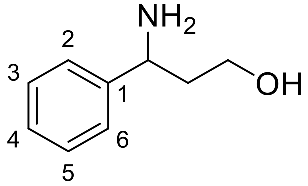

^{1}H NMR (500 MHz, CD_3CN, air, 25°C): δ 7.35 (4H, m, Ar**H**), 7.24 (1H, m, Ar**H**), 6.81 (3H, mesitylene Ar**H**), 4.03 (1H, dd, *J* = 8.0, 5.4 Hz, $C\mathbf{H}NH_2$), 3.62 (2H, t, *J* = 6.0 Hz, $C\mathbf{H_2}OH$), 2.51 (3H, br, O**H** and $N\mathbf{H_2}$), 2.26 (9H, mesitylene $C\mathbf{H_3}$), 1.78 (2H, m, $C\mathbf{H_2}CH_2OH$)

^{13}C{^{1}H} NMR (128.5 MHz, CD_3CN, air, 25°C): δ 148.5 (C1), 138.7 (mestitylene $\mathbf{C}CH_3$), 129.4 (C3 & C5), 127.8 (mestitylene **C**H), 127.6 (C4), 127.1 (C2 and C6), 61.5 ($\mathbf{C}H_2OH$), 55.8 ($\mathbf{C}HNH_2$), 42.0 ($\mathbf{C}H_2CH_2OH$), 21.3 (mestitylene $C\mathbf{C}H_3$)

Spectral Characterization – Under CO_2 (without DBU)

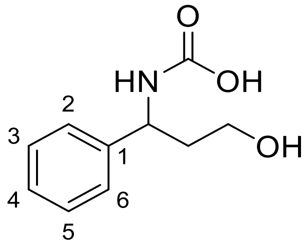

^{1}H NMR (500 MHz, CD_3CN, CO_2, 25°C): δ 7.34 (4H, m, Ar**H**), 7.22 (1H, m, Ar**H**), 6.80 (3H, mesitylene Ar**H**), 4.76 (1H, br, C**H**NHCOOH), 4.04 (1H, dd, *J* = 8.5, 5.4 Hz, unreacted $C\mathbf{H}NH_2$), 3.60 (2H, t, *J* = 5.8 Hz, $C\mathbf{H_2}OH$ overlap with unreacted $C\mathbf{H_2}OH$), 2.94 (3H, br, O**H** and $N\mathbf{H_2}$, overlap with unreacted species), 2.24 (9H, mesitylene $C\mathbf{H_3}$), 1.77 (2H, m, $C\mathbf{H_2}CH_2OH$ overlap with unreacted $C\mathbf{H_2}CH_2OH$)

^{13}C{^{1}H} NMR (128.5 MHz, CD_3CN, CO_2, 25°C): δ 138.7 (mestitylene $\mathbf{C}CH_3$), 129.4 (C3 & C5), 127.8 (mestitylene **C**H), 127.6 (C4), 127.0 (C2 and C6), 125.8 (free CO_2), 61.7 ($\mathbf{C}H_2OH$), 56.0 ($\mathbf{C}HNH_2$), 41.9 ($\mathbf{C}H_2CH_2OH$), 21.3 (mestitylene $C\mathbf{C}H_3$), C1 not observable.

Spectral Characterization – Under CO_2 (with DBU)

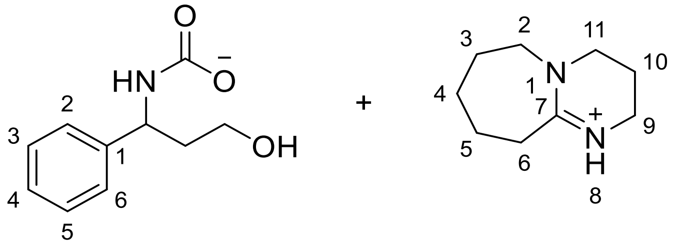

^{1}H NMR (500 MHz, CD_3CN, CO_2, 25°C): δ 7.33 (4H, m, Ar**H**), 7.22 (1H, m, Ar**H**), 6.80 (3H, mesitylene Ar**H**), 6.42 (1H, br, CHN**H**COOH), 4.77 (1H, br, C**H**NHCOOH), 4.02 (1H, dd, *J* = 8.5, 5.4 Hz, unreacted C**H**NH_2), 3.60 (2H, t, *J* = 5.8 Hz, C**H₂**OH overlap with unreacted C**H₂**OH), 3.52 (1H, br, CH_2O**H**), 3.46 (2H, m, DBU H2), 3.40 (2H, t, J = 5.9 Hz, H11), 3.23 (2H, t, J = 5.8 Hz, H9), 2.64 (2H, m, DBU H6), 2.24 (9H, mesitylene C**H₃**), 1.94 (2H, p, J = 5.9 Hz, DBU H10), 1.85 (2H, br, C**H₂**CH_2OH), 1.70 (4H, m, DBU H5 & H4), 1.63 (2H, m, DBU H3)

^{13}C{^{1}H} NMR (128.5 MHz, CD_3CN, CO_2, 25°C): δ 167.0 (DBU C7), 146.3 (C1), 138.7 (mestitylene **C**CH_3), 129.3 (C3 & C5), 127.7 (mestitylene **C**H), 127.5 (C4), 125.8 (free CO_2), 59.5 (br, **C**H_2OH), 54.3 (DBU C2), 53.0 (br, **C**HNH_2), 49.3 (DBU C11), 41.5 (br, **C**$H_2$$CH_2$OH), 39.0 (DBU C9), 33.1 (DBU C6), 29.6 (DBU C4), 27.2 (DBU C3), 24.6 (DBU C5), 21.3 (mestitylene C**C**H_3), 20.1 (DBU C10), C2 and C6 not detectable.

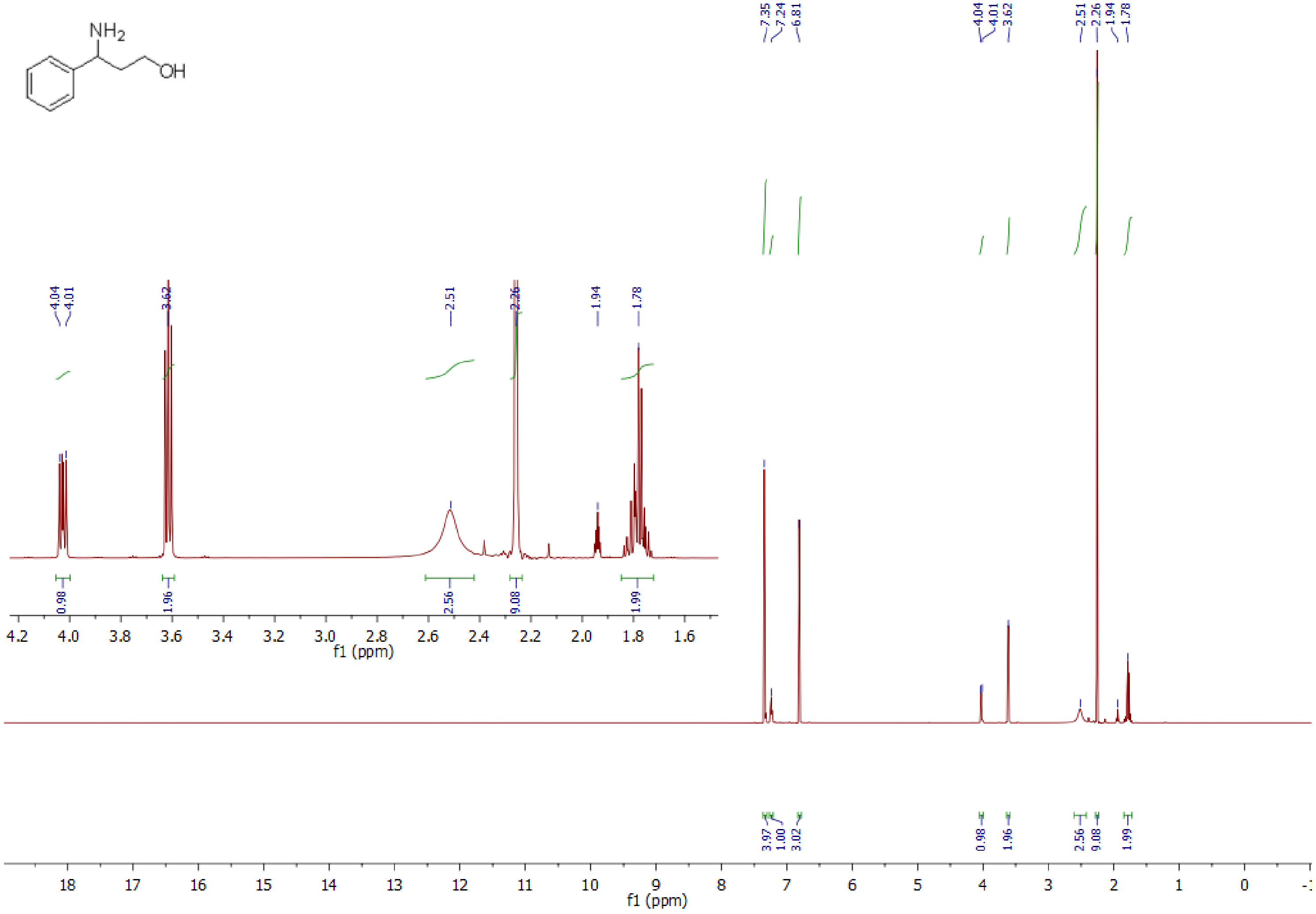

[1]H Spectrum of **1h** under air in CD_3CN at 25°C. [1]H-frequency 500 MHz spectrometer.

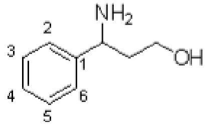

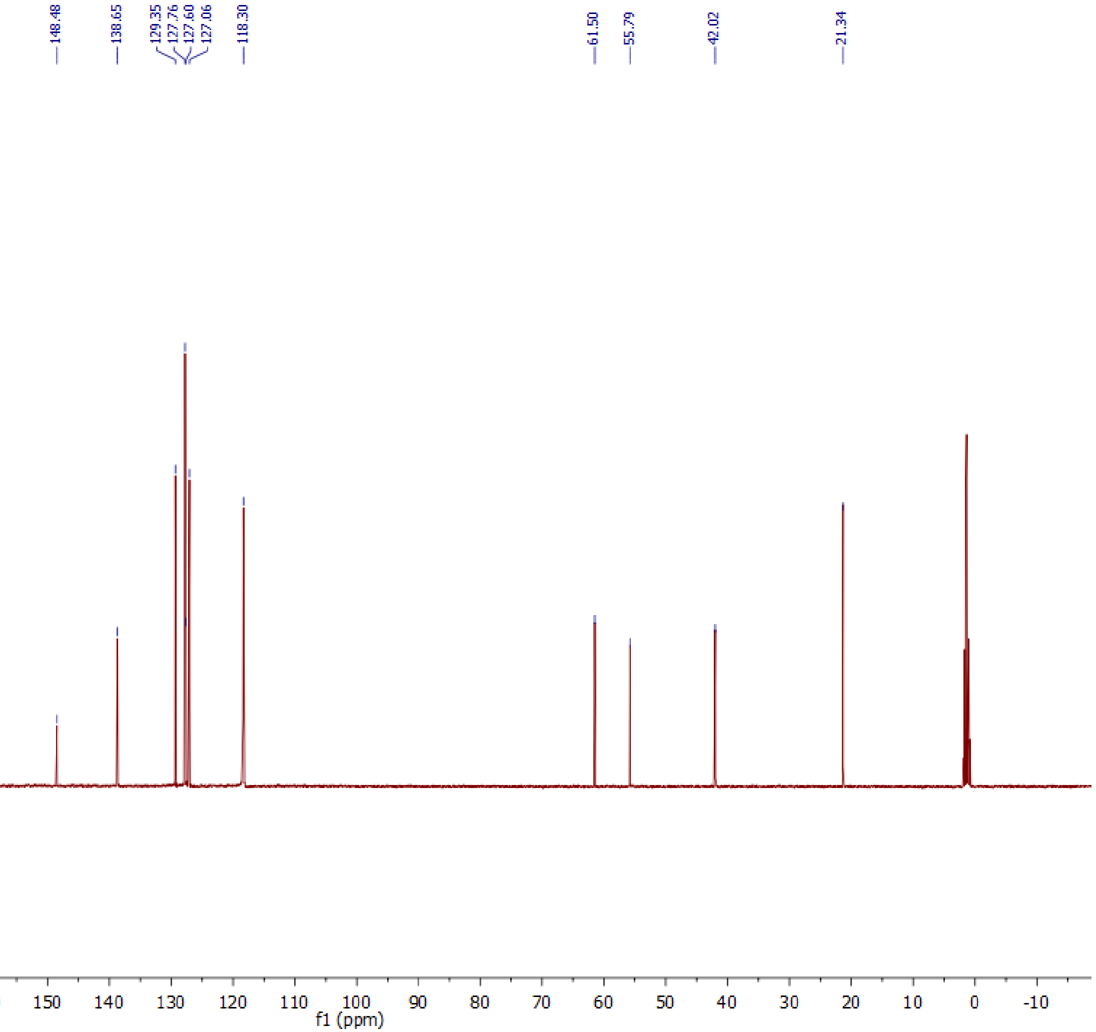

$^{13}C\{^{1}H\}$ Spectrum of **1h** under air in CD_3CN at 25°C. ^{1}H-frequency 500 MHz spectrometer.

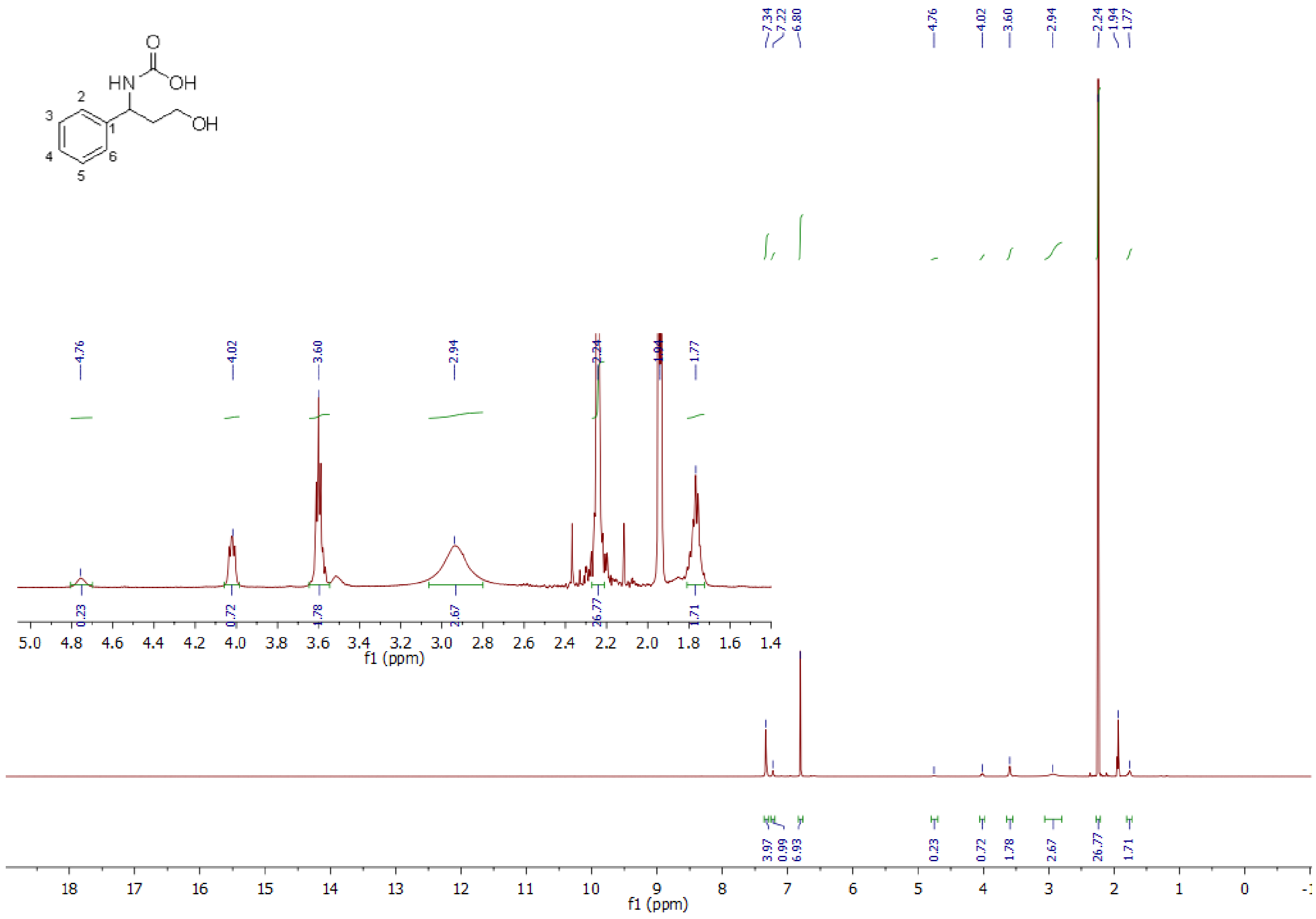

[1]H Spectrum of **1h**, forming **3h**, with Cs_2CO_3 under CO_2 (without DBU) in CD_3CN at 25°C. [1]H-frequency 500 MHz spectrometer.

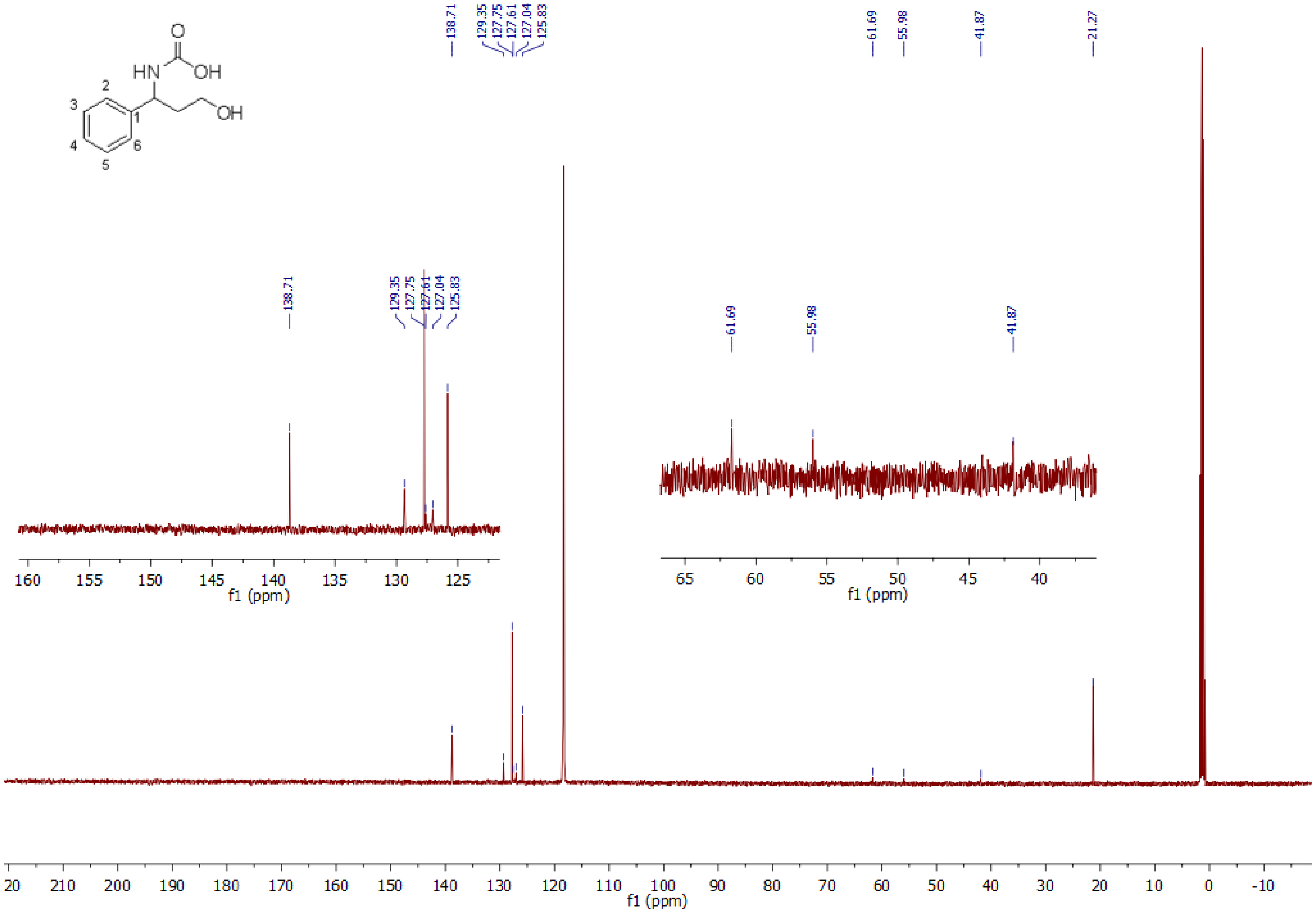

$^{13}C\{^1H\}$ Spectrum of **1h**, forming **3h**, with Cs_2CO_3 under CO_2 (without DBU) in CD_3CN at 25°C. ^1H-frequency 500 MHz spectrometer.

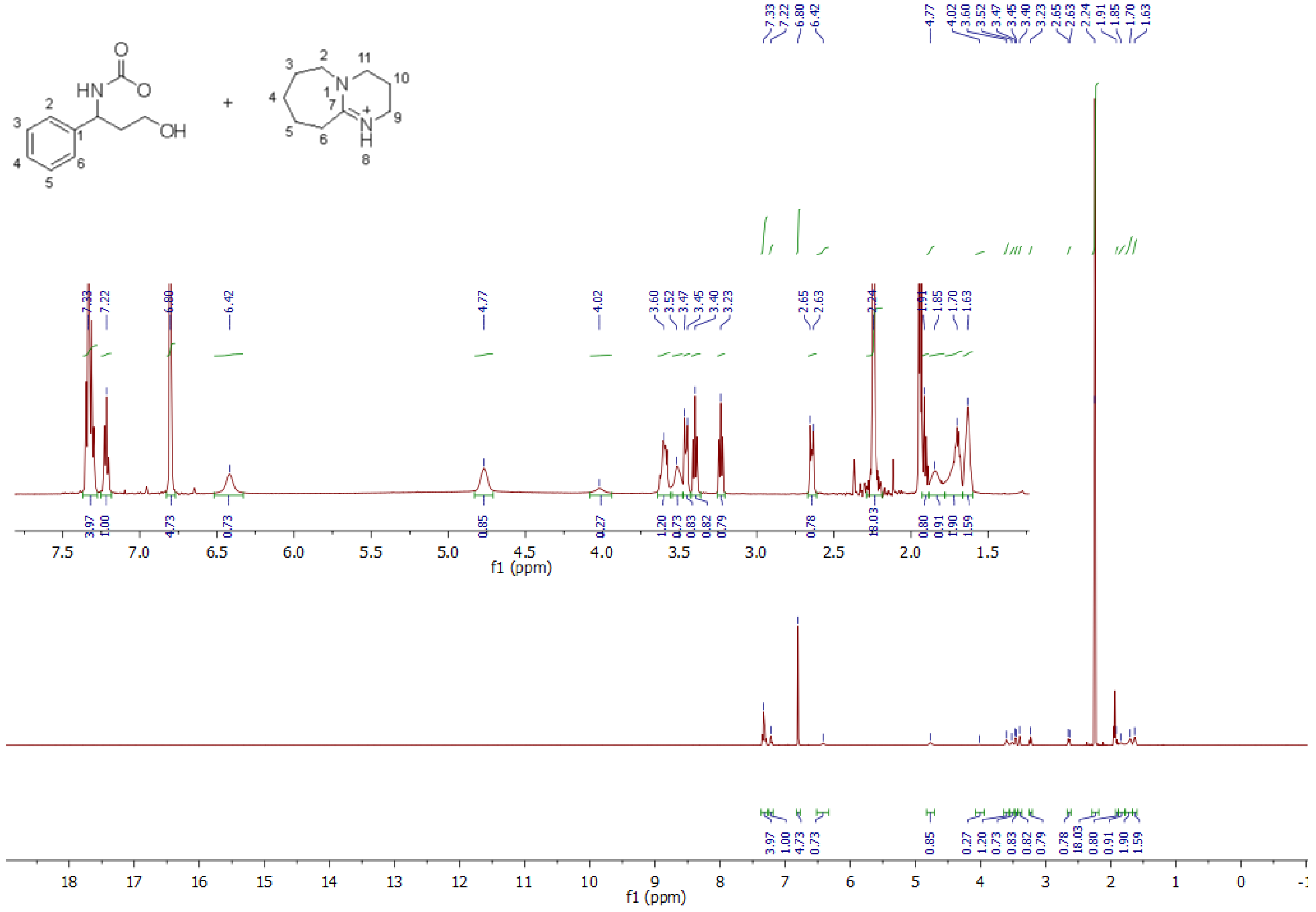

[1]H Spectrum of **1h**, forming **3h**, with Cs_2CO_3 under CO_2 (with DBU) in CD_3CN at 25°C. [1]H-frequency 500 MHz spectrometer.

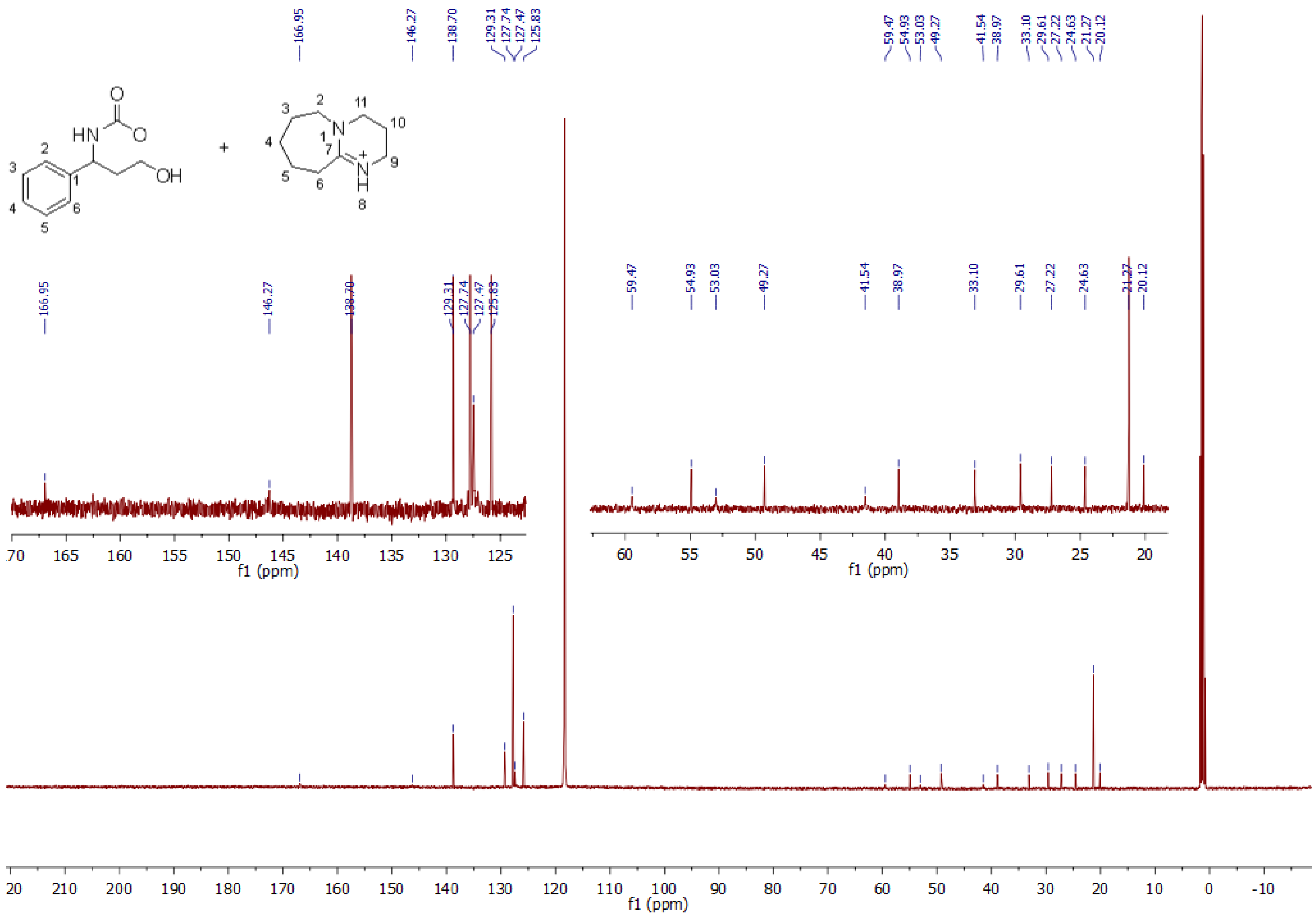

$^{13}C\{^1H\}$ Spectrum of **1h**, forming **3h**, with Cs_2CO_3 under CO_2 (with DBU) in CD_3CN at 25°C. ^1H-frequency 500 MHz spectrometer.

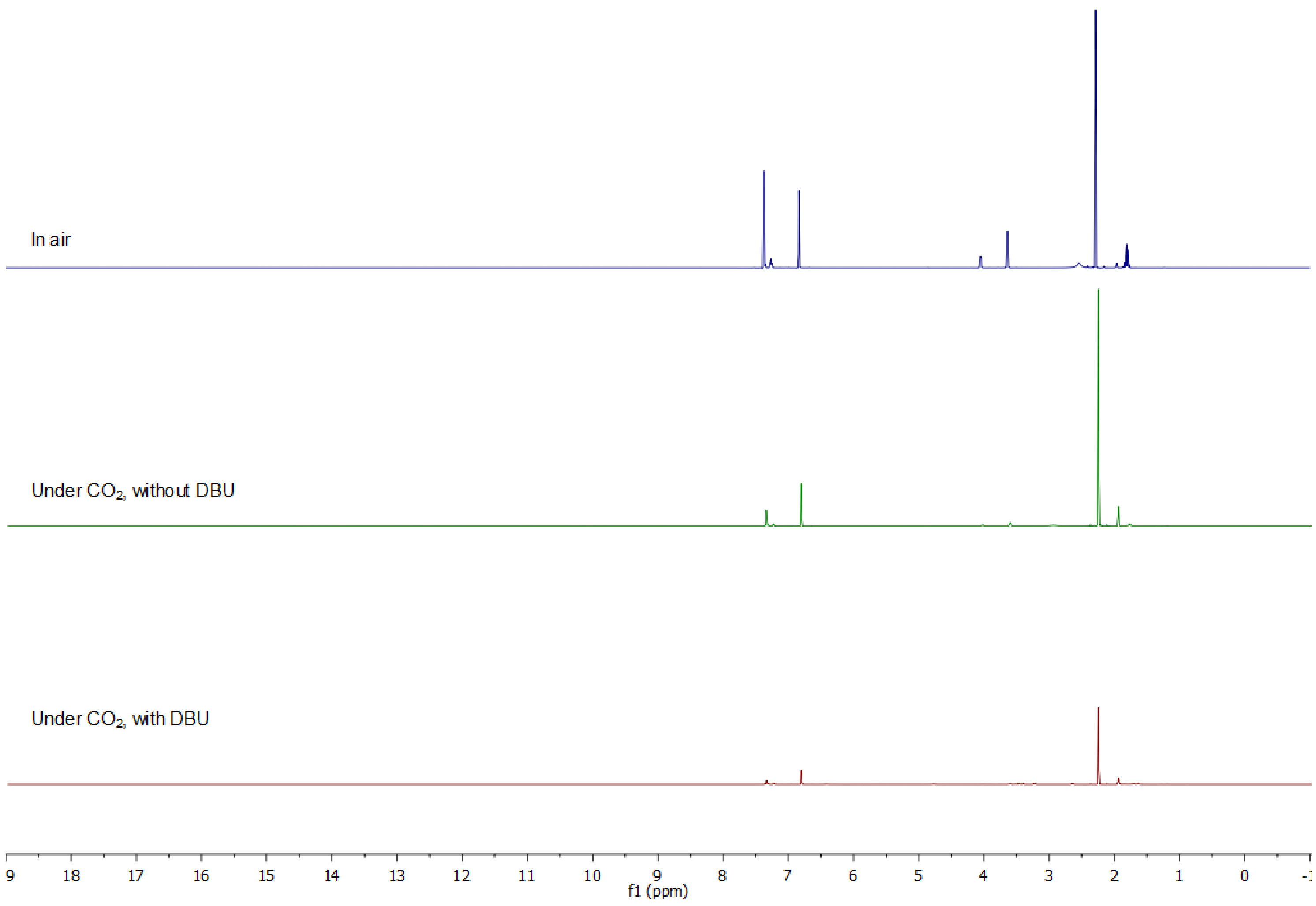

Stacked ^{1}H spectra of **1h** under various conditions in CD_3CN at 25°C. Experiments under CO_2 included Cs_2CO_3. [1]H-frequency 500 MHz spectrometer.

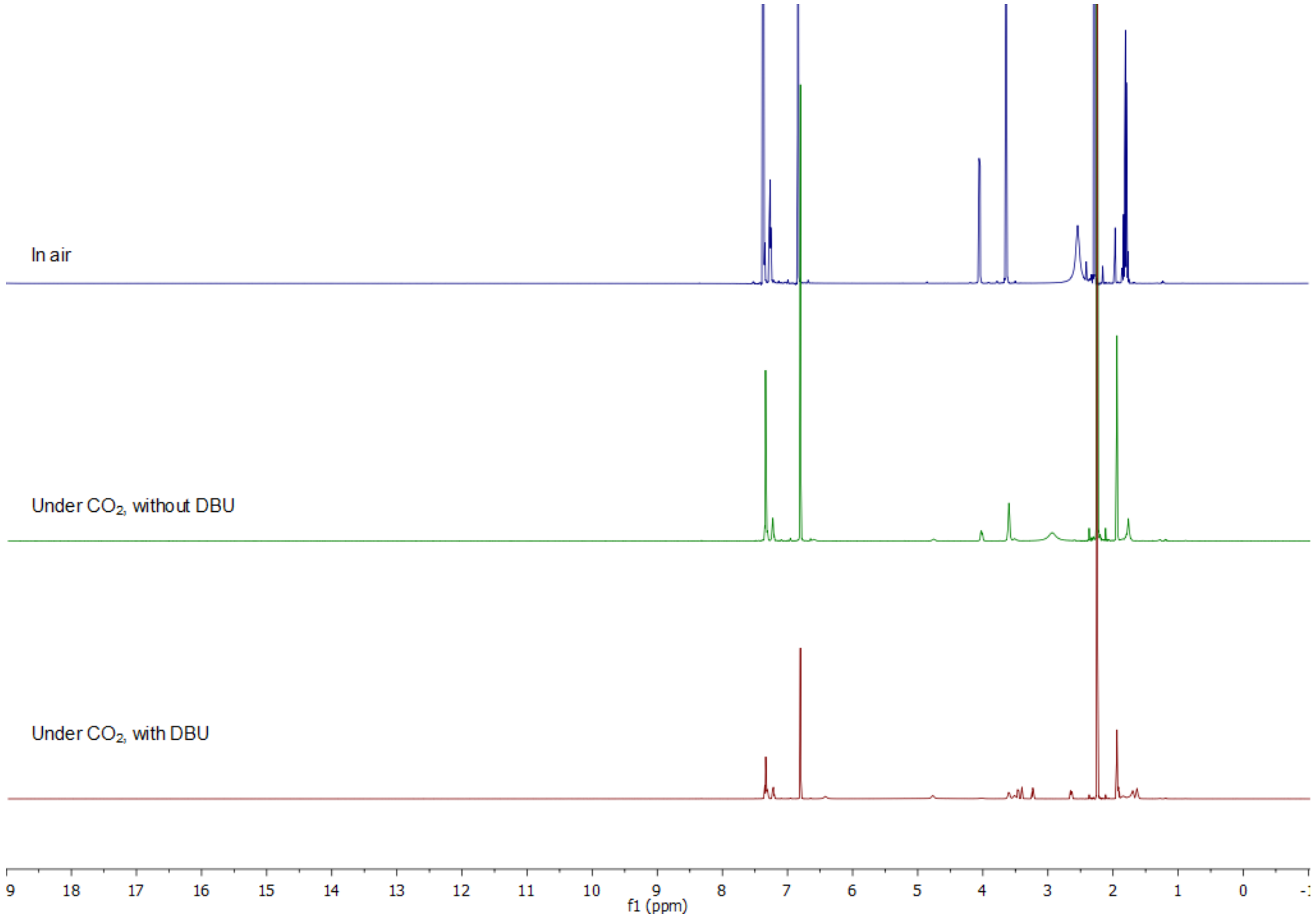

Stacked ^{1}H spectra of **1h** under various conditions in CD_3CN at 25°C. More details shown compared to previous page. [1]H-frequency 500 MHz spectrometer.

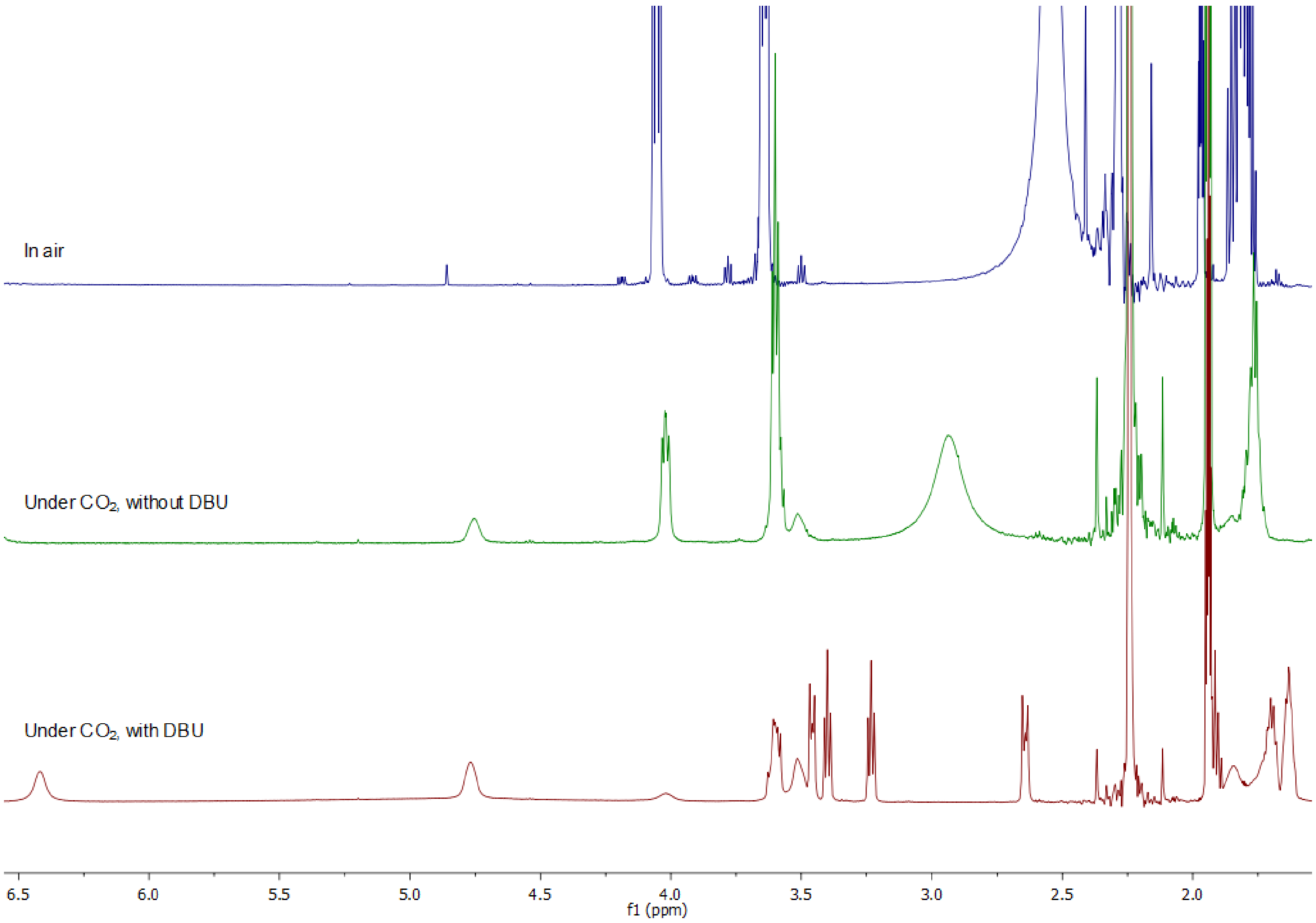

Stacked ^{1}H spectra of **1h** under various conditions in CD_3CN at 25°C. Selected region. [1]H-frequency 500 MHz spectrometer.

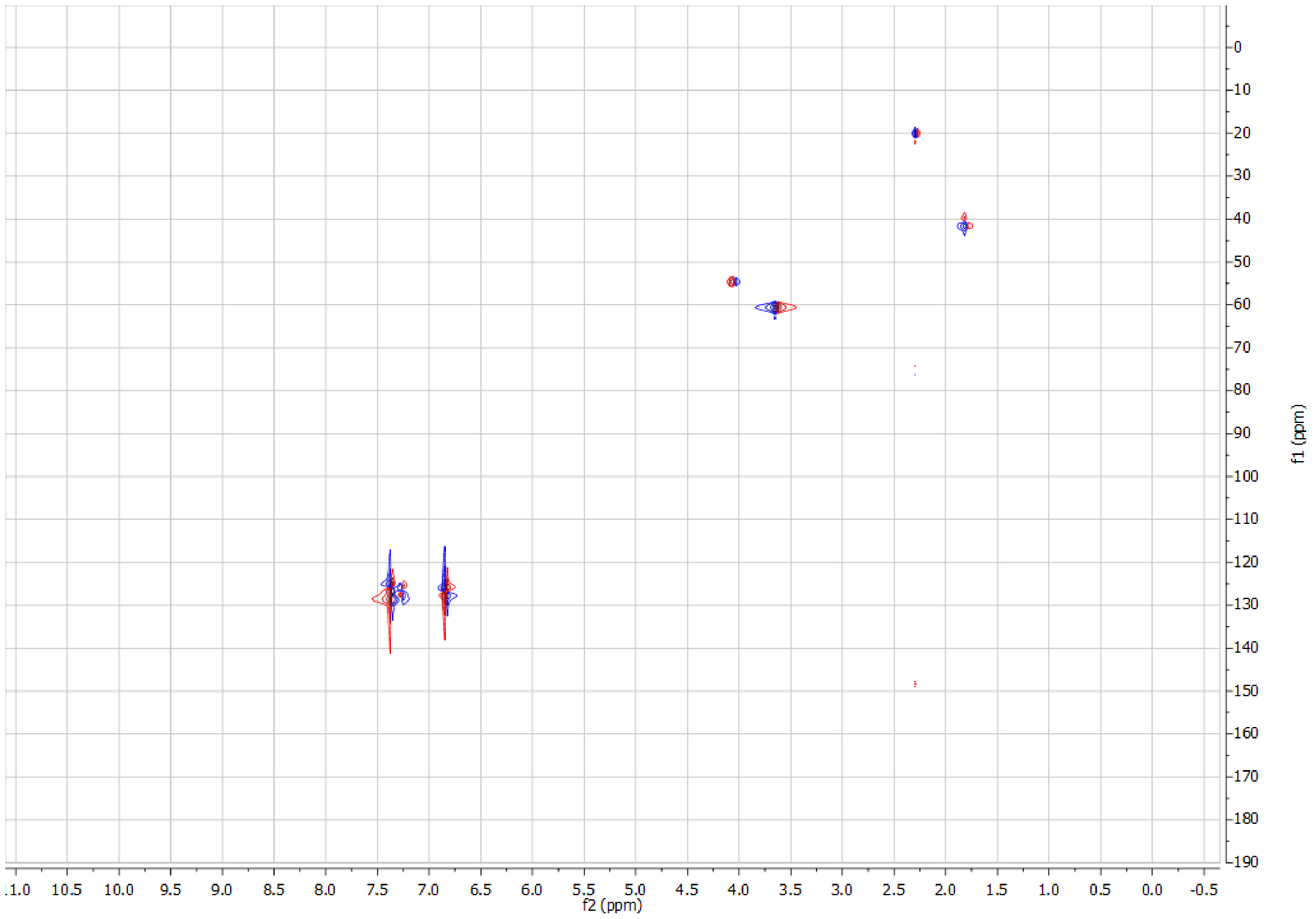

The HSQC spectrum of **1h** under air in CD_3CN at 25°C. [1]H-frequency 500 MHz spectrometer.

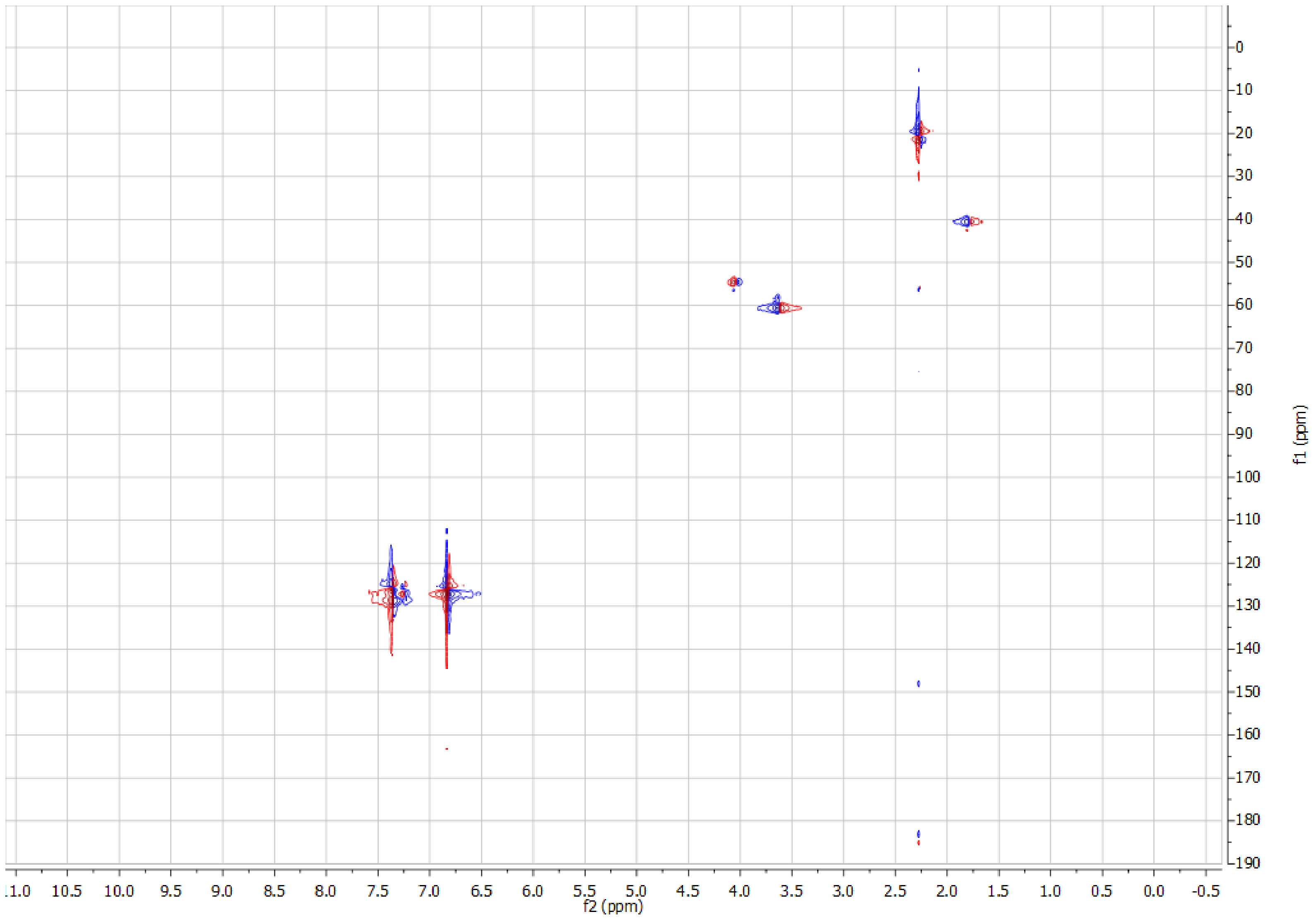

HSQC Spectrum of **1h**, forming **3h**, with Cs_2CO_3 under CO_2 (without DBU) in CD_3CN at 25°C. ^{1}H-frequency 500 MHz spectrometer.

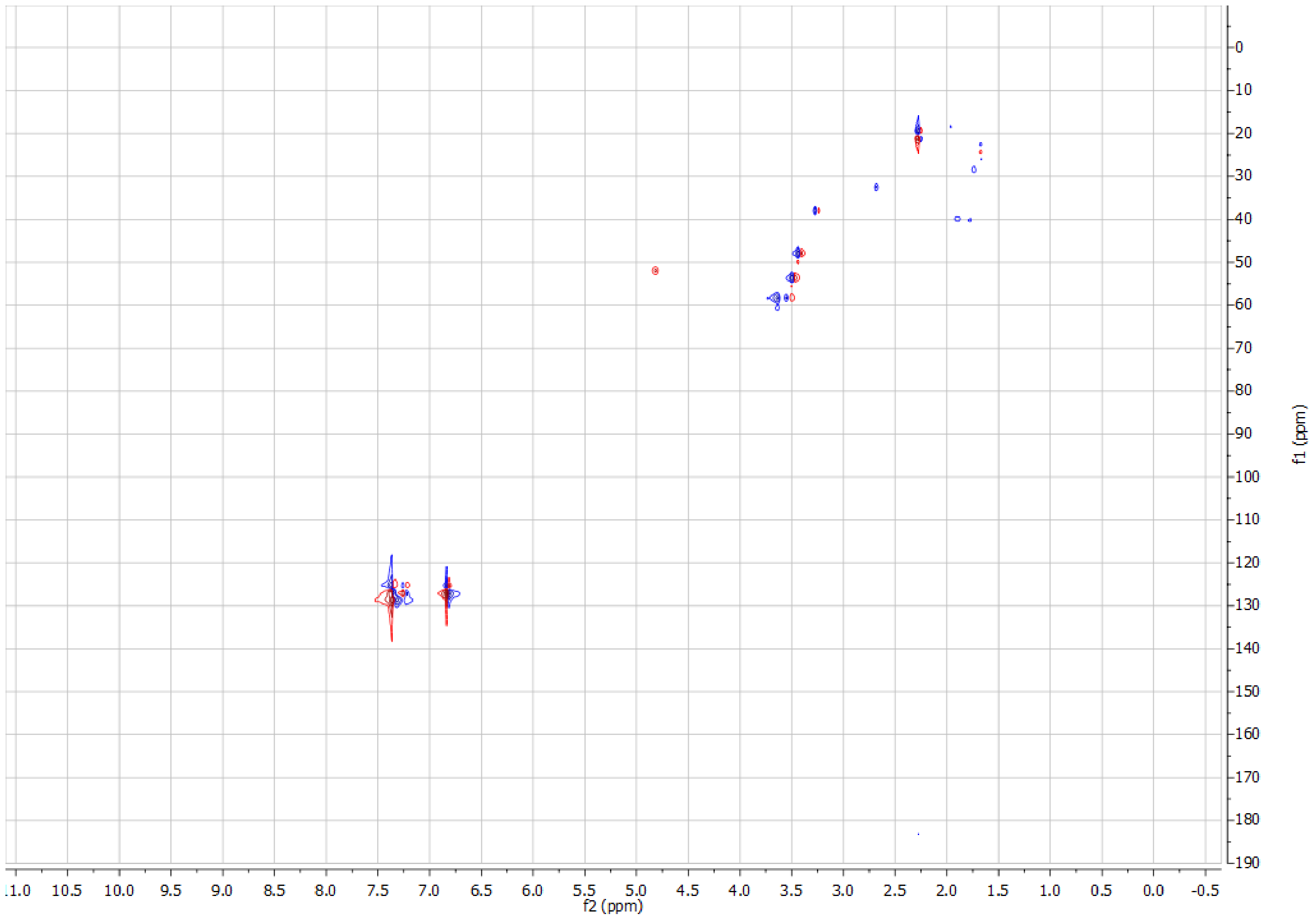

HSQC Spectrum of **1h**, forming **3h**, with Cs_2CO_3 under CO_2 (with DBU) in CD_3CN at 25°C. ^{1}H-frequency 500 MHz spectrometer.

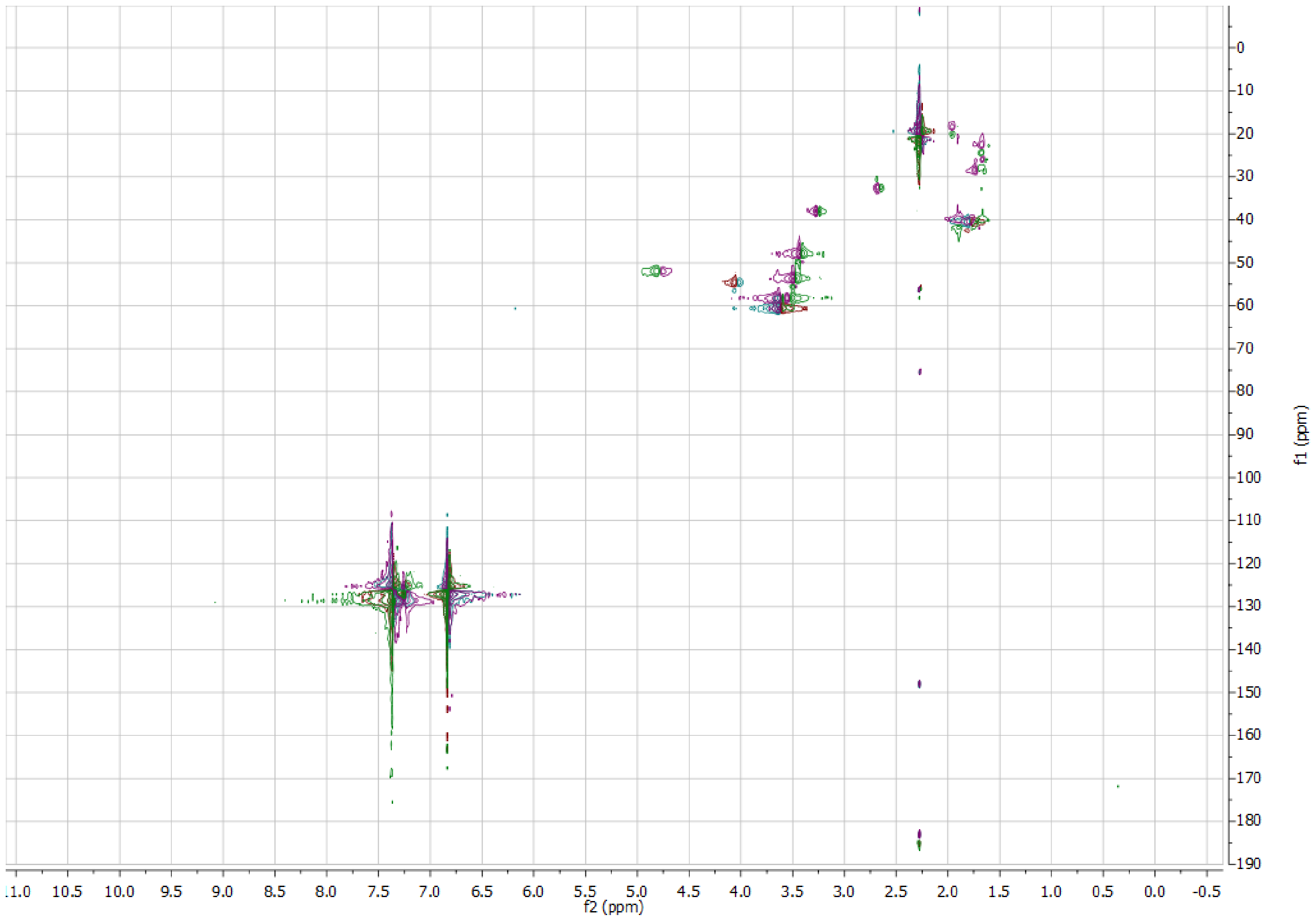

Overlaid HSQC spectra of **1h**, forming **3h**, with Cs_2CO_3 under CO_2 in CD_3CN at 25°C. With DBU in green/purple and without DBU in blue/red. [1]H-frequency 500 MHz spectrometer.

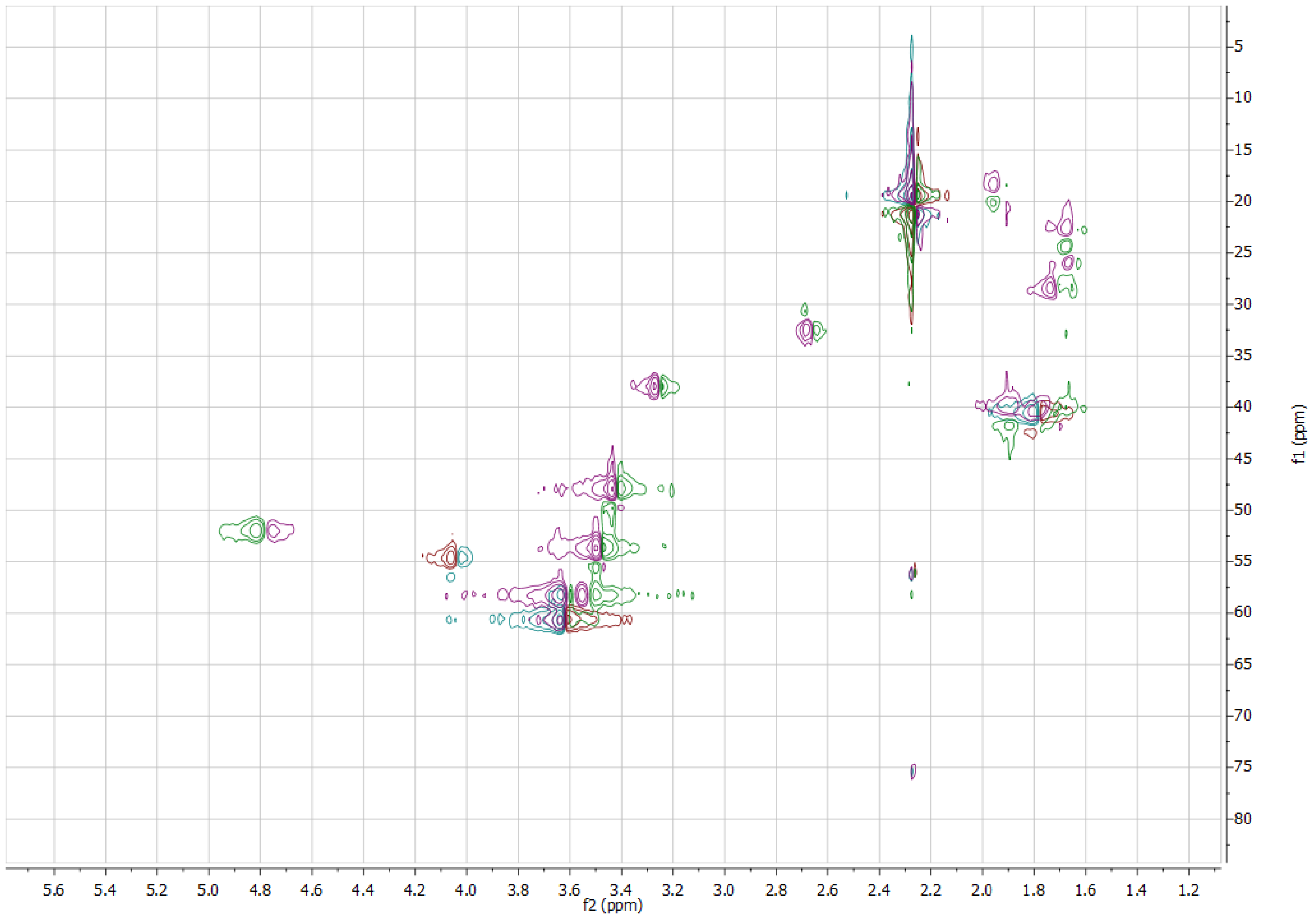

Overlaid HSQC spectra (selected region) of **1h**, forming **3h**, with Cs_2CO_3 under CO_2 in CD_3CN at 25°C. With DBU in green/purple and without DBU in blue/red. ^{1}H-frequency 500 MHz spectrometer.

3. FTIR Spectra

3.1 Discussion

Having studied solution behavior, we turned to analyzing the reaction solid phase by FTIR. Three suspensions in CH_3CN under CO_2 were prepared: standard conditions (20 mol% DBU, **1h**, Cs_2CO_3), no DBU, and control (only Cs_2CO_3). The solids obtained in the absence DBU showed peaks at 1580, 1444, and 1421 cm^{-1} consistent with an anionic carbamate species.[1] The characteristic peaks of ammonium species (RNH_3^+) at ca 2500 and 2000 cm^{-1} were absent, suggesting Cs^+ to be the counter cation. The solids obtained in the presence of DBU appeared very similar, however, a small peak at 3670 cm^{-1} was observed, consistent with DBH^+. While the DBU-derived carbamate salt may partially precipitate, most of it seems to remain in solution based on the NMR results (section **2.1** and **Figure S1**). Therefore, the forming precipitate mostly consists of the cesium carbamate salt.

3.2 Experimental Details

To three 250 ml filter funnels with bottom and side stopcocks was added a stir bar and Cs_2CO_3 (3 equiv, 12 mmol, 3.91 g) each. The filter funnels were evacuated and backfilled with CO_2 for four cycles. Under CO_2 flow, 60 ml dry ACN was added to filter funnel 1. Similarly, to filter funnel 2 and 3 was added a solution of DL-phenylalaninol (605 mg, 4.0 mmol) in 60 ml ACN. To filter funnel 3 was added DBU (20 mol%, 120 µl, 0.8 mmol). The filter funnels were placed in a ca 30° angle over stir plates and stirred for one hour (**Figure S2**). After this time, the filter funnels were turned upright and the solution was filtered under a positive pressure of CO_2 until constant weight (ca 15 min). The filter funnels were then sealed and transported to the FTIR spectrometer. The filter funnels were opened one at a time, a sample was quickly taken and a FTIR spectrum recorded.

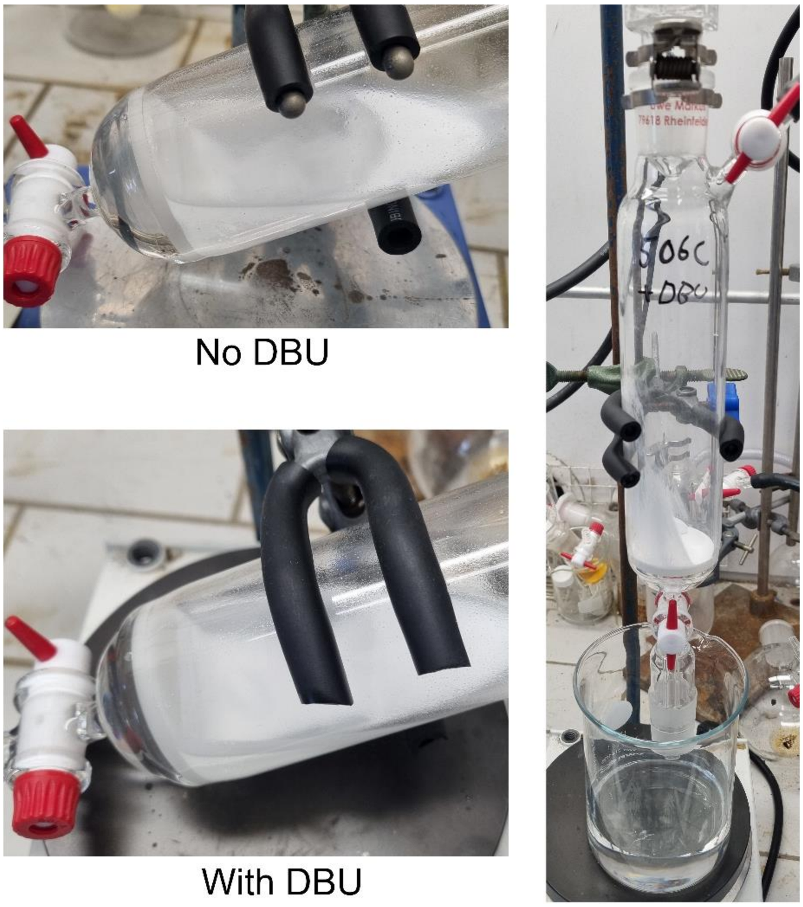

Figure S3. Sample preparation for FTIR studies. Top left: reaction without DBU, bottom left: reaction with DBU, right: filtration setup.

Cs_2CO_3 (Control)

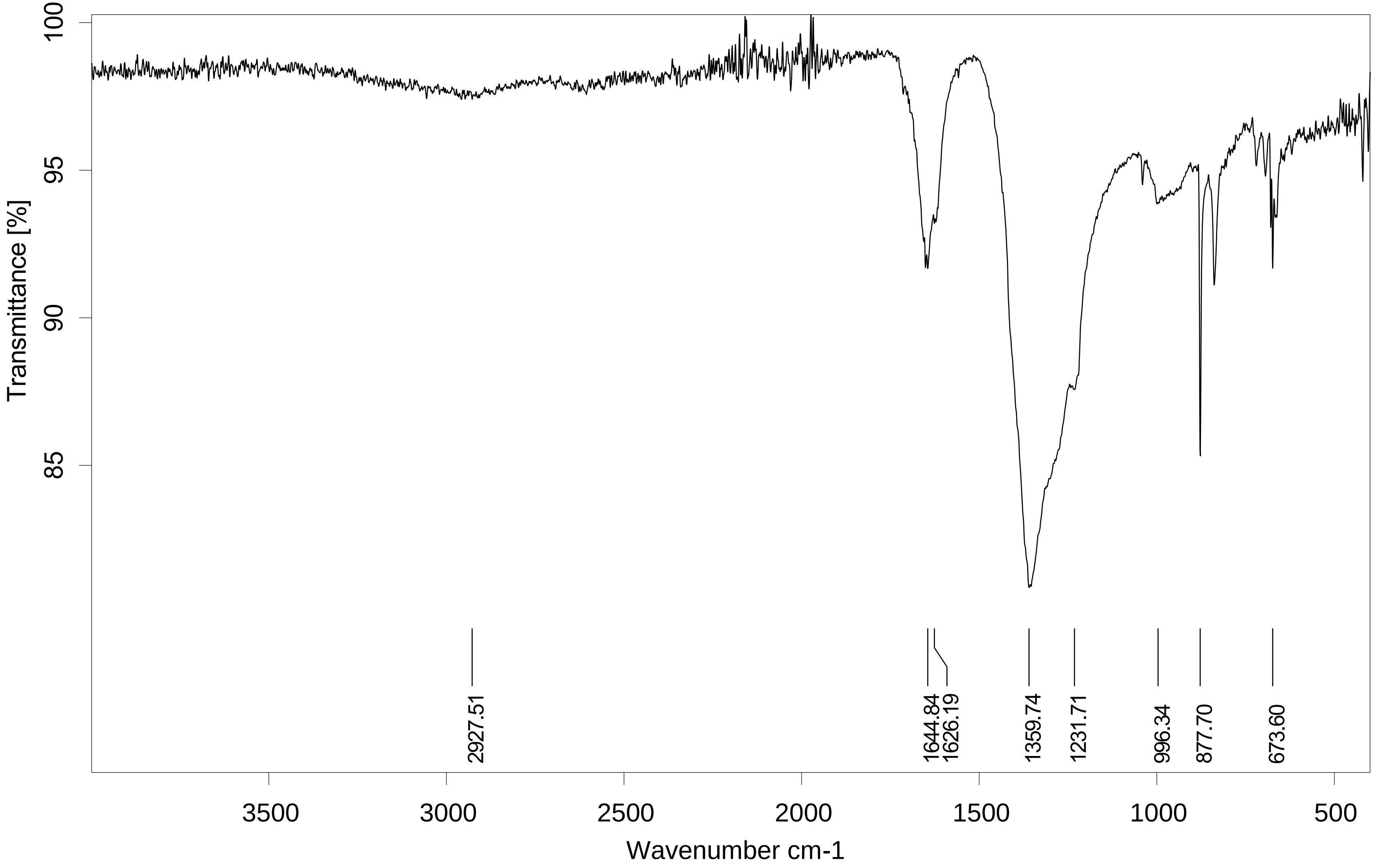

Without DBU

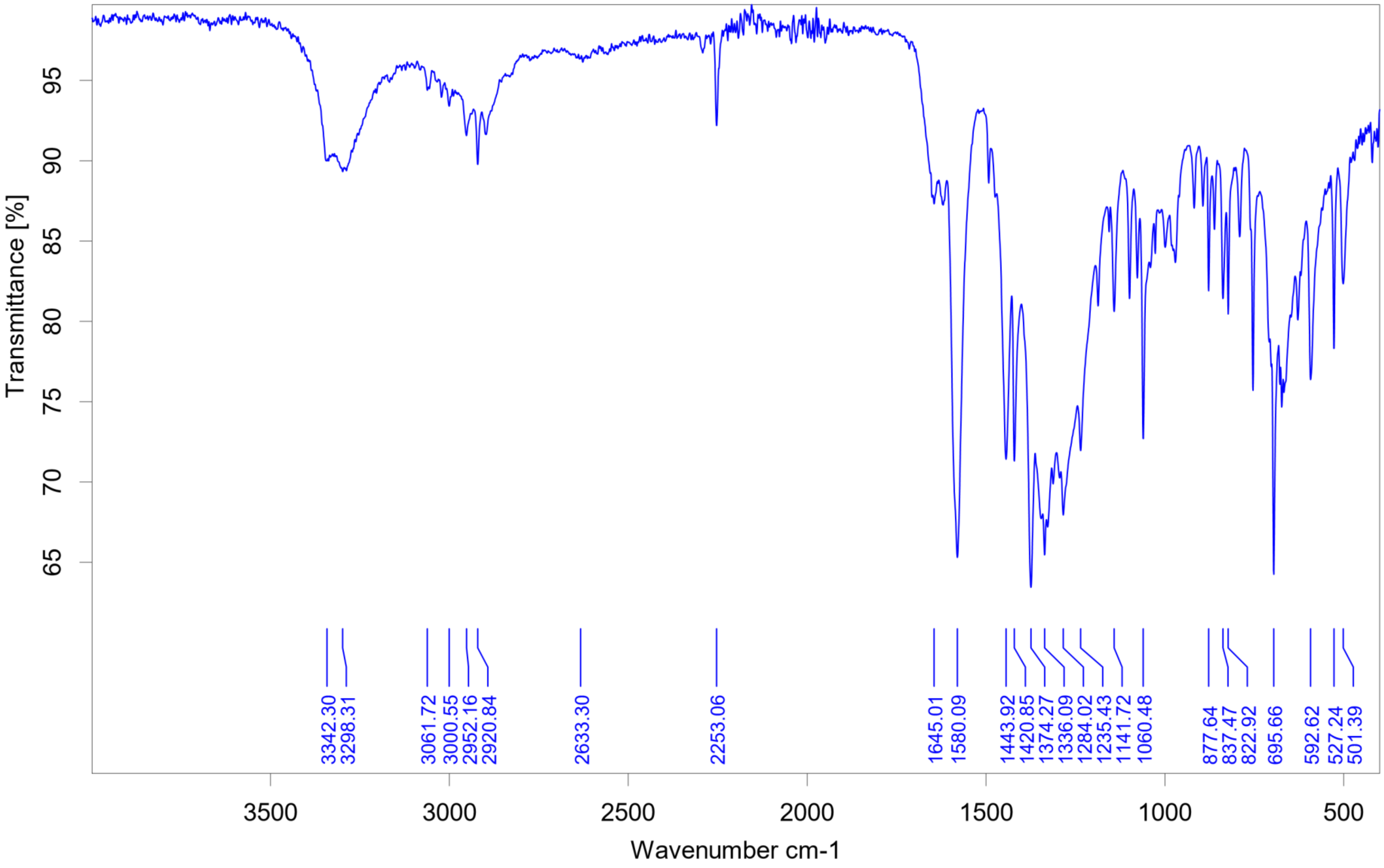
95
90
85
80
75
70
65
Transmittance [%]
3342.30
3298.31
3061.72
3000.55
2952.16
2920.84
2633.30
2253.06
1645.01
1580.09
1443.92
1420.85
1374.27
1336.09
1284.02
1235.43
1141.72
1060.48
877.64
837.47
822.92
695.66
592.62
527.24
501.39
3500
3000
2500
2000
1500
1000
500
Wavenumber cm-1

With DBU

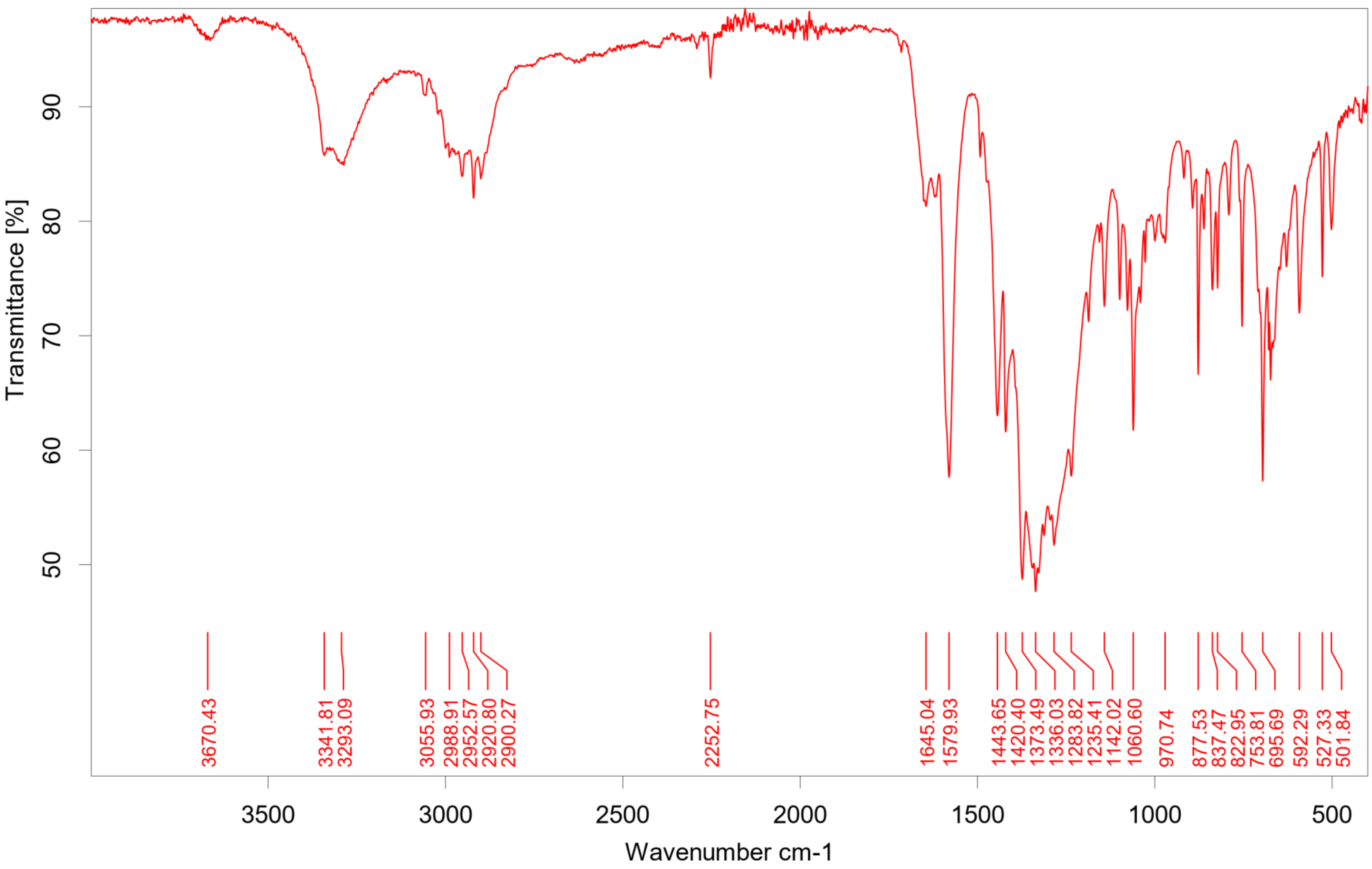

Transmittance [%]
90
80
70
60
50
3670.43
3341.81
3293.09
3055.93
2988.91
2952.57
2920.80
2900.27
2252.75
1645.04
1579.93
1443.65
1420.40
1373.49
1336.03
1283.82
1235.41
1142.02
1060.60
970.74
877.53
837.47
822.95
753.81
695.69
592.29
527.33
501.84
3500
3000
2500
2000
1500
1000
500
Wavenumber cm-1

The Effect of DBU (Overlaid Spectra of With and Without DBU)

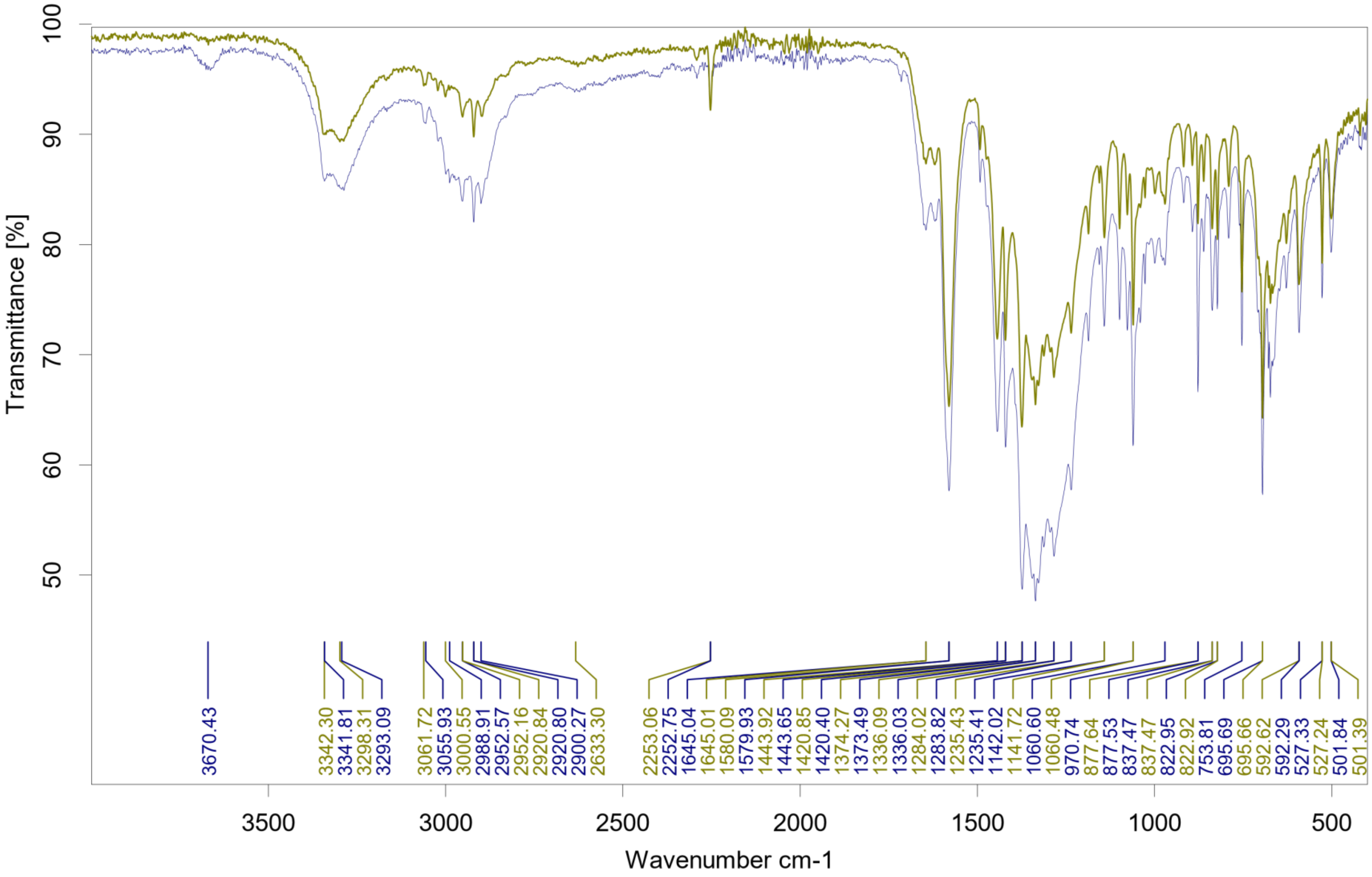

Green: no DBU, blue: with DBU.

All Experiments Overlaid

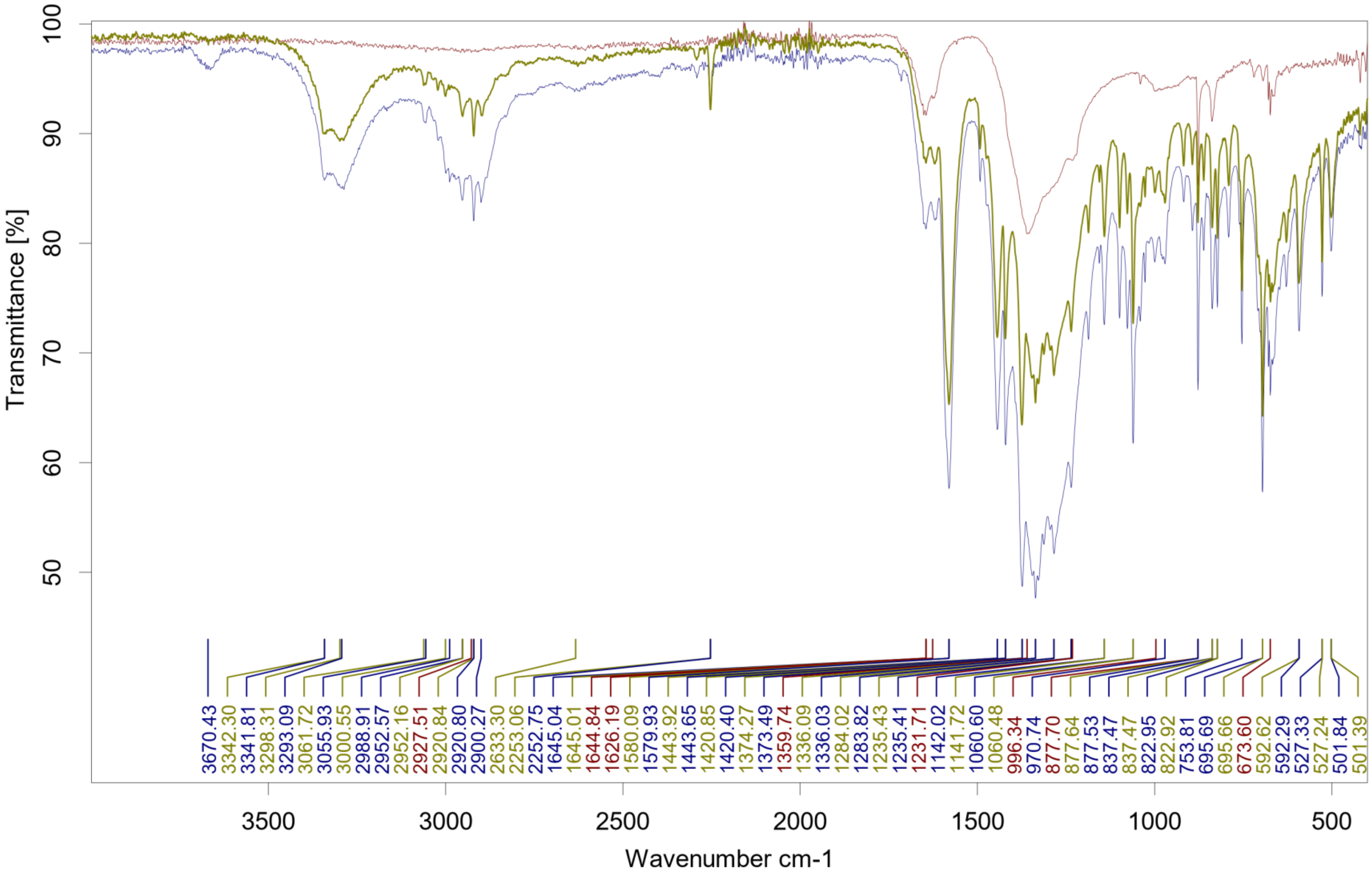

Red: only Cs_2CO_3 (control), green: no DBU, blue: with DBU.

4. Experimental Procedures and Characterization Data of Amino Alcohols

Compound 1c

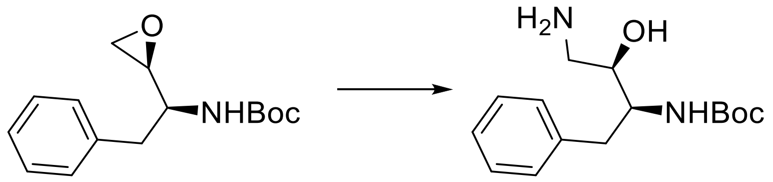

1e

To a 500 ml round-bottom flask was added tert-butyl (1S)-1-(oxiran-2-yl)-2-phenyllethyl)carbamate (50 mmol, 13.17g), which was dissolved in dry methanol (150 ml). Then, 7 M ammonia in methanol (150 ml) was added. The reaction mixture was stirred for two days at room temperature. The solvent was evaporated, leaving a white powder, which was purified by column chromatography using 10:100:1 EtOH:DCM:triethylamine (R_f = 0.18). Ninhydride stain was used for visualization. Fractions containing the product were combined and evaporated, yielding a white powder (8.9 g, 55%).

^{1}H NMR (400 MHz, DMSO-d_6, 25 °C): δ 7.19 (5H, m), 6.67-6.65 (1H, d), 4.75 (1H, broad), 3.54 (1H, m), 3.25 (1H, m), 3.05 (1H, m), 2.60 (1H, m), 2.52 (1H, m), 2.47 (1 H), 1.76 (1H, broad), 1.27-1.15 (9H, s).

^{13}C{^{1}H} NMR (102.8 MHz, DMSO-d_6, 25 °C): δ 28.66 (CH_3), 36.63 (CH_2), 45.14 (NH_2-CH_2), 54.73 (COONH-**C**H), 74.58 (OH-CH), 77.82 (NHCOO-**C**), 126.04 (ArCH), 128.33 (ArCH), 129.55 (ArCH), 140.33 (ArC), 155.83 (HNCOO).

HRMS (ESI-TOF) Calculated for $C_{15}H_{25}N_2O_3^+$ $[M+H]^+$ 281.1860; found 281.1846

Compound 1e

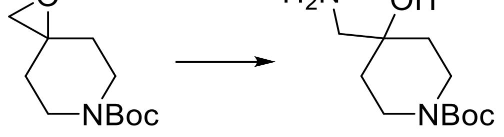

1d

To a 30 ml microwave vial was added tert-butyl 1-oxa-6-azaspiro[2.5]octane-6-carboxylate (6 mmol, 1.28g). A magnetic stirrer was added to the vial and the epoxide was dissolved in dry

methanol (9 ml). Commercial 7 M ammonia in methanol (9 ml) was added to the reaction mixture. The vial was sealed and heated in a microwave reactor at 120 °C for 2h. The solvent was evaporated and the remaining yellow wax was triturated with hexane three times (ca 3 x 30 ml) and dried in vacuo. This yielded a yellow solid (1.31 g, 95%).

^{1}H NMR ($CDCl_3$, 400 MHz, 25 °C): δ 3.87 (2H, m), 3.18 (2H, m), 2.61 (2H, s), 1.47 (13H, m).

^{13}C{^{1}H} NMR (102.8 MHz, $CDCl_3$, 25 °C): δ 28.46 (CH_3), 34.72 (CH_2), 39.93 (COON-**C**H_2), 60.55 (NH_2-CH_2), 68.45 (OH-C), 79.31 (NCOO-**C**), 154.88 (NCOO).

HRMS (ESI-TOF) Calculated for $C_{11}H_{23}N_2O_3^+$ $[M+H]^+$ 231.1704; found 231.1695

Compound 1j

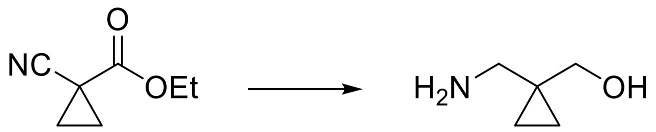

1j

An oven-dried 250 ml three-neck round-bottom flask with a stir bar and a pressure-equalized addition funnel was connected to a Schlenk line and placed under vacuum. Once cool, the flask was vented and lithium aluminum hydride (5.0 g) was added. A thermometer was connected to the flask, which was evacuated and filled with argon. Dry THF (100 ml) was added under argon flow and the flask cooled in an ice water bath to 0 °C. Commercial ethyl 1-cyano-1-cyclopropanecarboxylate (110 mmol, 14.2 ml) was dissolved in 30 ml of dry THF, and added dropwise to the reaction solution, keeping temperature below 10 °C. After addition was complete, the ice water bath was removed and the flask was left to stir for 3h at room temperature. Next, the flask was placed in an ice water bath and 13 g of $NaSO_4 \cdot 10\ H_2O$ was added under argon flow in small portions, keeping temperature below 10 °C. The reaction mixture was stirred vigorously overnight. A Celite plug was prepared and washed with 1 M NaOH, distilled water, and ethyl acetate, in this order. The reaction mixture was filtered through Celite. The filtrate was evaporated and the oily residue purified by distillation (97 °C / 20 mmHg). The product was a clear liquid (3.50 g, 31%).

^{1}H NMR (400 MHz, $CDCl_3$, 25 °C): δ 3.56 (2H, s), 2.78 (2H, s), 2.73 (2H, broad), 0.46 (1H, m), 0.39 (1H, m).

^{13}C{^{1}H} NMR (102.8 MHz, $CDCl_3$, 25 °C): δ 8.93 ($CyPrCH_2$), 23.65 (CyPrC), 49.22 (NH_2-CH_2), 69.83 (OH-CH_2).

HRMS (ESI-TOF) Calculated for $C_5H_{12}NO^+$ $[M+H]^+$ 102.0914; found 102.0909

Compound S01

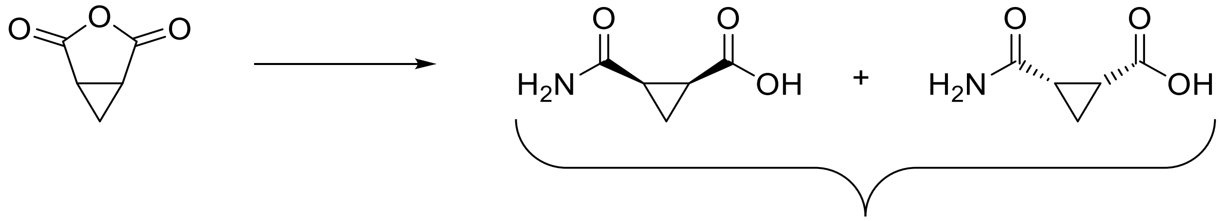

S01

1,2-Cyclopropanedicarboxylic anhydride (70 mmol, 7.8 g) was added to a 500 ml round-bottom flask and dissolved in dry methanol (50 ml). The reaction mixture was cooled in an ice water bath and 7 M ammonia in methanol (250 ml) was added dropwise via a pressure-equalized addition funnel. The flask was closed with a stopper and the reaction mixture was stirred at room temperature for 8 days. The solvent was evaporated. The solid residue was dissolved in 10 ml distilled water and then concentrated HCl added until the solution was strongly acidic, causing the product to precipitate out. The reaction mixture was placed in a refrigerator overnight. The solids were collected by filtration and washed with distilled water. The crude product was triturated in refluxing isopropanol (ca 200 ml) and dried in vacuo. This yielded a white powder (4.5 g, 44%). The product is a 1:1 racemic mixture.

^{1}H NMR (400 MHz, DMSO-d_6, 25 °C): δ 12.35 (1H, s), 7.71 (1H, s), 7.11 (1H, s), 1.90 (2H, m), 1.29 (1H, m), 1.12 (1H, m).

^{13}C{^{1}H} NMR (102.8 MHz, DMSO-d_6, 25 °C): δ 11.62 ($CyPrCH_2$), 22.09 (HOOC-**C**H), 22.67 (NH_2CO-**C**H_2), 171.87 (COOH), 172.15 ($CONH_2$).

HRMS (ESI-TOF) Calculated for $C_5H_7NNaO_3^+$ $[M+Na]^+$ 152.0319; found 152.0325

Compound 1o

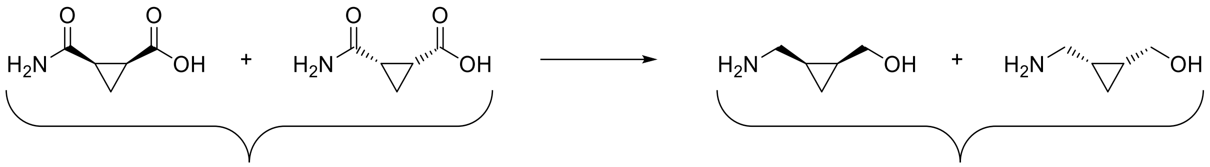

An oven-dried 250 ml three-neck round-bottom flask with a stir bar was connected to a Schlenk line and placed under vacuum. Once cool, the flask was vented and lithium aluminum hydride (2.5 g) was added. A thermometer was connected to the flask, which was evacuated and filled with argon. Dry THF (100 ml) was added under argon flow and the flask cooled in an ice water bath to 0 °C. Solid compound **S01** (31.0 mmol, 4.0 g) was added in small portions using a funnel via the side neck, keeping temperature below 10 °C. After addition was complete, the ice water bath was removed and the flask was left to stir for 30 min at room temperature. A reflux condenser was attached under argon and the mixture was refluxed for 4 h. Afterwards, the flask was left to cool to room temperature and then placed in an ice water bath. Under argon flow, 7 g of $NaSO_4 \cdot 10\ H_2O$ was added in small portions, keeping temperature below 10 °C. The reaction mixture was stirred vigorously overnight. A Celite plug was prepared and washed with 1 M NaOH, distilled water, and ethyl acetate, in this order. The reaction mixture was filtered through Celite. The filtrate was evaporated and the oily residue purified by distillation (100 °C/20 mmHg). The product was a clear oil (1.0 g, 32%) as a 1:1 racemic mixture.

^{1}H NMR (400 MHz, $CDCl_3$, 25 °C): δ 4.00 (1H, m), 3.44 (1H, m), 3.19 (1H, m), 2.85 (2H, broad), 2.22 (1H, m), 1.33 (1H, m), 1.06 (1H, m), 0.77 (1H, m), 0.18 (1H, m).

^{13}C{^{1}H} NMR (102.8 MHz, $CDCl_3$, 25 °C): δ 9.81 ($CyPrCH_2$), 17.01 (CyPrCH), 17.91 (CyPrCH), 41.55 (NH_2-CH_2), 63.30 (OH-CH_2).

HRMS (ESI-TOF) Calculated for $C_5H_{12}NO^+$ $[M+H]^+$ 102.0914; found 102.0917

Compound 1l

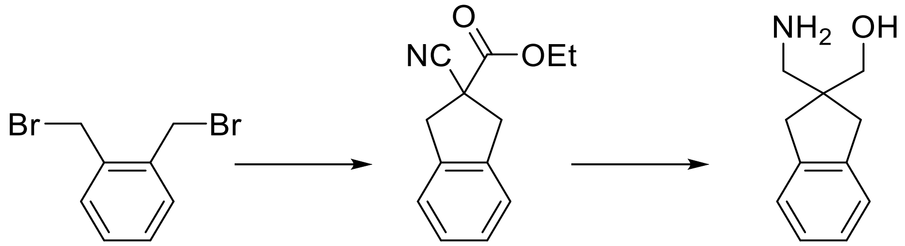

S02 **1l**

To a 1000 ml round-bottom flask was added 1,2-bis(bromomethyl)benzene (24.5 g, 92 mmol), which was dissolved in 600 ml ACN. While stirring, ethyl 1-cyano-1-cyclopropanecarboxylate (9.9 ml, 92 mmol) was added, followed by K_2CO_3 (51 g, 4 equiv). A reflux condenser was attached and the solution was refluxed for 1 h, and then allowed to cool to room temperature. The solvent was evaporated, and the residue was partitioned between 200 ml water and 200 ml 3:1 EtOAc:toluene mixture, and transferred to a separatory funnel. The phases were separated and the aqueous phase extracted 3 x 100 ml using 3:1 EtOAc:toluene. The organic layers were combined and dried with $MgSO_4$. Filtration and evaporation of the solvent gave compound **S02** (20 g, 100%) as a white solid, which was used directly in the next step.

Lithium aluminum hydride (2.1 g) was added to an oven-dried 500 ml three-neck round-bottom flask. The flask was connected to a reflux condenser, pressure-equalized addition funnel, and thermometer. The apparatus was evacuated and filled with argon. Dry THF (100 ml) was added. The flask was cooled to 0°C in an ice water bath. Compound **S02** (44.8 mmol, 9.64 g) was dissolved in 100 ml dry THF and added slowly via the addition funnel, keeping the reaction temperature below 10 °C. After the addition was complete, the reaction mixture was stirred at room temperature for 3 hours. The flask was placed in an ice water bath and 7 g of $NaSO_4·10\ H_2O$ was added in small portions, keeping the reaction temperature of the solution below 10 °C. The reaction mixture was stirred vigorously overnight. A Celite plug was prepared and washed with 1 M NaOH, distilled water, and ethyl acetate, in this order. The reaction mixture was filtered through Celite. The filtrate was evaporated, leaving a yellow oil, which solidified on standing. The crude was purified by distillation (140 °C/0.15 mmHg). The yellowish distillate solidified on standing, which was recrystallized from EtOAc/hexane to give **1l** as a white solid (3.15 g, 40%).

^{1}**H NMR** (400 MHz, $CDCl_3$, 25 °C): δ 7.16 (4H, m), 3.74 (2H, s), 3.05 (2H, s), 2.96-2.92 (2H, d), 2.77-2.73 (2H, d).

^{13}**C{^{1}H} NMR** (102.8 MHz, $CDCl_3$, 25 °C): δ 39.53 (CH_2), 48.01 (C), 51.26 (NH_2-CH_2), 71.13 (OH-CH_2), 124.99 (ArCH), 126.41 (ArCH), 141.84 (ArCH).

HRMS (ESI-TOF) Calculated for $C_{11}H_{16}NO^+$ $[M+H]^+$ 178.1227; found 178.1218

5. Experimental Procedures

5.1 General Optimization Details

All optimization reactions for cyclic carbamate **2a** were carried out on 0.5 mmol scale in oven-dried 25 ml Schlenk tubes using 7 ml solvent (ACN). After the reaction, mesitylene (70 µl) was added as an internal standard and the solution mixed. Approximately 0.1 ml of the solution was withdrawn and diluted with ca 1.3 ml EtOAc, and then analyzed by GC-MS. The relative area (A_{rel}) of product was determined according to equation S1, where the area of product (A(product)) is divided by the area of mesitylene (A(mesitylene)).

$$A_{rel}\ (\%) = \frac{A(\text{product})}{A(\text{mesitylene})} \times 100 \qquad \text{(S1)}$$

5.1 Attempted Re-optimization for Cyclic Carbamate 2f

During the preparation of this work, we qualitatively noticed that cyclization seemed to proceed slower for six- and seven-membered cyclic carbamates. Consequently, T3P was added over 12 hours for these substrates.

Standard conditions readily provided five-membered cyclic carbamates (**Scheme 2** in the manuscript), however, six-membered product **2f** was only obtained in trace amounts (Table S1, entry 1). Conducting the reaction in alternative solvents THF, DMSO, DMF, NMP, and DCM did not improve the yield. Re-optimization was initially attempted by increasing temperature (entries 2 and 3). The yields did slightly improve, but not to a significant degree. Solubility was thought to be the limiting factor, and therefore, fully miscible organic bases were probed (entries 4-6). Again, only marginal improvements were observed.

Switching from Cs_2CO_3 to anhydrous K_3PO_4 alone, or in combination with DBU or potassium-ion chelating 18-crown-6 gave only small improvements in yield (entries 7-10).

Dual addition, *i.e.*, simultaneous slow addition of T3P and **1f** to the reaction mixture at 25°C or 60°C did not significantly improve the outcome (entries 11 and 12).

Table S1. Optimization details for six membered cyclic carbamate 2f

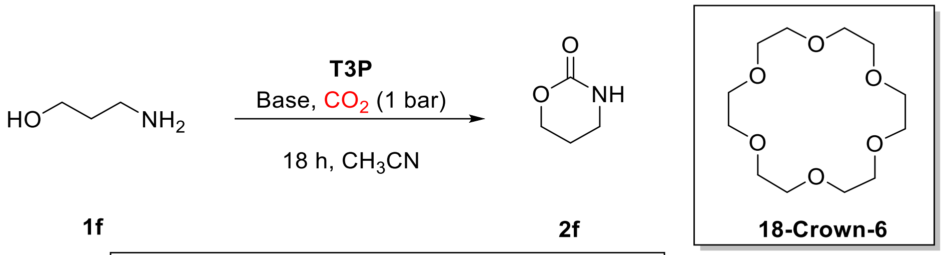

Standard additon: **T3P** added over 12h

Dual addition: **1f** and **T3P** added over 12h

Entry	Condition	Reagent addition	Temperature (°C)	A_{rel} of **2f** (%)
1	Cs_2CO_3 (3.0) + DBU (0.2)	Standard	25	9
2	Cs_2CO_3 (3.0) + DBU (0.2)	Standard	40	11
3	Cs_2CO_3 (3.0) + DBU (0.2)	Standard	60	11
4	DIPEA (3.0) + DBU (0.2)	Standard	25	16
5	NMI (3.0) + DBU (0.2)	Standard	25	6
6	DBU (3.2)	Standard	25	10
7	K_3PO_4 (3.0) + DBU (0.2)	Standard	25	8
8	K_3PO_4 (3.0) + 18-Crown-6 (0.2)	Standard	25	14
9	K_3PO_4 (3.0) + 18-Crown-6 (0.2) + DBU (0.2)	Standard	25	16
10	K_3PO_4 (3.0)	Standard	25	11
11	Cs_2CO_3 (3.0) + DBU (0.2)	Double addition	25	13
12	Cs_2CO_3 (3.0) + DBU (0.2)	Double addition	60	10

DIPEA = Diisopropylethylamine, NMI = N-methylimidazole.

As the cyclization of **2f** seemed troublesome, we considered benchmarking the reactivity of T3P by comparing it to conventional dehydrating agent such as TsCl. To this end, we applied standard conditions by simply replacing T3P with TsCl, which gave **2f** in a similar yield as with T3P (Table S2, entry 1). Replacing Cs_2CO_3 with other alkali carbonates gave no product at all (entries 2-4). This suggests the enhanced solubility provided by cesium-ions is critical. Organic bases were non-productive (entries 5 and 6).

Table S2. Optimization details for six membered cyclic carbamate 8b

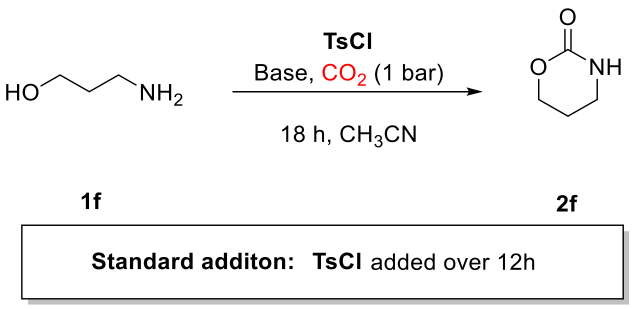

Entry	Condition	Reagent addition	Temperature (°C)	A_{rel} of **2f** (%)
1	Cs_2CO_3 (3.0) + DBU (0.2)	Standard	25	8
2	Li_2CO_3 (3.0) + DBU (0.2)	Standard	25	0
3	Na_2CO_3 (3.0) + DBU (0.2)	Standard	25	0
4	K_2CO_3 (3.0) + DBU (0.2)	Standard	25	0
5	NMI (3.0)	Standard	25	0
6	DIPEA (3.0)	Standard	25	3

DIPEA = Diisopropylethylamine, NMI = N-methylimidazole, TsCl = *p*-Toluenesulfonyl chloride.

5.4 Synthetic Procedures

Condition A – Standard Procedure

An oven-dried 300 ml Schlenk tube with a large oval magnetic stir bar was connected to a Schlenk line and placed under oil pump vacuum. Once cooled to room temperature, the tube was vented and Cs_2CO_3 (3.9 g, 3.0 equiv) was added. The Schlenk tube was evacuated and filled with CO_2 (1 bar) gas. The corresponding amino alcohol (4 mmol) was added to a 400 ml beaker and dissolved in 60 ml ACN, unless indicated otherwise. The solution added to the Schlenk tube under CO_2 flow. Typically, a white precipitate formed. Then, DBU (120 µl, 20 mol%) was added, and a septum was installed to the Schlenk tube. An empty balloon connected to a syringe was pierced through the septum (**Figure S4**). The balloon was filled with CO_2, the side tap of the Schlenk tube was closed, and the Schlenk tube was disconnected from the Schlenk line.

Commercial 50% T3P in DMF solution (4.56 ml, 2 eq) was added to an 8 ml vial and diluted with acetonitrile (4 ml). The solution was taken up in a syringe and added through the septum to the Schlenk tube over 4 h using a syringe pump, unless indicated otherwise. The reaction mixture was stirred overnight at room temperature.

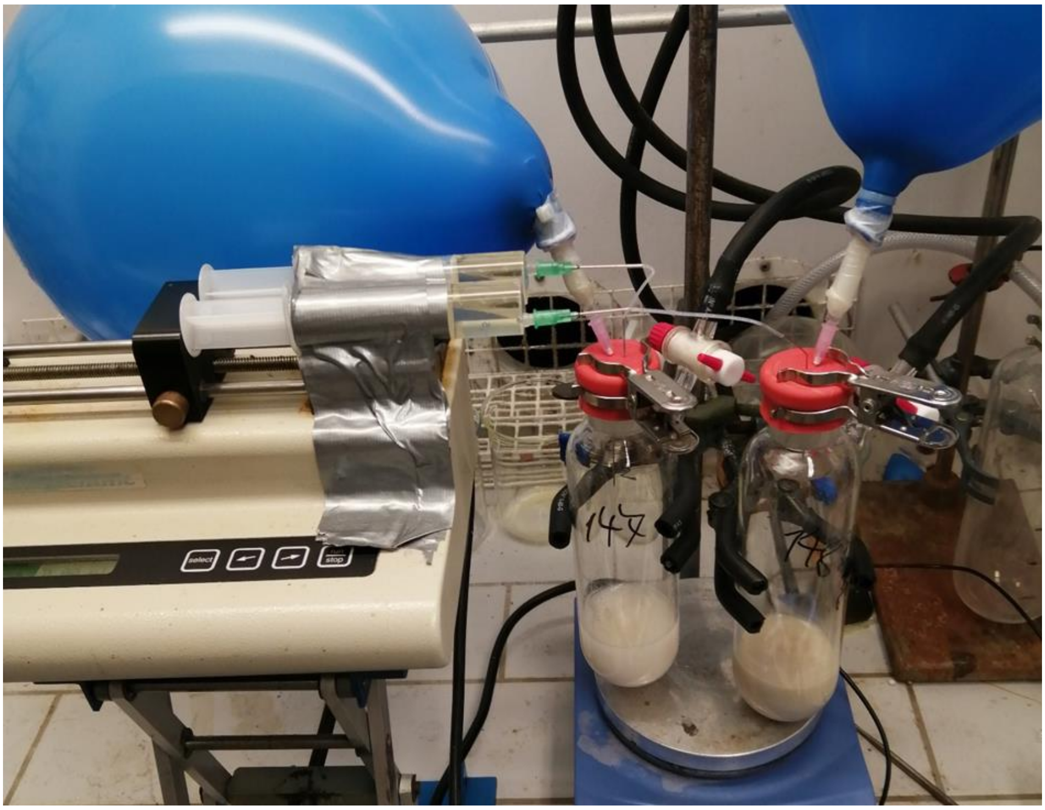

Figure S4. The experimental setup for condition A.

Condition B – Dual Addition

An oven-dried 300 ml Schlenk tube with a large oval magnetic stir bar was connected to a Schlenk line and placed under oil pump vacuum. Once cooled to room temperature, the tube was vented and Cs_2CO_3 (3.9 g, 3.0 equiv) was added. The Schlenk tube was evacuated and filled with CO_2 (1 bar) gas, followed by addition of 60 ml ACN and DBU (120 µl, 20 mol%) under CO_2 flow. A balloon was connected as described above (condition A).

The corresponding amino alcohol (4 mmol) was dissolved in 8 ml dry DMF, and the solution taken up in a syringe. T3P was taken up in a separate syringe as described above (condition A). The solutions were added simultaneously through the septum to the Schlenk tube over 12 h using a syringe pump, unless indicated otherwise. The reaction mixture was stirred overnight at room temperature.

Condition C – TsCl Instead of T3P

The procedure was done similarly to condition A, but TsCl was used instead of T3P. TsCl (8 mmol, 1525 mg) was dissolved in 8 ml ACN. The TsCl solution was taken up in a syringe equipped with a large internal diameter steel needle (**Figure S5**). This was done because TsCl was found to corrode and clog standard needles (condition A). Addition was done over 4 h, unless specified otherwise. TsCl is stable in ACN solution, but appears to decompose in DMF.

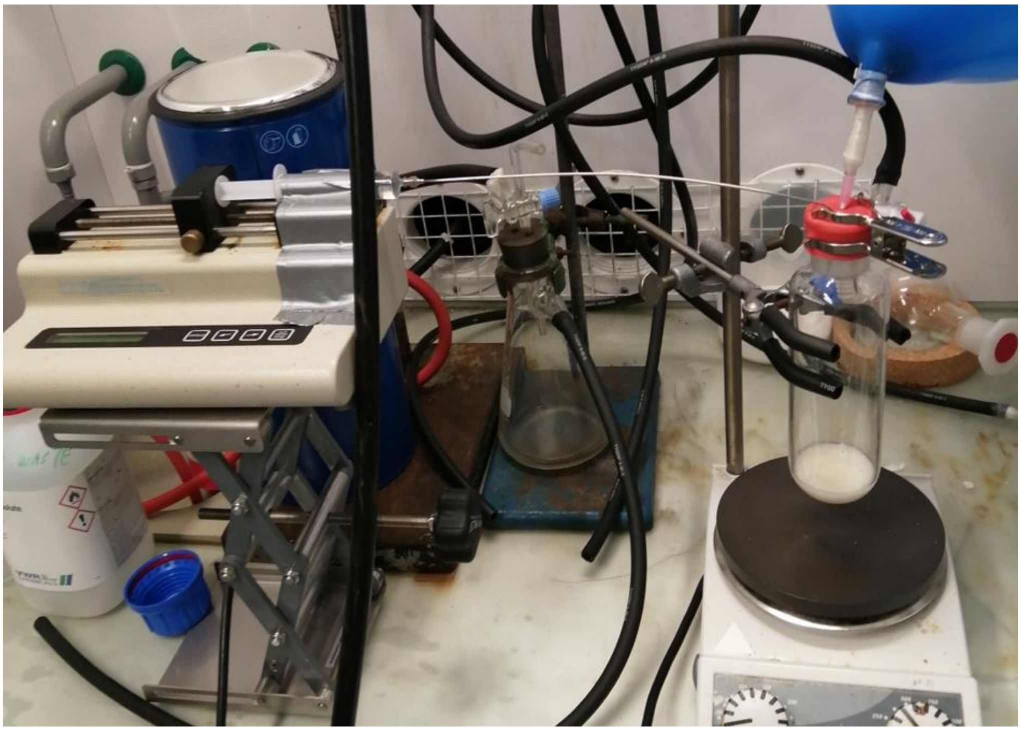

Figure S5. The experimental setup for condition C.

Workup Procedure

50 ml Saturated NH_4Cl-solution was added to the reaction mixture. The aqueous phase was extracted with 3 x 50 ml of 3:1 EtOAc:toluene solution. The organic layers were combined, and dried using anhydrous Na_2SO_4 unless indicated otherwise. The solution was filtered and solvents were evaporated in rotary evaporator.

Condition D – Reference Compound Synthesis Using CDI

A known method was applied.[10]

To an oven-dried 250 ml two-neck round-bottom flask with a stir bar was added corresponding amino alcohol (6 mmol). The apparatus was connected to a Schlenk line, evacuated, and filled with argon. Under argon flow, DCM (100 ml) was added and a pressure-equalized addition funnel was connected. The flask was placed in an ice water bath. CDI (7.2 mmol, 1.17 g) was dissolved in 50 ml DCM and added to the addition funnel. The CDI solution was added dropwise over 1 h. After addition was complete, the ice water bath was removed and the flask was left to stir for 2 h at room temperature. Afterwards, the solution was poured into a separatory funnel along with 100 ml 1M HCl. The phases were separated, organic layer collected, and the aqueous layer extracted 2 x 50 ml DCM. The organics were combined and dried over anhydrous Na_2SO_4. Filtration and evaporation of the solvent gave pure product.

6. Characterization Data of Isolated Carbamates

Compound 2a

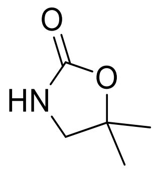

According to condition A. After the reaction, the solvent was evaporated, and then the standard workup procedure as followed. The crude product was washed with hexane to yield a white solid (363 mg, 79%).

^{1}H NMR (400 MHz, DMSO-d_6, 25 °C): δ 7.30 (1H, s), 3.21 (2H, s), 1.35 (6H, m).

^{13}C{^{1}H} NMR (102.8 MHz, DMSO-d_6, 25 °C): δ 27.32 (CH_3), 52.12 (COONH-**C**H), 79.98 (NHCOO-**C**), 158.67 (NHCOO).

HRMS (ESI-TOF) Calculated for $C_5H_9NNaO_2^+$ $[M+Na]^+$ 138.0526; found 138.0528

Compound 2b

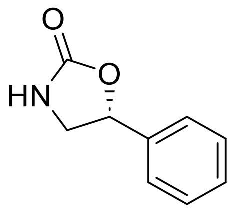

Using T3P: According to condition A. After the reaction, the solvent was evaporated, and then the standard workup procedure as followed. The crude product was dissolved in EtOAc and eluted with EtOAc through a small silica plug, yielding a white solid (525 mg, 80%).

Using TsCl: According to condition C. After the reaction, the solvent was evaporated, and then the standard workup procedure as followed. The crude product was purified by column chromatography eluting with 1:1 hexane:EtOAc (R_f = 0.26). This yielded a white solid (399 mg, 61%). The reaction produces a significantly more complex mixture compared to T3P as a dehydrating agent (condition A).

Using CDI: (*R*)- and (*S*)-enantiomers of **2b** were synthesized according to condition D using commercial enantiopure amino alcohols. Both enantiomers were obtained as white solids.

(*R*)-**2b**: 686 mg, 70%, (*S*)- **2b**: 773 mg, 79%. Racemic (*rac*)-**2b** was made by mixing (*R*)- and (*S*)-enantiomers in 1:1 ratio.

^{1}H NMR (400 MHz, $CDCl_3$, 25 °C): δ 7.41 (5H, m), 6.08 (1H, s), 5.65 (1H, m), 4.01 (1H, m), 3.58 (1H, m).

^{13}C{^{1}H} NMR (102.8 MHz, $CDCl_3$, 25 °C): δ 48.35 (COONH-**C**H_2), 77.93 (NHCOO-**C**H), 125.70 (ArCH), 128.93 (ArCH), 138.40 (ArC), 159.88 (NHCOO).

HRMS (ESI-TOF) Calculated for $C_9H_9NNaO_2^+$ $[M+Na]^+$ 186.0526; found 186.0533

Compound 2c

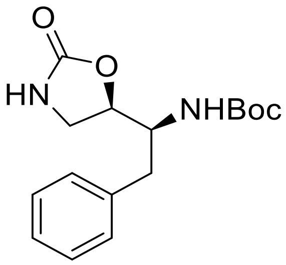

Using T3P: According to condition A in DMF. After the reaction, the solvent was evaporated, and then the standard workup procedure as followed. The crude product was purified by column chromatography eluting with 1:2 hexane:EtOAc and visualized with $KMnO_4$-stain (R_f = 0.33). This yielded a white solid (1000 mg, 83%). A comparative ^{1}H NMR analysis of the crude product and purified product in DMSO-d_6 shows only one diastereomer to be present, see spectra on pages S50 – S53. Traces of toluene are present in the crude.

Using TsCl: According to condition C in DMF. TsCl dissolved in ACN and added over 12 h. Isolation as described above gave a white solid (701 mg, 57%).

^{1}H NMR (400 MHz, DMSO-d_6, 25 °C): δ 7.53 (1H, s), 7.23 (5H, m), 6.94 (1H, d, *J* = 9.3 Hz), 4.47 (2H, m), 3.83 (1H, m), 3.51 (1H, t, *J* = 8.8 Hz), 3.35 (1H, m, partial overlap with residual water), 2.85 (1H, dd, *J* = 13.8, 3.5 Hz), 2.58 (1H, dd, *J* = 13.6, 10.7 Hz), 1.27 (9H, s).

^{1}H NMR (400 MHz, $CDCl_3$, 25 °C): δ 7.32 (2H, t), 7.23 (3H, t), 5.50 (1H, s), 4.50 (2H, m), 4.06 (1H, m), 3.63 (1H, m), 3.50 (1H, m) 3.03 (1H, m), 2.89 (1H, m), 1.36 (9H, s).

$^{13}C\{^1H\}$ NMR (102.8 MHz, $CDCl_3$, 25 °C): δ 28.22 (CH_3), 35.86 (COONH-**C**H_2), 43.21 (CH_2), 53.70 (COONH-**C**H_2), 76.96 (NHCOO-**C**), 80.14 (NHCOO-**C**H), 126.85 (ArCH), 128.69 (ArCH), 129.56 (ArCH), 136.24 (ArC), 155.46 (NHCOO), 159.18 (NHCOO).

HRMS (ESI-TOF) Calculated for $C_{16}H_{22}N_2NaO_4^+$ $[M+Na]^+$ 329.1472; found 329.1467

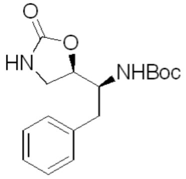

2c (crude)

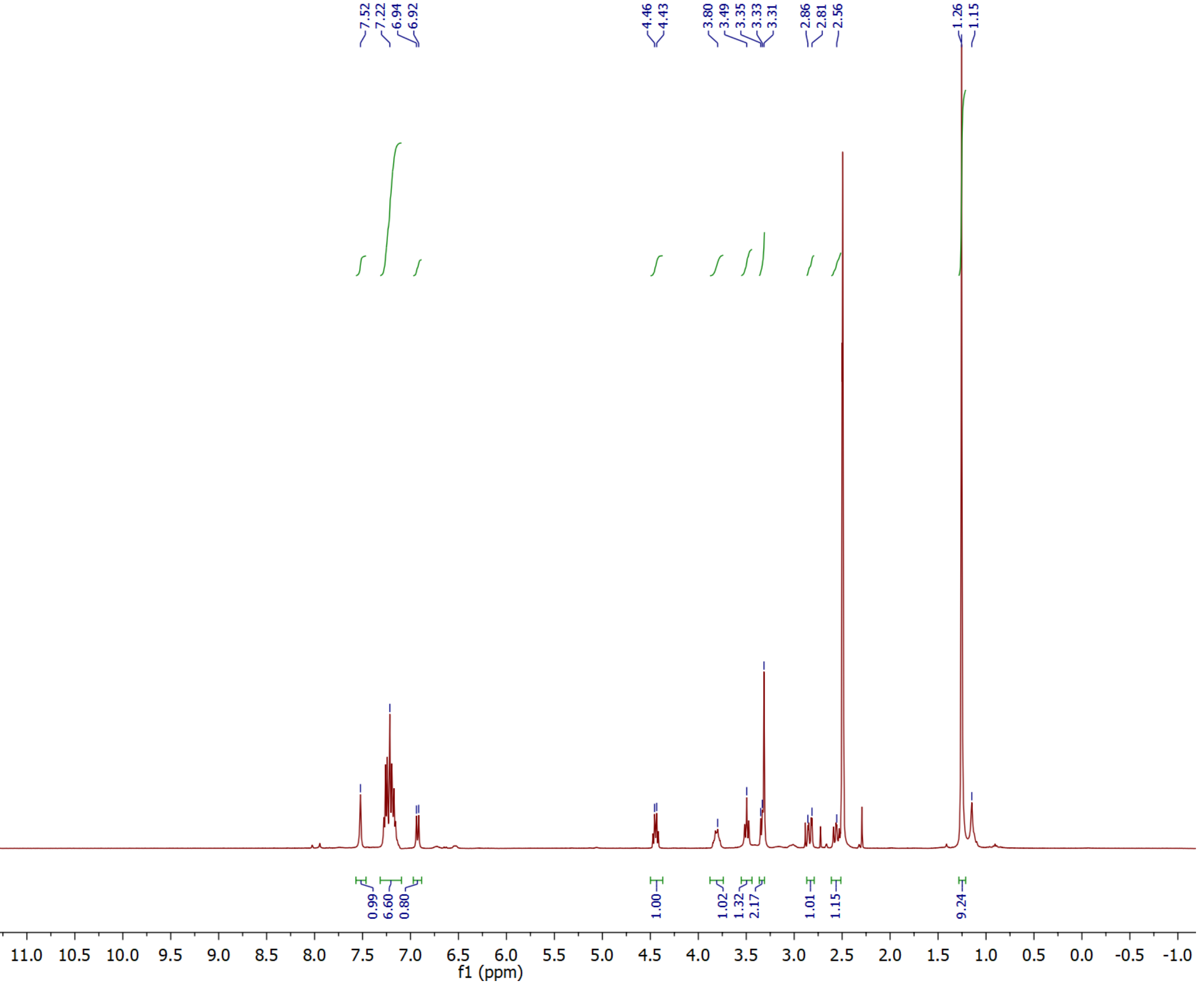

^{1}H Spectrum of crude compound **2c** in DMSO-d_6 at 25°C. Traces of toluene are present. ^{1}H-frequency 400 MHz spectrometer.

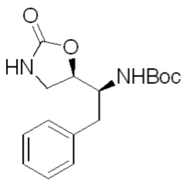

2c (purified)

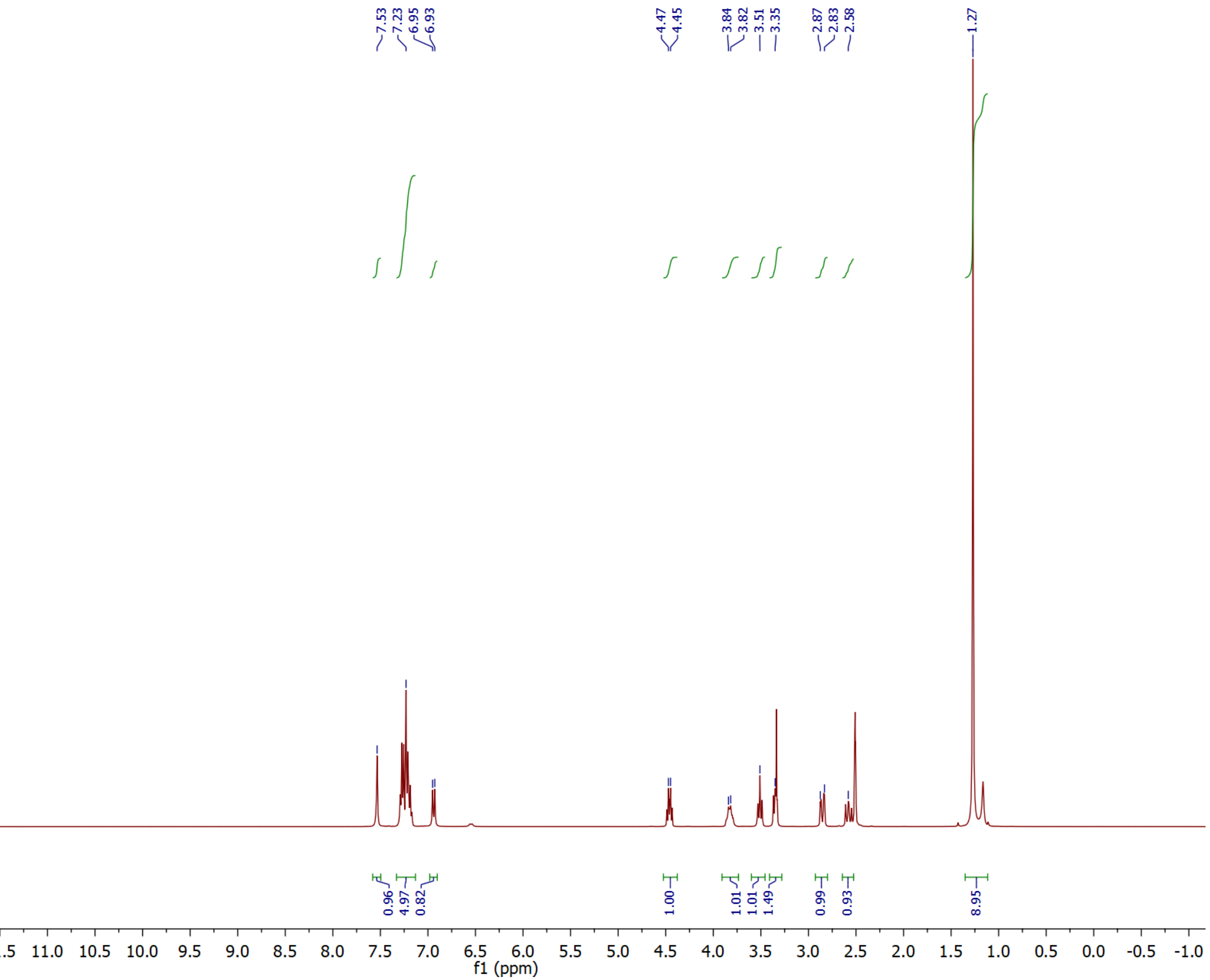

[1]H Spectrum of purified compound **2c** in DMSO-d_6 at 25°C. [1]H-frequency 400 MHz spectrometer.

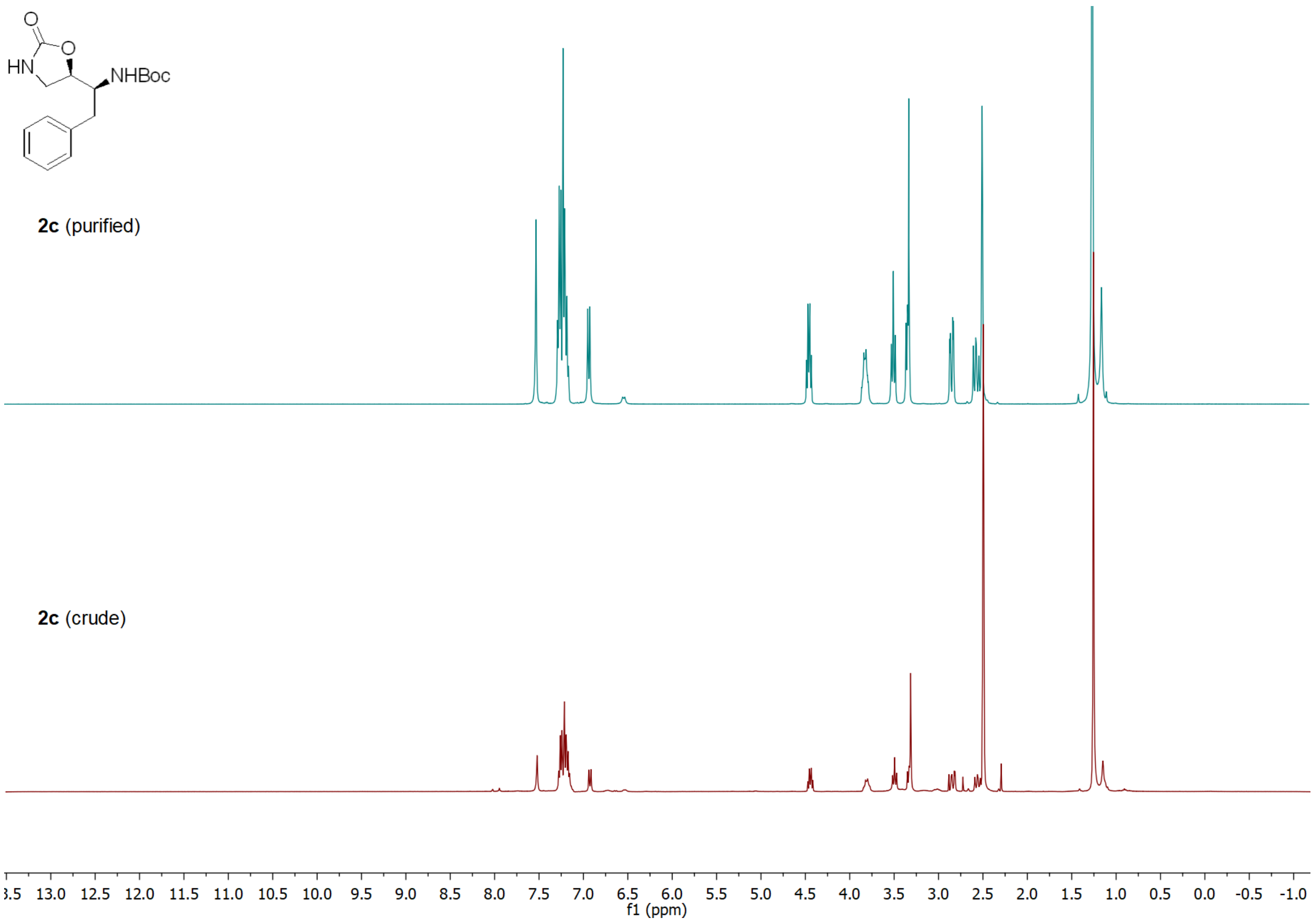

Stacked ^1H Spectra of crude and purified compound **2c** in DMSO-d_6 at 25°C. ^1H-frequency 400 MHz spectrometer.

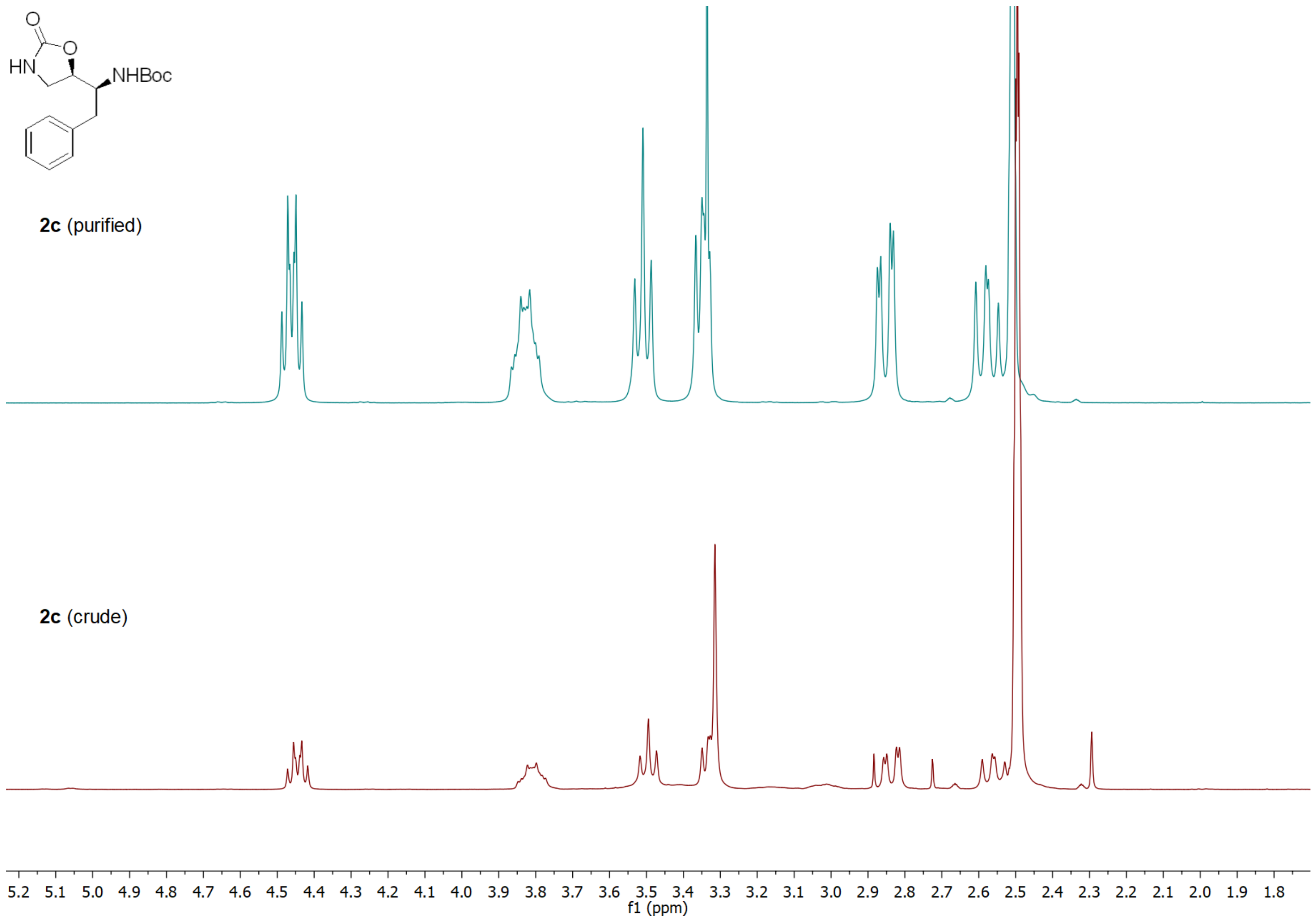

Stacked ^{1}H Spectra of crude and purified compound **2c** in DMSO-d_6 at 25°C. Selected region. ^{1}H-frequency 400 MHz spectrometer.

Compound 2d

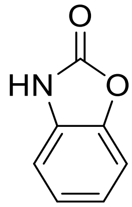

According to condition A. T3P added over 12 h. After the reaction, the solvent was evaporated, and then the standard workup procedure as followed. The crude product was dissolved in ethyl acetate and eluted with ethyl acetate through a small silica plug, yielding a reddish brown solid (318 mg, 59%).

^{1}H NMR (400 MHz, $CDCl_3$, 25 °C): δ 7.39 (1H, s), 7.20 (5H, m).

^{13}C{^{1}H} NMR (102.8 MHz, $CDCl_3$, 25 °C): δ 110.17 (ArCH), 122.79 (ArCH), 124.23 (ArCH), 129.35 (COONH-Ar**C**), 143.90 (NHCOO-Ar**C**), 156.16 (NHCOO).

HRMS (ESI-TOF) Calculated for $C_7H_5NNaO_2^+$ $[M+Na]^+$ 158.0213; found 158.0208

Compound 2e

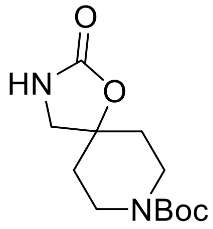

According to condition A. After the reaction, the solvent was evaporated, and then the standard workup procedure as followed. The crude product was purified by column chromatography eluting with 1:50:100 EtOH:hexane:EtOAc (R_f = 0.23). This yielded a white solid (727 mg, 71%).

^{1}H NMR (400 MHz, DMSO-d_6, 25 °C): δ 7.53 (1H, s), 3.56 (2H, m), 3.25 (4H, m), 1.72-1.66 (4H, m), 1.41 (9H, s).

^{13}C{^{1}H} NMR (102.8 MHz, DMSO-d_6, 25 °C): δ 28.52 (CH_3), 35.54 (CH_2), 40.00 (COON-**C**H_2), 50.28 (COONH-**C**H_2), 79.33 (NCOO-**C**), 79.43 (NHCOO-**C**), 154.21 (NHCOO), 158.19 (NCOO).

HRMS (ESI-TOF) Calculated for $C_{12}H_{20}N_2NaO_4^+$ $[M+Na]^+$ 279.1316; found 279.1306

Compound 2f

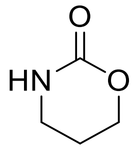

According to condition A in DMF. T3P added over 12 h. After the reaction, the solvent was evaporated, and then the standard workup procedure as followed. The crude product was purified by column chromatography eluting with 1:3 MeOH:EtOAc (R_f = 0.58). The fractions containing product were combined, evaporated, and the residue purified by column chromatography eluting with EtOAc (R_f = 0.17). This yielded a yellow oil, consisting mostly of **2f** (16 mg, 4%), as determined by ^{1}H NMR using mesitylene as an internal standard.

^{1}H NMR (400 MHz, DMSO-d_6, 25 °C): δ 4.40 (1H, m), 4.12 (2H, m), 3.14 (2H, m), 1.79 (2H, m).

^{13}C{^{1}H} NMR (102.8 MHz, DMSO-d_6, 25 °C): δ 21.40 (CH_2), 39.35 (COONH-**C**H_2), 66.65 (NHCOO-**C**H_2), 153.32 (NHCOO).

HRMS (ESI-TOF) Calculated for $C_4H_7NNaO_2^+$ $[M+Na]^+$ 124.0369; not found

Compound 2g

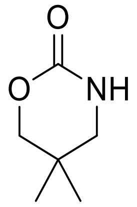

According to condition A. T3P added over 12 h. After the reaction, the solvent was evaporated, and then the standard workup procedure as followed. The crude product was recrystallized from EtOAc, yielding a white solid (345 mg, 67%).

^{1}H NMR (400 MHz, $CDCl_3$, 25 °C): δ 6.21 (1H, m), 3.92 (2H, s), 3.07 (2H, s), 1.10 (6H, s).

$^{13}C\{^{1}H\}$ NMR (102.8 MHz, $CDCl_3$, 25 °C): δ 22.67 (CH_3), 27.98 (C), 51.79 (COONH-**C**H_2), 76.12 (NHCOO-**C**H_2), 154.04 (NHCOO).

HRMS (ESI-TOF) Calculated for $C_6H_{11}NNaO_2^+$ $[M+Na]^+$ 152.0682; found 152.0680

Compound 2h

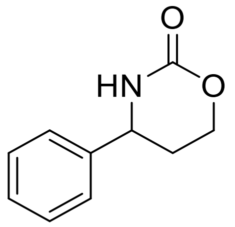

According to condition A. T3P added over 12 h. After the reaction, the solvent was evaporated, and then the standard workup procedure as followed. The crude product was dissolved in EtOAc and eluted with EtOAc through a small silica plug, yielding a white solid (528 mg, 74%).

^{1}H NMR (400 MHz, $CDCl_3$, 25 °C): δ 7.36 (5H, m), 5.66 (1H, s), 4.69 (1H, m), 4.33 (2H, m), 2.28 (1H, m), 2.02 (1H, m).

$^{13}C\{^{1}H\}$ NMR (102.8 MHz, $CDCl_3$, 25 °C): δ 30.46 (CH_2), 55.04 (COONH-**C**H_2), 64.98 (NHCOO-**C**H_2), 126.02 (ArCH), 128.41 (ArCH), 129.07 (ArCH), 141.09 (ArC), 154.02 (NHCOO).

HRMS (ESI-TOF) Calculated for $C_{10}H_{11}NNaO_2^+$ $[M+Na]^+$ 200.0682; found 200.0690

Compound 2i

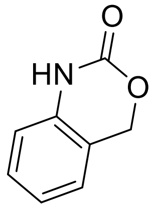

According to condition A. T3P added over 12 h. After the reaction, the solvent was evaporated, and then the standard workup procedure as followed. The crude product was purified by

column chromatography eluting with 2:1 hexane:EtOAc (R_f = 0.26). This yielded a white solid (539 mg, 90%).

^{1}H NMR (400 MHz, $CDCl_3$, 25 °C): δ 8.87 (1H, s), 7.26 (1H, m), 7.10 (2H, m), 6.89 (1H, d), 5.34 (2H, m).

^{13}C{^{1}H} NMR (102.8 MHz, $CDCl_3$, 25 °C): δ 68.73 (NHCOO-**C**H_2), 114.25 (ArCH), 117.88 (ArCH), 123.36 (ArCH), 124.21 (ArCH), 129.24 (COONH-Ar**C**), 141.09 (ArC), 135.56 (ArC), 153.76 (NHCOO).

HRMS (ESI-TOF) Calculated for $C_8H_7NNaO_2^+$ $[M+Na]^+$ 172.0369; found 172.0376

Compound 2j

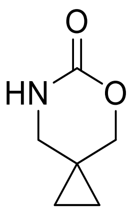

According to condition B. T3P added over 12 h. After the reaction, the solvent was evaporated, and then the standard workup procedure as followed. The crude product was dissolved in EtOAc and eluted with EtOAc through a small silica plug, yielding a white solid (229 mg, 45%).

^{1}H NMR (400 MHz, $CDCl_3$, 25 °C): δ 6.33 (1H, s), 4.02 (2H, s), 3.19 (2H, s), 0.75 (2H, m), 0.69 (2H, m).

^{13}C{^{1}H} NMR (102.8 MHz, $CDCl_3$, 25 °C): δ 9.56 (CyPrCH_2), 14.90 (CyPrC), 47.78 (COONH-**C**H_2), 73.45 (NHCOO-**C**H_2), 155.04 (NHCOO).

HRMS (ESI-TOF) Calculated for $C_6H_9NNaO_2^+$ $[M+Na]^+$ 150.0526; found 150.0523

Compound 2k

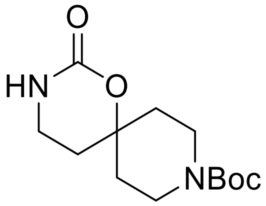

According to condition A. T3P added over 12 h. After the reaction, the solvent was evaporated, and then the standard workup procedure as followed. The crude product was purified by column chromatography with gradient elution from 2:100 to 15:100 EtOH:DCM. This yielded a pale yellow solid (497 mg, 46%).

^{1}H NMR (400 MHz, $CDCl_3$, 25 °C): δ 6.99 (s, 1H), 3.81 (s, 2H), 3.32 (td, *J* = 6.4, 2.2 Hz, 2H), 3.26 – 3.16 (m, 2H), 1.85 – 1.73 (m, 4H), 1.59 – 1.49 (m, 2H), 1.40 (s, 9H).

^{13}C{^{1}H} NMR (102.8 MHz, $CDCl_3$, 25 °C): δ 154.2, 154.0, 79.2, 76.7, 38.6 (br), 35.4, 34.6 (br), 30.4, 28.0.

HRMS (ESI-TOF) Calculated for $C_{13}H_{22}N_2O_4Na^+$ [M+Na]$^+$ 293.1472; found 293.146

Compound 2l

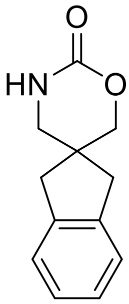

According to condition A. T3P added over 12 h. After the reaction, the solvent was evaporated, and then the standard workup procedure as followed. The crude product was washed with hexane. This yielded a pale yellow solid (768 mg, 95%).

^{1}H NMR (400 MHz, $CDCl_3$, 25 °C): δ 7.22 (4H, m), 6.58 (1H, broad), 4.16 (2H, s), 3.36 (2H, s), 3.01 (4H, q).

^{13}C{^{1}H} NMR (102.8 MHz, $CDCl_3$, 25 °C): δ 39.84 (Ar-CH_2), 40.09 (C), 50.44 (CONH-CH_2), 74.03 (COO-**C**H_2), 125.04 (ArCH), 127.04 (ArCH), 140.24 (ArC), 154.26 ($COONH_2$).

HRMS (ESI-TOF) Calculated for $C_{12}H_{13}NNaO_2^+$ $[M+Na]^+$ 226.0839; found 226.0828

Compound 2m

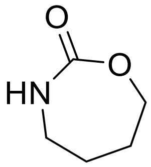

According to condition A. T3P added over 12 h. After the reaction, the solvent was evaporated, and then the standard workup procedure as followed. No product was obtained.

Using CDI: According to condition D, reaction scaled to 1.0 mmol. A clear oil was obtained (4 mg, 4%).

^{1}H NMR (400 MHz, $CDCl_3$, 25 °C): δ 5.17 (1H, br), 4.18 (2H, m), 3.20 (2H, dd, *J* = 10.6, 4.8 Hz), 1.93 (2H, m), 1.77 (2H, m).

^{13}C{^{1}H} NMR (102.8 MHz, $CDCl_3$, 25 °C): δ 162.2, 70.4, 42.8, 29.4, 27.2

HRMS (ESI-TOF) Calculated for $C_5H_9NNaO_2^+$ $[M+Na]^+$ 138.0526; found 138.0524

Compound 2n

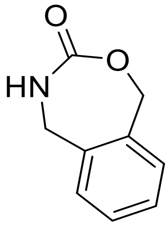

According to condition A. T3P added over 12 h. After the reaction, the solvent was evaporated, and then the standard workup procedure as followed. The crude product was purified by column chromatography with 1:40 MeOH:EtOAc (R_f = 0.33). This yielded a white solid (307 mg, 47%).

Using CDI: According to condition D, reaction scaled to 1.0 mmol. A white solid was obtained (25 mg, 15%).

^{1}H NMR (400 MHz, $CDCl_3$, 25 °C): δ 7.35 (3H, m), 7.23 (1H, m), 6.44 (1H, s), 5.21 (2H, s), 4.37 (2H, s).

^{13}C{^{1}H} NMR (102.8 MHz, $CDCl_3$, 25 °C): δ 45.25 (COONH-**C**H_2), 68.62 (NHCOO-**C**H_2), 127.39 (ArCH), 128.36 (ArCH), 128.56 (ArCH), 129.50 (ArCH), 135.32 (ArC), 137.06 (ArC), 156.57 (NHCOO).

HRMS (ESI-TOF) Calculated for $C_9H_9NNaO_2^+$ $[M+Na]^+$ 186.0526; found 186.0529

Compound 2o

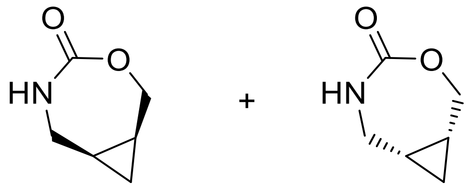

According to condition B. T3P added over 12 h. After the reaction, the solvent was evaporated, and then the standard workup procedure as followed. The crude product was purified by column chromatography eluting with 5:100 MeOH:EtOAc (R_f = 0.18). This yielded a white solid (167 mg, 33%). The product is a 1:1 racemic mixture.

Using CDI: According to condition D, reaction scaled to 1.0 mmol. No product was obtained.

^{1}H NMR (400 MHz, $CDCl_3$, 25 °C): δ 6.14 (1H, s), 4.44 (1H, m), 4.05 (1H, m), 3.45 (1H, m), 3.16 (1H, m), 1.60 (1H, s), 0.88 (1H, m), 0.51 (1H, m).

^{13}C{^{1}H} NMR (102.8 MHz, $CDCl_3$, 25 °C): δ 11.16 (CyPrCH_2), 14.17 (CyPrCH), 14.73 (CyPrCH), 44.00 (COONH-**C**H_2), 69.58 (NHCOO-**C**H_2), 157.48 (NHCOO).

HRMS (ESI-TOF) Calculated for $C_6H_9NNaO_2^+$ $[M+Na]^+$ 150.0526; found 186.0526

Compound 2p

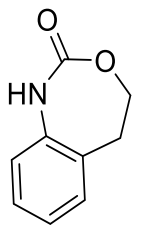

According to condition A. T3P added over 12 h. After the reaction, the solvent was evaporated, and then the standard workup procedure as followed. The crude was purified by column chromatography eluting with 1:1 hexane:EtOAc. This yielded a pale pink solid (404 mg, 62%).

^{1}H NMR (400 MHz, $CDCl_3$, 25 °C): δ 8.49 (s, 1H), 7.17 (td, *J* = 7.7, 1.6 Hz, 1H), 7.09 (dd, *J* = 7.6, 1.5 Hz, 1H), 6.98 (ddd, *J* = 13.9, 7.6, 1.2 Hz, 2H), 4.52 (dt, *J* = 2274.5, 4.8 Hz, 2H), 3.21 (dt, *J* = 916.3, 4.8 Hz, 2H)

^{13}C{^{1}H} NMR (102.8 MHz, $CDCl_3$, 25 °C): δ 156.7, 136.3, 130.5, 128.0, 126.4, 123.3, 119.6, 68.2, 34.6

HRMS (ESI-TOF) Calculated for $C_9H_9NO_2Na^+$ $[M+Na]^+$ 186.0525; found 186.0518

Compound 2q

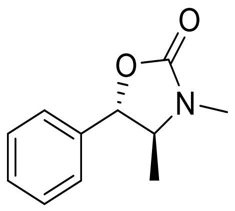

According to condition A, but reaction scaled down to 1.6 mmol from 4.0 mmol. After the reaction, the solvent was evaporated, and then the standard workup procedure as followed. The crude product was dissolved in small amount of DCM and adsorbed onto a small silica pad. The pad was washed with 40 ml 1:6 EtOAc:hexane to remove impurities. The product was then eluted with 40 ml of EtOAc. This yielded a white solid (188 mg, 61 %).

To confirm the identity of **2q**, a ^{1}H NMR sample obtained using T3P (condition A) was spiked with **2q** independently prepared using CDI (see below). No new signals were observed after spiking, indicating that the two compounds are indeed the same and no inversion had taken place. The inversion product **2r** was also independently synthesized (see below), confirming that its ^{1}H spectrum differs significantly from that of **2q** (see stacked spectra on page S64).

^{1}H NMR (400 MHz, $CDCl_3$, 25 °C): δ 7.45 – 7.31 (m, 5H), 4.90 (d, *J* = 7.8 Hz, 1H), 3.54 (dq, *J* = 7.8, 6.2 Hz, 1H), 2.86 (s, 3H), 1.36 (d, *J* = 6.2 Hz, 3H)

^{13}C{^{1}H} NMR (102.8 MHz, $CDCl_3$, 25 °C): δ 157.82, 137.68, 128.95, 128.87, 125.92 (d, *J* = 1.9 Hz), 82.45, 61.33, 28.80, 17.36

HRMS (ESI-TOF) Calculated for $C_9H_9NO_2Na^+$ $[M+Na]^+$ 186.0525; found 186.0518

Synthesis of 2q Reference Material – Retention of Configuration

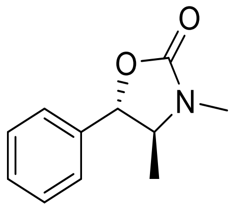

Cyclization product **2q** was independently synthetized by a slightly modified literature method, which is known to proceed with retention of configuration.[11,12]

To a 50 ml flask was added (+)-pseudoephedrine (330 mg, 2 mmol), which was dissolved in dry THF (20 ml). 1.1'-Carbonyldiimidazole (332 mg, 1.05 equiv) was added, followed by DBU (298.6 µl, 2 equiv). The solution was then refluxed until no staring material was observable on TLC (ca 2 h). The solution was cooled to room temperature, and then poured into a separatory funnel along with 50 ml water. The aqueous phase was extracted with 2 x 20 ml of EtOAc, the organics were combined and dried with Na_2SO_4. Filtration and evaporation of the solvent afforded a yellow oil (324 mg, 85 %).

Synthesis of 2r Reference Material – Inversion of Configuration

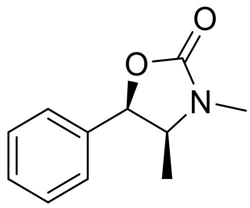

According to published procedure.[2]

To a 50 ml flask was added (+)-pseudoephedrine (5 mmol) and Boc_2O (1.1 equiv, 5.5 mmol), which were dissolved in DCM (30 ml). Triethylamine (1.2 equiv, 6 mmol) was added and the solution was stirred at room temperature for 18 hours. The solution was poured into a separatory funnel along with 100 ml of water and 30 ml of DCM. The layers were separated and the organic layer collected, which was washed with saturated NaCl solution and then dried over anhydrous Na_2SO_4. The solution was filtered and the solvent evaporated. The solid residue was used in the next step without further purification.

To a 50 ml flask containing the crude Boc-protected (+)-pseudoephedrine was added pyridine (13 ml) and TsCl (3 equiv, 15 mmol). The solution was refluxed overnight (18 hours) and cooled to room temperature. The solution was poured into a separatory funnel along with 100 ml of water and 50 ml of EtOAc. The phases were separated, organic layer collected, which was washed with 50 ml of water and 50 ml of brine, and then dried over anhydrous Na_2SO_4. The solvent was evaporated and crude product purified with column chromatography using a gradient of 15% to 50% EtOAc in hexane. The fractions containing product were combined and the solvent evaporated. The crude consisted mostly of **2r**, although **2q** was present as a minor component. The crude was recrystallized from DCM:hexane, yielding transparent crystals of diastereomerically pure **2r** (325 mg, 34%). NMR data matches literature.[13]

^{1}H NMR (400 MHz, $CDCl_3$, 25 °C): δ 7.43 – 7.29 (m, 3H), 7.30 – 7.23 (m, 2H), 5.58 (d, *J* = 8.2 Hz, 1H), 4.02 (dq, *J* = 8.2, 6.6 Hz, 1H), 2.88 (s, 3H), 0.78 (d, *J* = 6.6 Hz, 3H).

^{13}C{^{1}H} NMR (102.8 MHz, $CDCl_3$, 25 °C): δ 158.1, 135.2, 128.5, 128.5, 126.2, 78.4, 57.1, 29.0, 14.3.

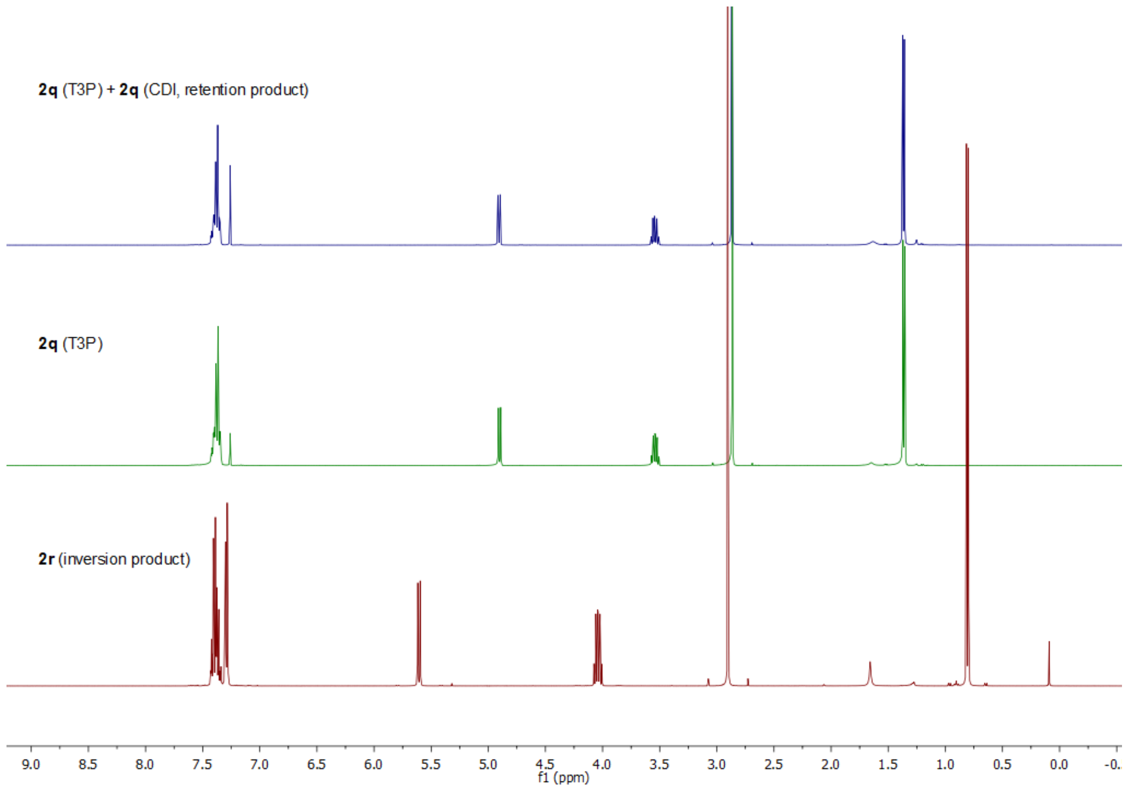

Stacked ^{1}H specta of compounds **2q** and **2r** in $CDCl_3$ at 25°C (selected region). [1]H-frequency 400 MHz spectrometer.

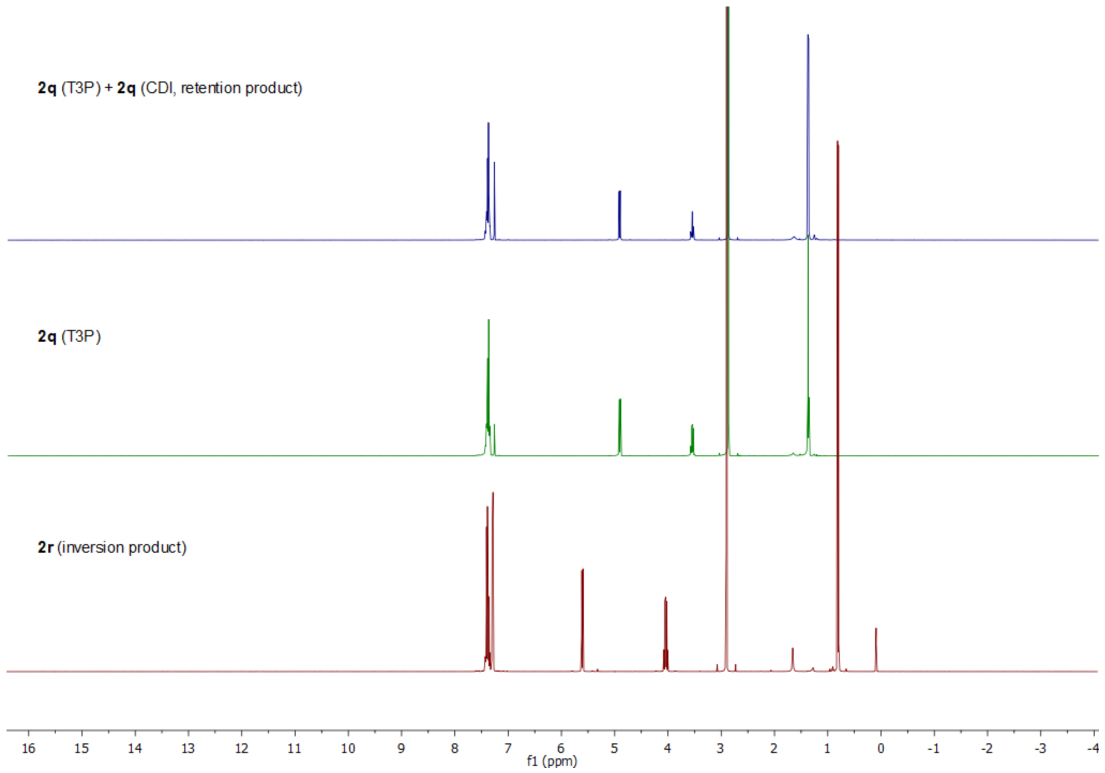

Stacked ^{1}H specta of compounds **2q** and **2r** in $CDCl_3$ at 25°C. [1]H-frequency 400 MHz spectrometer.

7. Chiral HPLC Analysis

Compound 2b (R-enantiomer), synthesized using CDI

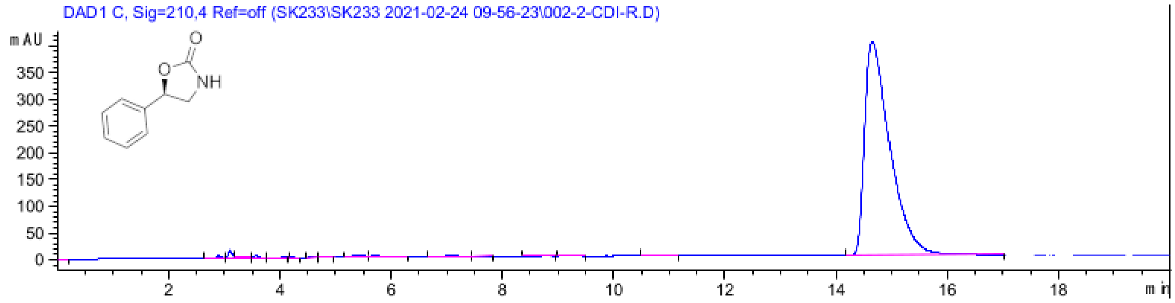

Signal 3: DAD1 C, Sig=210,4 Ref=off

Peak #	RetTime [min]	Type	Width [min]	Area [mAU*s]	Height [mAU]	Area %
1	0.047	BB	0.0752	2.30537	4.54559e-1	0.0176
2	2.898	BV	0.0793	24.35183	4.48608	0.1862
3	3.106	VV R	0.0815	69.33328	12.73506	0.5301
4	3.311	VB E	0.0809	8.34193	1.49970	0.0638
5	3.569	BB	0.0734	23.53783	4.78631	0.1800
6	4.072	BV	0.1067	5.41223	7.11522e-1	0.0414
7	4.186	VB	0.1065	3.25661	4.84210e-1	0.0249
8	4.646	BV	0.0794	3.65793	6.73644e-1	0.0280
9	4.785	VB	0.0945	15.36792	2.40710	0.1175
10	5.392	BV	0.1969	57.35923	4.32106	0.4386
11	5.706	VB	0.2703	45.29453	2.21795	0.3463
12	7.074	BV	0.2552	39.83121	2.10085	0.3046
13	7.608	VB	0.1764	7.11683	4.87331e-1	0.0544
14	8.778	BV	0.2056	9.42533	5.67847e-1	0.0721
15	9.190	VB	0.2352	15.61205	8.62284e-1	0.1194
16	10.841	BB	0.2369	9.34626	4.89127e-1	0.0715
17	14.655	BB	0.4786	1.27387e4	398.82861	97.4037

Totals : 1.30783e4 438.11325

Compound 2b (S-enantiomer), synthesized using CDI

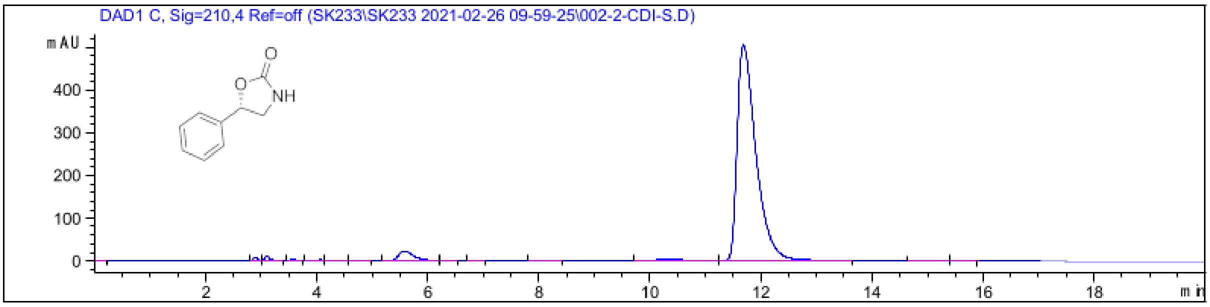

Signal 3: DAD1 C, Sig=210,4 Ref=off

Peak #	RetTime [min]	Type	Width [min]	Area [mAU*s]	Height [mAU]	Area %
1	0.042	BB	0.0751	2.23503	4.41529e-1	0.0149
2	2.762	BV E	0.1486	12.54696	1.34819	0.0839
3	2.898	VV E	0.0794	7.07879	1.30215	0.0473
4	3.106	VV R	0.0797	70.03992	13.24224	0.4682
5	3.302	VB E	0.0630	1.29060	2.95205e-1	8.627e-3
6	3.569	BB	0.0731	156.25505	31.92787	1.0445
7	4.063	BV	0.1001	11.65264	1.74071	0.0779
8	4.188	VB	0.1089	9.82344	1.31928	0.0657
9	4.750	BB	0.1607	16.15729	1.46454	0.1080
10	5.414	BV	0.2034	22.86767	1.48724	0.1529
11	5.703	VB	0.1556	14.06765	1.30698	0.0940
12	6.263	BB	0.2505	10.34899	5.02091e-1	0.0692
13	7.050	BV	0.2960	115.89538	6.14554	0.7747
14	7.324	VV	0.2987	89.13539	3.78590	0.5959
15	8.037	VB	0.2956	110.09225	5.54502	0.7359
16	8.635	BB	0.1802	11.68712	8.50963e-1	0.0781
17	9.171	BB	0.1884	14.52475	9.82764e-1	0.0971
18	10.036	BB	0.2627	23.62728	1.10849	0.1579
19	11.742	BB	0.3603	7886.02832	333.00659	52.7164
20	14.766	BB	0.4648	6373.97900	207.25912	42.6087

Totals : 1.49593e4 615.06243

Compound 2b (Racemic)

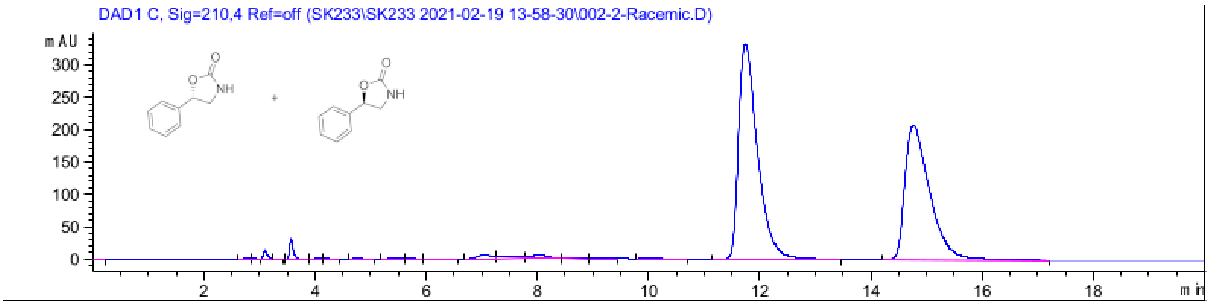

Signal 3: DAD1 C, Sig=210,4 Ref=off

Peak #	RetTime [min]	Type	Width [min]	Area [mAU*s]	Height [mAU]	Area %
1	0.042	BB	0.0751	2.23503	4.41529e-1	0.0149
2	2.762	BV E	0.1486	12.54696	1.34819	0.0839
3	2.898	VV E	0.0794	7.07879	1.30215	0.0473
4	3.106	VV R	0.0797	70.03992	13.24224	0.4682
5	3.302	VB E	0.0630	1.29060	2.95205e-1	8.627e-3
6	3.569	BB	0.0731	156.25505	31.92787	1.0445
7	4.063	BV	0.1001	11.65264	1.74071	0.0779
8	4.188	VB	0.1089	9.82344	1.31928	0.0657
9	4.750	BB	0.1607	16.15729	1.46454	0.1080
10	5.414	BV	0.2034	22.86767	1.48724	0.1529
11	5.703	VB	0.1556	14.06765	1.30698	0.0940
12	6.263	BB	0.2505	10.34899	5.02091e-1	0.0692
13	7.050	BV	0.2960	115.89538	6.14554	0.7747
14	7.324	VV	0.2987	89.13539	3.78590	0.5959
15	8.037	VB	0.2956	110.09225	5.54502	0.7359
16	8.635	BB	0.1802	11.68712	8.50963e-1	0.0781
17	9.171	BB	0.1884	14.52475	9.82764e-1	0.0971
18	10.036	BB	0.2627	23.62728	1.10849	0.1579
19	11.742	BB	0.3603	7886.02832	333.00659	52.7164
20	14.766	BB	0.4648	6373.97900	207.25912	42.6087

Totals : 1.49593e4 615.06243

Compound 2b (R-enantiomer), synthesized using T3P

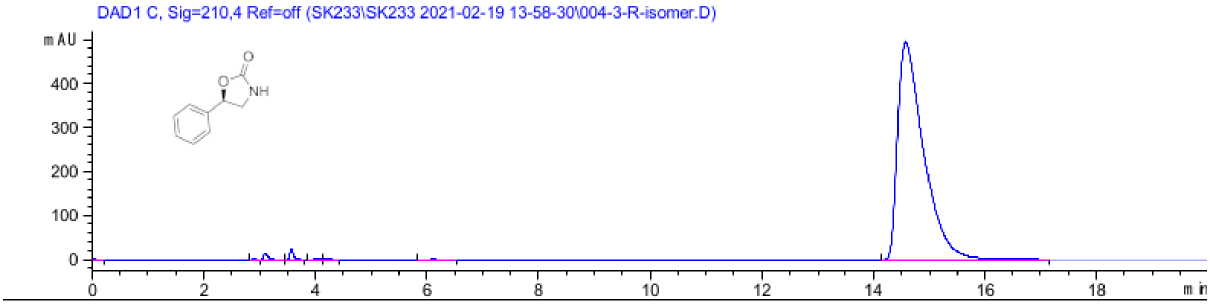

Signal 3: DAD1 C, Sig=210,4 Ref=off

Peak #	RetTime [min]	Type	Width [min]	Area [mAU*s]	Height [mAU]	Area %
1	0.042	BB	0.0813	2.65560	4.74460e-1	0.0164
2	2.905	BV E	0.0725	3.66385	7.57155e-1	0.0227
3	3.107	VB R	0.0793	71.58826	13.63275	0.4426
4	3.570	BB	0.0726	108.51421	22.37474	0.6709
5	4.064	BV	0.1046	9.36810	1.35552	0.0579
6	4.190	VB	0.1105	8.00470	1.03155	0.0495
7	6.131	BB	0.2189	7.42321	4.18574e-1	0.0459
8	14.579	BB	0.4825	1.59644e4	494.67224	98.6942

Totals : 1.61756e4 534.71699

Compound 2b (R-enantiomer), synthesized using TsCl

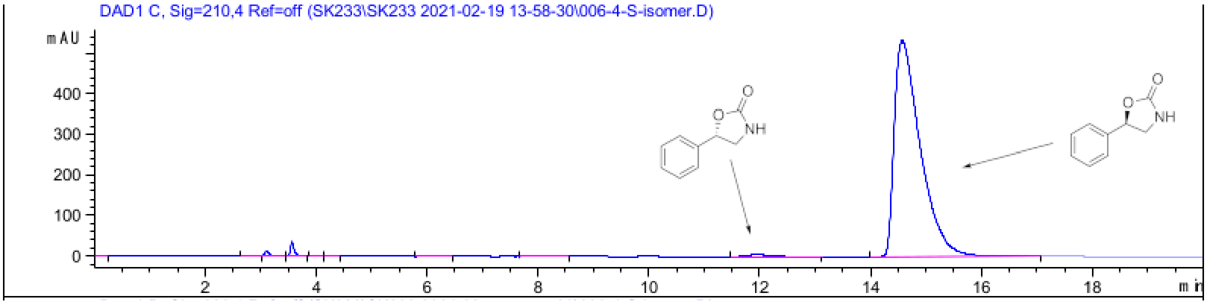

Signal 3: DAD1 C, Sig=210,4 Ref=off

Peak #	RetTime [min]	Type	Width [min]	Area [mAU*s]	Height [mAU]	Area %
1	0.045	BB	0.0758	2.63691	5.14590e-1	0.0149
2	2.903	BV E	0.1102	11.63554	1.44033	0.0657
3	3.109	VB R	0.0803	73.52049	13.76430	0.4153
4	3.571	BB	0.0725	180.61276	37.32206	1.0202
5	4.063	BV	0.0999	9.24417	1.38480	0.0522
6	4.191	VB	0.1147	8.24755	1.10970	0.0466
7	6.096	BB	0.2088	6.23541	3.65872e-1	0.0352
8	8.003	BB	0.2989	31.33416	1.27401	0.1770
9	11.956	BB	0.3561	130.67474	5.40339	0.7381
10	14.573	BB	0.4862	1.72492e4	532.04773	97.4347
Totals :				1.77033e4	594.62678	

Both reagents T3P and TsCl gave the same *R*-enantiomer as the major product. Reaction with T3P was stereoselective (>99% ee), but TsCl gave some *S*-enantiomer also (98% ee).

8. FTIR Spectra of Selected Cyclic Carbamates

Compound 2a

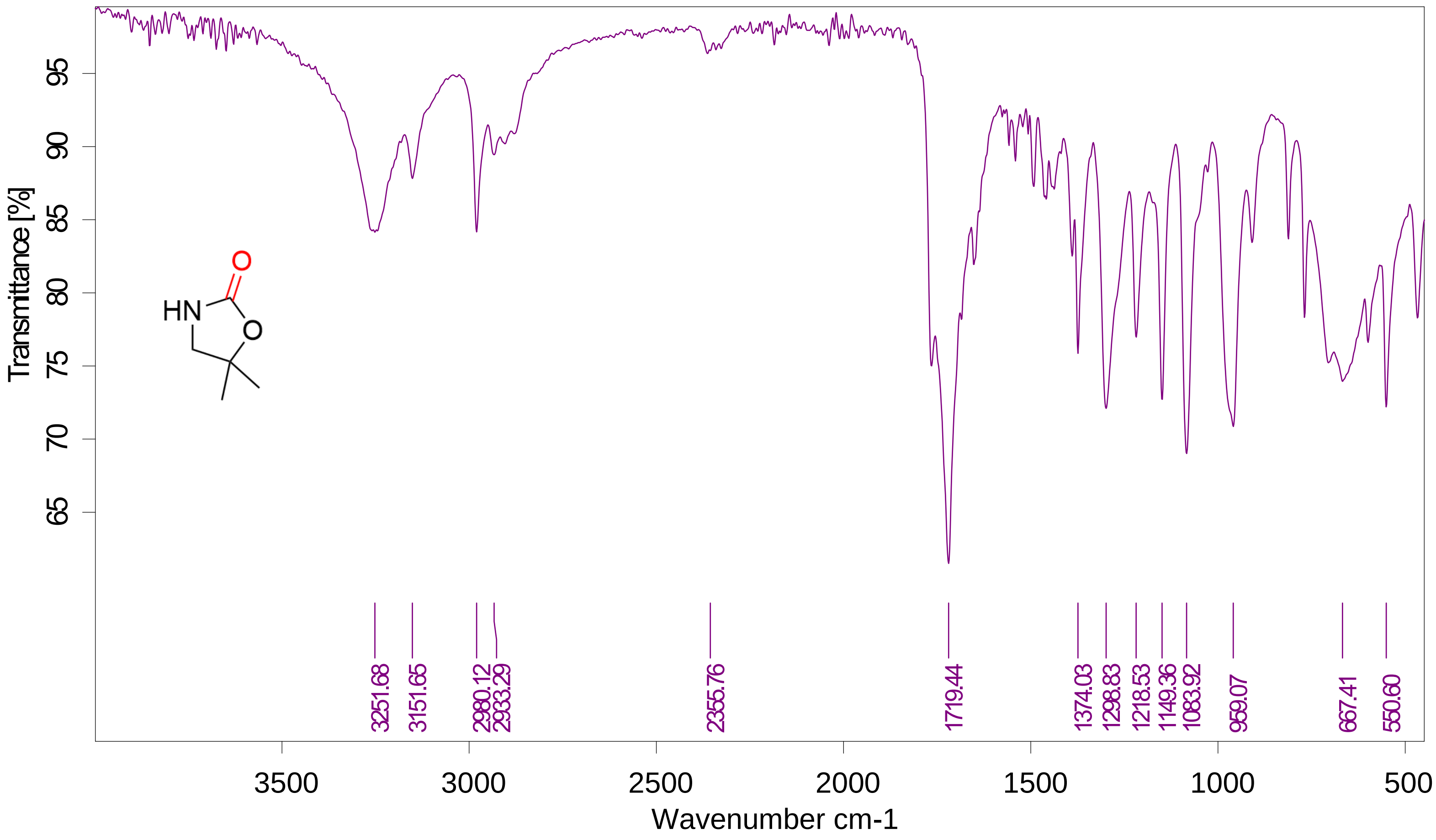

FTIR spectrum of compound **2a**.

Compound 2b

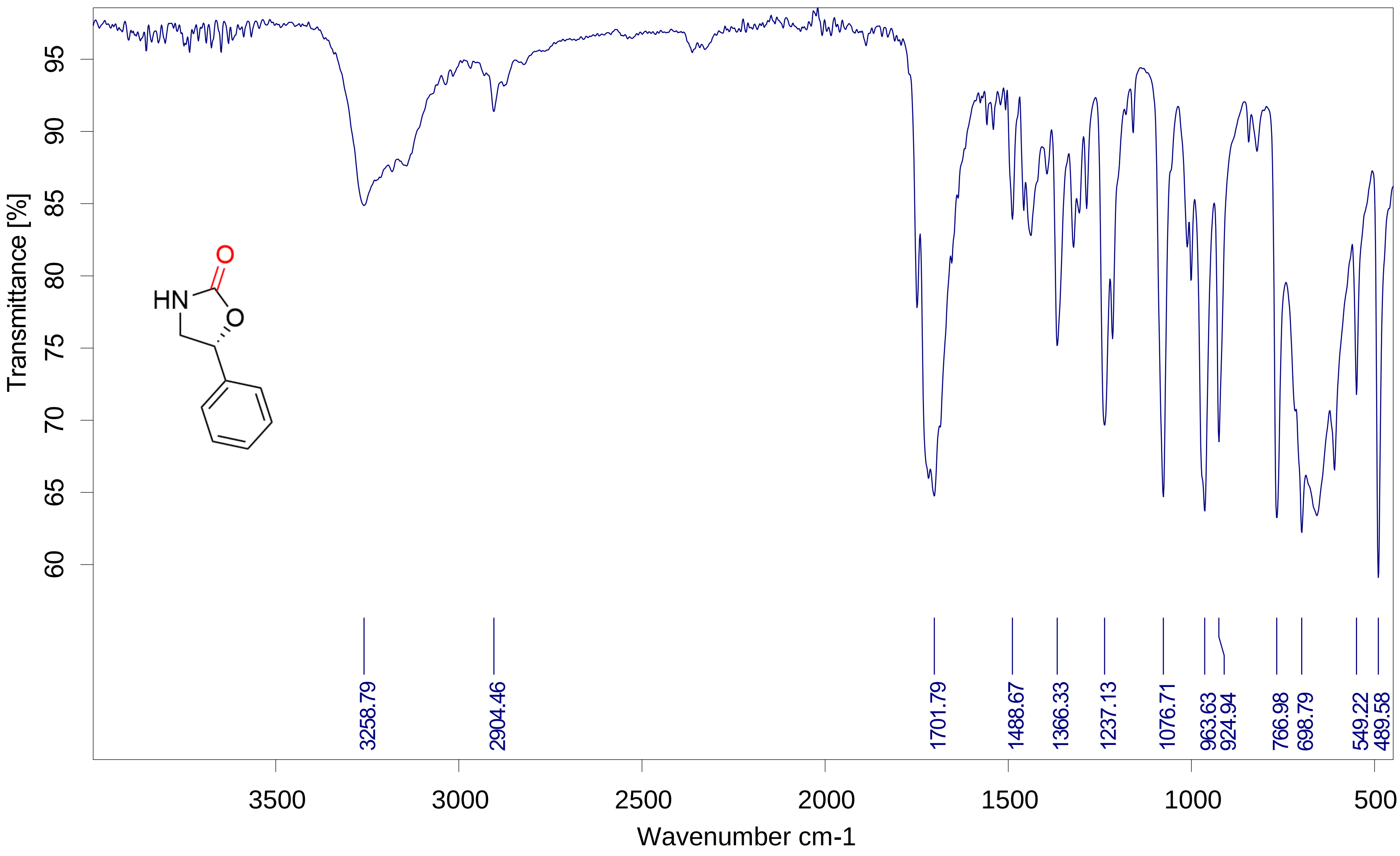

FTIR spectrum of compound **2b**.

Compound 2i

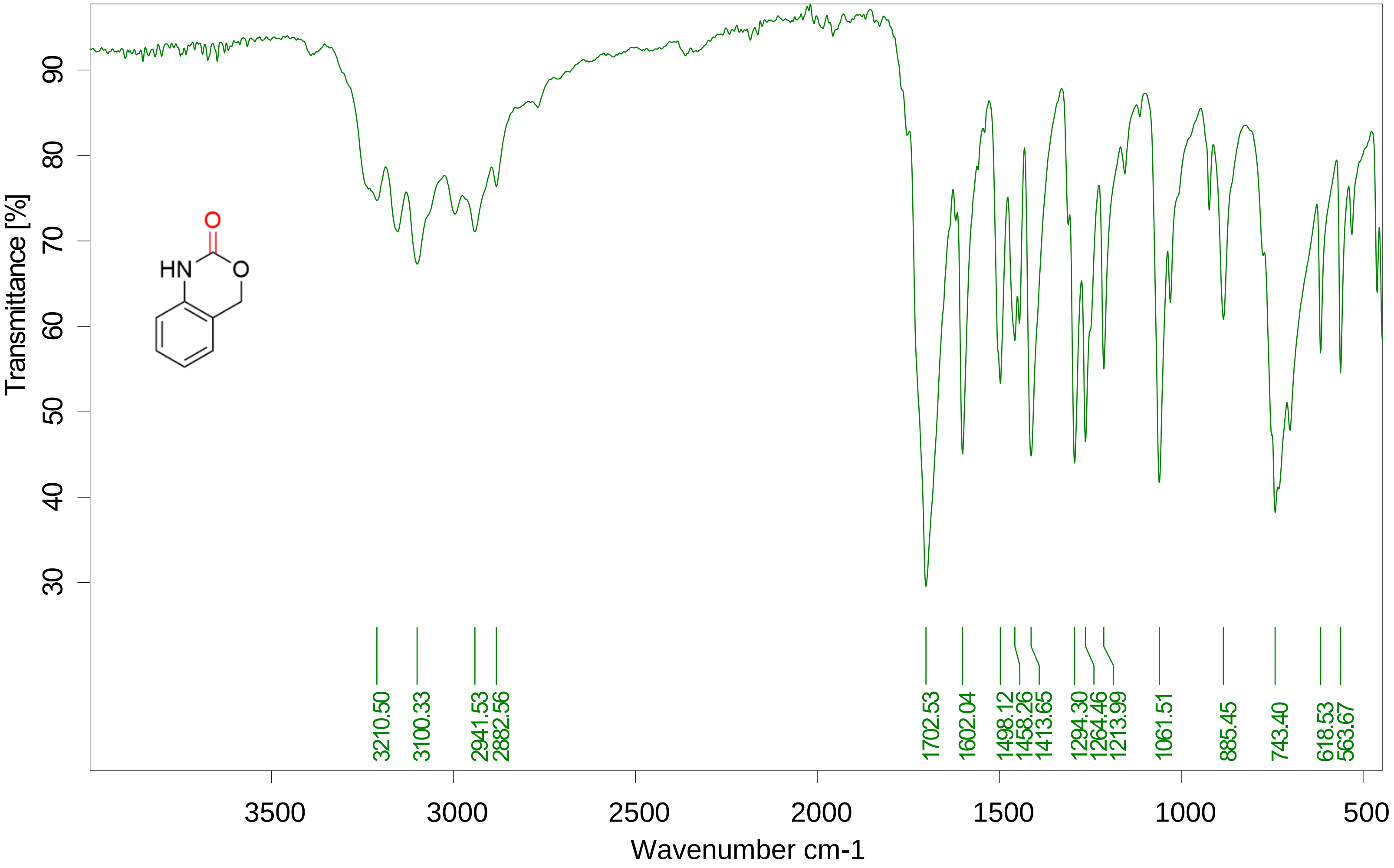

FTIR spectrum of compound **2i**.

Compound 2l

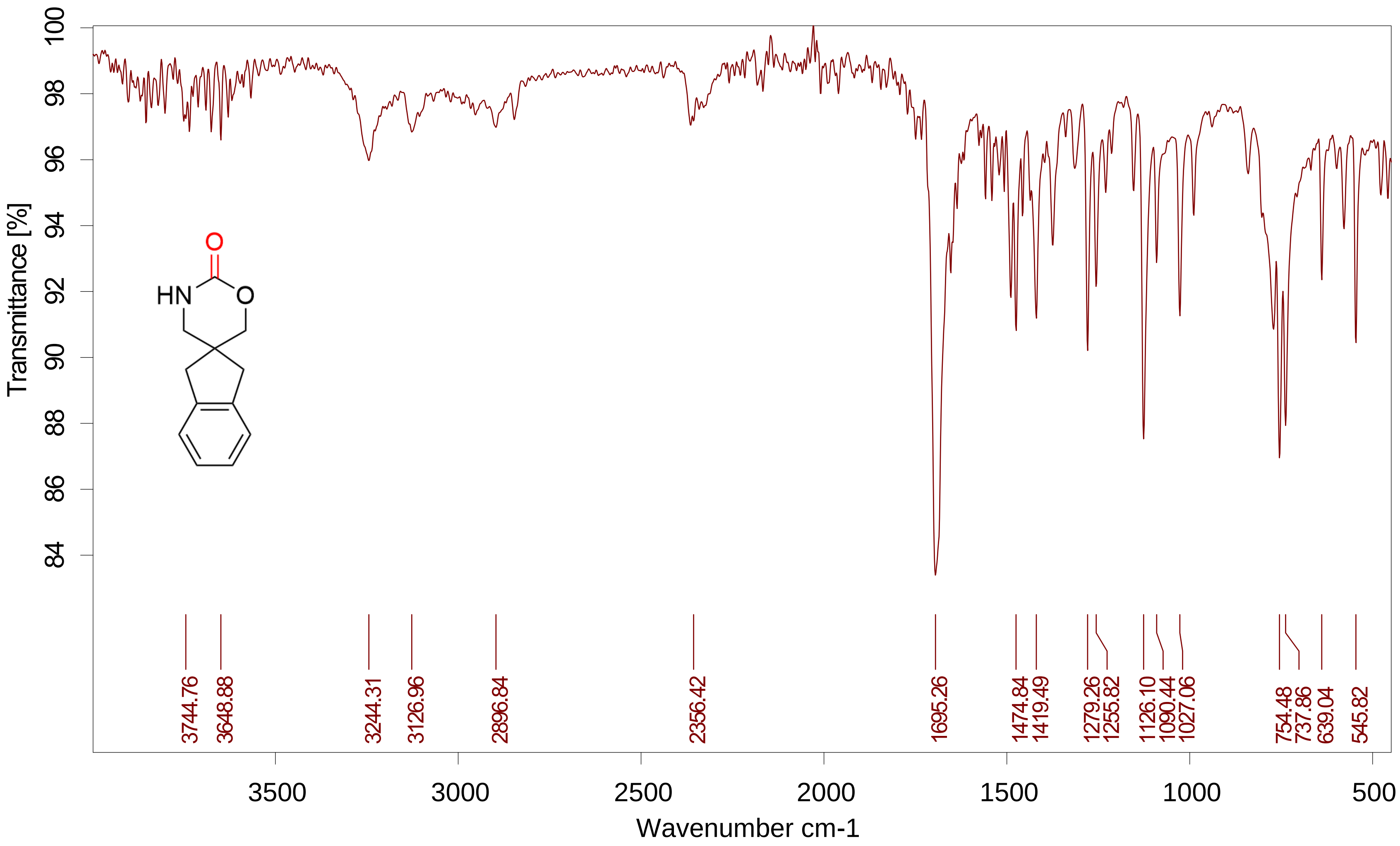

FTIR spectrum of compound **2l**.

Compound 2n

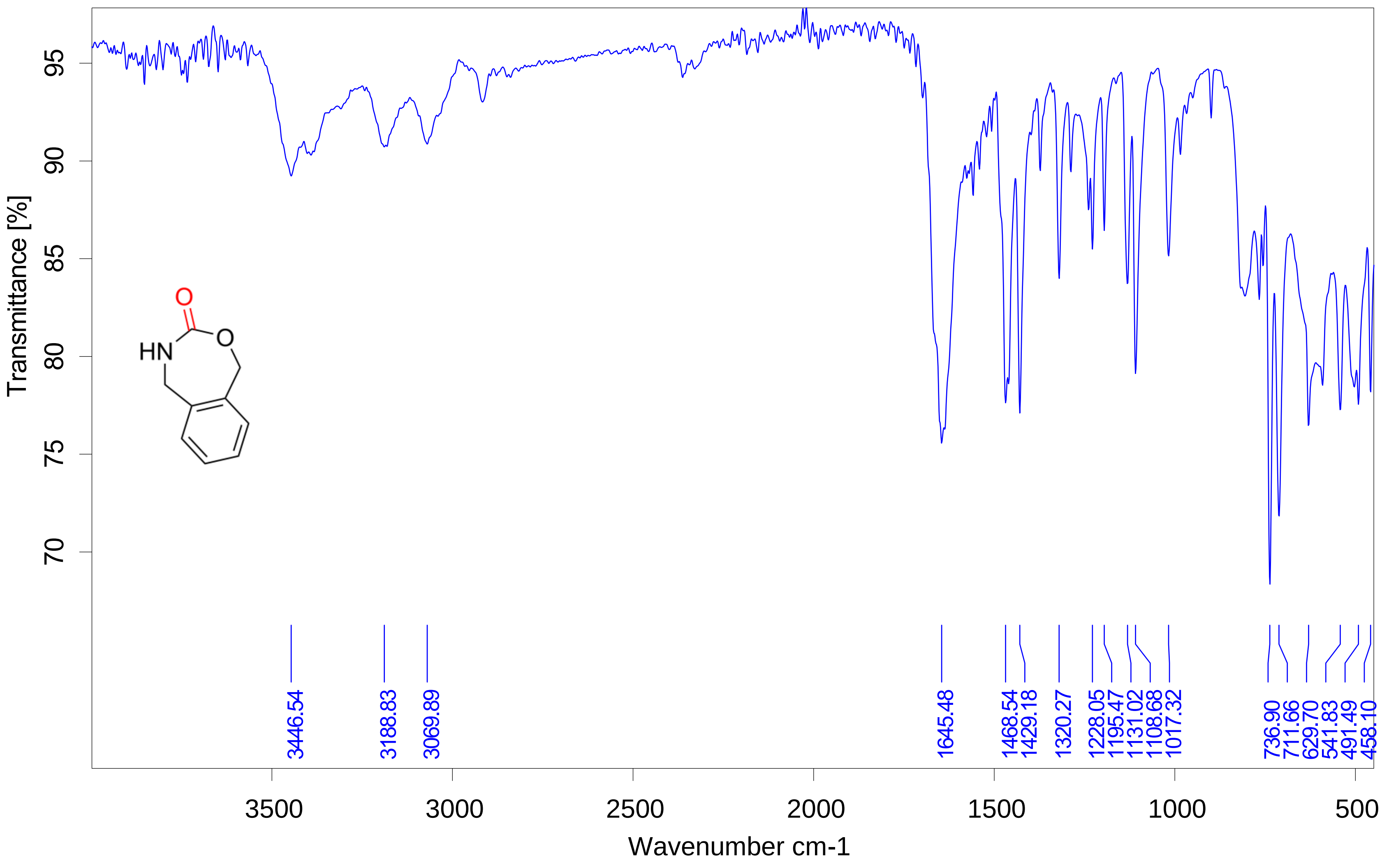

FTIR spectrum of compound **2n**.

Compound 2o

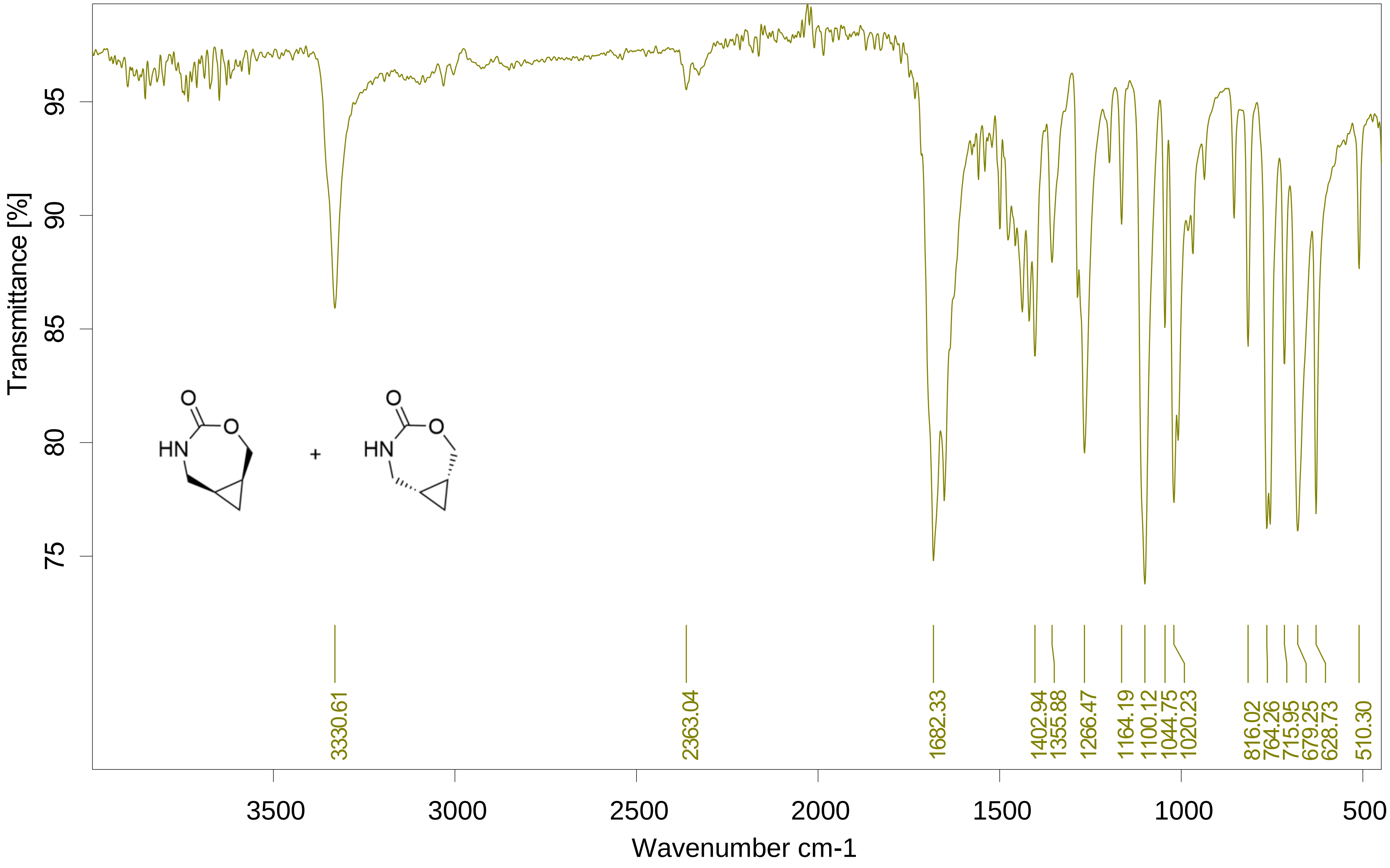

FTIR spectrum of compound **2o**.

9. NMR spectra of Isolated Amino Alcohols

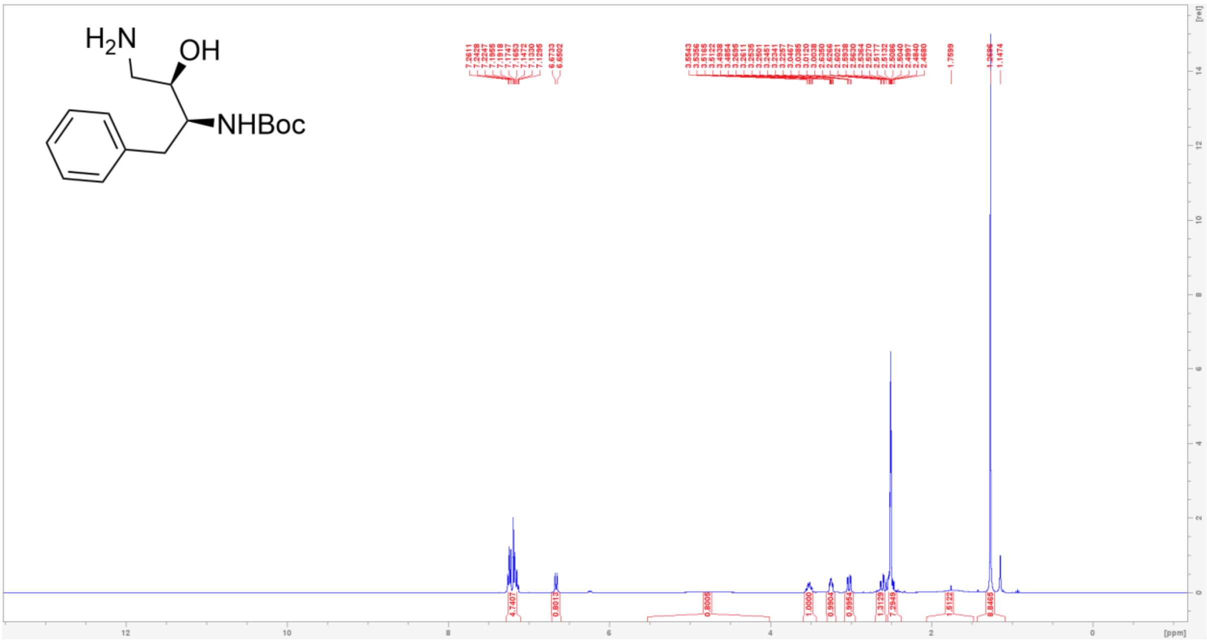

[1]H Spectrum of compound **1c** in DMSO-d$_6$ at 25°C. [1]H-frequency 400 MHz spectrometer.

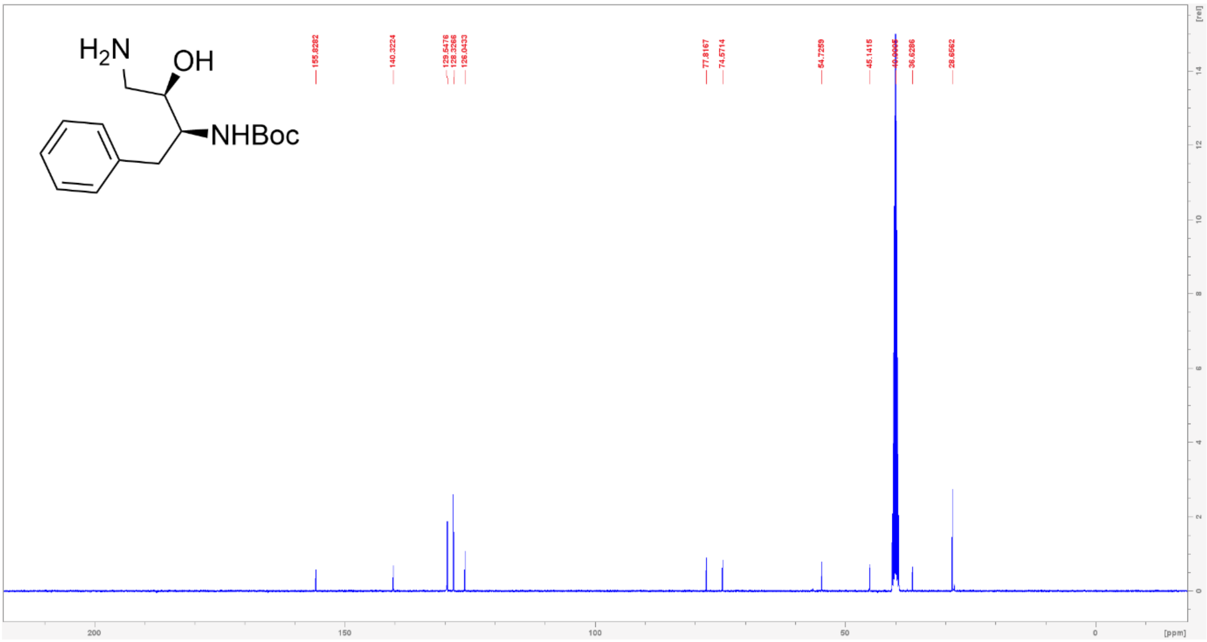

[13]C{[1]H} Spectrum of compound **1c** in DMSO-d$_6$ at 25°C. [1]H-frequency 400 MHz spectrometer.

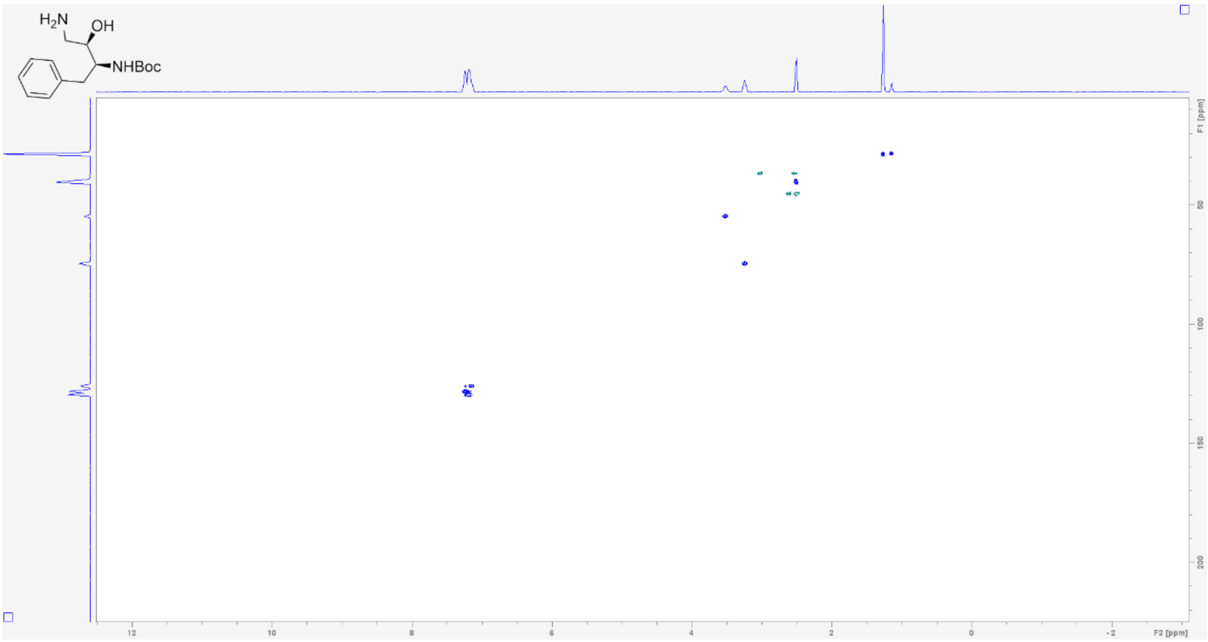

HSQC Spectrum of compound **1c** in DMSO-d_6 at 25°C. [1]H-frequency 400 MHz spectrometer.

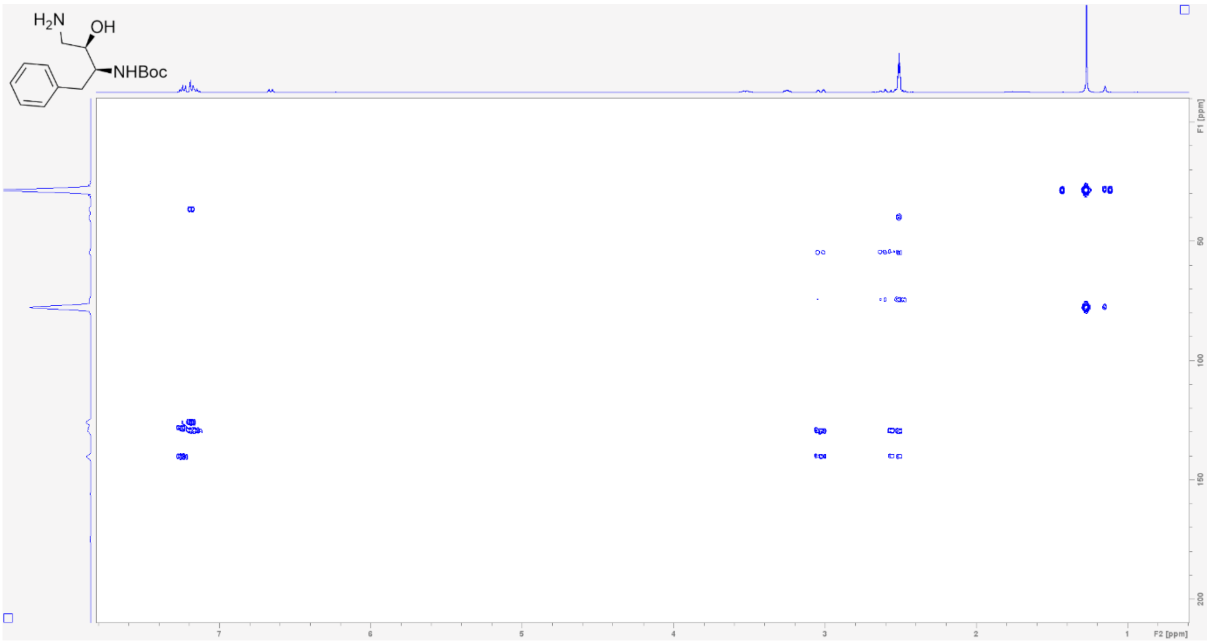

HMBC of compound **1c** in DMSO-d_6 at 25°C. [1]H-frequency 400 MHz spectrometer.

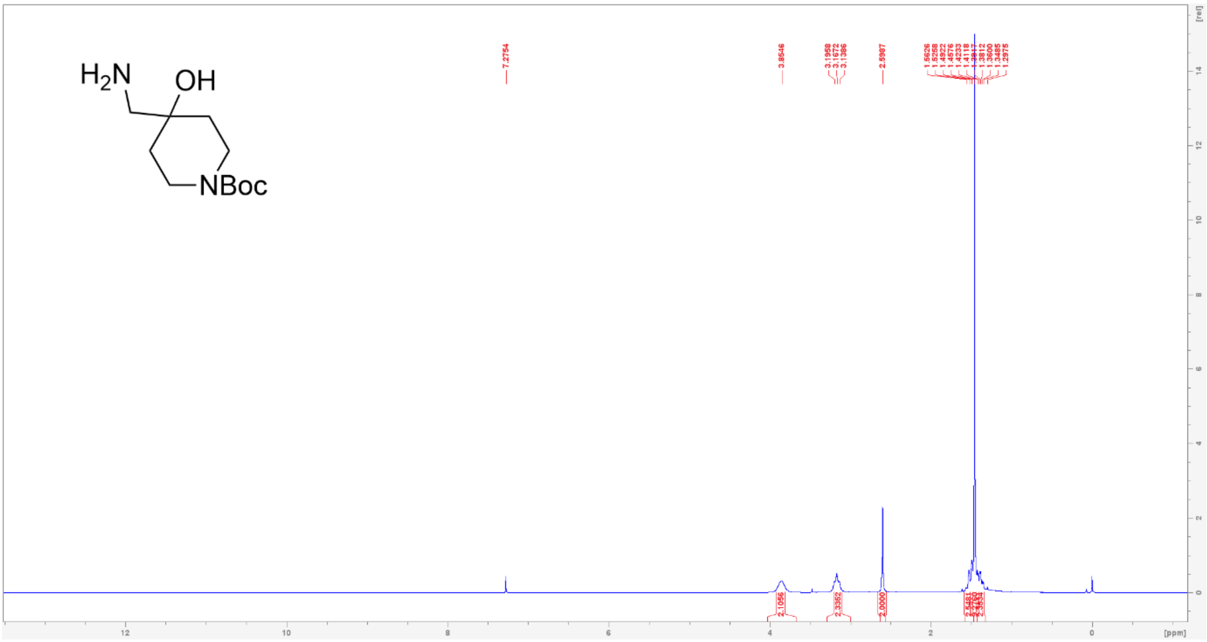

[1]H Spectrum of compound **1e** in $CDCl_3$ at 25°C. [1]H-frequency 400 MHz spectrometer.

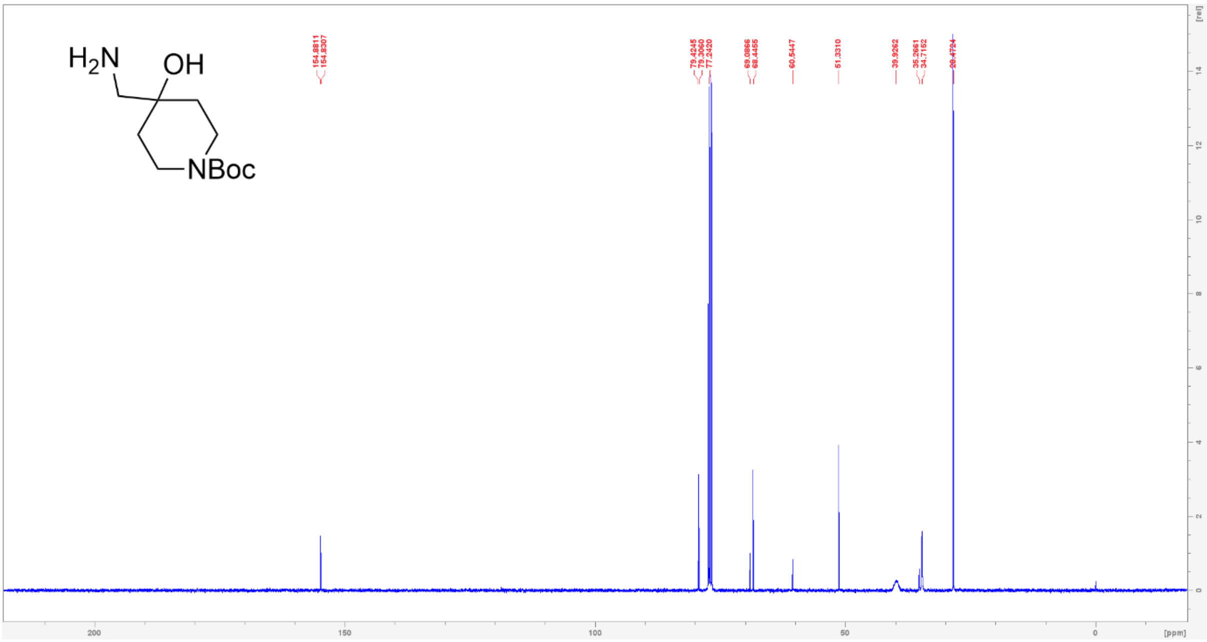

[13]C{[1]H} Spectrum of compound **1e** in $CDCl_3$ at 25°C. [1]H-frequency 400 MHz spectrometer.

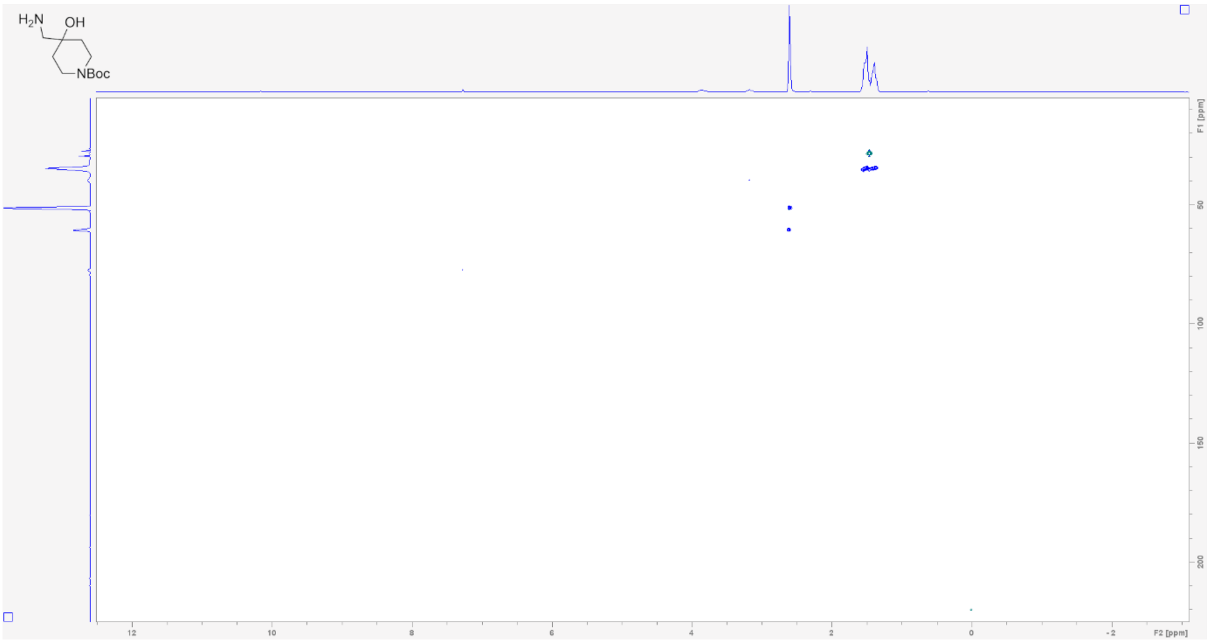

HSQC Spectrum of compound **1e** in $CDCl_3$ at 25°C. ^{1}H-frequency 400 MHz spectrometer.

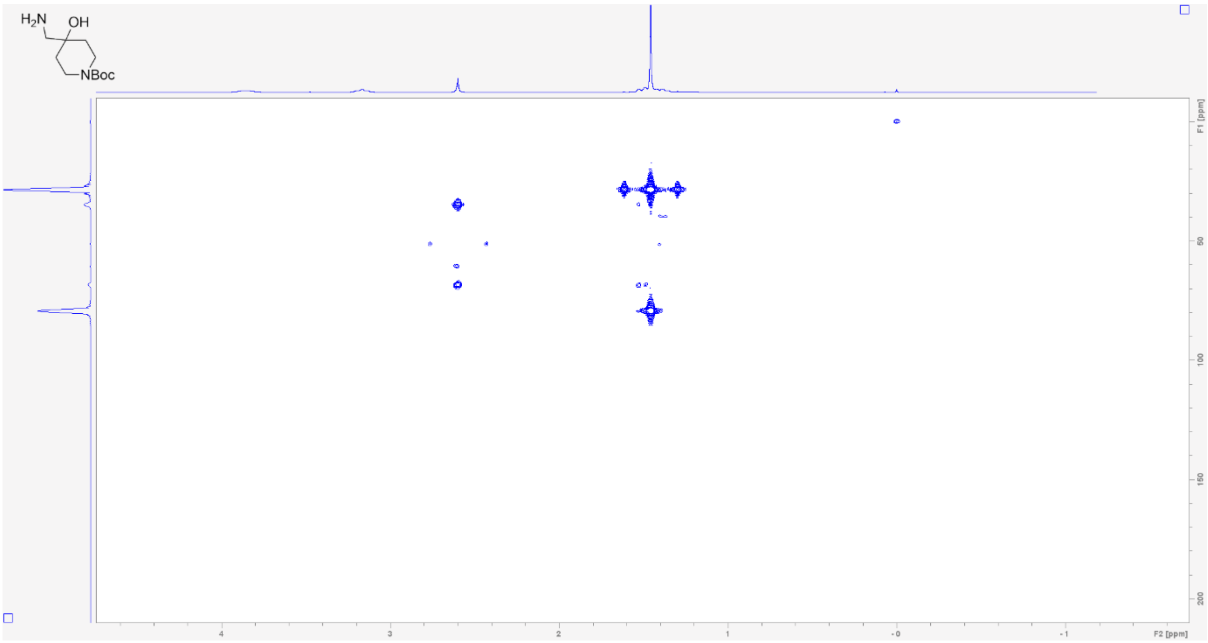

HMBC Spectrum of compound **1e** in $CDCl_3$ at 25°C. ^{1}H-frequency 400 MHz spectrometer.

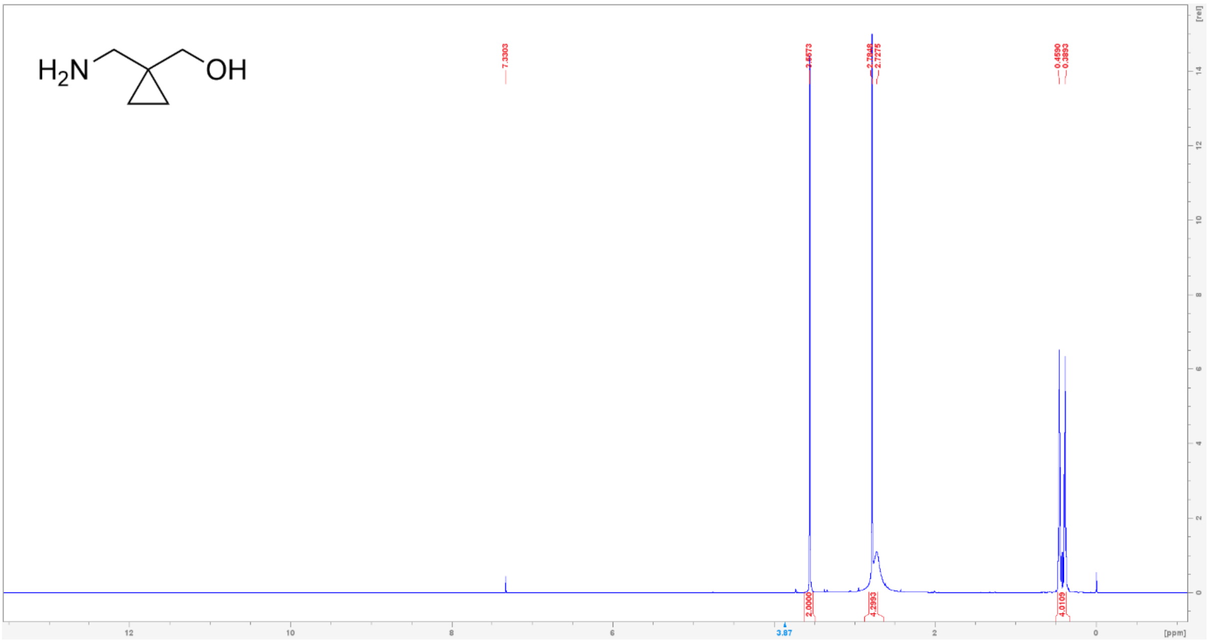

[1]H Spectrum of compound **1j** in $CDCl_3$ at 25°C. [1]H-frequency 400 MHz spectrometer.

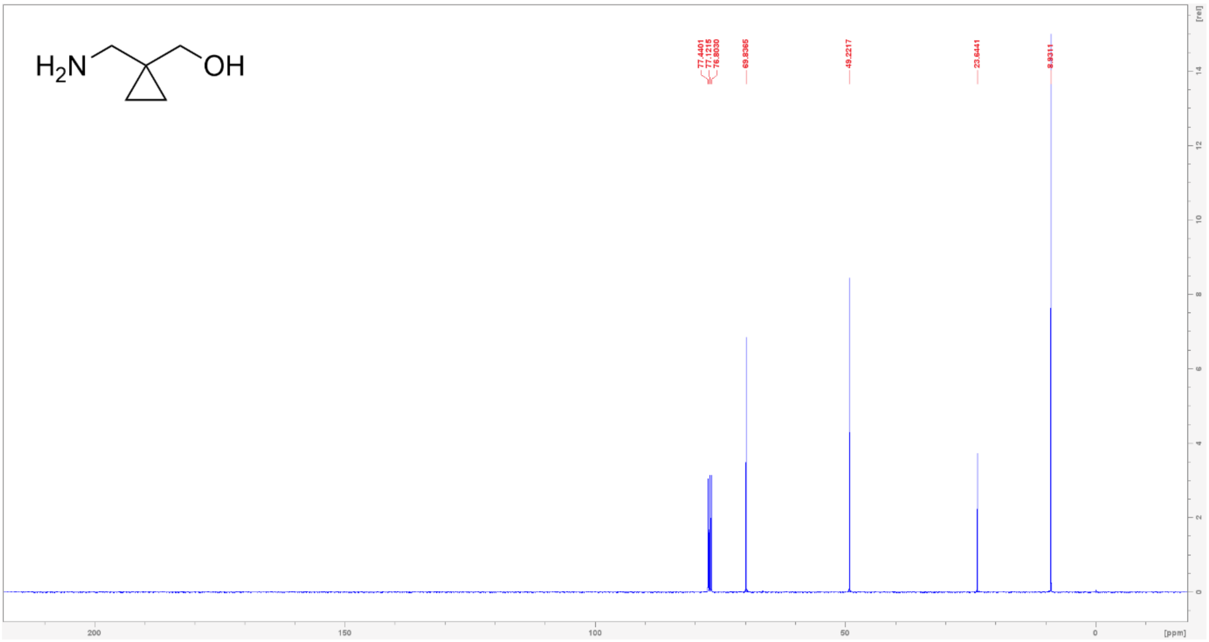

[13]C{[1]H} Spectrum of compound **1j** in $CDCl_3$ at 25°C. [1]H-frequency 400 MHz spectrometer.

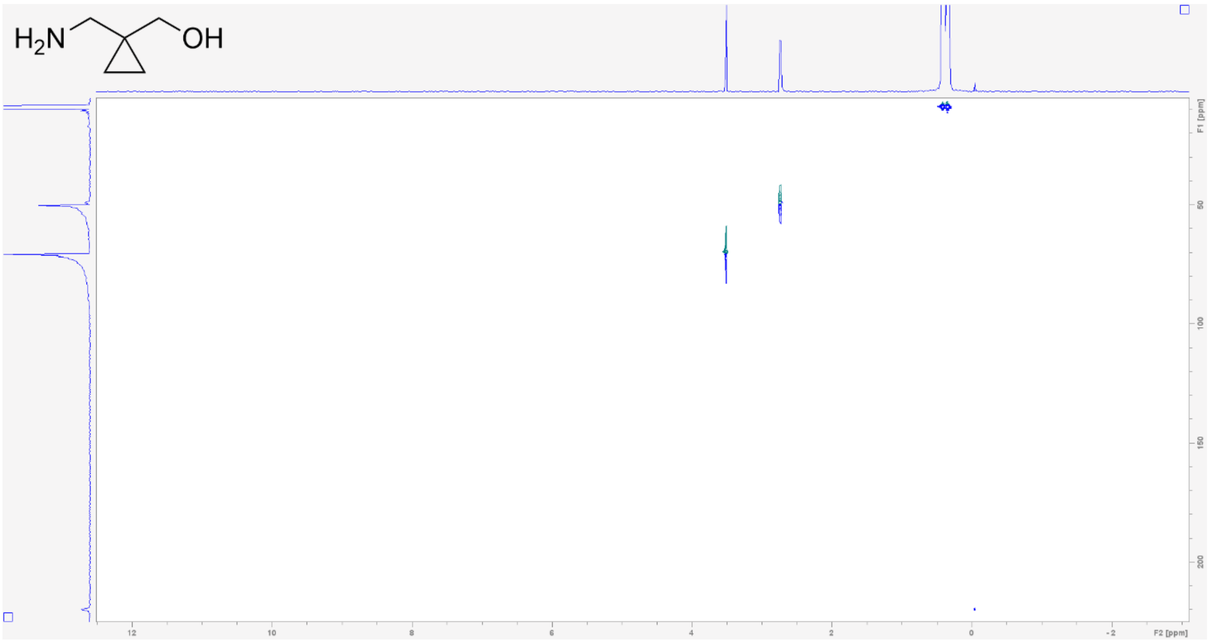

HSQC Spectrum of compound **1j** in $CDCl_3$ at 25°C. ^{1}H-frequency 400 MHz spectrometer.

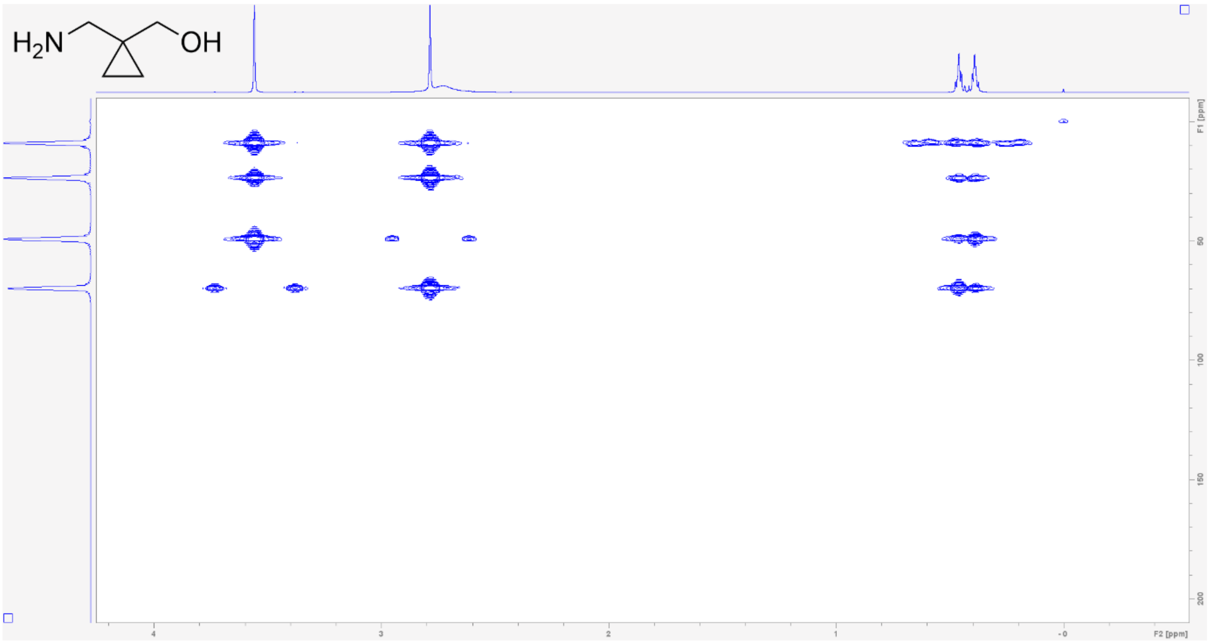

HMBC Spectrum of compound **1j** in $CDCl_3$ at 25°C. ^{1}H-frequency 400 MHz spectrometer.

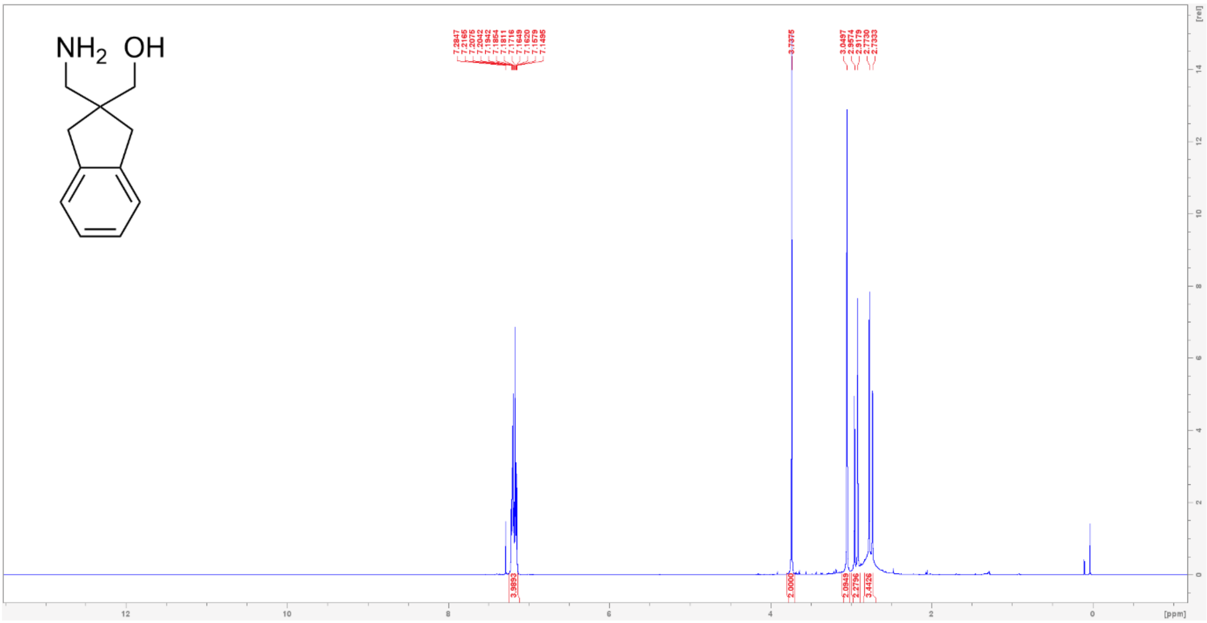

^{1}H Spectrum of compound **1l** in $CDCl_3$ at 25°C. ^{1}H-frequency 400 MHz spectrometer.

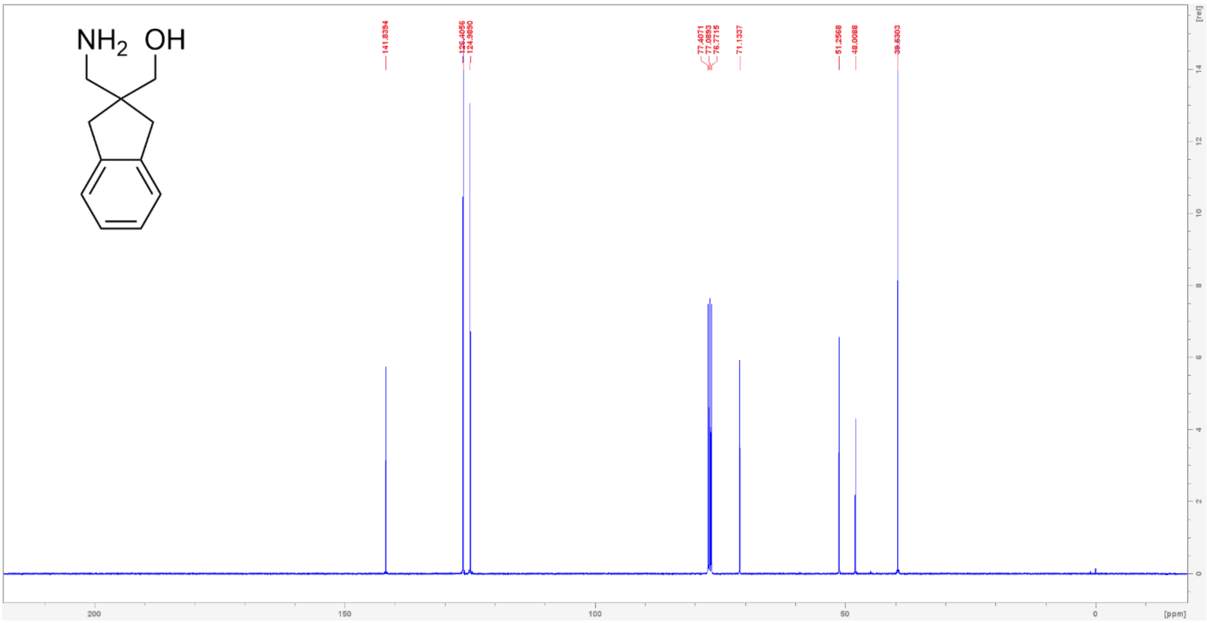

$^{13}C\{^{1}H\}$ Spectrum of compound **1l** in $CDCl_3$ at 25°C. ^{1}H-frequency 400 MHz spectrometer.

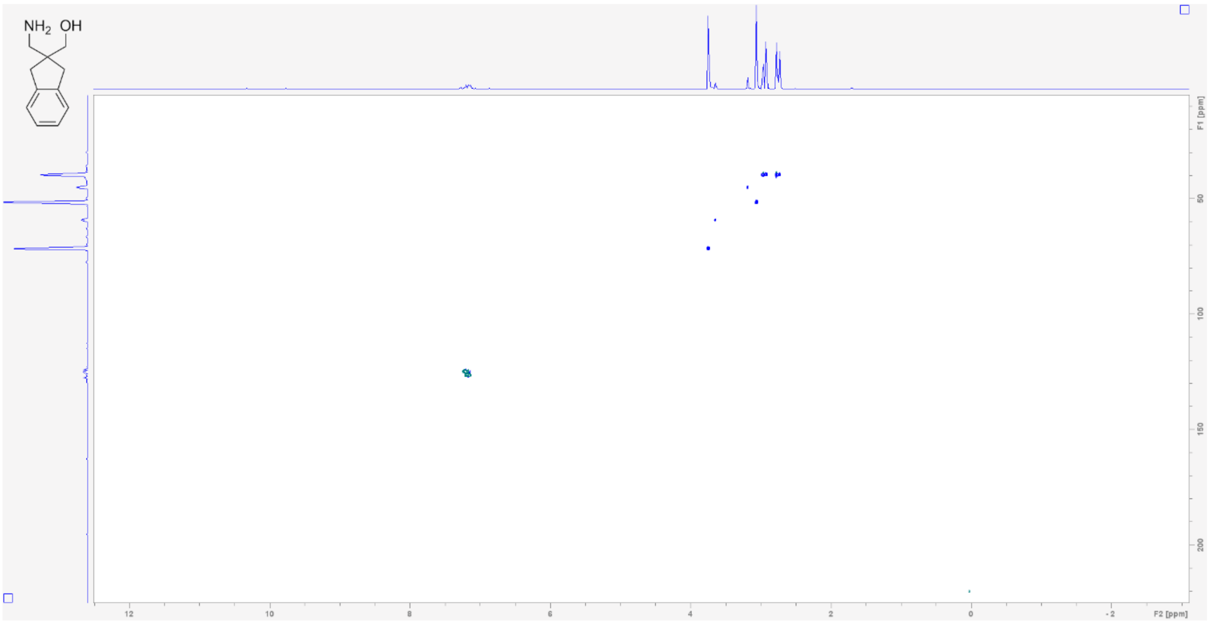

HSQC Spectrum of compound **1l** in $CDCl_3$ at 25°C. ^{1}H-frequency 400 MHz spectrometer.

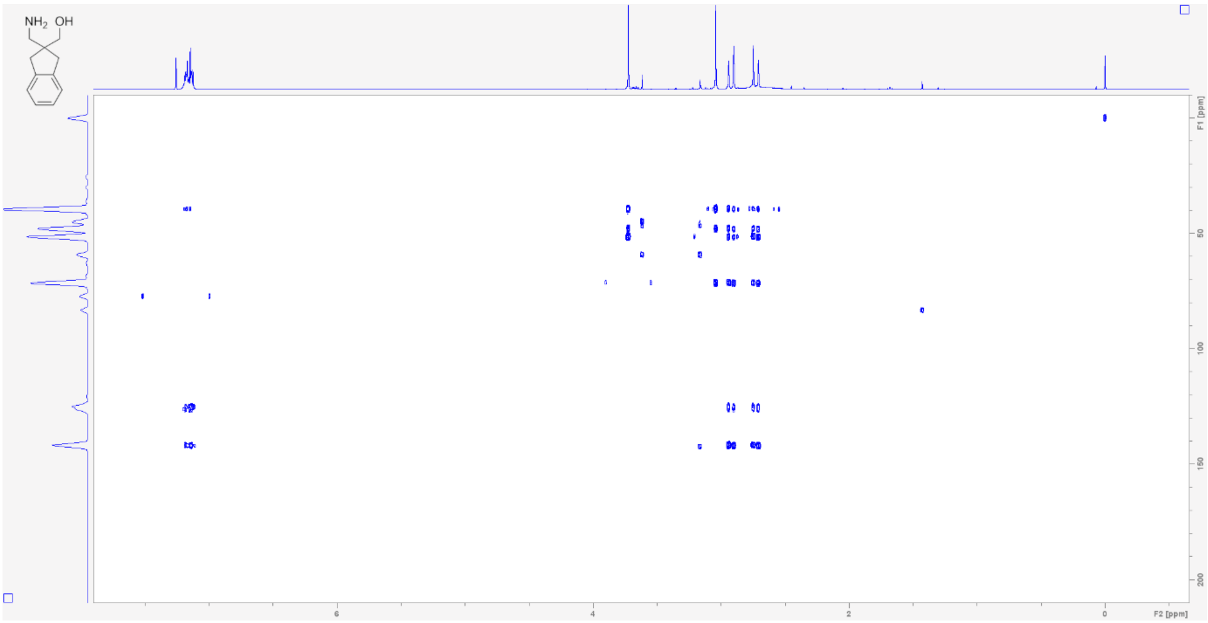

HMBC Spectrum of compound **1l** in $CDCl_3$ at 25°C. ^{1}H-frequency 400 MHz spectrometer.

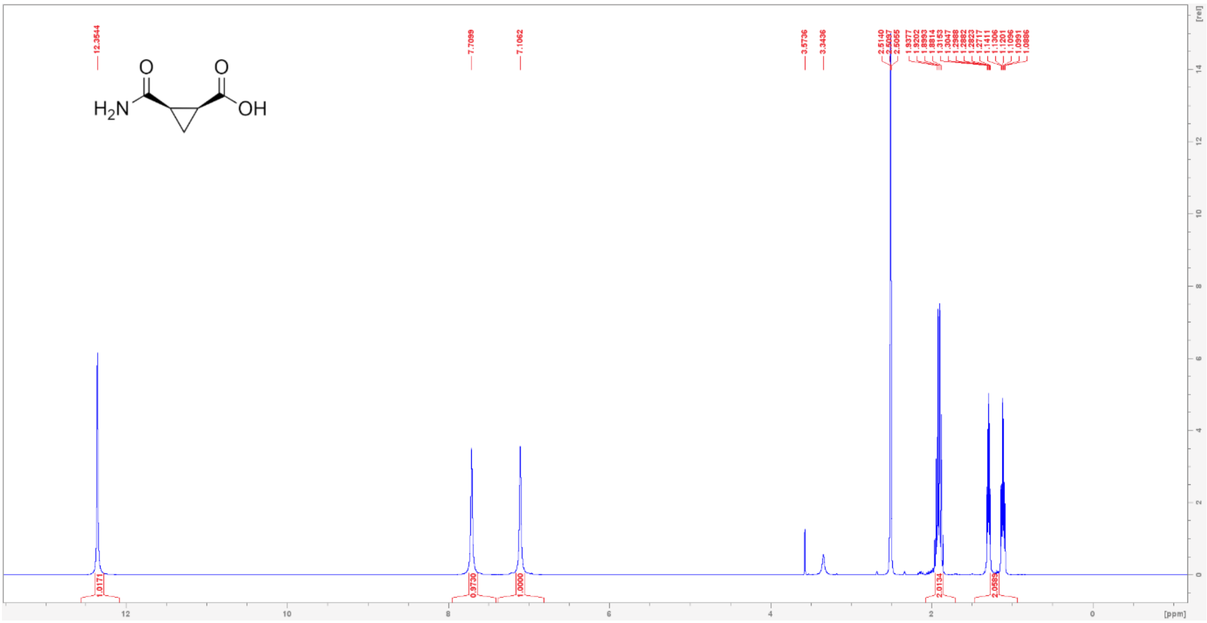

[1]H Spectrum of compound **S01o** in DMSO-d_6 at 25°C. [1]H-frequency 400 MHz spectrometer.

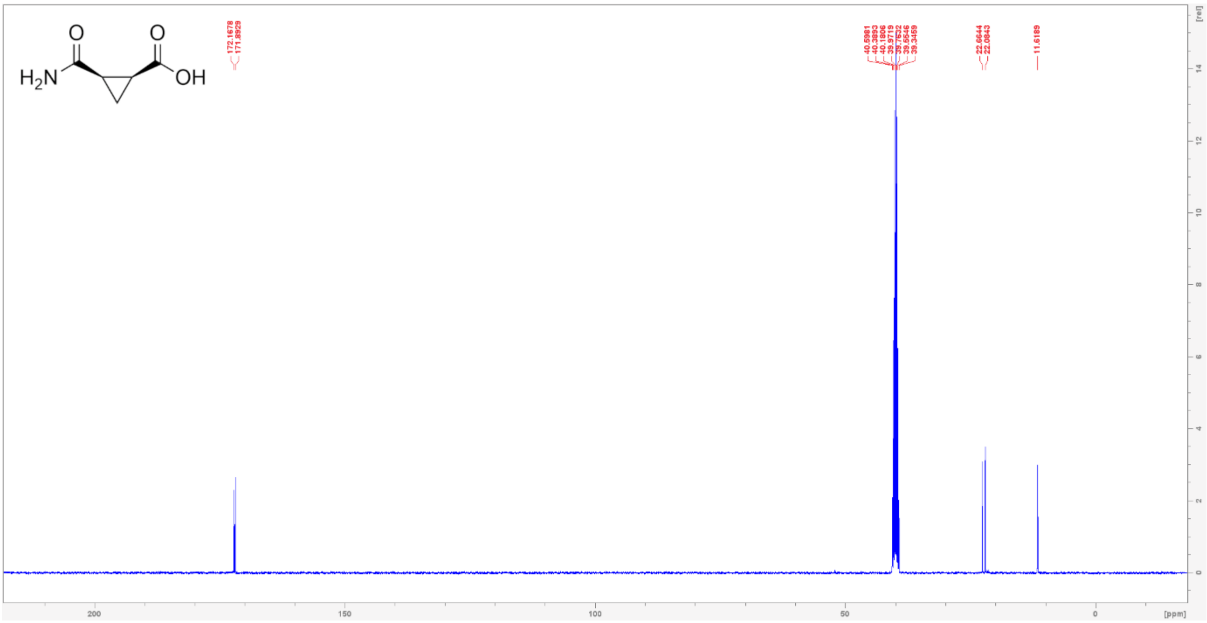

$^{13}C\{^1H\}$ Spectrum of compound **S01o** in DMSO-d_6 at 25°C. [1]H-frequency 400 MHz spectrometer.

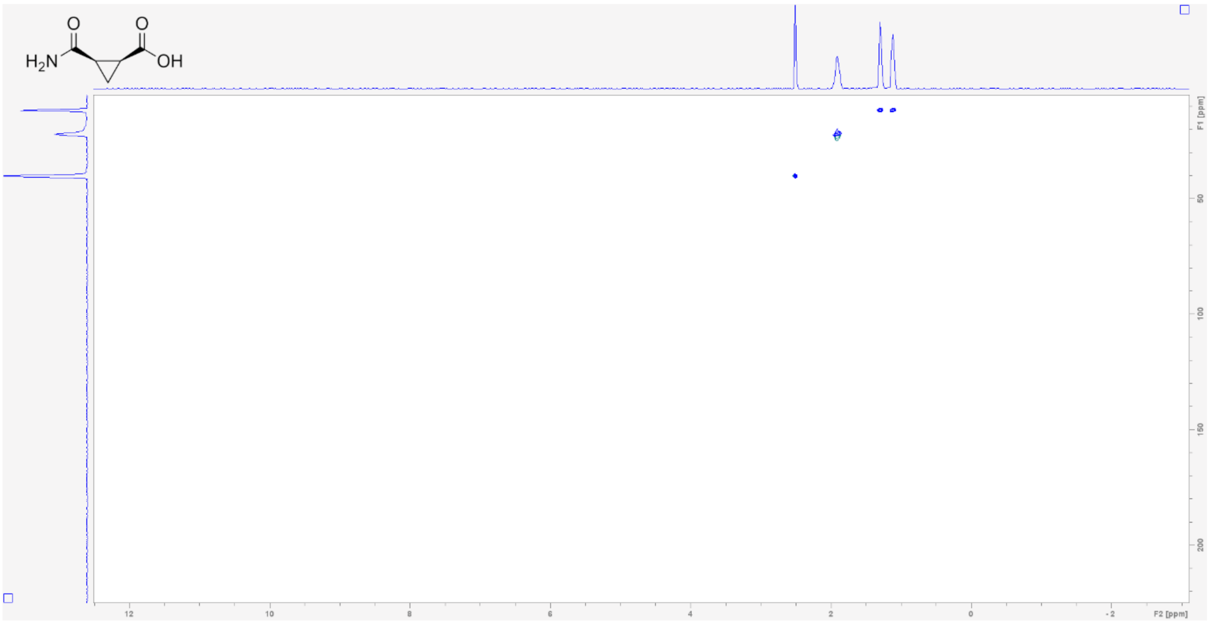

HSQC Spectrum of compound **S01o** in DMSO-d_6 at 25°C. [1]H-frequency 400 MHz spectrometer.

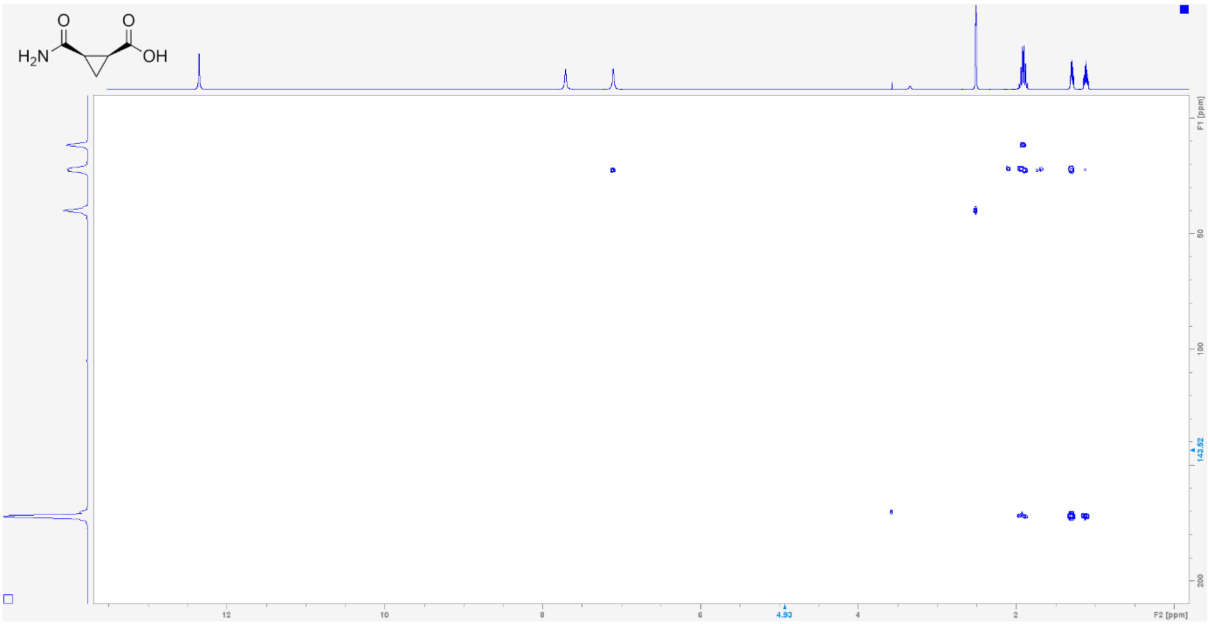

HMBC Spectrum of compound **S01o** in DMSO-d_6 at 25°C. [1]H-frequency 400 MHz spectrometer.

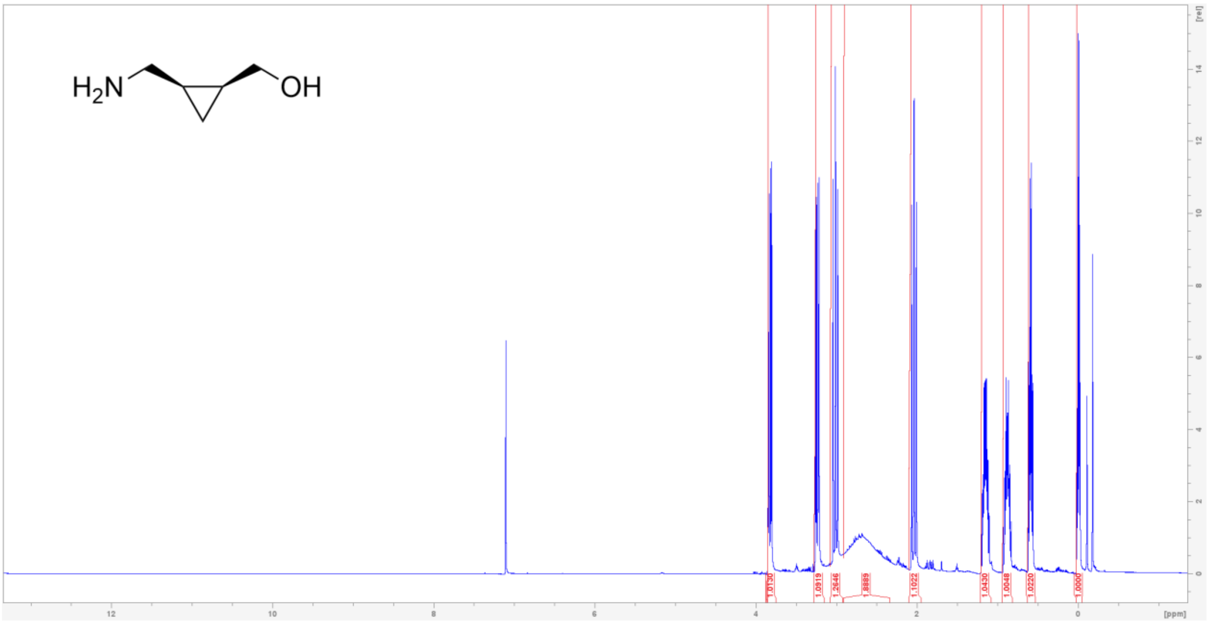

[1]H Spectrum of compound **1o** in $CDCl_3$ at 25°C. [1]H-frequency 400 MHz spectrometer.

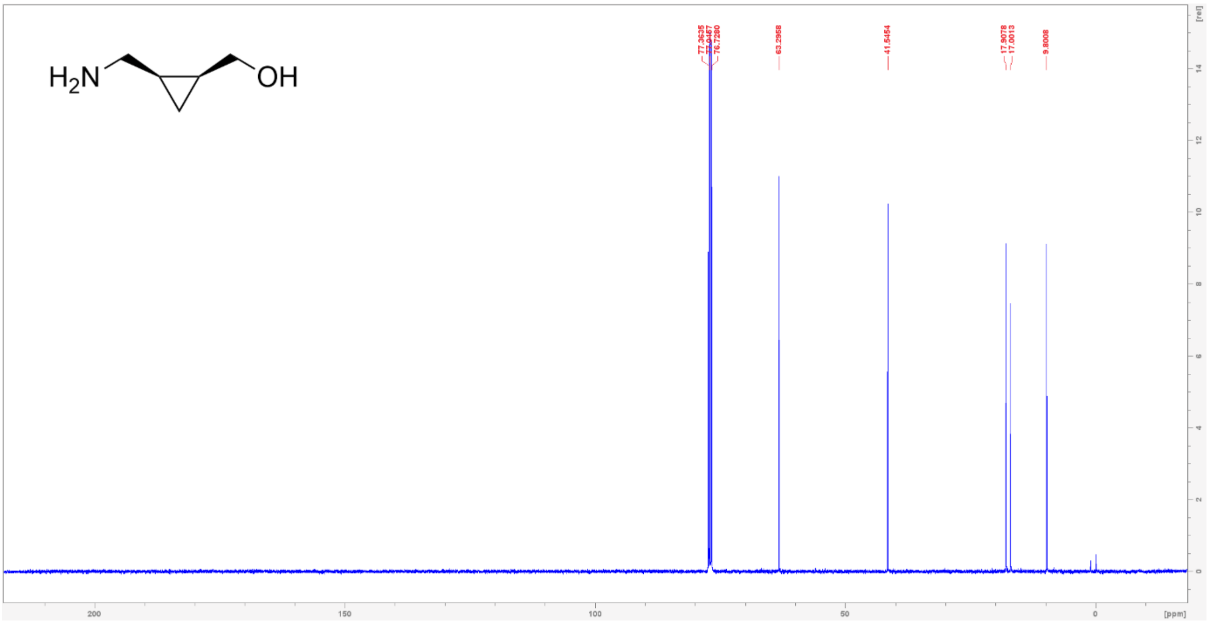

[13]C{[1]H} Spectrum of compound **1o** in $CDCl_3$ at 25°C. [1]H-frequency 400 MHz spectrometer.

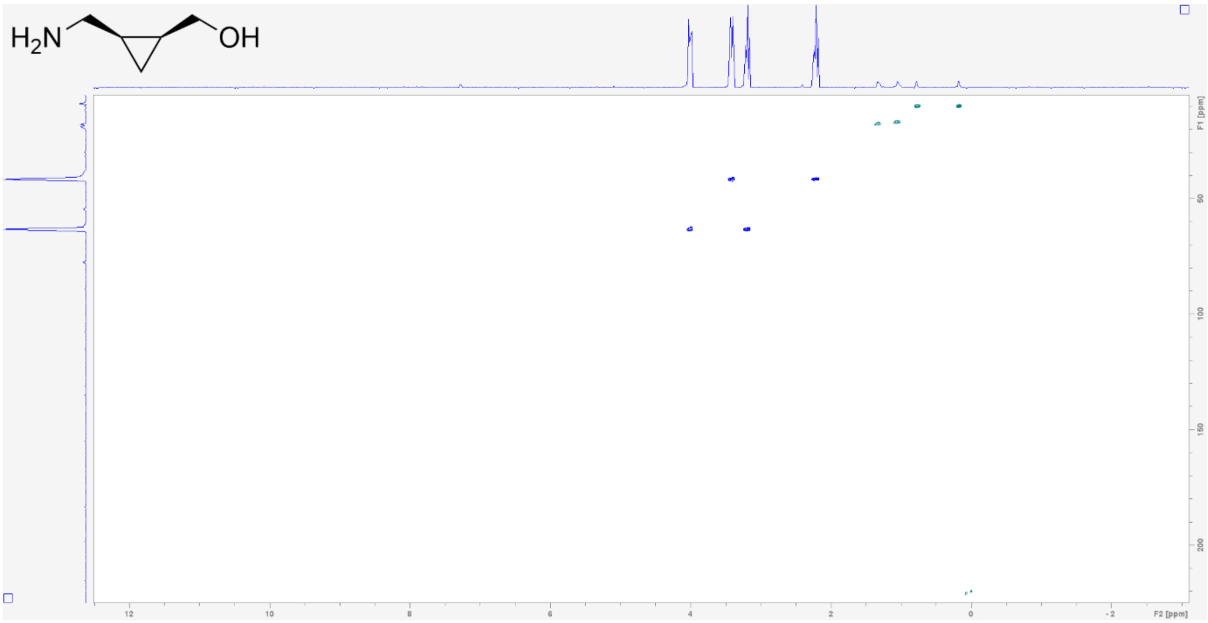

HSQC Spectrum of compound **1o** in $CDCl_3$ at 25°C. ^{1}H-frequency 400 MHz spectrometer.

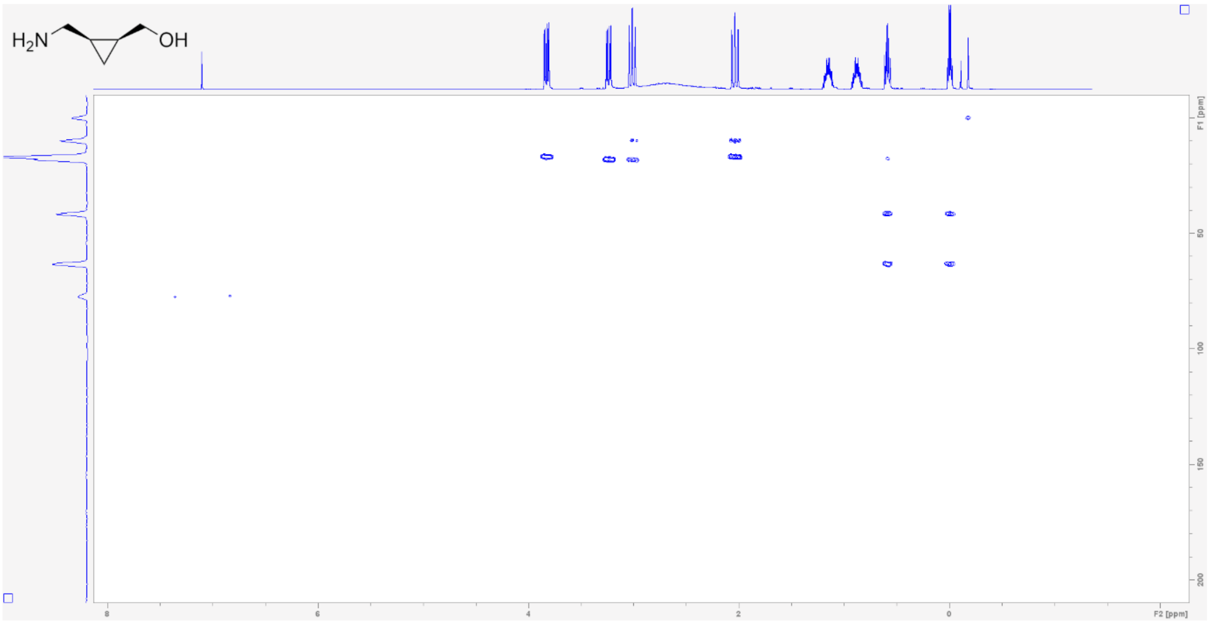

HMBC Spectrum of compound **1o** in $CDCl_3$ at 25°C. ^{1}H-frequency 400 MHz spectrometer.

10. NMR Spectra of Isolated Carbamates

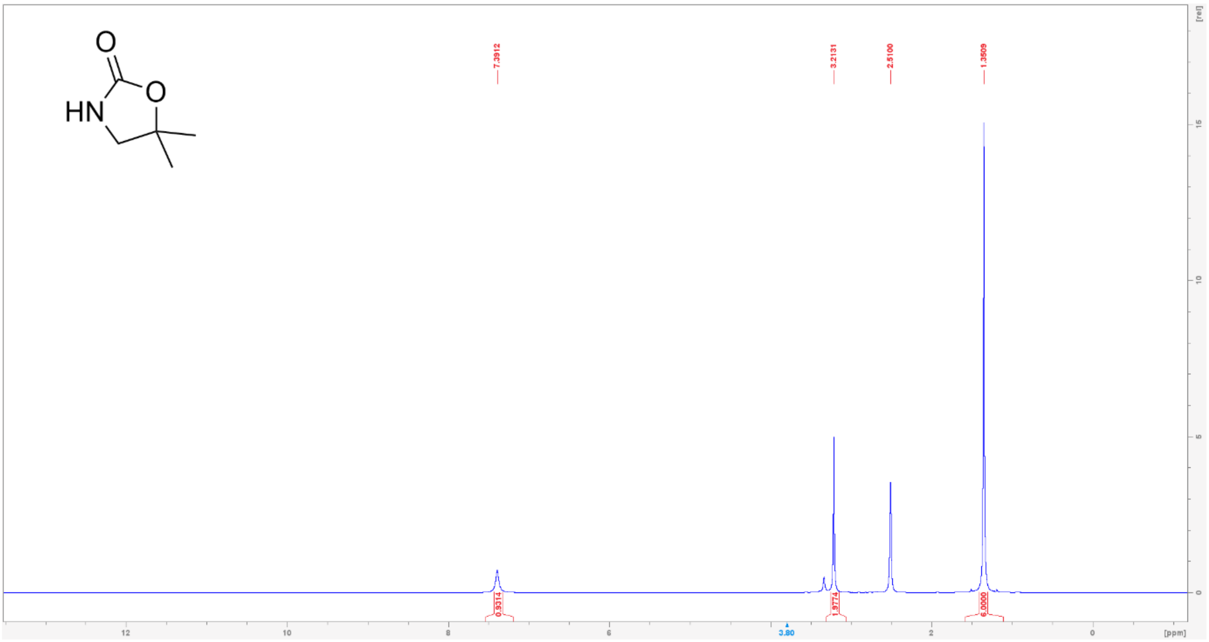

[1]H Spectrum of compound **2a** in DMSO-d_6 at 25°C. [1]H-frequency 400 MHz spectrometer.

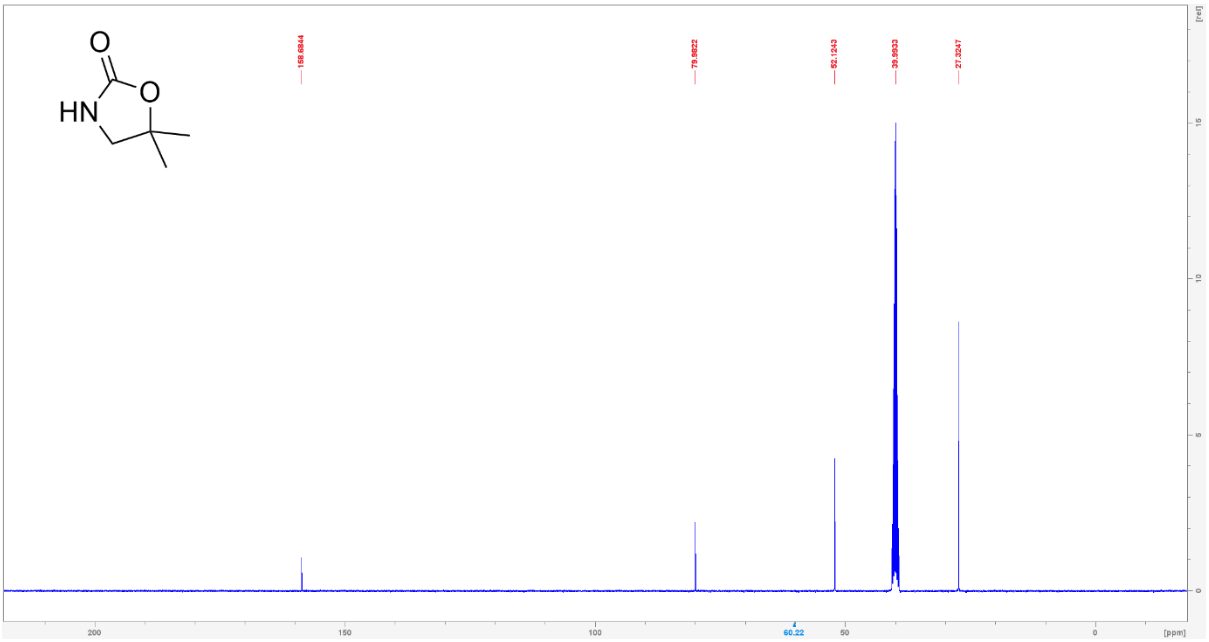

[13]C{[1]H} Spectrum of compound **2a** in DMSO-d_6 at 25°C. [1]H-frequency 400 MHz spectrometer.

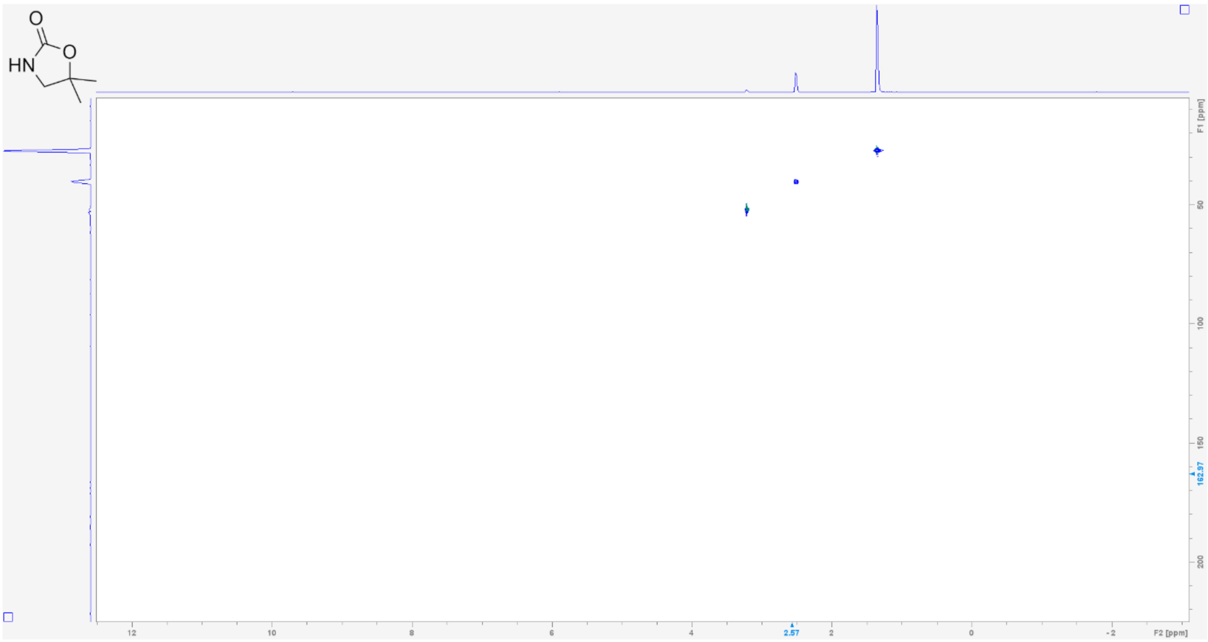

HSQC Spectrum of compound **2a** in DMSO-d_6 at 25°C. ^{1}H-frequency 400 MHz spectrometer.

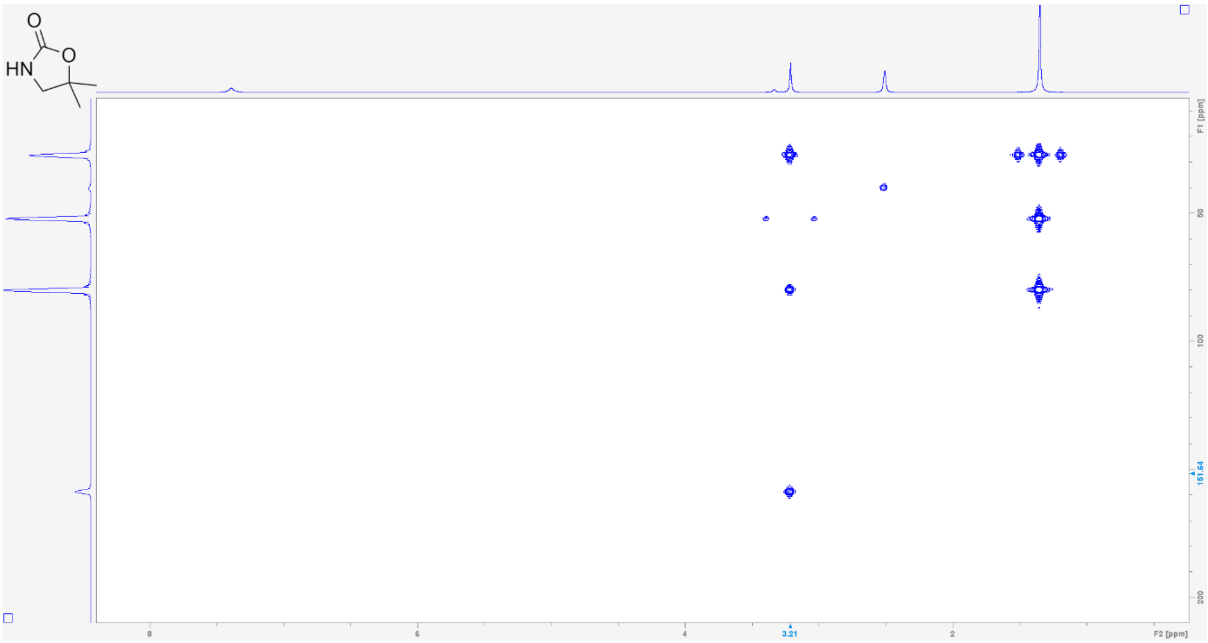

HMBC Spectrum of compound **2a** in DMSO-d_6 at 25°C. ^{1}H-frequency 400 MHz spectrometer.

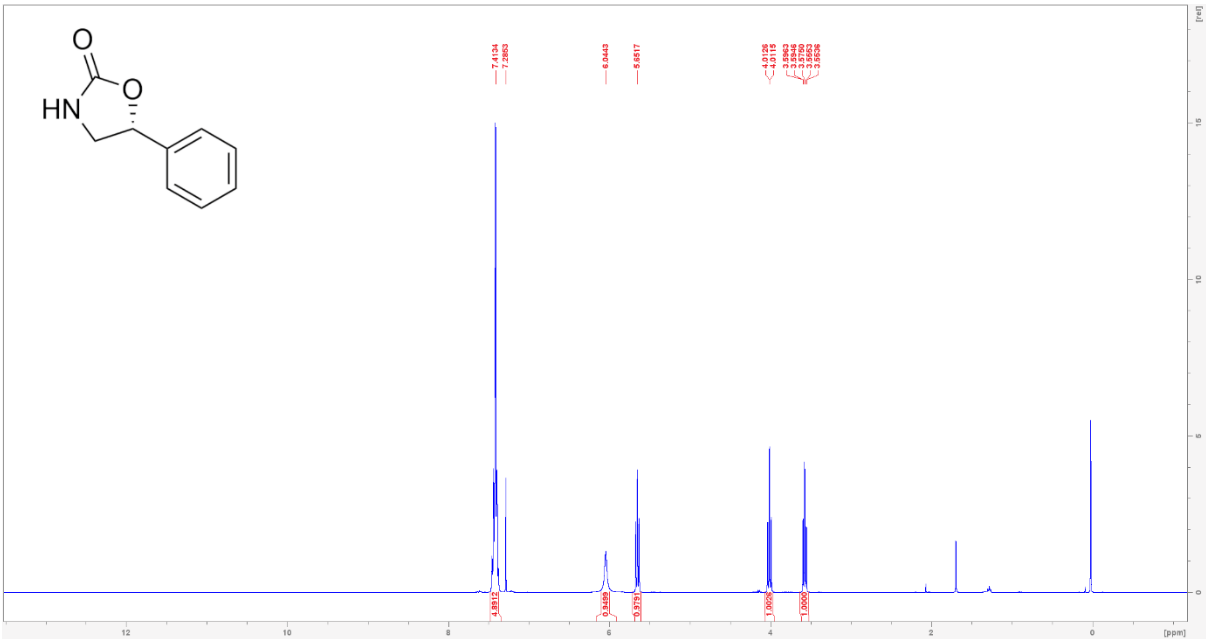

[1]H Spectrum of compound **2b** in $CDCl_3$ at 25°C. [1]H-frequency 400 MHz spectrometer.

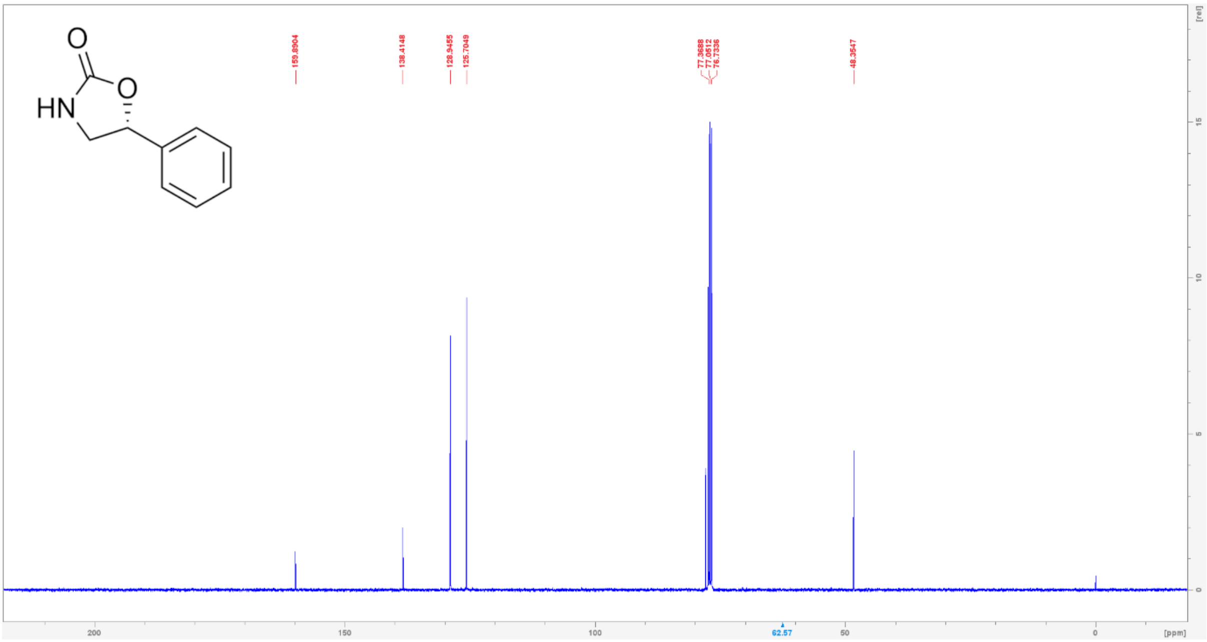

$^{13}C\{^{1}H\}$ Spectrum of compound **2b** in $CDCl_3$ at 25°C. [1]H-frequency 400 MHz spectrometer.

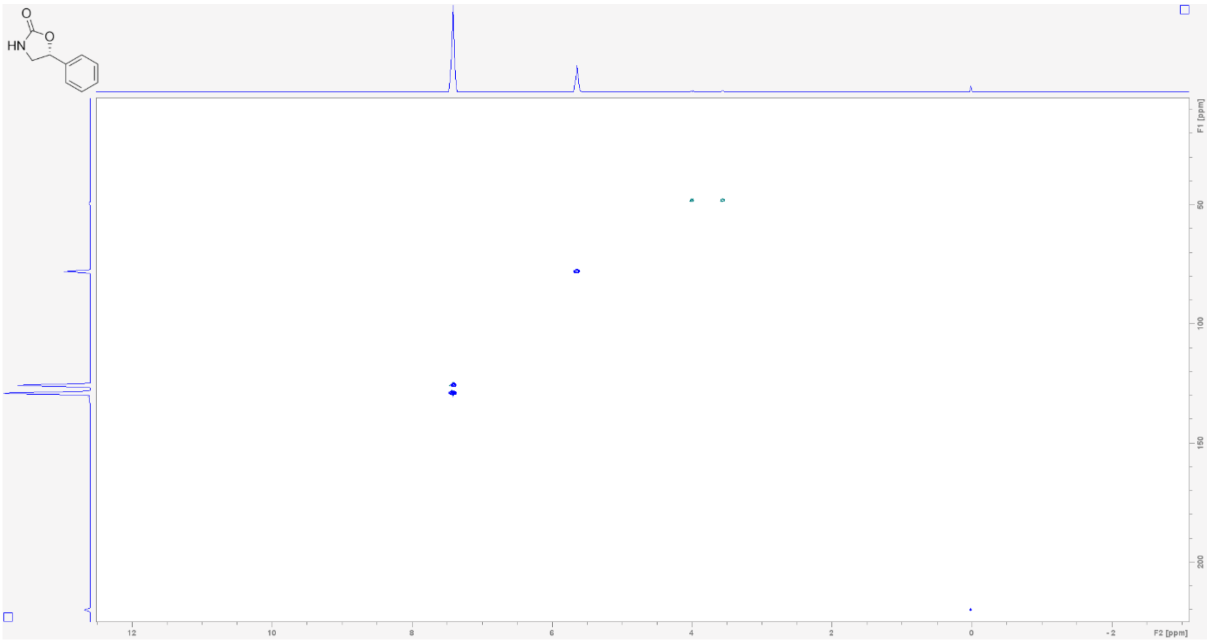

HSQC Spectrum of compound **2b** in $CDCl_3$ at 25°C. [1]H-frequency 400 MHz spectrometer.

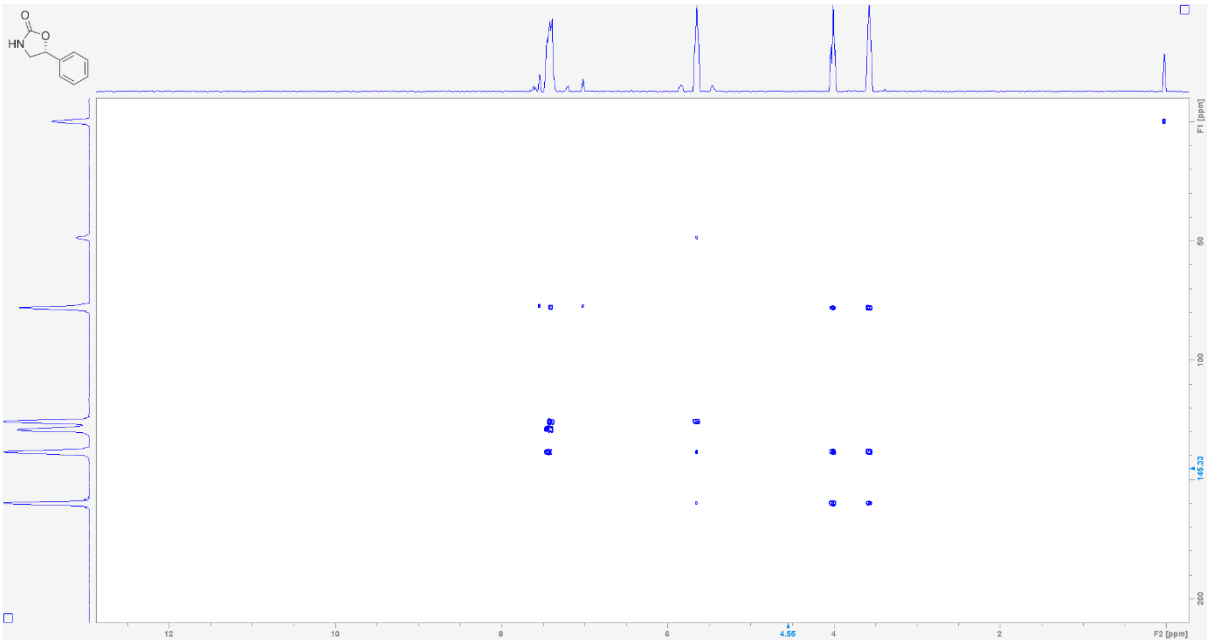

HMBC Spectrum of compound **2b** in $CDCl_3$ at 25°C. [1]H-frequency 400 MHz spectrometer.

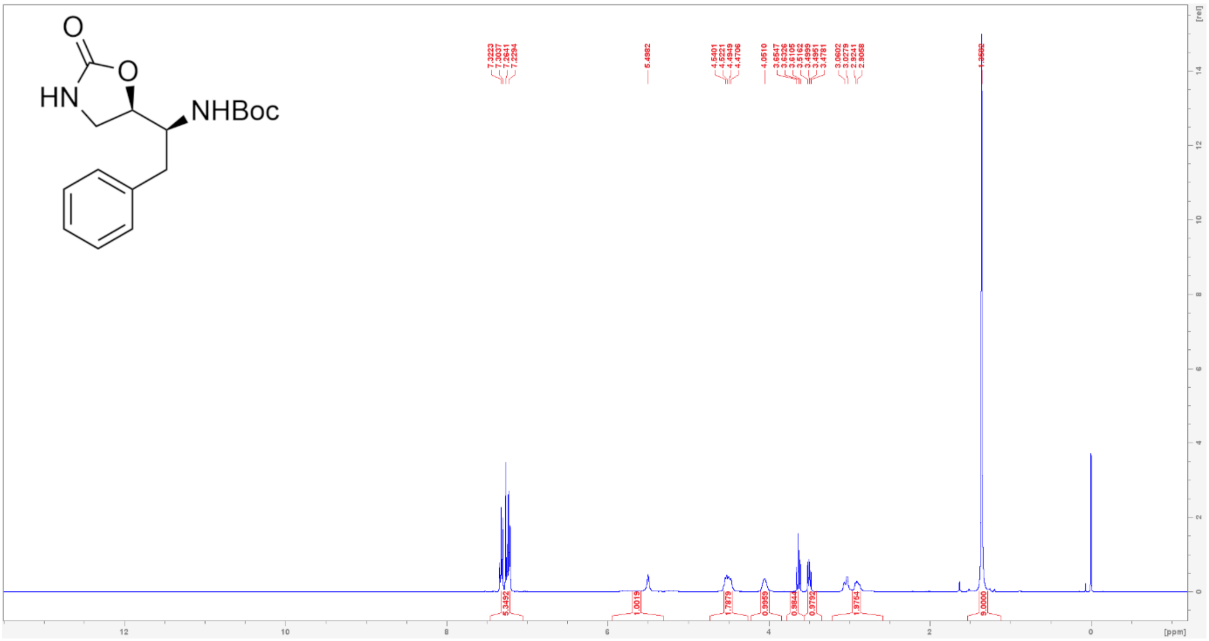

[1]H Spectrum of compound **2c** in $CDCl_3$ at 25°C. [1]H-frequency 400 MHz spectrometer.

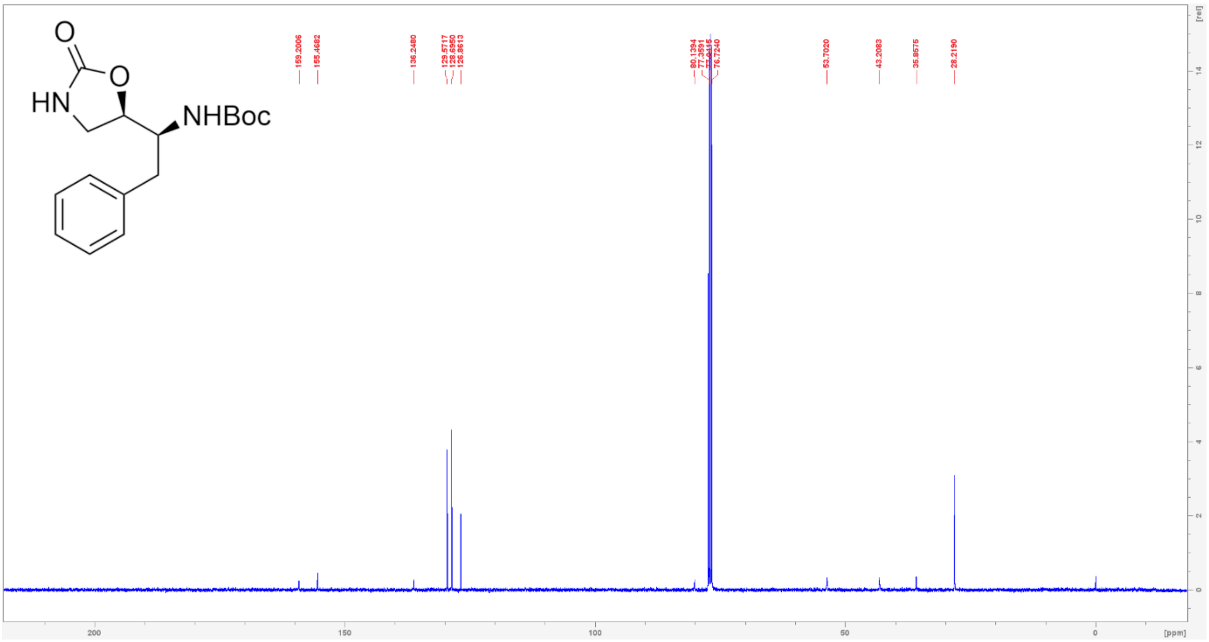

[13]C{[1]H} Spectrum of compound **2c** in $CDCl_3$ at 25°C. [1]H-frequency 400 MHz spectrometer.

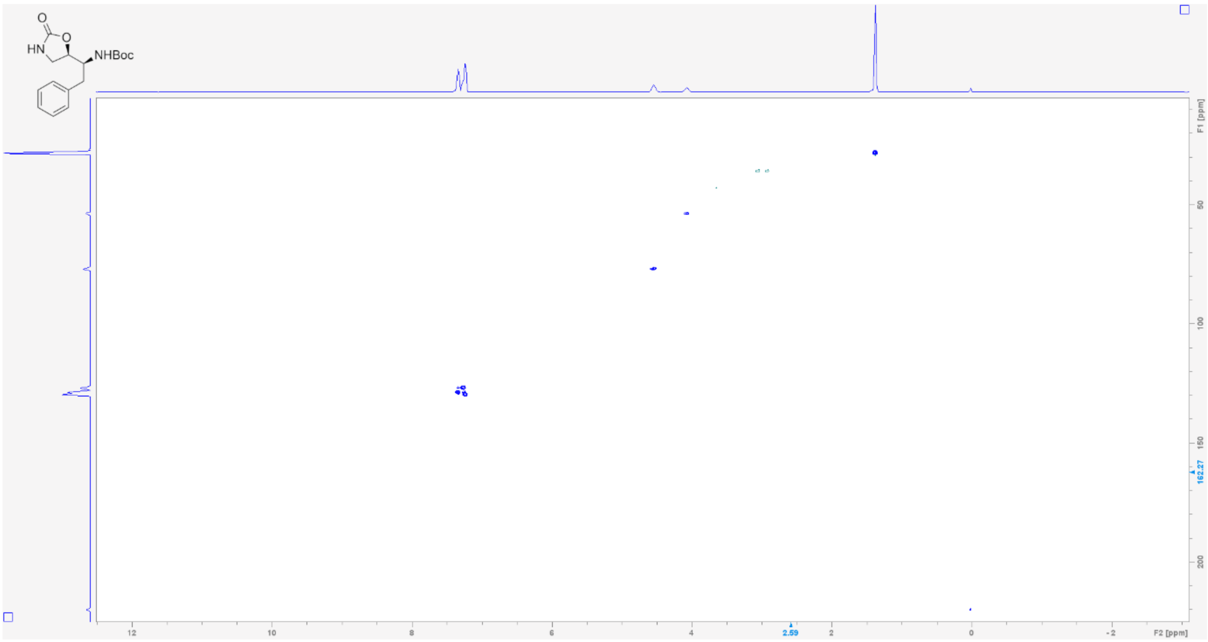

HSQC Spectrum of compound **2c** in $CDCl_3$ at 25°C. [1]H-frequency 400 MHz spectrometer.

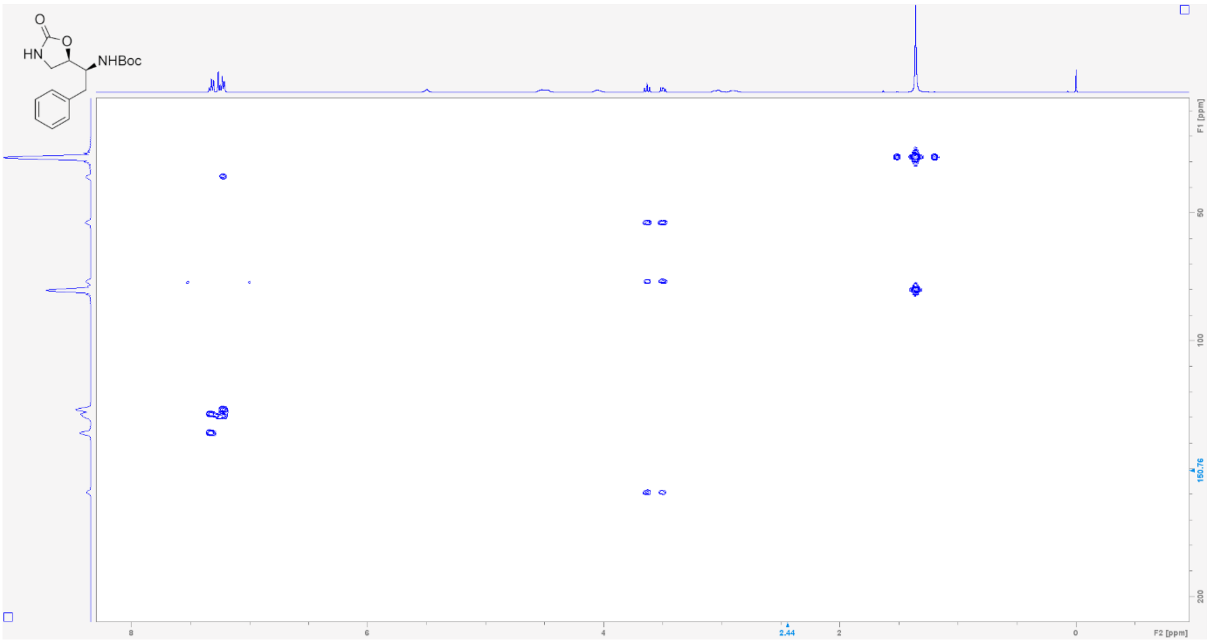

HMBC Spectrum of compound **2c** in $CDCl_3$ at 25°C. [1]H-frequency 400 MHz spectrometer.

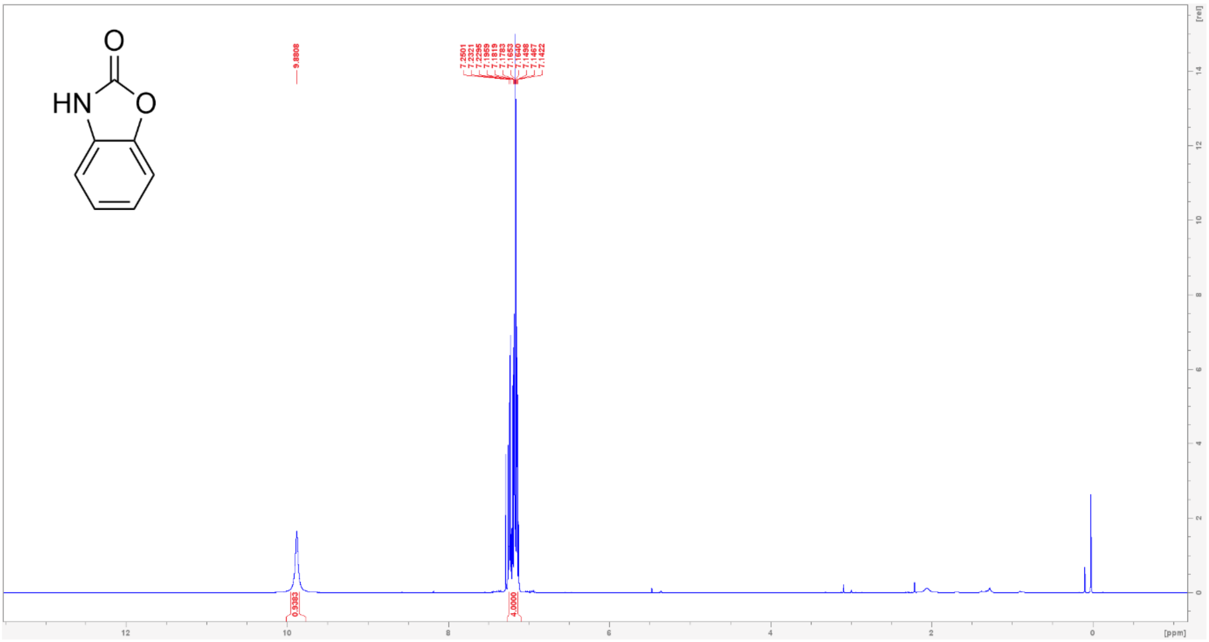

^{1}H Spectrum of compound **2d** in $CDCl_3$ at 25°C. ^{1}H-frequency 400 MHz spectrometer.

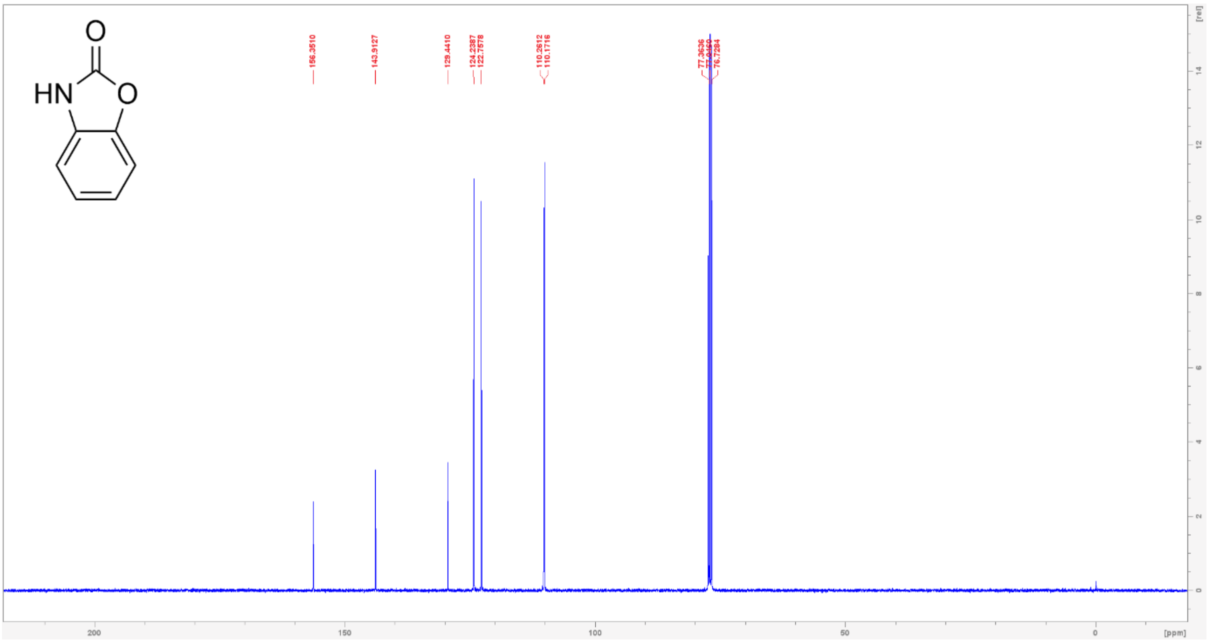

^{13}C{^{1}H} Spectrum of compound **2d** in $CDCl_3$ at 25°C. ^{1}H-frequency 400 MHz spectrometer.

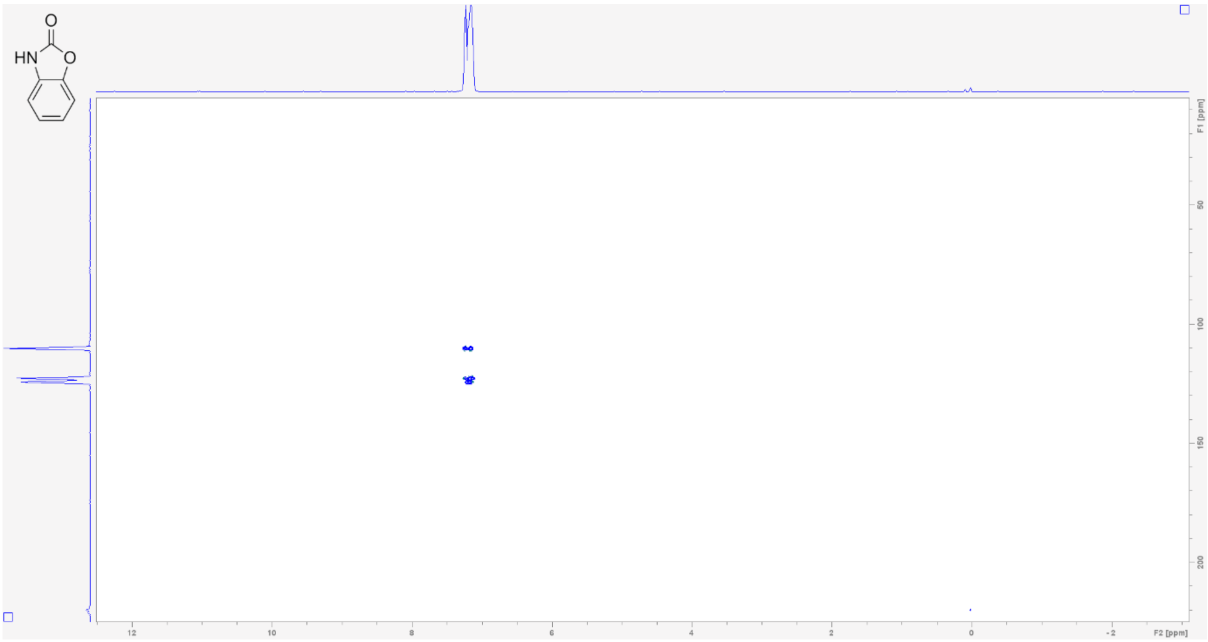

HSQC Spectrum of compound **2d** in $CDCl_3$ at 25°C. ^{1}H-frequency 400 MHz spectrometer.

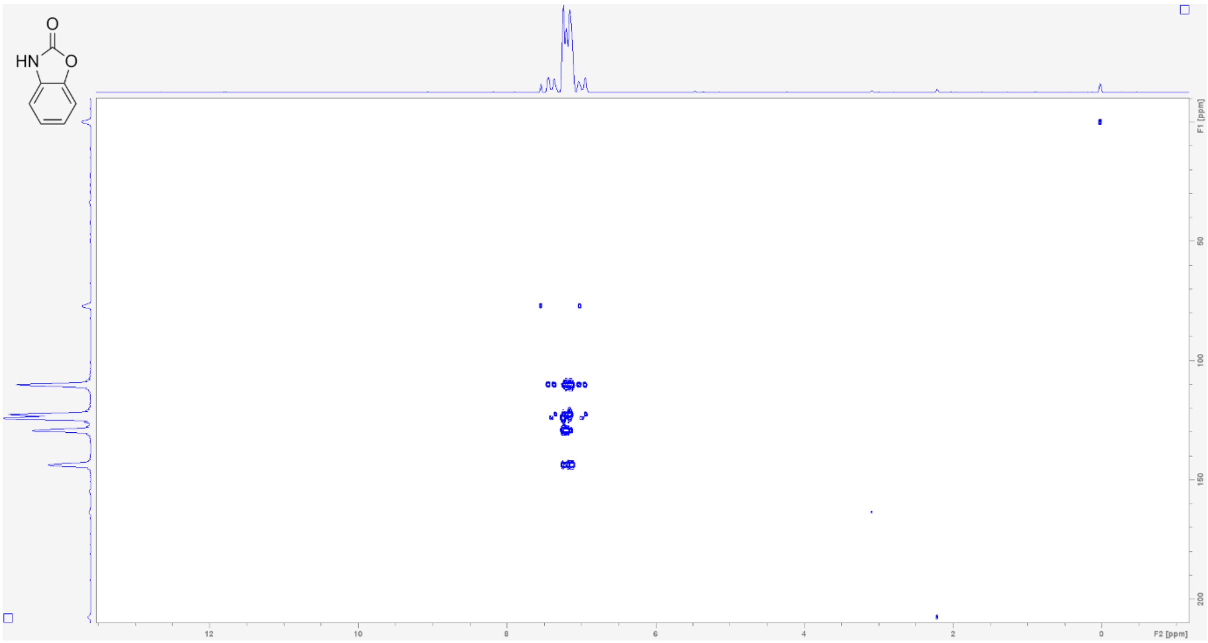

HMBC Spectrum of compound **2d** in $CDCl_3$ at 25°C. ^{1}H-frequency 400 MHz spectrometer.

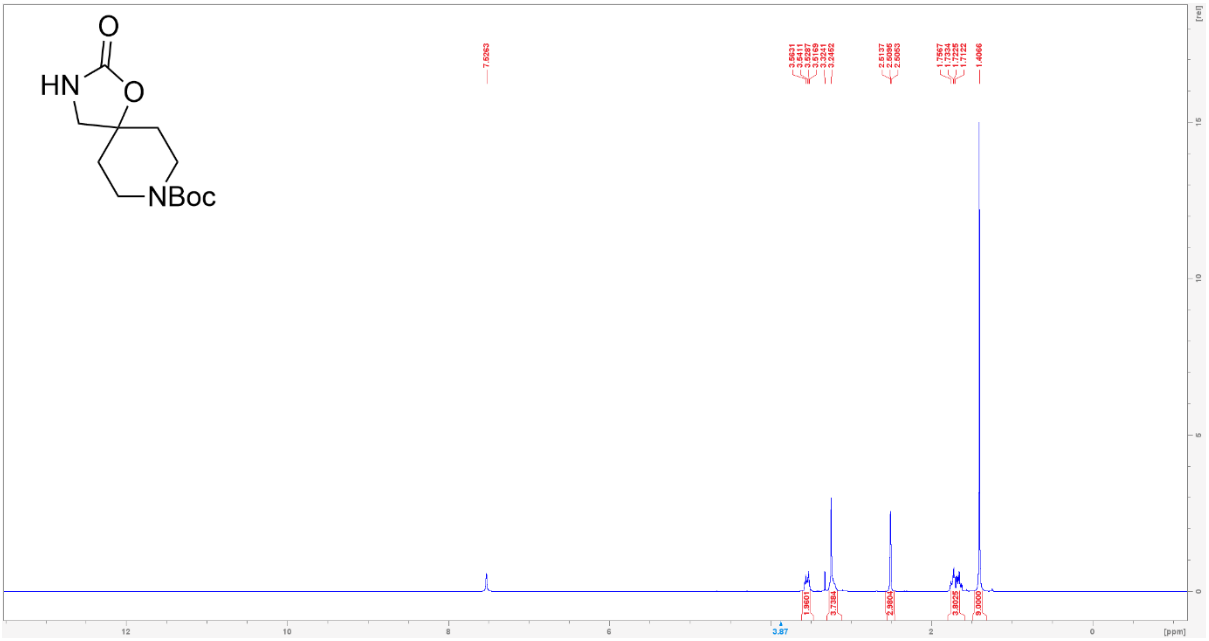

^{1}H Spectrum of compound **2e** in DMSO-d_6 at 25°C. ^{1}H-frequency 400 MHz spectrometer.

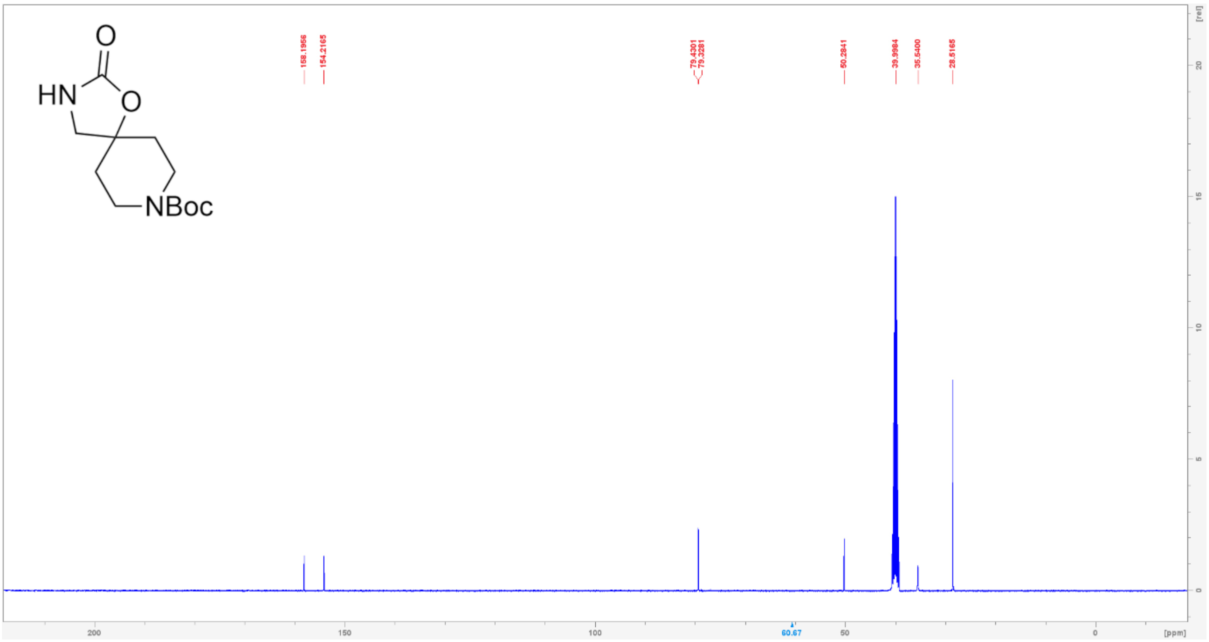

^{13}C{^{1}H} Spectrum of compound **2e** in DMSO-d_6 at 25°C. ^{1}H-frequency 400 MHz spectrometer.

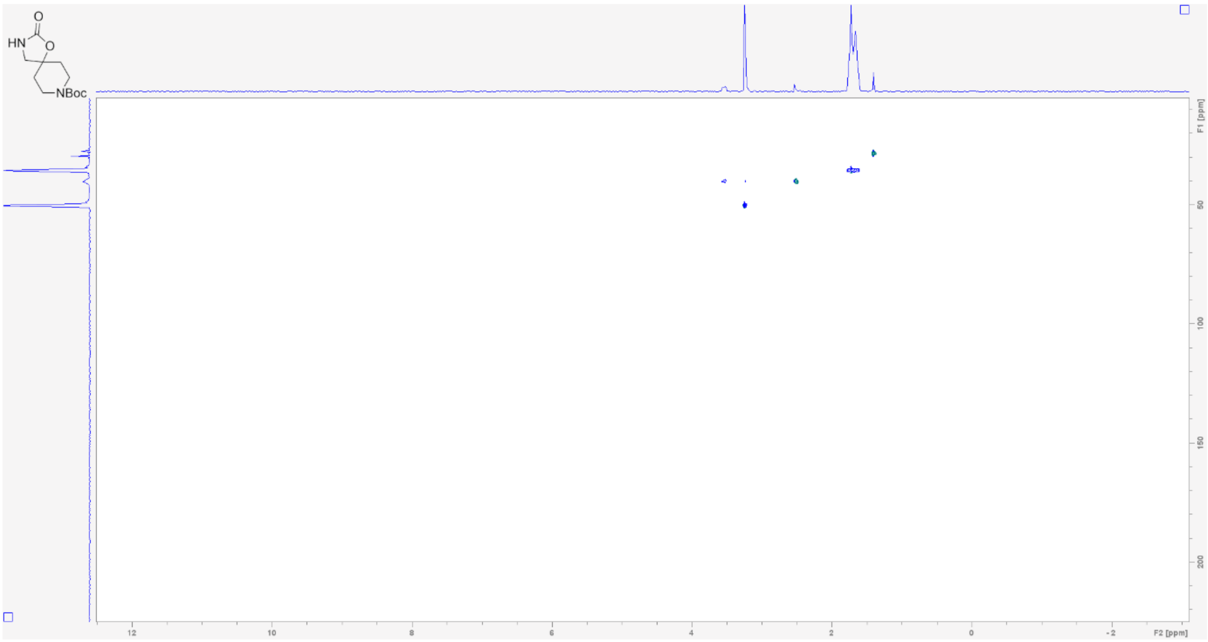

HSQC Spectrum of compound **2e** in DMSO-d$_6$ at 25°C. [1]H-frequency 400 MHz spectrometer.

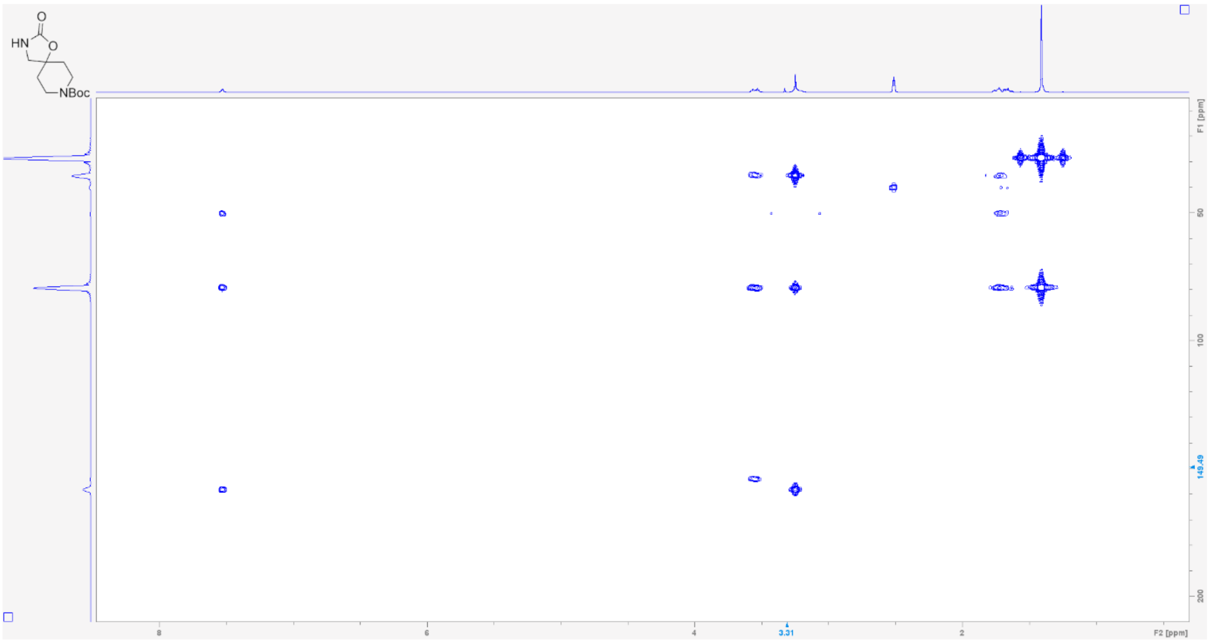

HMBC Spectrum of compound **2e** in DMSO-d$_6$ at 25°C. [1]H-frequency 400 MHz spectrometer.

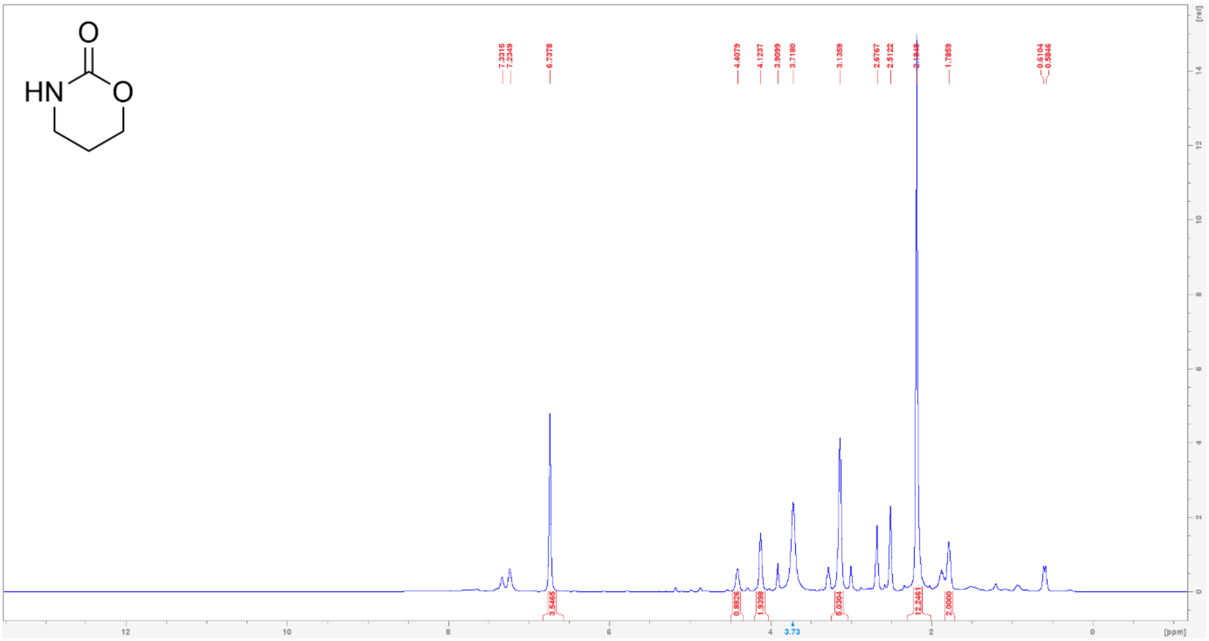

[1]H Spectrum of compound **2f** in DMSO-d$_6$ at 25°C with mesitylene as internal standard. [1]H-frequency 400 MHz spectrometer.

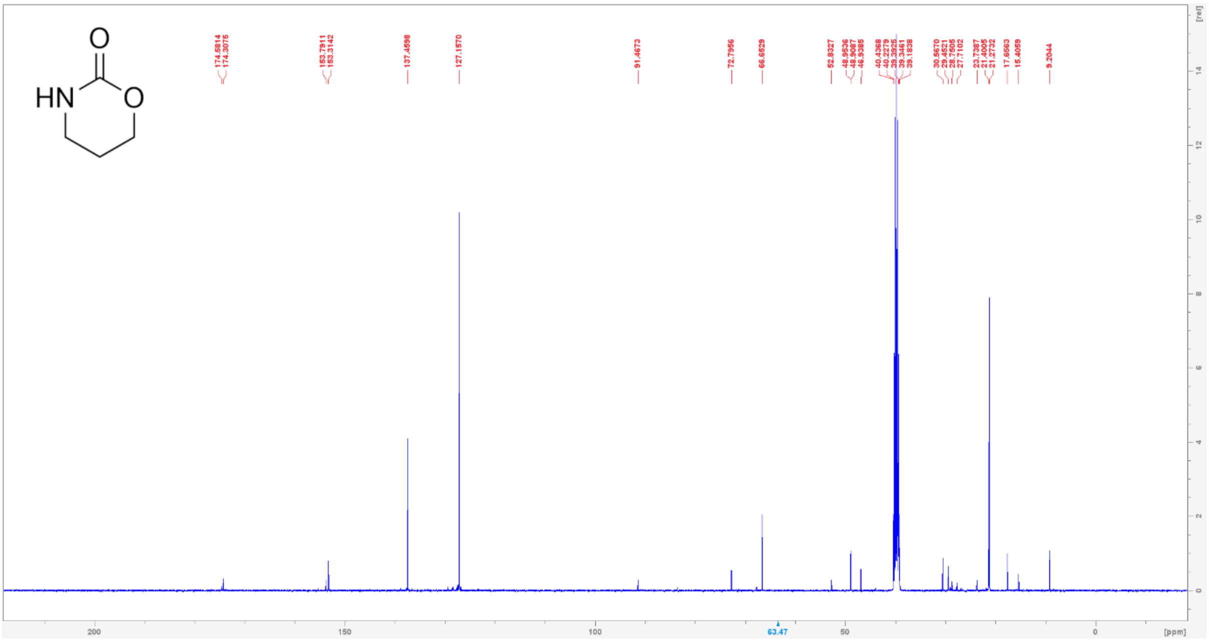

[13]C{[1]H} Spectrum of compound **2f** in DMSO-d$_6$ at 25°C with mesitylene as internal standard. [1]H-frequency 400 MHz spectrometer.

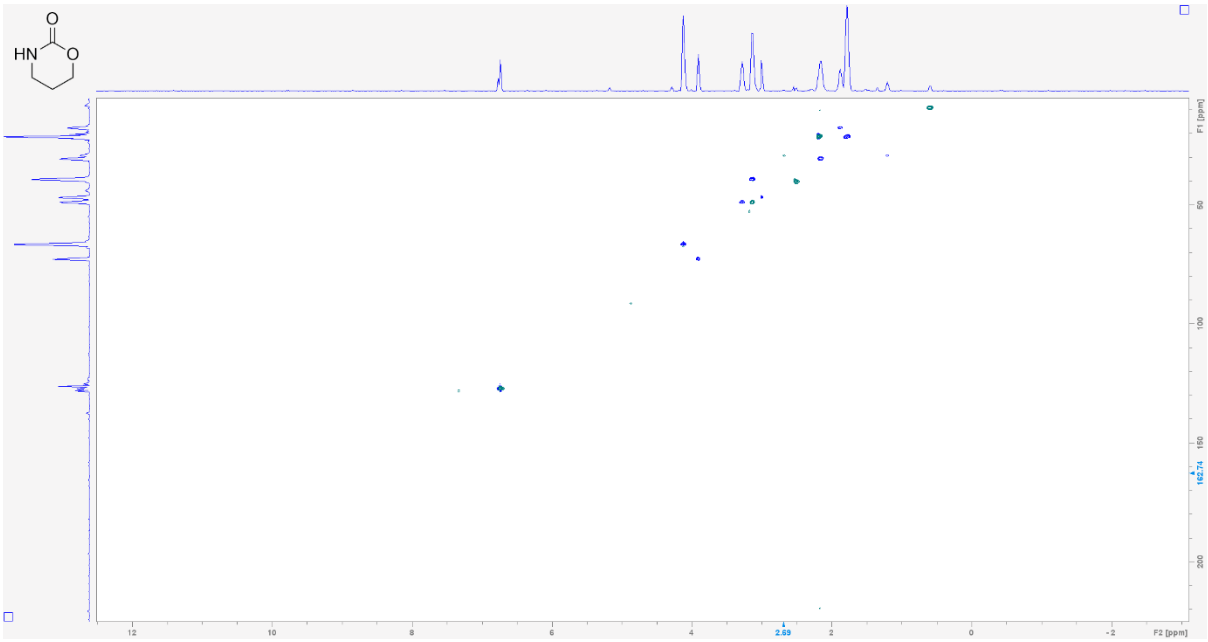

HSQC Spectrum of compound **2f** in DMSO-d_6 at 25°C with mesitylene as internal standard. [1]H-frequency 400 MHz spectrometer.

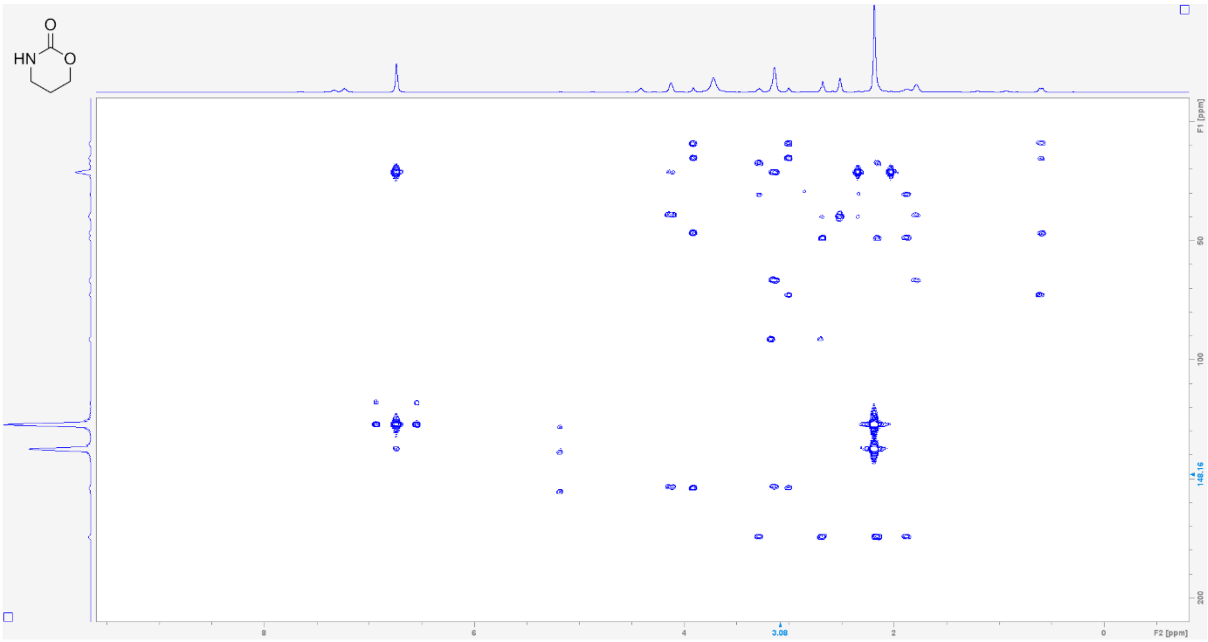

HMBC Spectrum of compound **2f** in DMSO-d_6 at 25°C with mesitylene as internal standard. [1]H-frequency 400 MHz spectrometer.

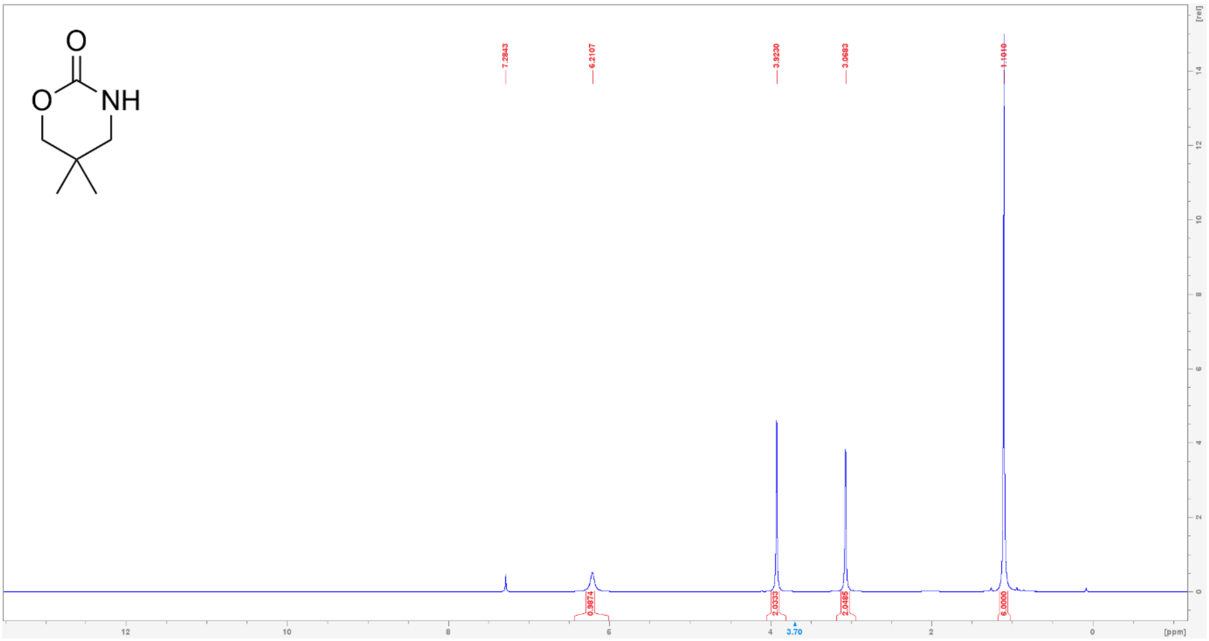

[1]H Spectrum of compound **2g** in $CDCl_3$ at 25°C. [1]H-frequency 400 MHz spectrometer.

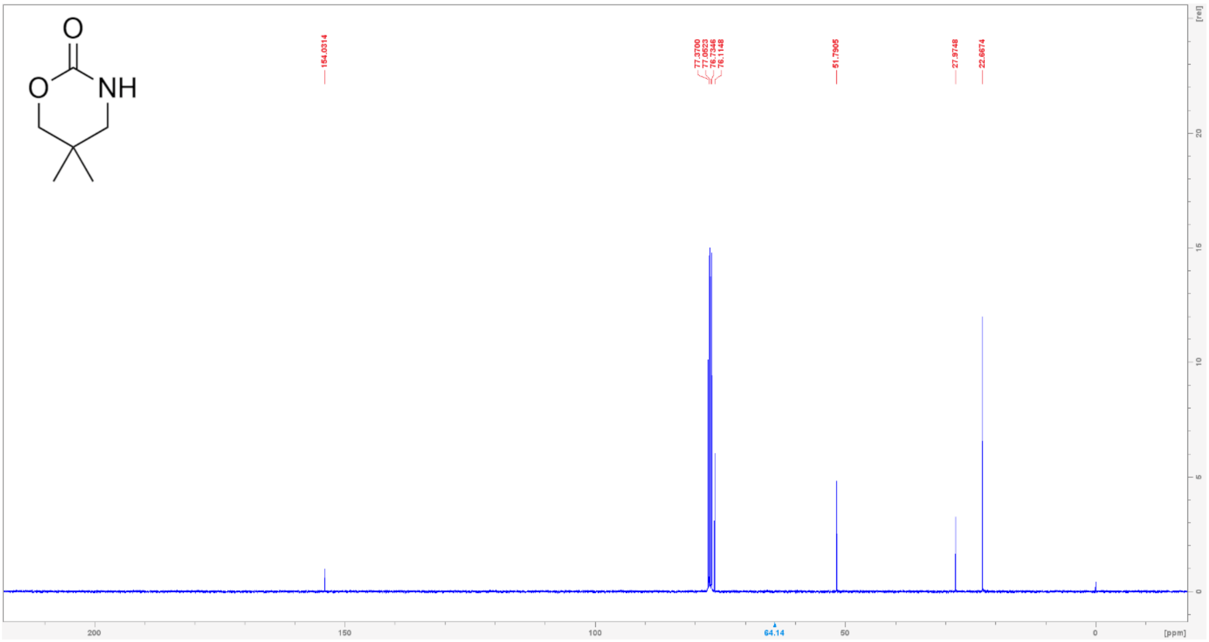

[13]C{[1]H} Spectrum of compound **2g** in $CDCl_3$ at 25°C. [1]H-frequency 400 MHz spectrometer.

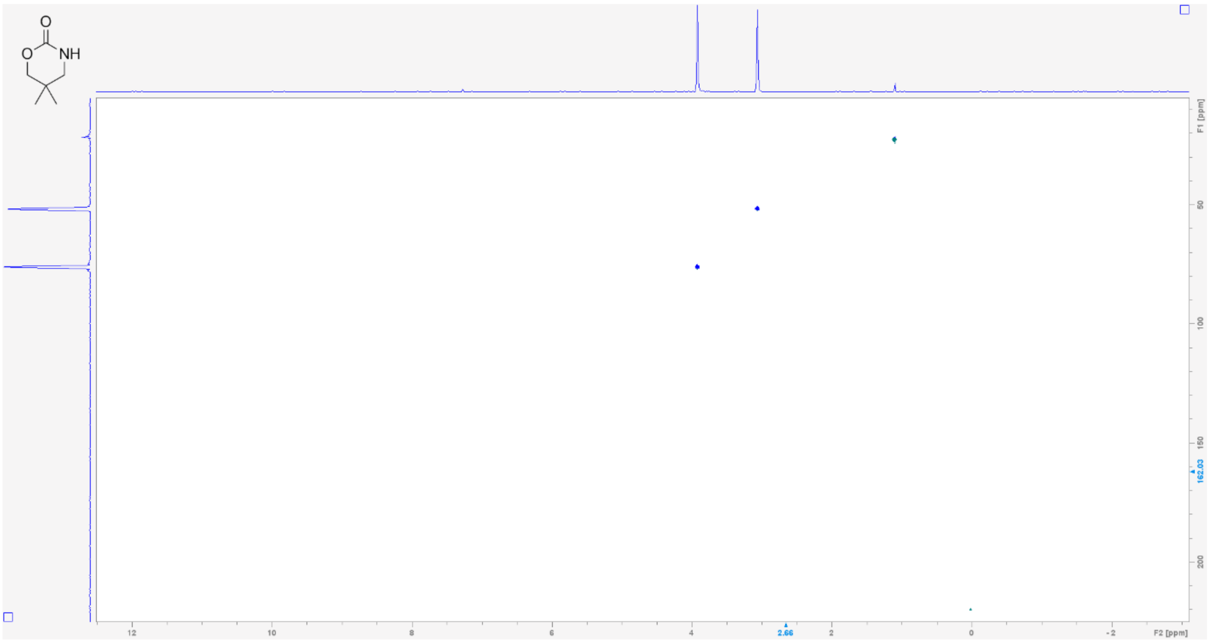

HSQC Spectrum of compound **2g** in $CDCl_3$ at 25°C. [1]H-frequency 400 MHz spectrometer.

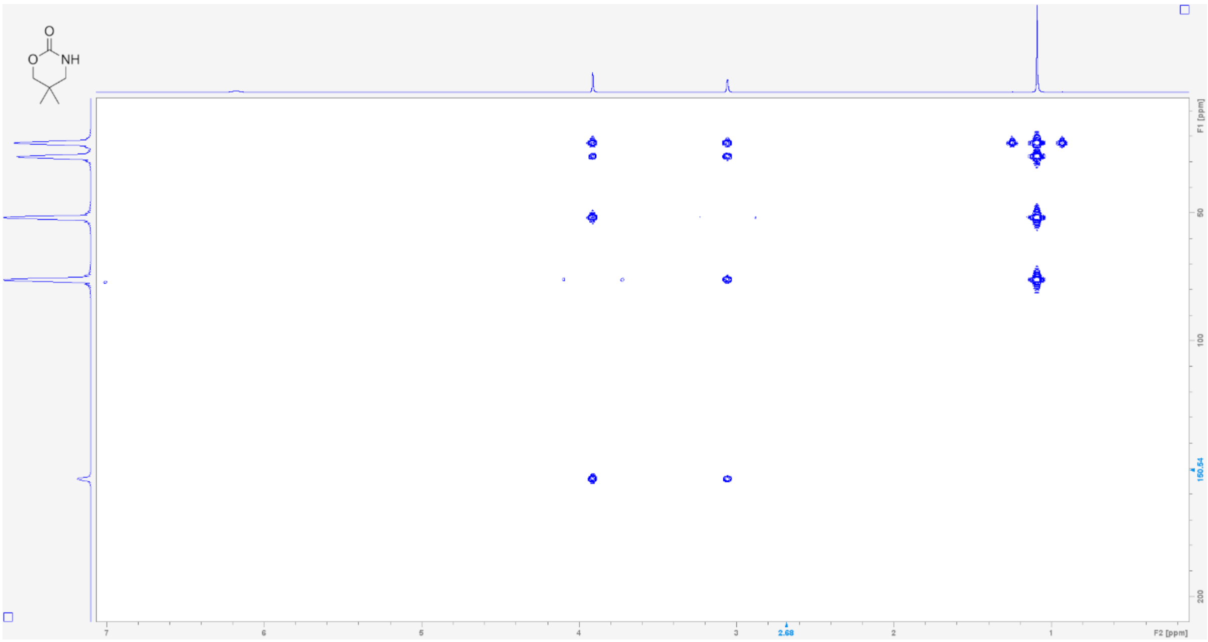

HMBC Spectrum of compound **2g** in $CDCl_3$ at 25°C. [1]H-frequency 400 MHz spectrometer.

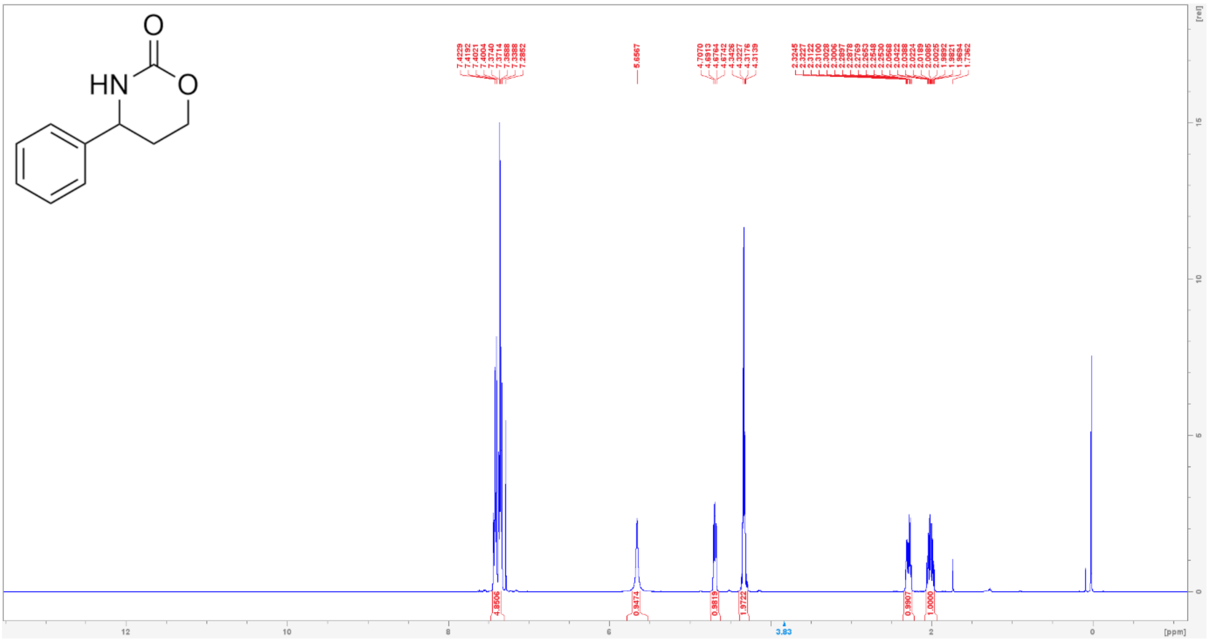

[1]H Spectrum of compound **2h** in $CDCl_3$ at 25°C. [1]H-frequency 400 MHz spectrometer.

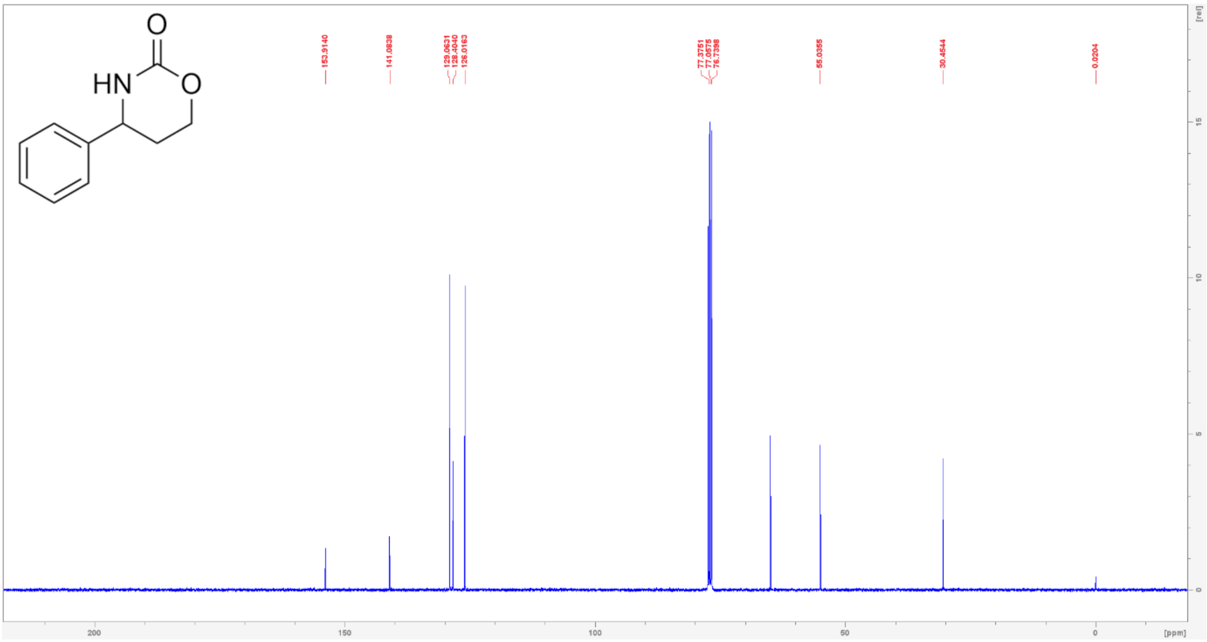

[13]C{[1]H} Spectrum of compound **2h** in $CDCl_3$ at 25°C. [1]H-frequency 400 MHz spectrometer.

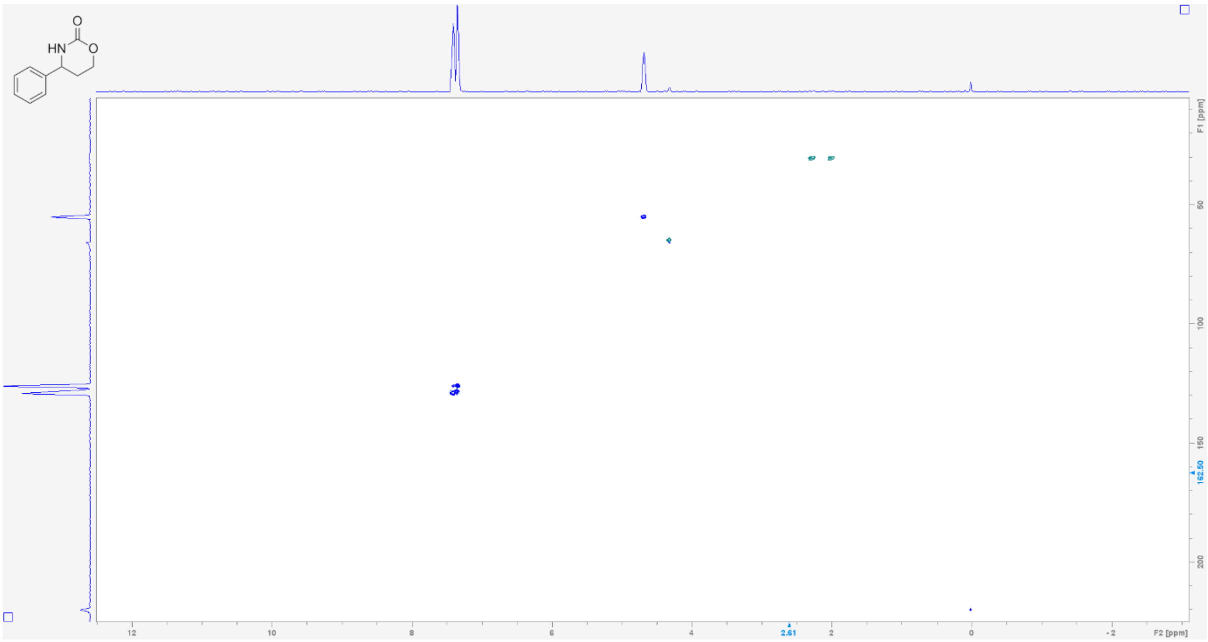

HSQC Spectrum of compound **2h** in $CDCl_3$ at 25°C. [1]H-frequency 400 MHz spectrometer.

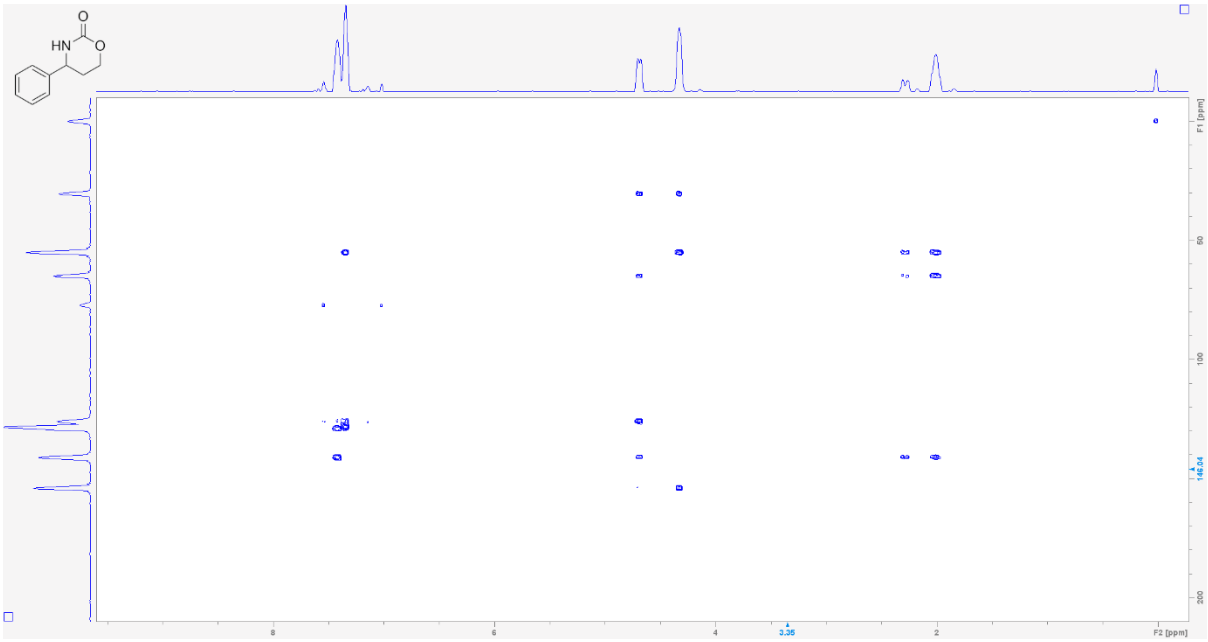

HMBC Spectrum of compound **2h** in $CDCl_3$ at 25°C. [1]H-frequency 400 MHz spectrometer.

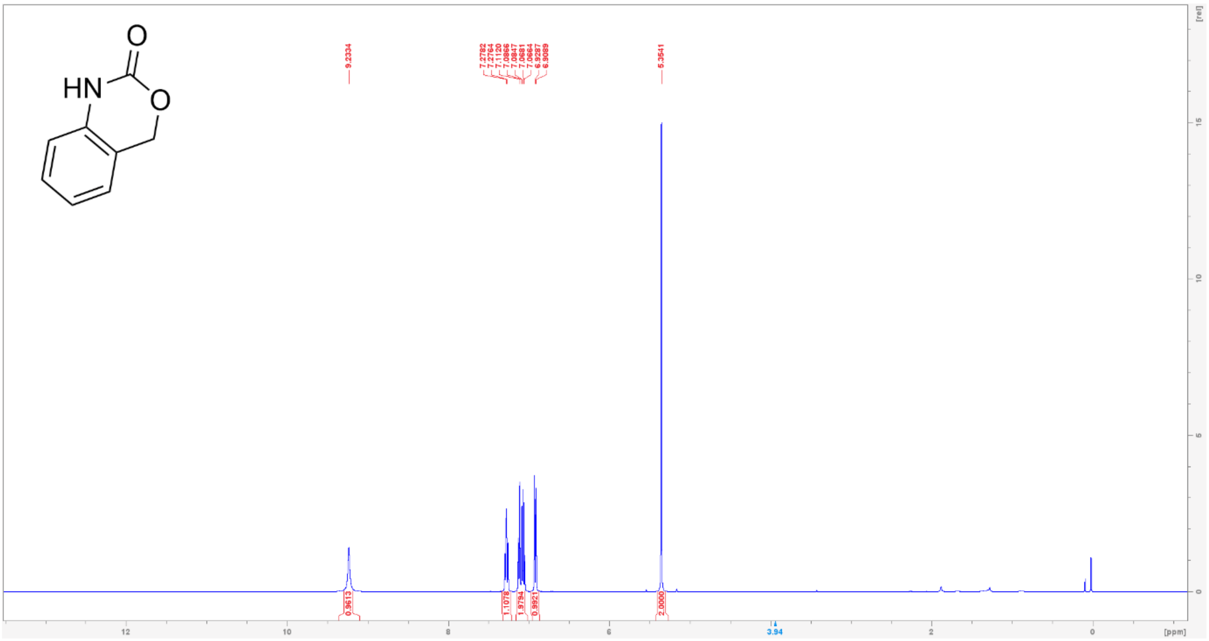

^{1}H Spectrum of compound **2i** in $CDCl_3$ at 25°C. ^{1}H-frequency 400 MHz spectrometer.

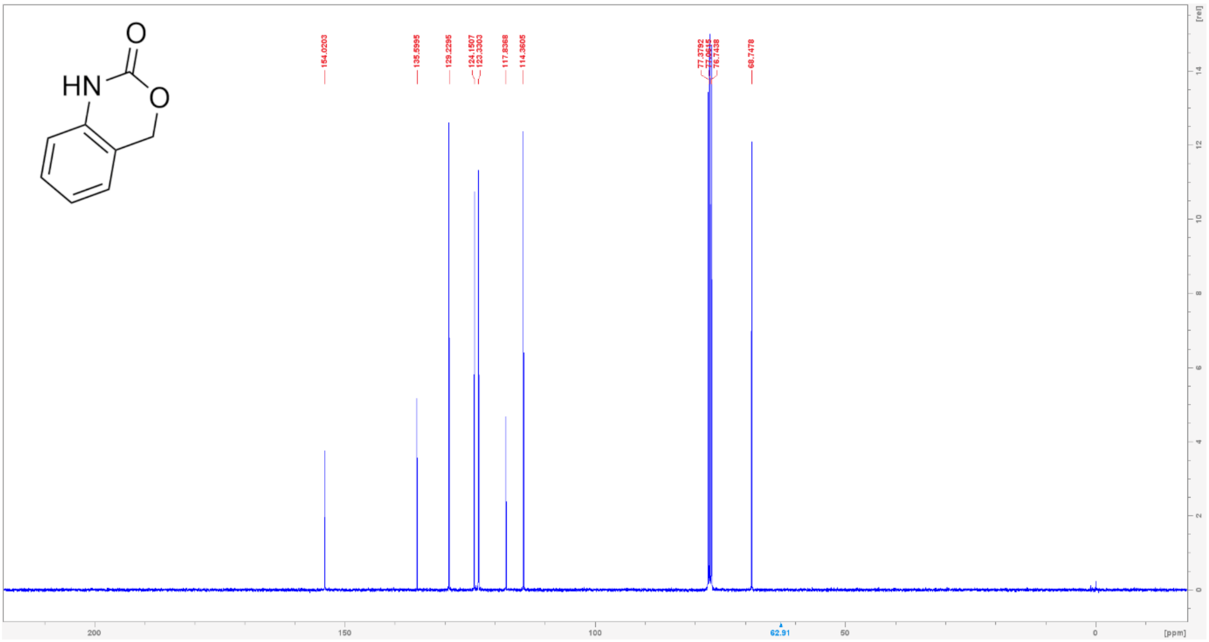

^{13}C{^{1}H} Spectrum of compound **2i** in $CDCl_3$ at 25°C. ^{1}H-frequency 400 MHz spectrometer.

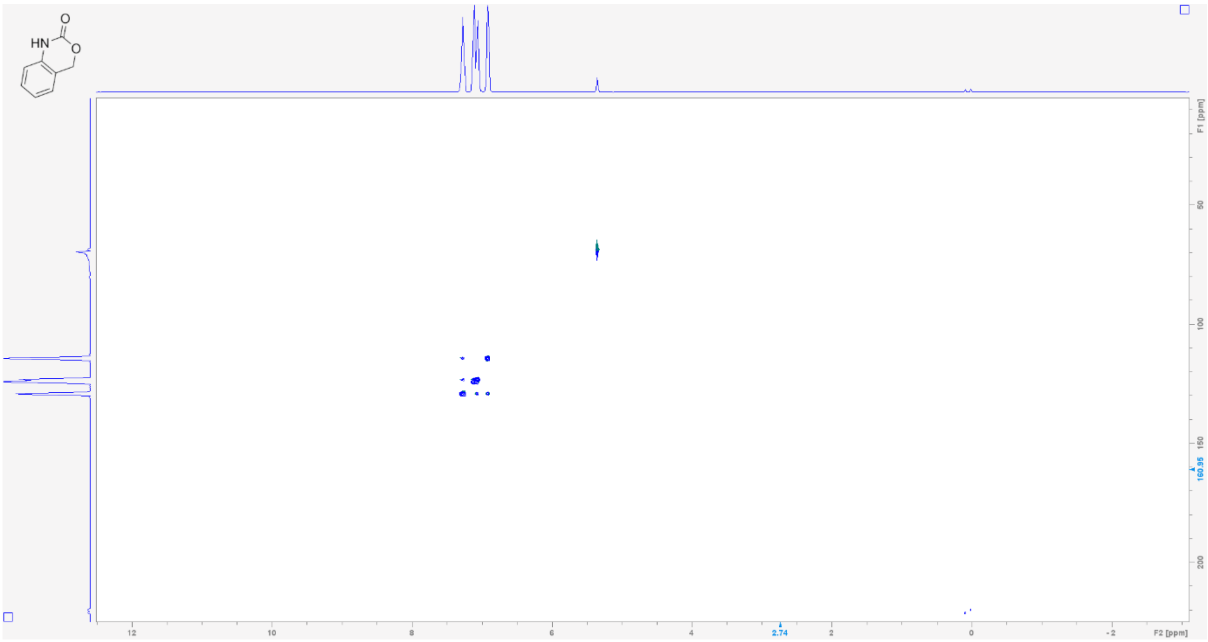

HSQC Spectrum of compound **2i** in $CDCl_3$ at 25°C. ^{1}H-frequency 400 MHz spectrometer.

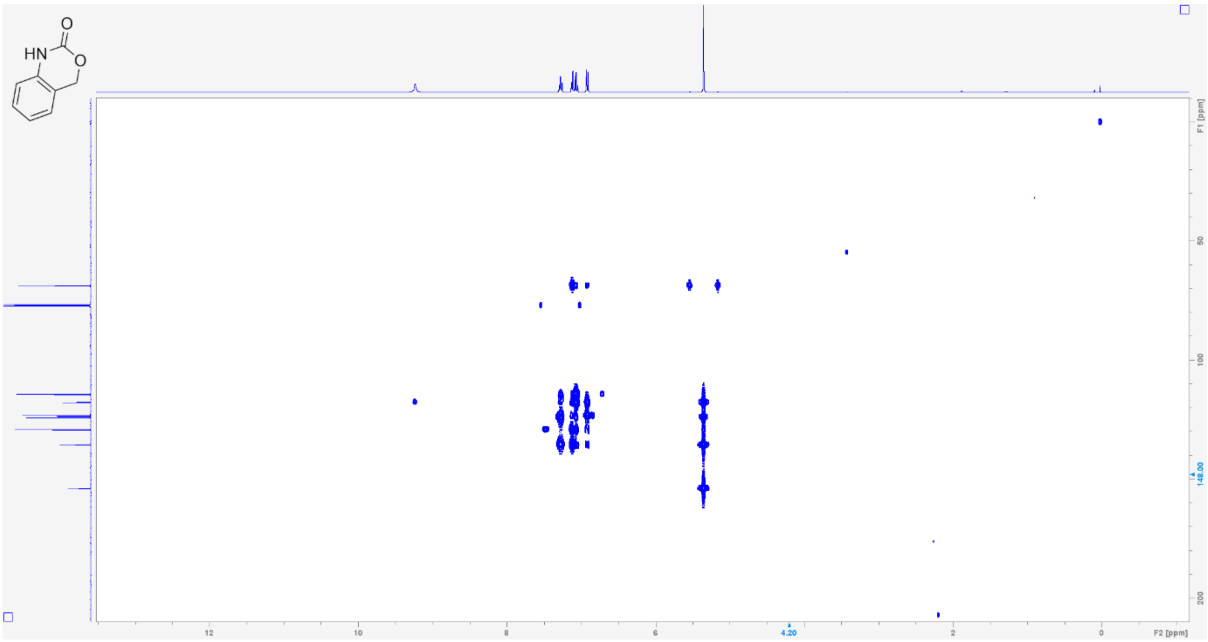

HMBC Spectrum of compound **2i** in $CDCl_3$ at 25°C. ^{1}H-frequency 400 MHz spectrometer.

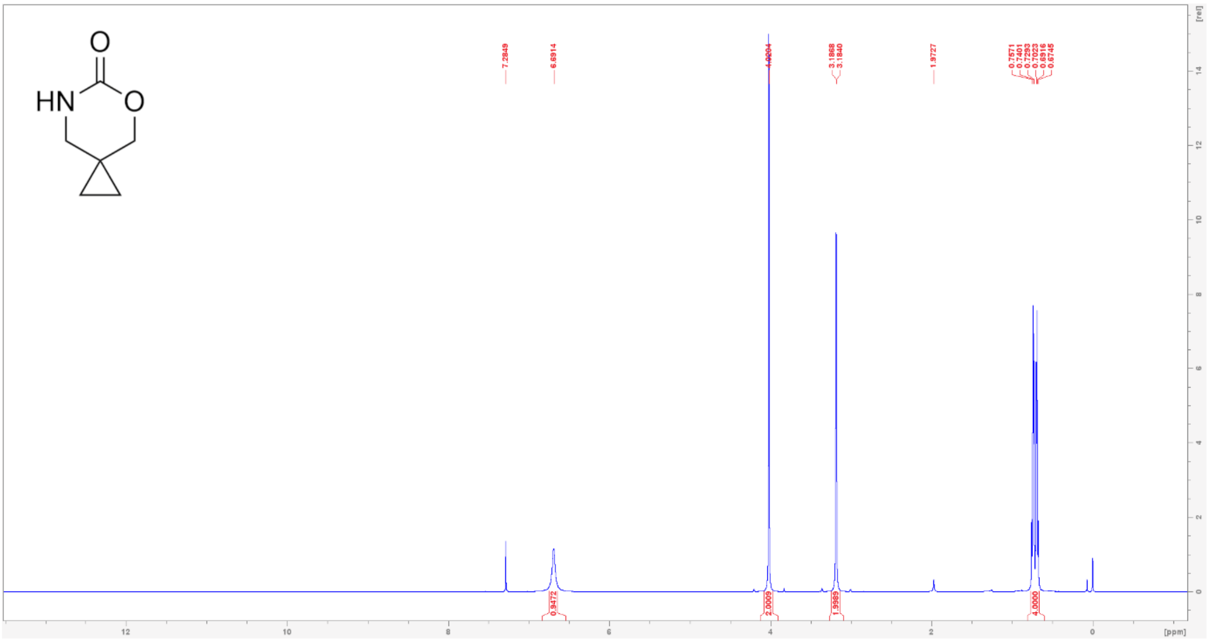

^{1}H Spectrum of compound **2j** in $CDCl_3$ at 25°C. ^{1}H-frequency 400 MHz spectrometer.

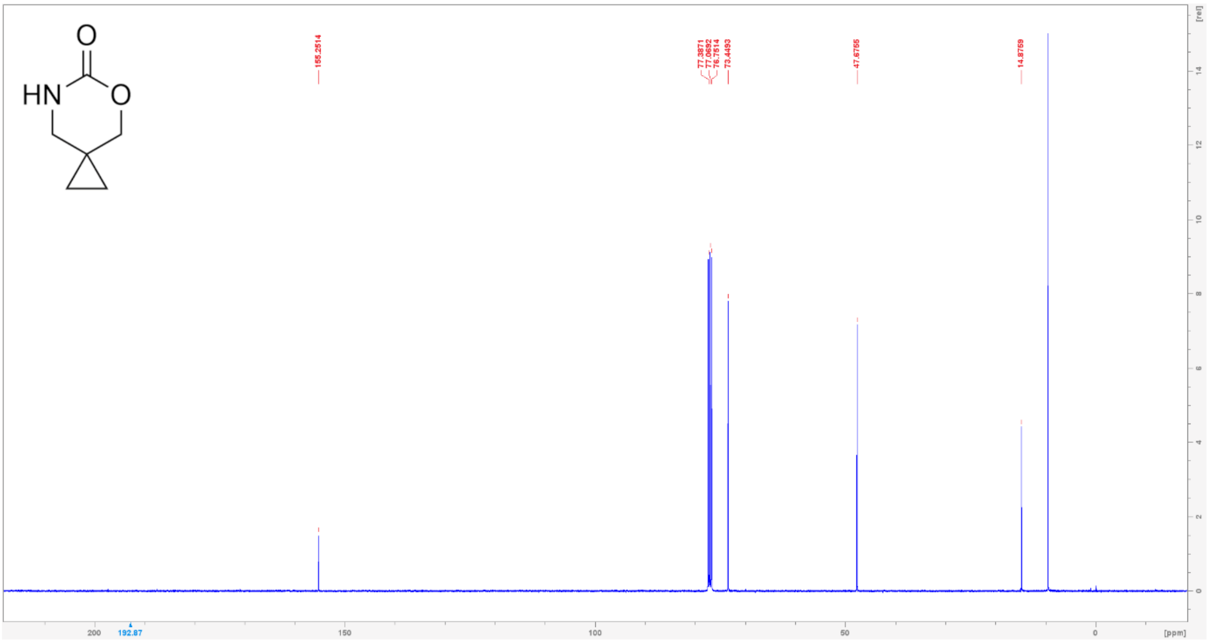

$^{13}C\{^{1}H\}$ Spectrum of compound **2j** in $CDCl_3$ at 25°C. ^{1}H-frequency 400 MHz spectrometer.

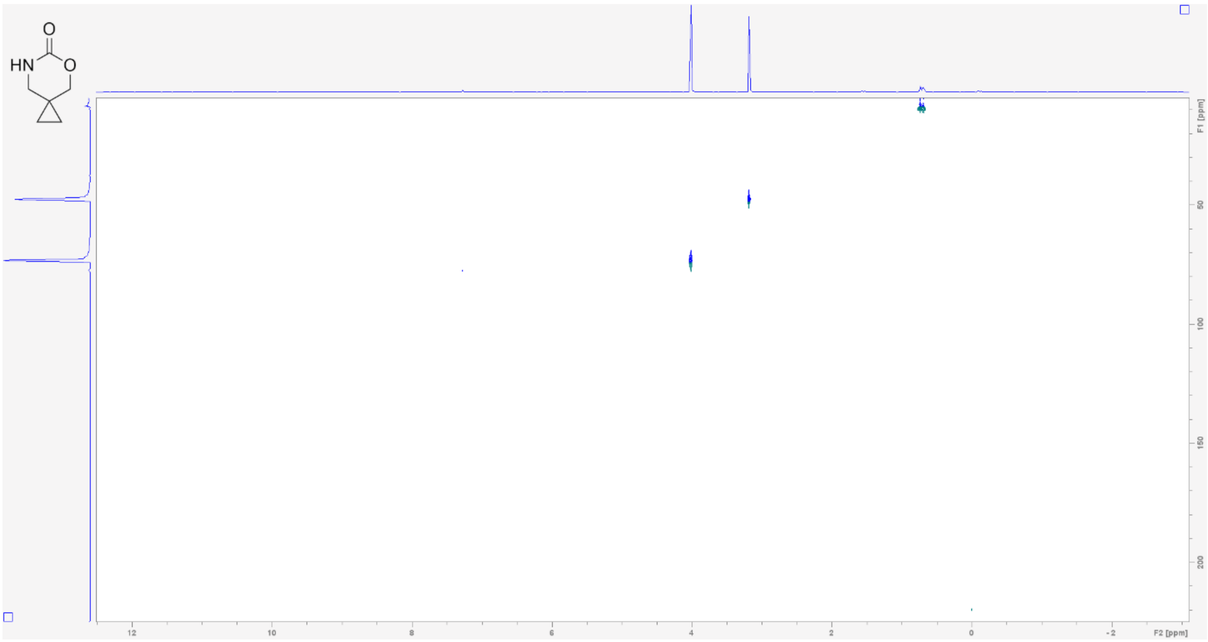

HSQC Spectrum of compound **2j** in $CDCl_3$ at 25°C. [1]H-frequency 400 MHz spectrometer.

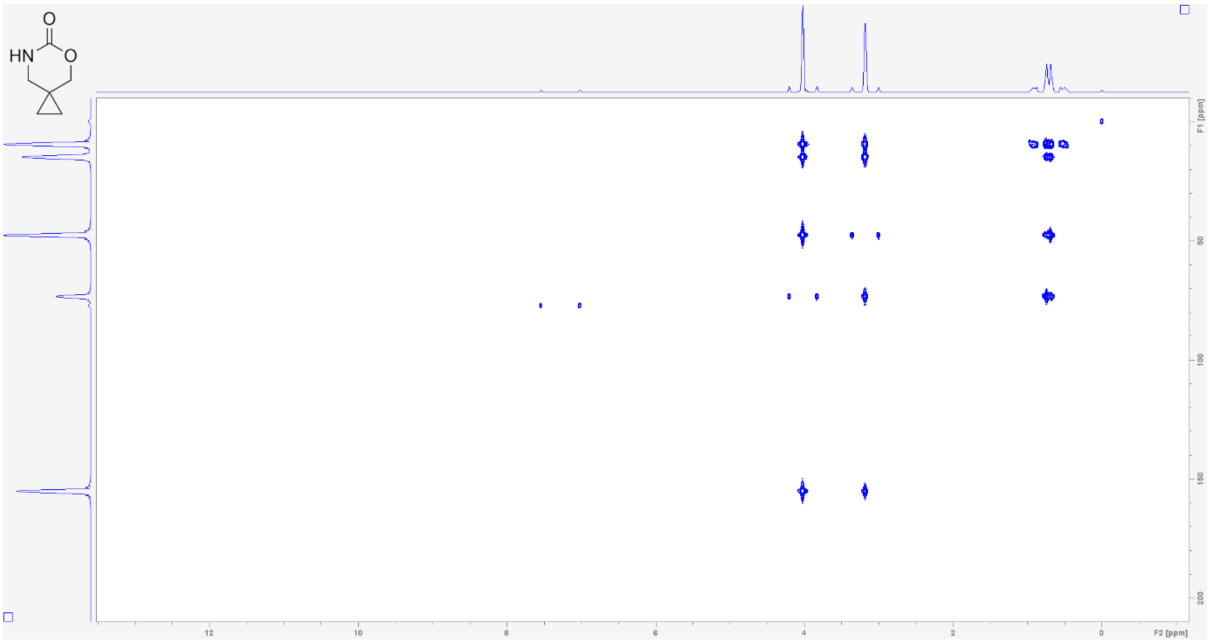

HMBC Spectrum of compound **2j** in $CDCl_3$ at 25°C. [1]H-frequency 400 MHz spectrometer.

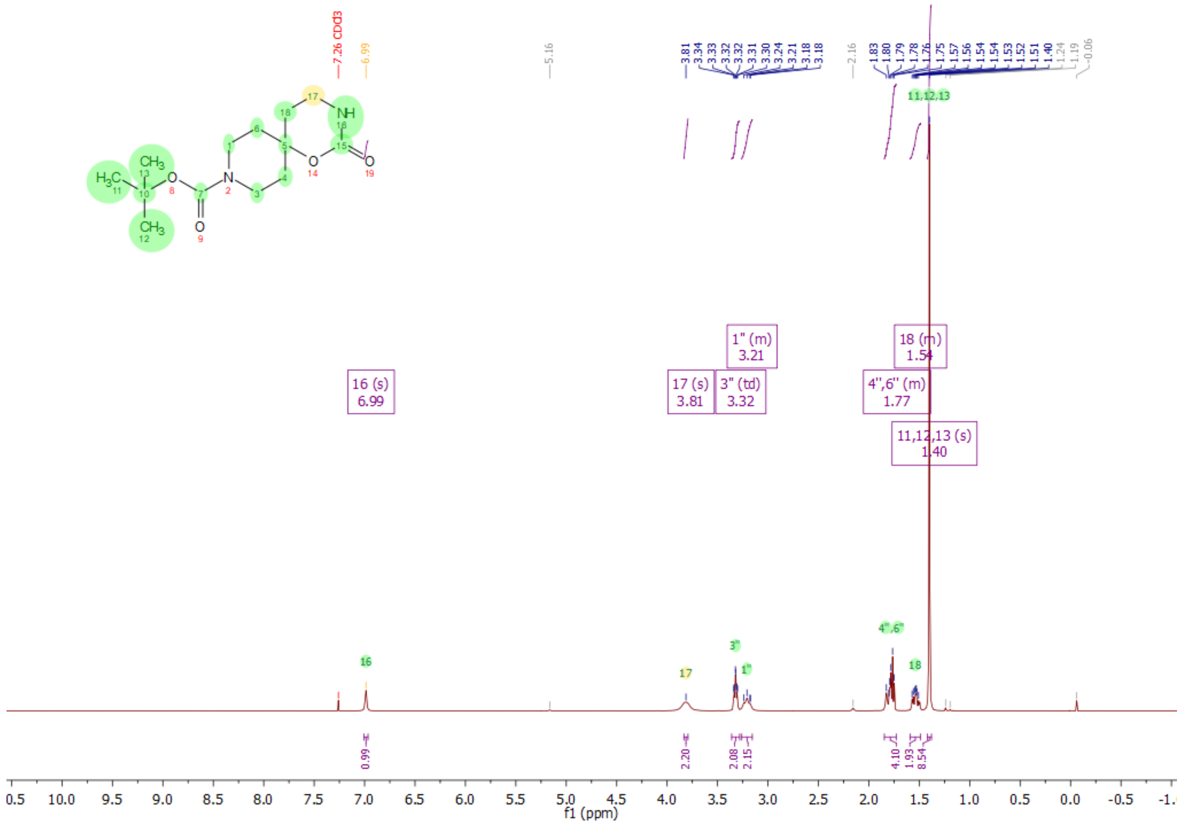

[1]H Spectrum of compound **2k** in $CDCl_3$ at 25°C. [1]H-frequency 400 MHz spectrometer.

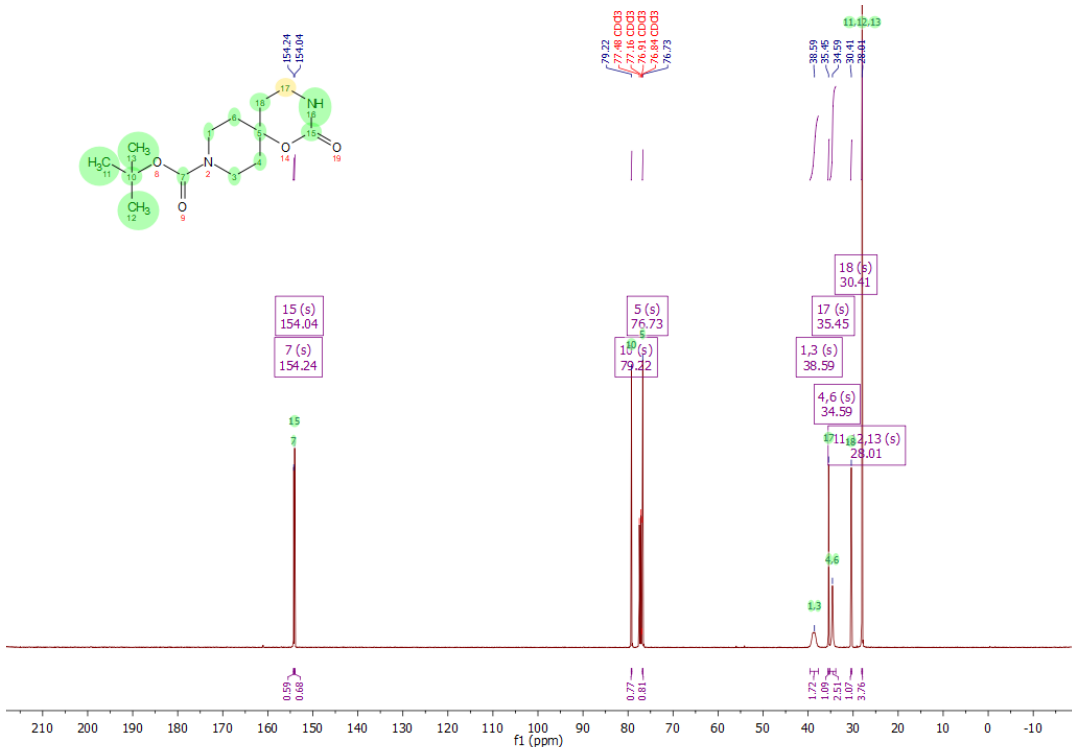

[13]C{[1]H} Spectrum of compound **2k** in $CDCl_3$ at 25°C. [1]H-frequency 400 MHz spectrometer.

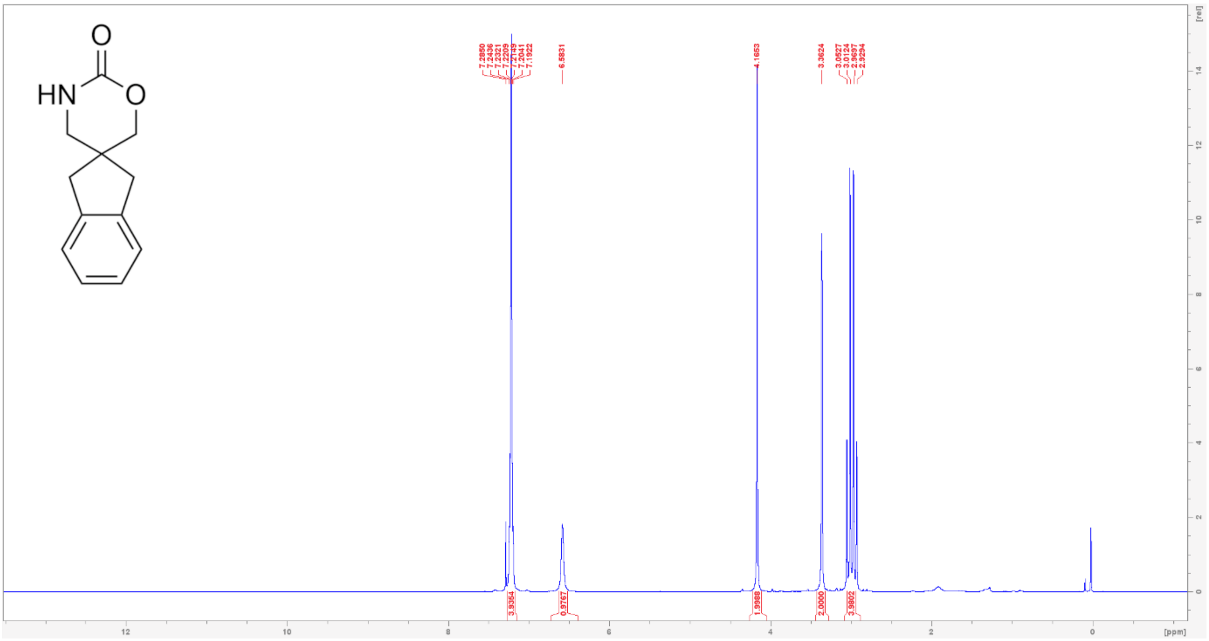

^{1}H Spectrum of compound **2l** in $CDCl_3$ at 25°C. ^{1}H-frequency 400 MHz spectrometer.

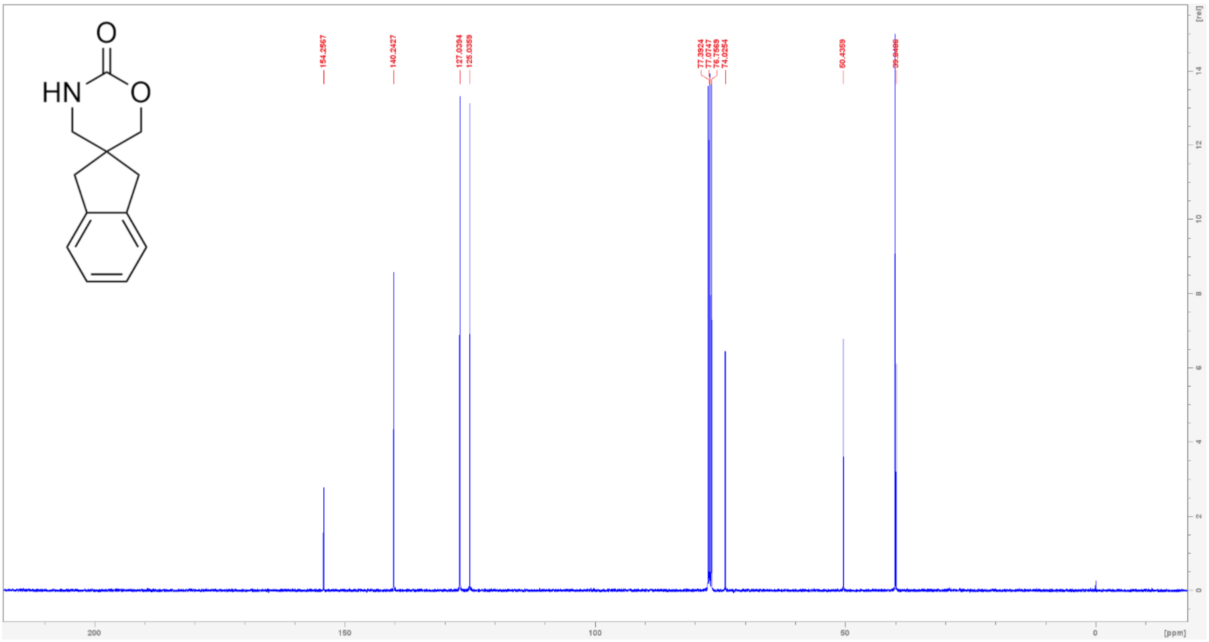

^{13}C{^{1}H} Spectrum of compound **2l** in $CDCl_3$ at 25°C. ^{1}H-frequency 400 MHz spectrometer.

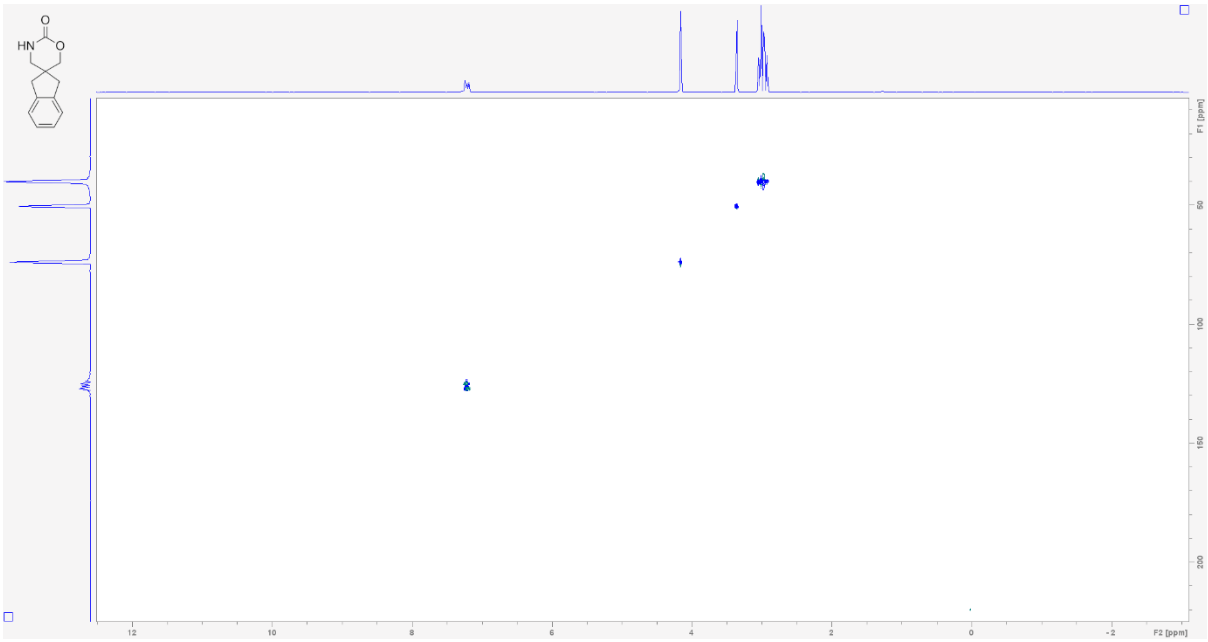

HSQC Spectrum of compound **2l** in $CDCl_3$ at 25°C. [1]H-frequency 400 MHz spectrometer.

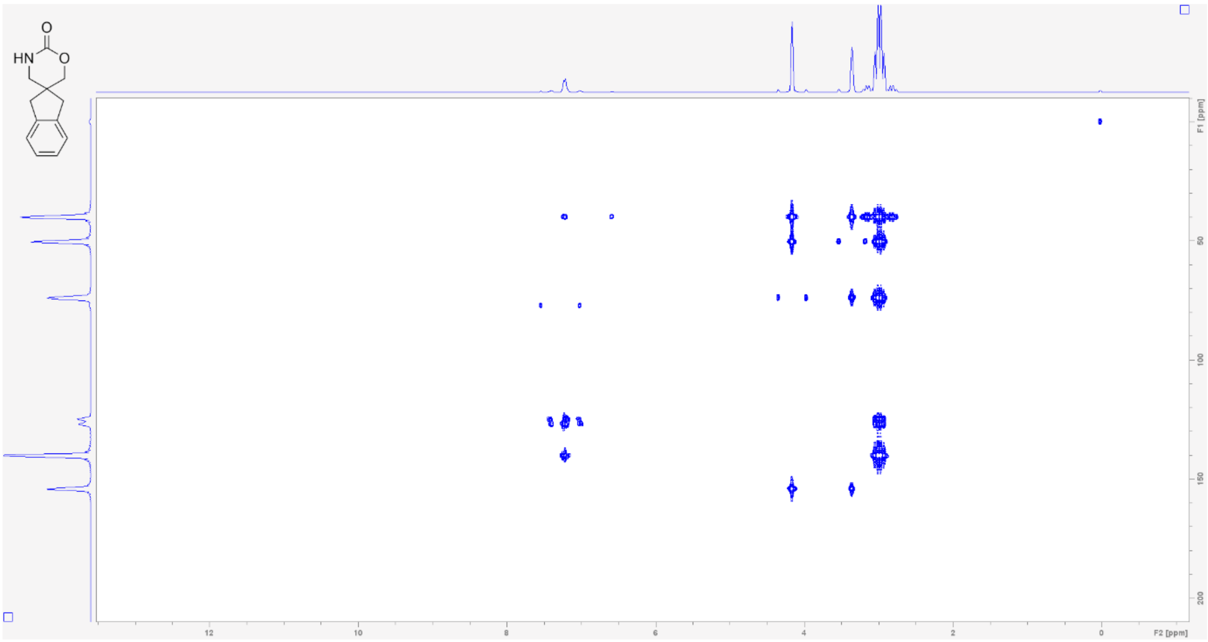

HMBC Spectrum of compound **2l** in $CDCl_3$ at 25°C. [1]H-frequency 400 MHz spectrometer.

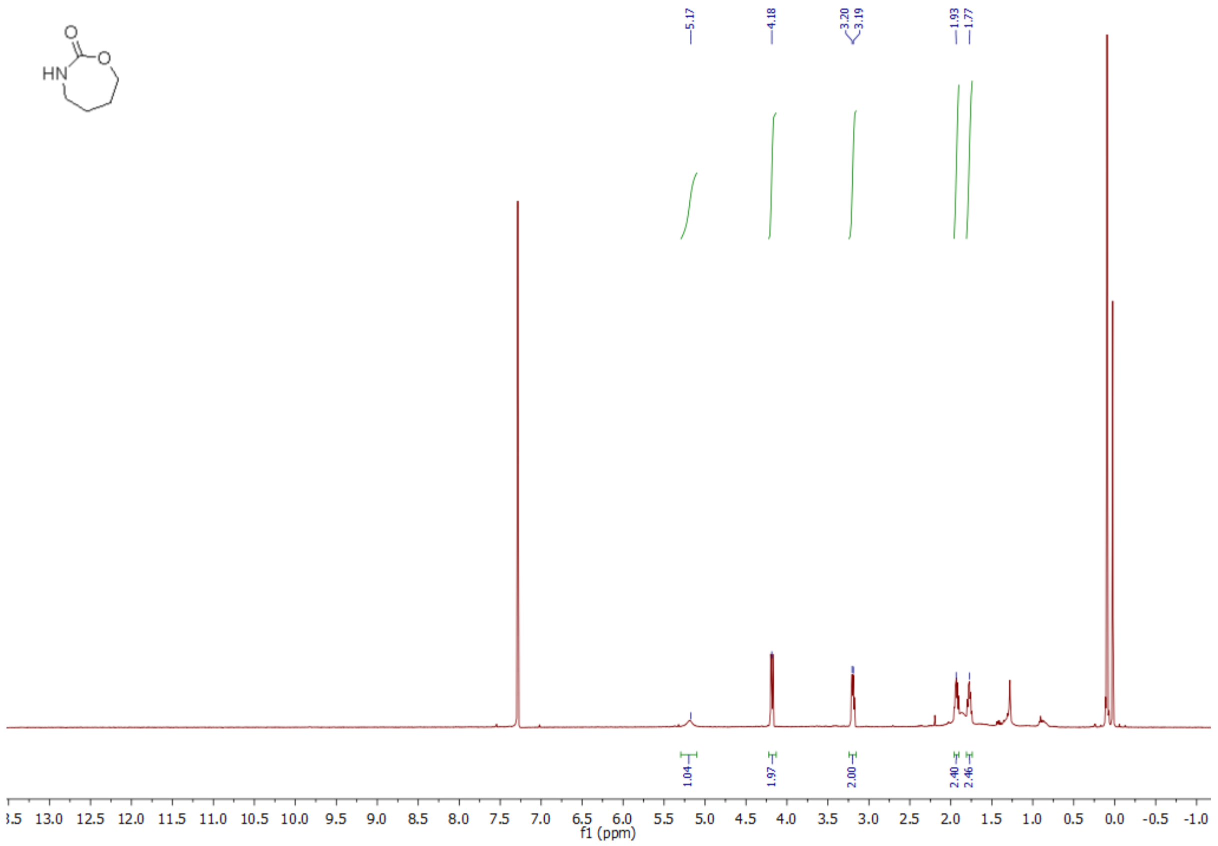

[1]H Spectrum of compound **2m** in $CDCl_3$ at 25°C. [1]H-frequency 400 MHz spectrometer.

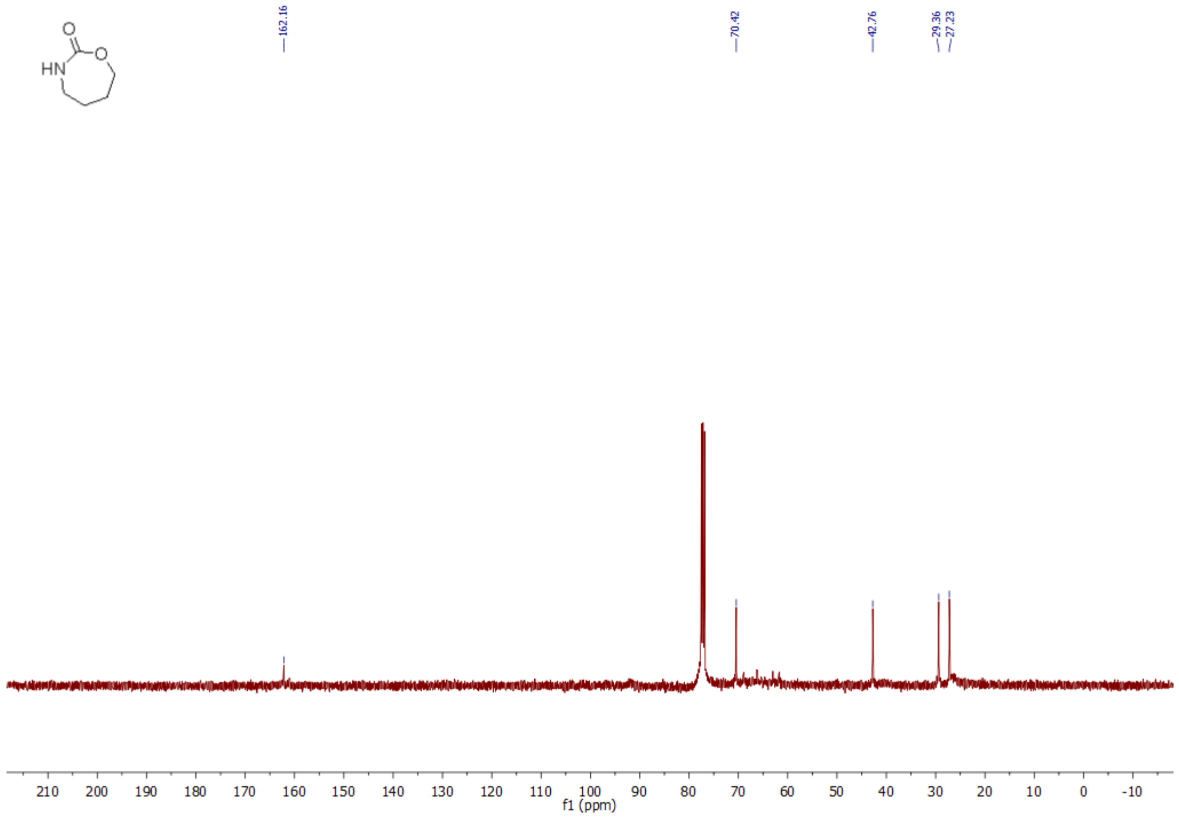

[13]C{[1]H} Spectrum of compound **2m** in $CDCl_3$ at 25°C. [1]H-frequency 400 MHz spectrometer.

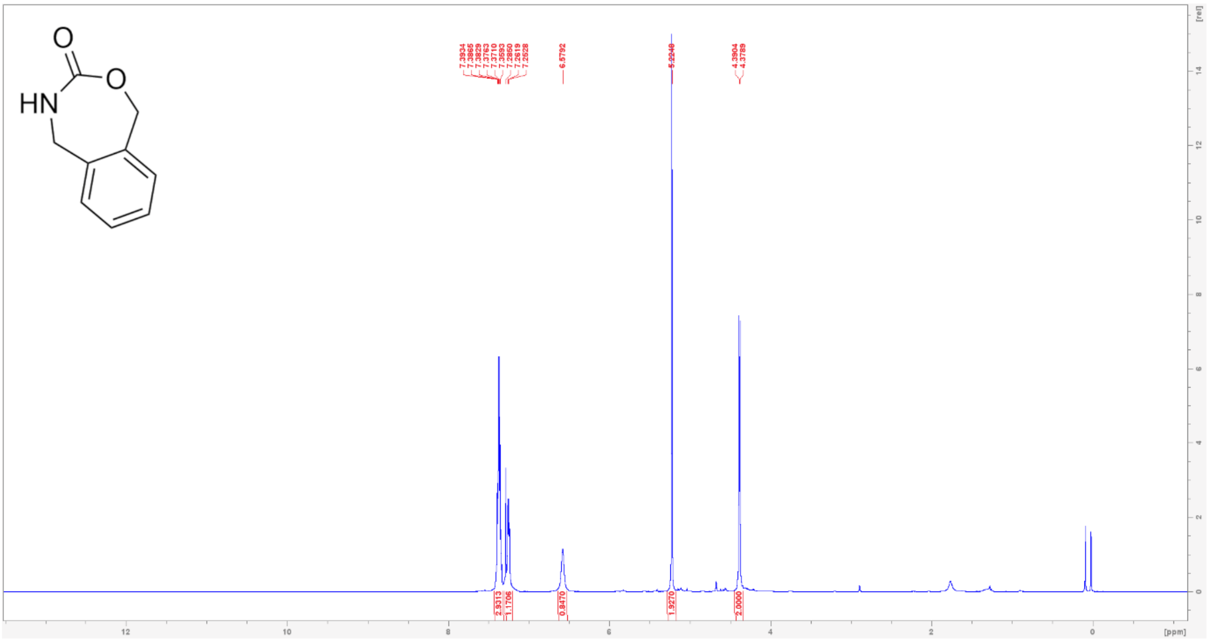

[1]H Spectrum of compound **2n** in $CDCl_3$ at 25°C. [1]H-frequency 400 MHz spectrometer.

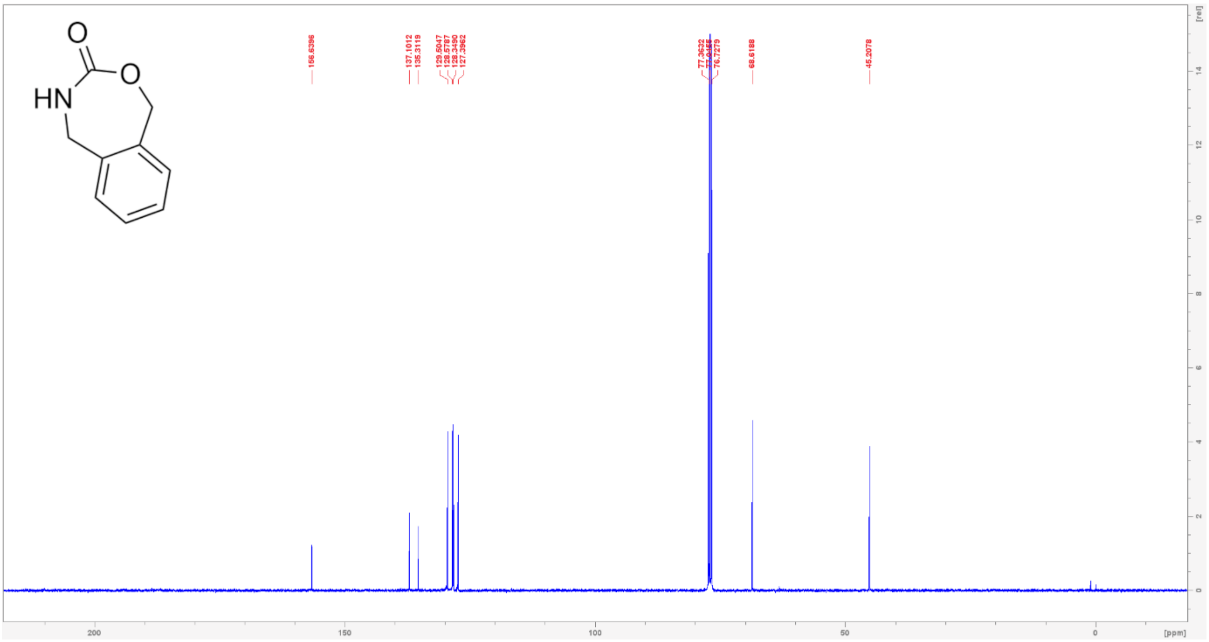

[13]C{[1]H} Spectrum of compound **2n** in $CDCl_3$ at 25°C. [1]H-frequency 400 MHz spectrometer.

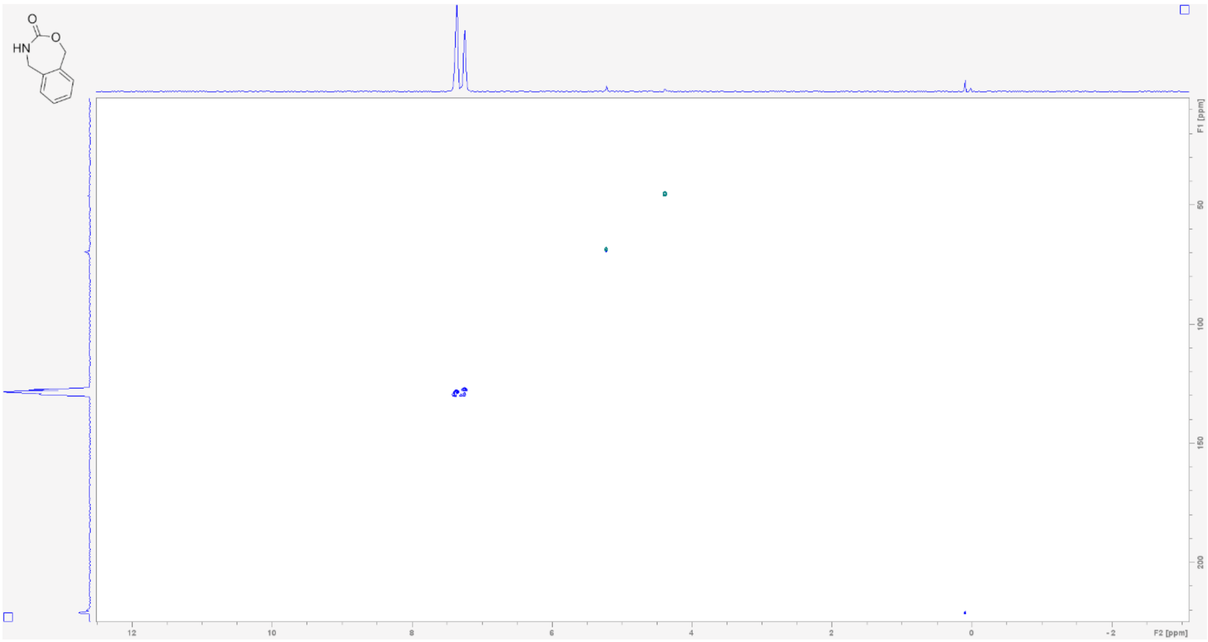

HSQC Spectrum of compound **2n** in $CDCl_3$ at 25°C. ^{1}H-frequency 400 MHz spectrometer.

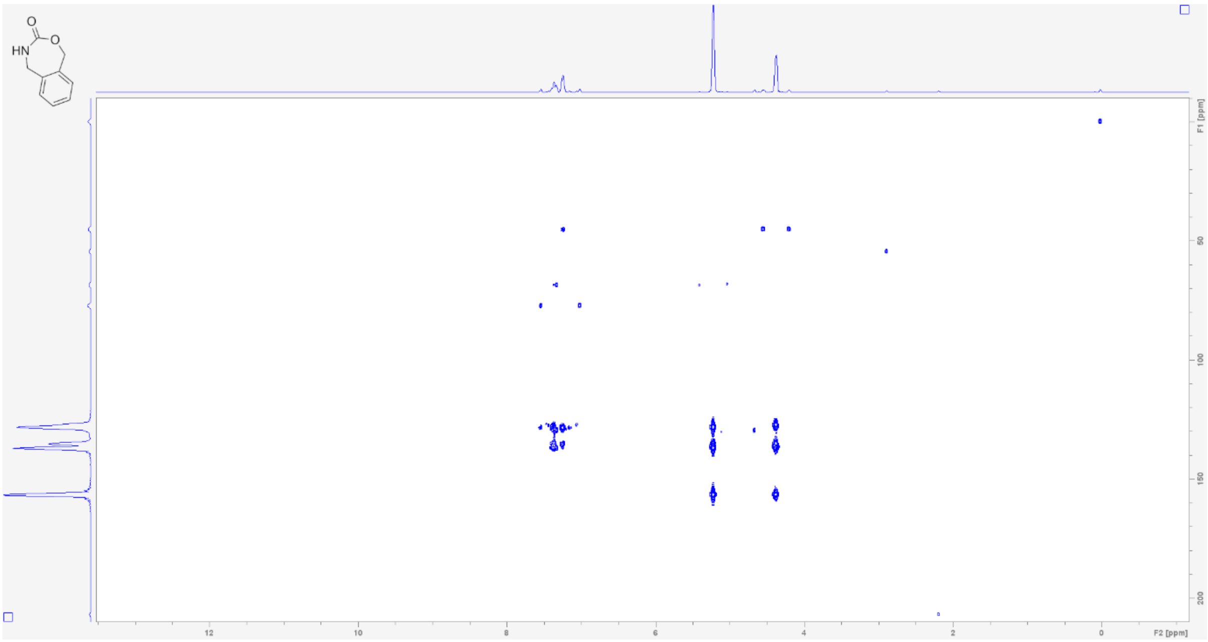

HMBC Spectrum of compound **2n** in $CDCl_3$ at 25°C. ^{1}H-frequency 400 MHz spectrometer.

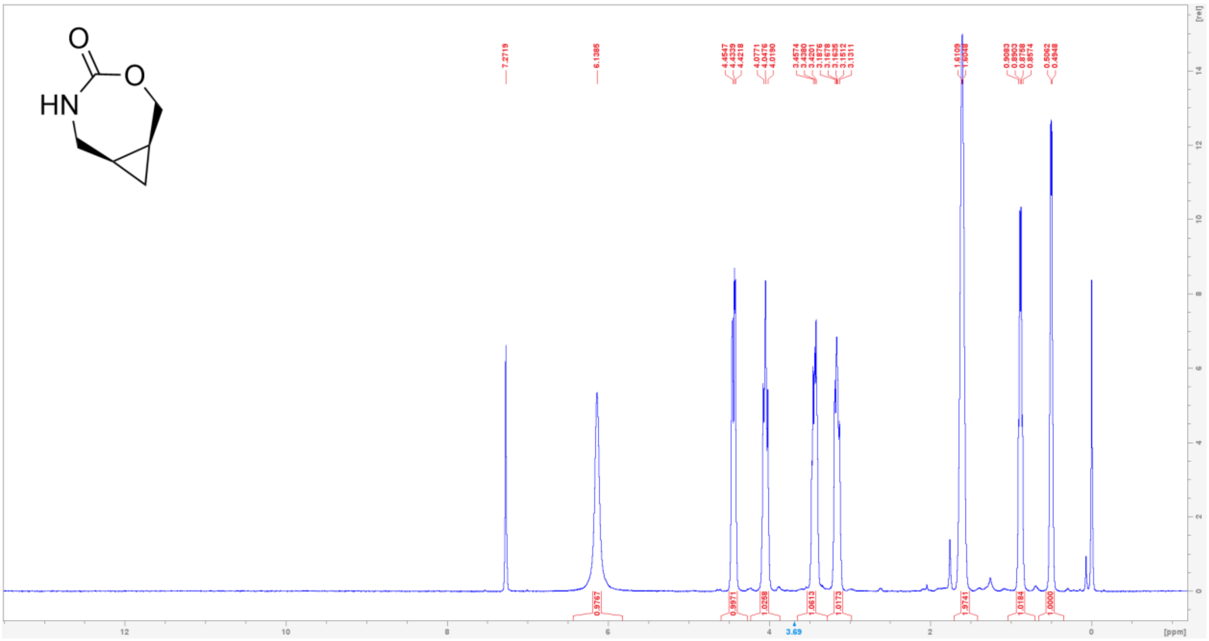

[1]H Spectrum of compound **2o** in $CDCl_3$ at 25°C. [1]H-frequency 400 MHz spectrometer.

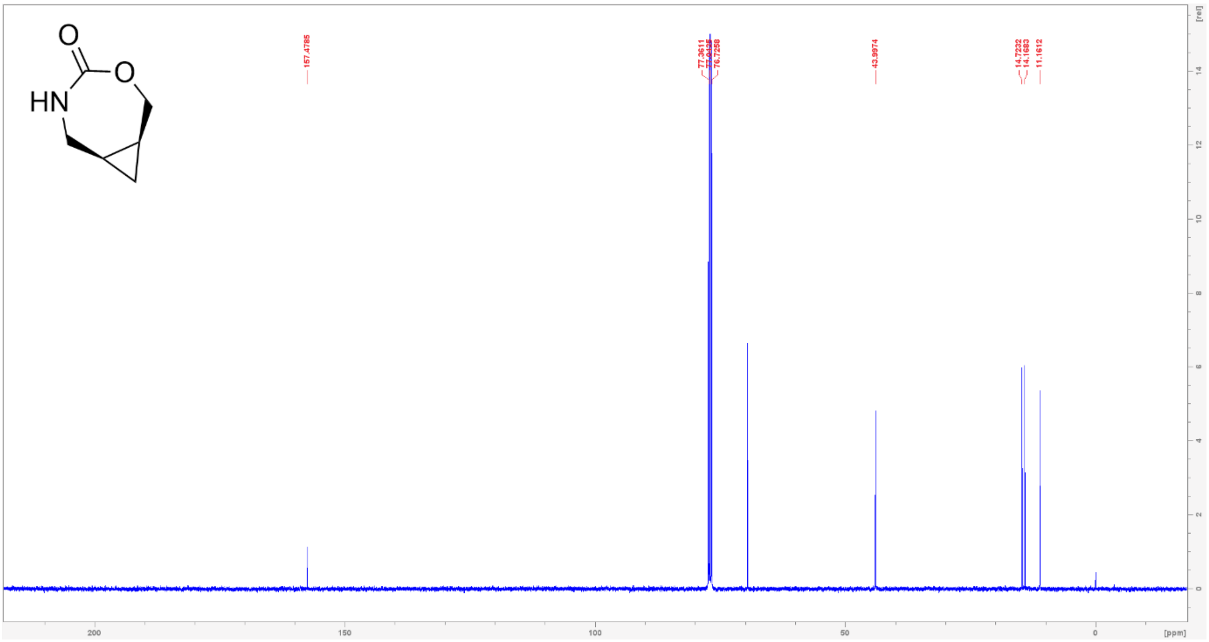

[13]C{[1]H} Spectrum of compound **2o** in $CDCl_3$ at 25°C. [1]H-frequency 400 MHz spectrometer.

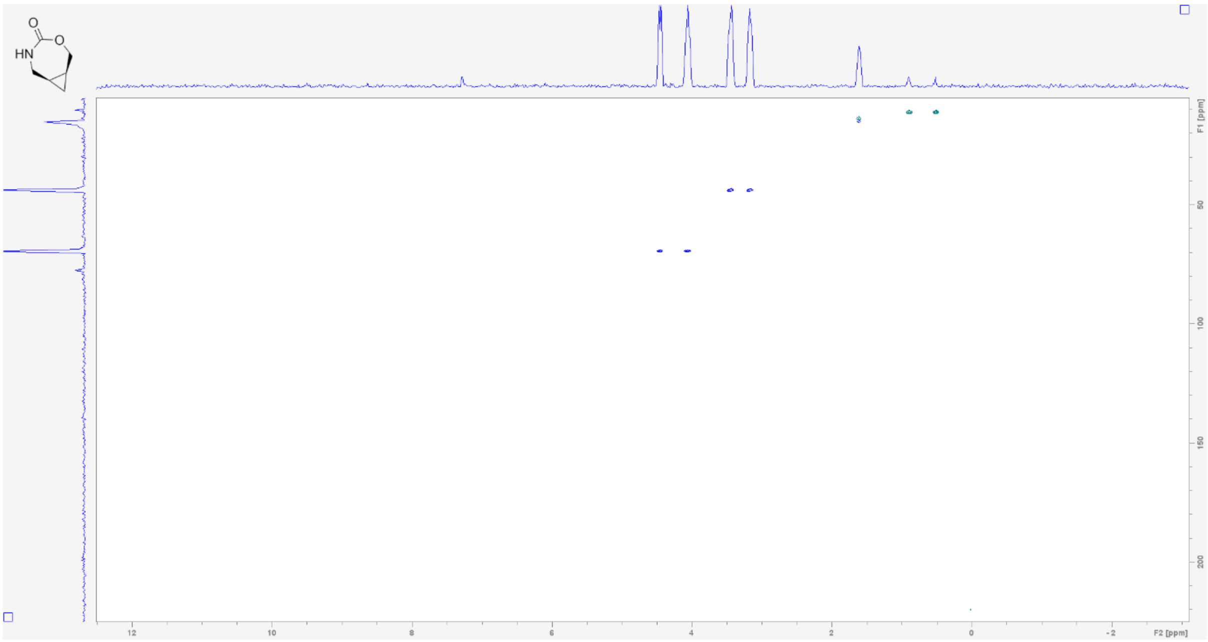

HSQC Spectrum of compound **2o** in $CDCl_3$ at 25°C. [1]H-frequency 400 MHz spectrometer.

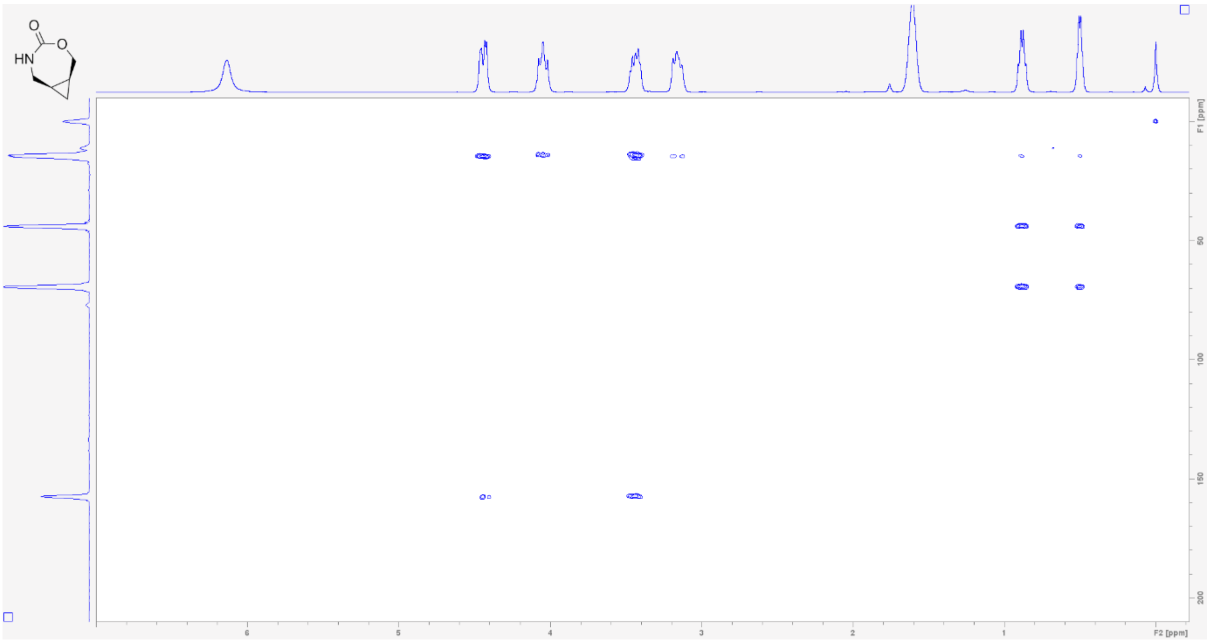

HMBC Spectrum of compound **2o** in $CDCl_3$ at 25°C. [1]H-frequency 400 MHz spectrometer.

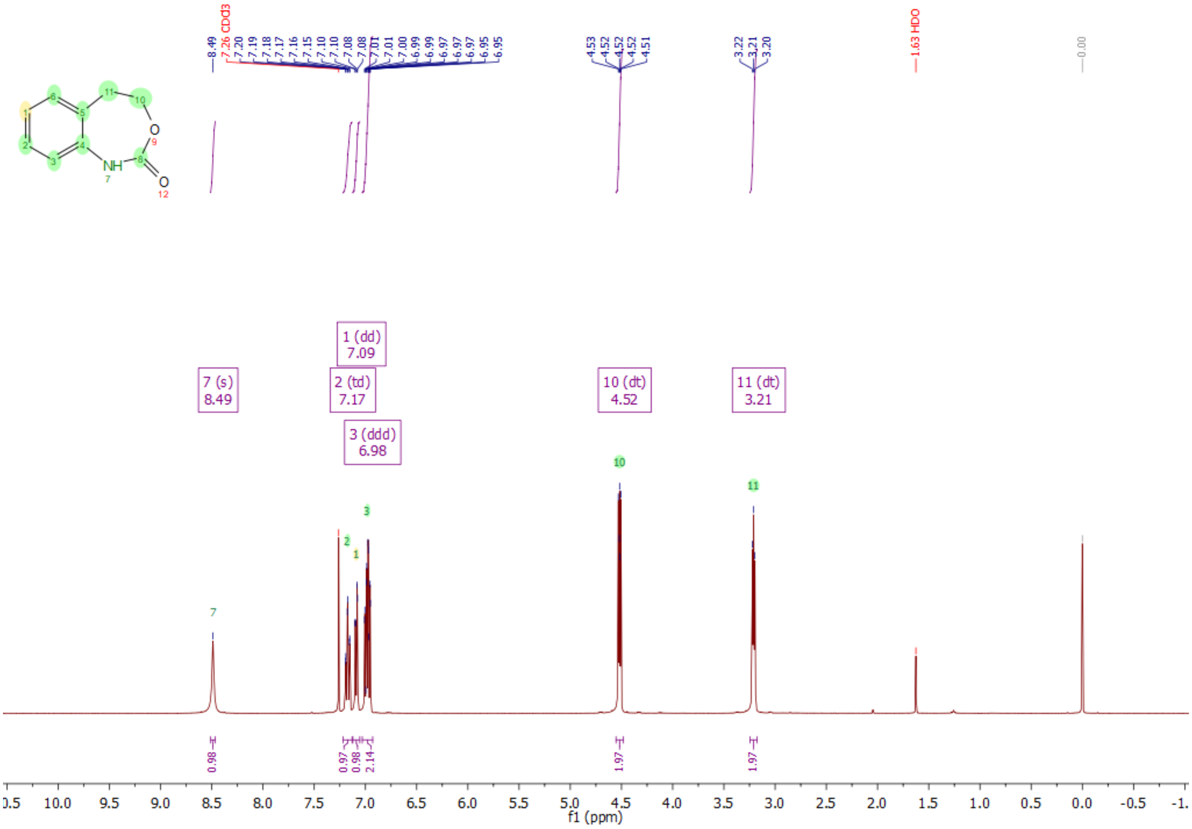

^{1}H Spectrum of compound **2p** in $CDCl_3$ at 25°C. ^{1}H-frequency 400 MHz spectrometer.

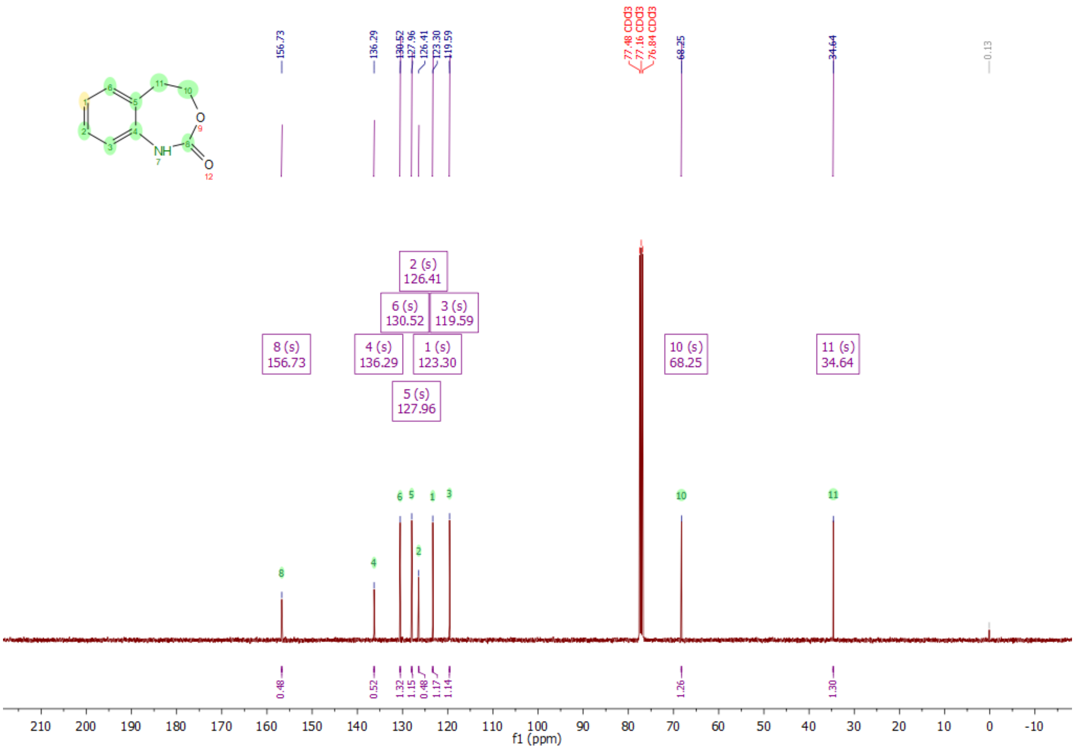

$^{13}C\{^{1}H\}$ Spectrum of compound **2p** in $CDCl_3$ at 25°C. ^{1}H-frequency 400 MHz spectrometer.

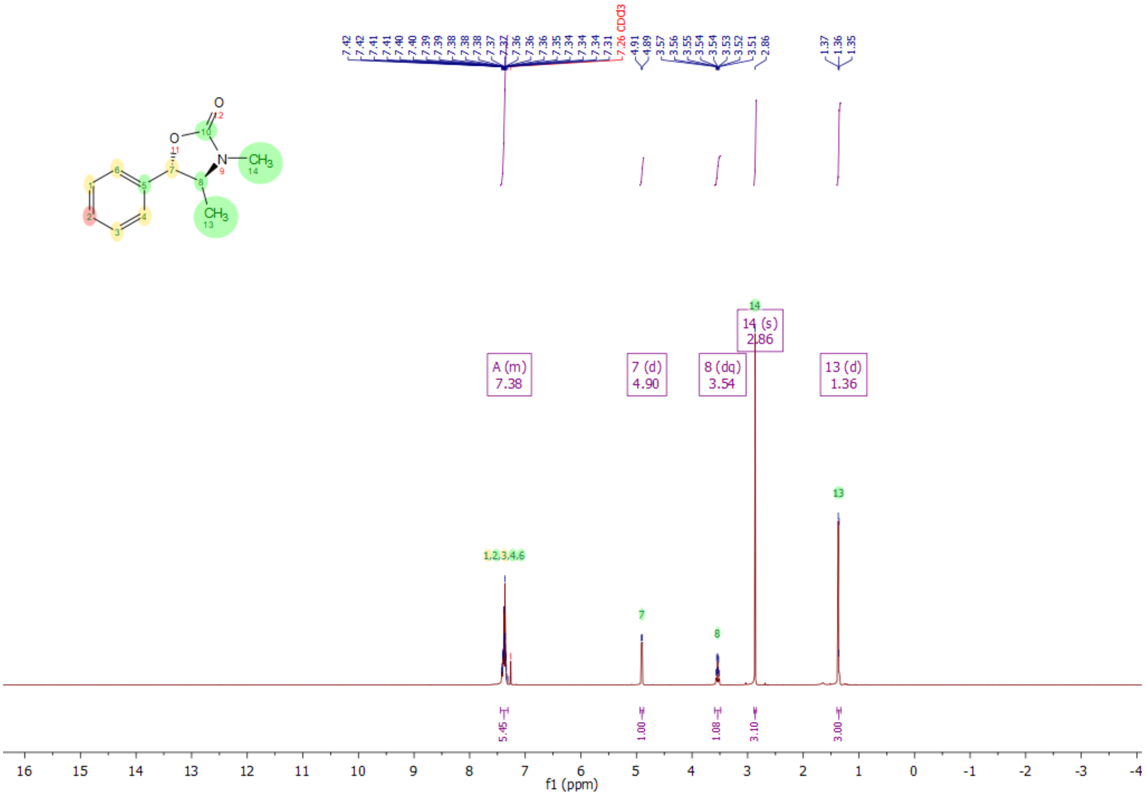

[1]H Spectrum of compound **2q** in $CDCl_3$ at 25°C. [1]H-frequency 400 MHz spectrometer.

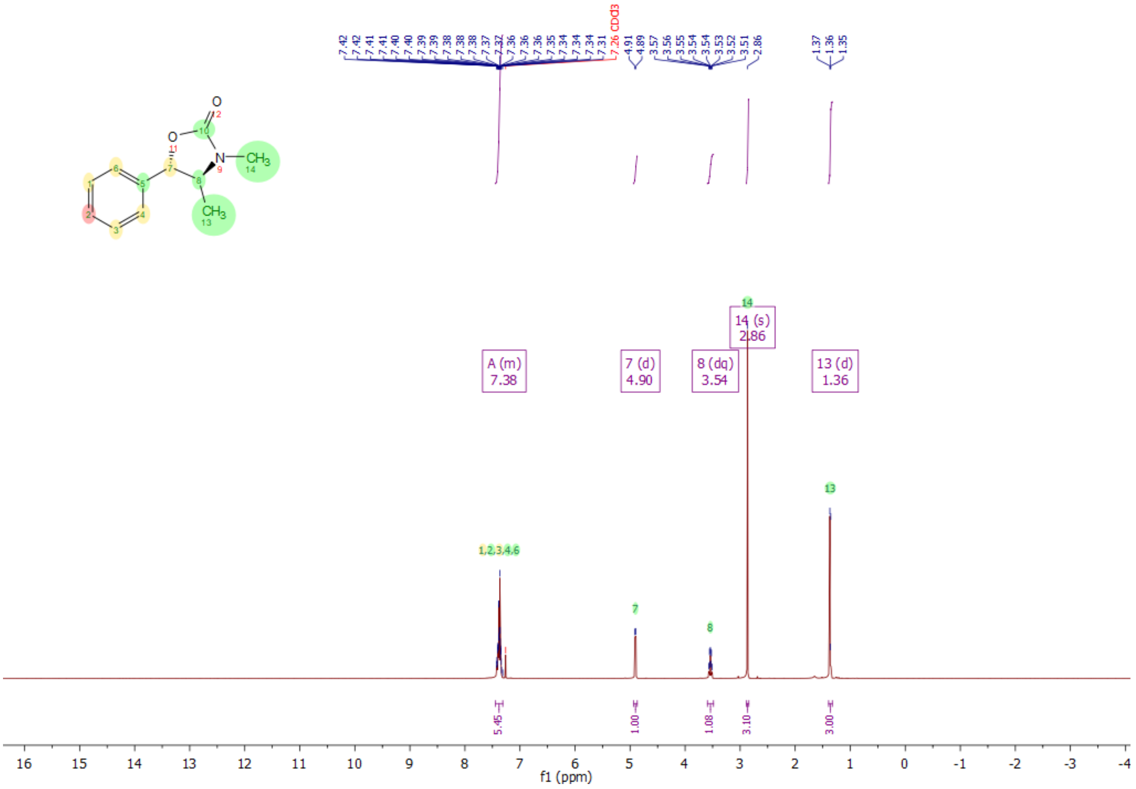

[1]H Spectrum of compound **2q** spiked with reference compound in $CDCl_3$ at 25°C. [1]H-frequency 400 MHz spectrometer.

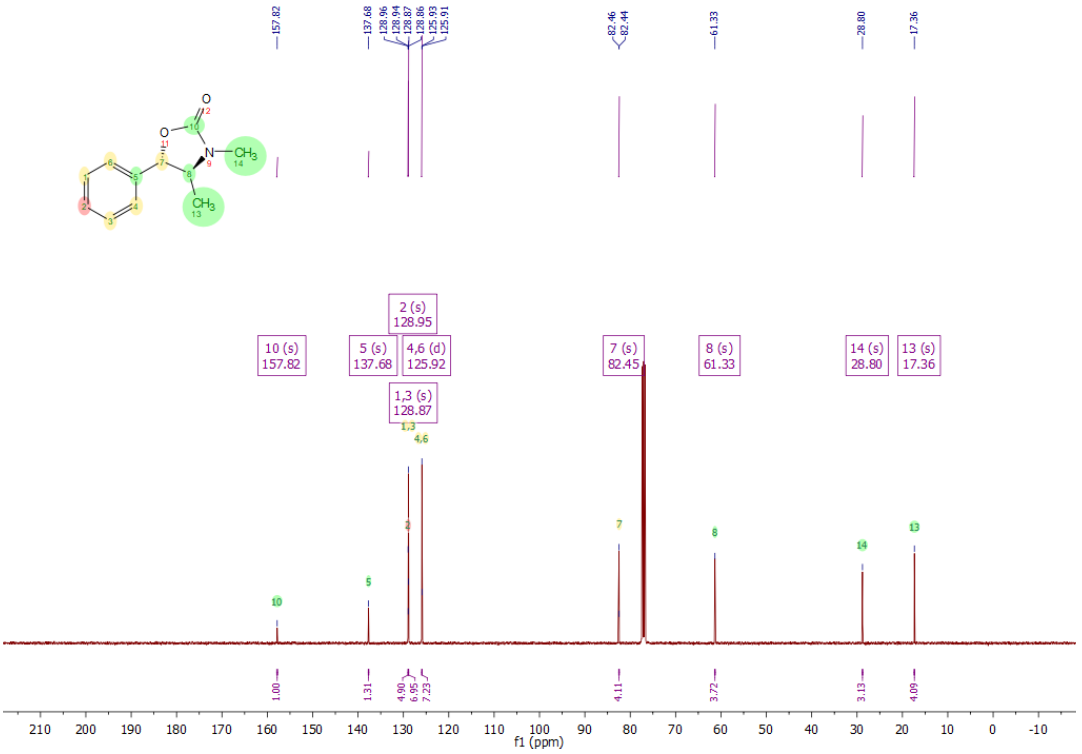

$^{13}C\{^{1}H\}$ Spectrum of compound **2q** in $CDCl_3$ at 25°C. ^{1}H-frequency 400 MHz spectrometer.

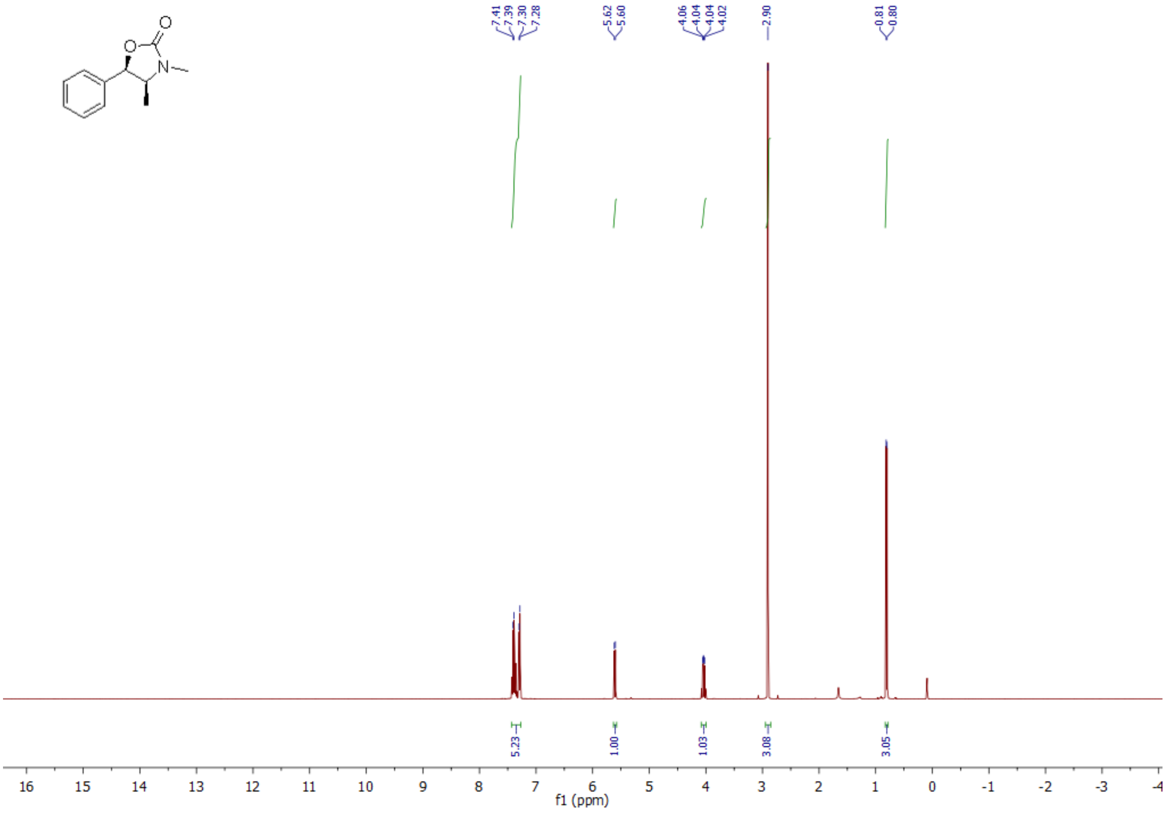

^{1}H Spectrum of compound **2r** in $CDCl_3$ at 25°C. ^{1}H-frequency 400 MHz spectrometer.

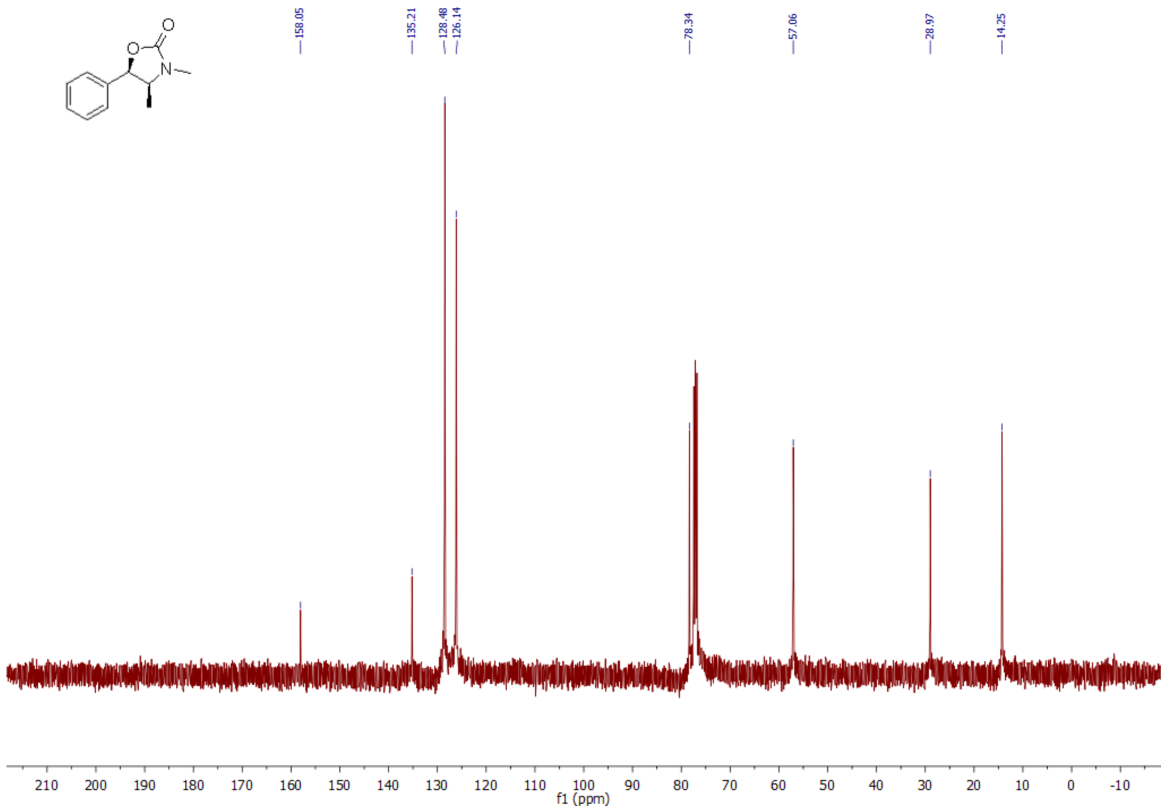

$^{13}C\{^{1}H\}$ Spectrum of compound **2q** in $CDCl_3$ at 25°C. ^{1}H-frequency 400 MHz spectrometer.

11. References

1 T. Hase, *Tables for Organic Spectrometry*, Otatieto, 10th edn., 2008.

2 C. Agami, F. Couty, L. Hamon and O. Venier, *Tetrahedron Lett*, 1993, **34**, 4509–4512.

3 T. Niemi, I. Fernández, B. Steadman, J. K. Mannisto and T. Repo, *Chemical Communications*, 2018, **54**, 3166–3169.

4 X. Li, J. Benet-Buchholz, E. C. Escudero-Adán and A. W. Kleij, *Angewandte Chemie International Edition*, 2023, **62**, e202217803.

5 C. A. Brooks, L. S. Barton, D. J. Behm, H. S. Eidam, R. M. Fox, M. Hammond, T. H. Hoang, D. A. Holt, M. A. Hilfiker, B. G. Lawhorn, J. R. Patterson, P. Stoy, T. J. Roethke, G. Ye, S. Zhao, K. S. Thorneloe, K. B. Goodman and M. Cheung, *ACS Med Chem Lett*, 2019, **10**, 1228–1233.

6 M. E. Jung and G. Piizzi, *Chem Rev*, 2005, **105**, 1735–1766.

7 J. Kaneti, A. J. Kirby, A. H. Koedjikov and I. G. Pojarlieff, *Org Biomol Chem*, 2004, **2**, 1098–1103.

8 J. A. S. Aho, J. K. Mannisto, S. P. M. Mattila, M. Hallamaa and J. Deska, *Journal of Organic Chemistry*, DOI:10.1021/acs.joc.4c02645.

9 P. V. Kortunov, L. S. Baugh, M. Siskin and D. C. Calabro, *Energy and Fuels*, 2015, **29**, 5967–5989.

10 K. J. Padiya, S. Gavade, B. Kardile, M. Tiwari, S. Bajare, M. Mane, V. Gaware, S. Varghese, D. Harel and S. Kurhade, *Org Lett*, 2012, **14**, 2814–2817.

11 S. Cutugno, G. Martelli, L. Negro and D. Savoia, *European J Org Chem*, 2001, **2001**, 517–522.

12 C. Larrlvée-Aboussafy, B. P. Jones, K. E. Price, M. A. Hardink, R. W. McLaughlin, B. M. Lillie, J. M. Hawkins and R. Vaidyanathan, *Org Lett*, 2010, **12**, 324–327.

13 J. Paz, C. Pérez-Balado, B. Iglesias and L. Muñoz, *Journal of Organic Chemistry*, 2010, **75**, 3037–3046.
